# Transcriptomic time-series analysis of early development in olive from germinated embryos to juvenile tree

**DOI:** 10.1186/s12864-018-5232-6

**Published:** 2018-11-19

**Authors:** Jaime Jiménez-Ruiz, María de la O Leyva-Pérez, Isabel Vidoy-Mercado, Araceli Barceló, Francisco Luque

**Affiliations:** 1 Center for Advanced Studies in Olive Grove and Olive Oils, Department of Experimental Biology, University of Jaén, Campus de las Lagunillas s/n, 23071 Jaén, Spain; 2 Centro de Investigación y Formación Agraria de Churriana, Instituto de Investigación y Formación Agraria y Pesquera (IFAPA), Málaga, Spain

**Keywords:** Seedling development, Olive, Transcriptome, RNAseq, Time series analysis, Dormancy, Hormones

## Abstract

**Background:**

Despite its relevance, almost no studies account for the genetic control in the early stages of tree development, i.e. from germination on. This study seeks to make a quite complete transcriptome for olive development and to elucidate the dynamic regulation of the transcriptomic response during the early-juvenile period by RNAseq time-series expression analysis. The transcriptome was made from 342,049,597 paired-end reads of 101 bp in length. The assembled transcriptome contained 109,125 unigenes (N50 = 1490 bp, average length = 839).

**Results:**

The time-series-expression analysis showed that, embryonic structures present at the first month after the induction of germination reached a more differentiated state in two-month-old seedlings. Once the plants were between three and four months old and reached a size around 6–7 nodes, the first developmental stages appeared to be complete and the developing seedling became a juvenile plant. In addition, an AGL-gene was rapidly downregulated during the induction of germination. The repression of this gene was very strong, as evidenced by the low levels of gene expression during plant development from the embryonic seedling to undetectable levels of expression in the adult tree. These results suggest that this gene may be involved in seed dormancy and could be a repressor of the germination. Also, an *APL1*-like olive gene was found to be expressed at high levels during flowering, and was also expressed during the cold incubation in the activation of embryo germination, suggesting a probable role in embryonic development.

**Conclusions:**

The early development from germination to the juvenile stage of olive seedlings occurred when plants reached a size around 6–7 nodes, and general changes of relevant groups of genes involved in development are described. An AGL-gene was proposed to be involved in germination repression. An *APL1*-like gene was found to have a probable role in embryonic development.

**Electronic supplementary material:**

The online version of this article (10.1186/s12864-018-5232-6) contains supplementary material, which is available to authorized users.

## Background

Plant development is a complex process scarcely studied in woody plants. After seed germination, the seedling grows to become a sapling that later makes phase transitions from the juvenile stage to become an adult tree, which has acquired competence for reproduction, completing the life cycle in the reproductive phase. Despite its relevance, the genetic control of the early steps of tree development has received little attention. In practical terms, the study of the genetic changes in normal olive-tree development is relevant to breeders seeking to shorten the unproductive juvenile period [1]. Understanding the molecular basis of these characteristics and improving the efficiency of such breeding programs require the development of genomic information and tools. In previous work, we generated cDNA sequences from Sanger-sequencing and pyrosequencing 454 Roche technology to identify the whole transcriptome of olive by sequencing different juvenile and adult tissues from several olive-tree cultivars [2]. Their assembly and functional annotation facilitated the generation of an EST Olive Array to enable the first study of the juvenile-to-adult transition genetic control in *Olea europaea* [3]. In that study, the transcriptional changes that took place during development of trees 6 to 33 months old were analyzed by microarray hybridization. The study revealed that transcriptomic changes preceded the juvenile-to-adult phase transition and occurred when the tree trunk had developed between 30 and 45 nodes and at least 18 months prior to the first flowering.

In Arabidopsis, the main genetic changes at the juvenile-to-adult phase transition are controlled by a complementary expression pattern of the miRNAs, *miR156* and *miR172* [4, 5]. These miRNAs also control the vegetative phase change in trees [6]. Also, *miR156* and *miR172* have been found in olive [7], and target genes such as the *AP2-like* transcription-factor family appear to be regulated in the same way as in Arabidopsis [3]. However, before reaching the juvenile-to-adult transition, the seedling should have developed nearly 45 nodes, but studies are lacking on this period of olive-tree development and in woody plants in general. New olive transcriptomes have been published recently, regarding to a developmental process as it is the plant architecture [8], or in response to a biotic stress [9, 10]. Here, we report a transcriptional study of monthly samples from a number of seedlings from open pollinated olive trees cv. Arbequina over the first six months after germination. This study was aimed at developing a quite complete transcriptome dataset for olive development using RNAseq by Illumina and at studying the dynamic regulation of the transcriptomic response during the early-juvenile period by time-series-expression analysis.

## Methods

### Plant material

**Fig. 1:**
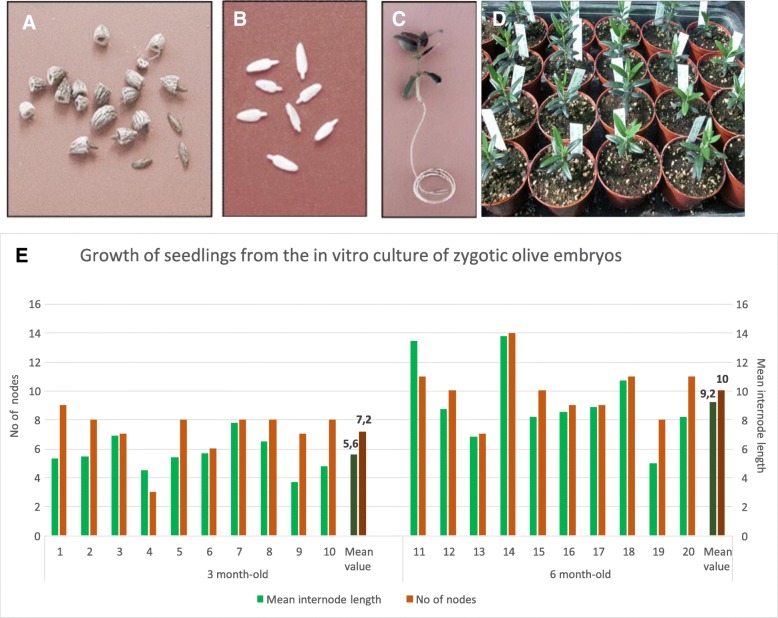
Plant material at different stages during the germination and growth. Seeds separated from mesocarp and endocarp (**a**); embryos extracted from seeds previously activated by water imbibition for 24 h (**b**); seedlings grown in vitro under chamber conditions until they were two months old (**c**); three-month-old seedlings in pots (**d**); growth data of seedlings from the in vitro culture of zygotic olive embryos at 3 and 6 months from induction of germination (**e**)

Olive fruits from the open pollinated olive cultivar Arbequina were collected and their seeds separated from the mesocarp (Fig. 1a). Seeds were induced to geminate by water imbibition for 24 h. Then, activated germ embryos were extracted from the seeds (Fig. 1b), cultivated in Murashige–Skoog medium [11], one-third-strength medium, and subjected to vernalization (13 °C) in the dark for two weeks. Seedlings were grown in vitro under chamber conditions with a 16-h photoperiod of fluorescent light and at 25 °C constant temperature until they were two months old (Fig. 1c). Seedlings were then potted (Fig. 1d), acclimatized, and allowed to grow in a conditioned greenhouse (25 °C) until they were six months old. Whole plants were collected at 1, 2, 3, 4, 5, and 6 months after seed activation (10 plants/time point), immediately frozen in liquid nitrogen and kept at − 80 °C until the extraction of the total RNA. Growth data (length and number of nodes per plant) were measured when plants were three and six months old (Fig. 1e).

### RNA sample preparation and next generation sequencing

Total RNA was purified from seeds at times 0 and 24 h of water imbibition, germinating embryos and seedlings less than one month old. For RNA isolation in older samples the roots and the shoots were first separated and total RNA taken from both parts separately. RNA was obtained using a Spectrum Plant Total RNA kit (Sigma-Aldrich, St. Louis, MO, USA) following the manufacturer’s instructions. Any DNA contamination was removed by DNase I treatment in a column (Roche, Basel, Switzerland). The RNA-quality tests were performed with the Agilent 2100 bioanalyzer (Agilent Technologies, Santa Clara, CA, USA) using an RNA 6000 Pico assay kit (Agilent Technologies). Equimolar amounts of RNA from each of 10 plants were pooled in two pools of 5 plants each. Then cDNA libraries were prepared, and the cDNA synthesis with the incorporation of dUTP within the second strand enabled the directional sequencing of all molecules while preserving the strand information of the RNA [12]. NGS sequencing was performed by GeneSystems (Valencia, Spain) with an Illumina HiSeq 2000 sequencer. Two biological replicates per sample were sequenced and each had two technical replicates sequenced on different lanes in the flow cell. The pool of the cDNA libraries was sequenced by paired-end sequencing (101 × 2) on an Illumina HiSeq 2000 sequencer.

### Data preprocessing

Preprocessing of the raw Illumina RNAseq reads was carried out by using Fastqmcf [13] to get reads with lengths greater than 50 bp and Q score of 30 or greater, and NGS_QC software [14] was used for quality control. Trimming and cleaning of the obtained reads was performed using the software Trimgalore (http://www.bioinformatics.babraham.ac.uk/projects/trim_galore/) [15].

### De novo assembly

The Trinity 1.3.3 software [16] version trinityrnaseq_r20140413p1 (http://github.com/trinityrnaseq/trinityrnaseq/wiki) was used for de novo assembly in ‘Picasso’ supercomputer (www.scbi.uma.es). The command line used were “Trinity --seqType fq --JM 1000G --left Sample1_R1.fastq Sample2_R1.fastq … --right Sample1_R2.fastq Sample2_R2.fastq … --SS_lib_type RF --CPU 80 --normalize_by_read_set”. Contigs shorter than 200 bases and any sequence with no best hit for a plant but for a possible contaminant were removed from the assembly using blast 2.2.20 software [17] with a minimum expectation value of 1e^− 5^, so 0.6% of transcripts (2216) were removed. The Transcriptome composition was then analyzed using Full-LengtherNext 0.0.8 (https://rubygems.org/gems/full_lengther_next; [18]). Full-LengtherNext (FLN) is able to classify unigenes to full-length, 5′- end, 3′-end and internal through comparing with its orthologs in the database. It also fixes frame shifts and suggests putative new genes, analyzing which genes classified as unknown are probably coding and which are putative non-coding RNA sequences. The putative new genes (showing coding gene structure) among the unknown transcripts were kept, but non-coding unknown sequences were removed. After applying this filter, more than one isoform were found for 26,393 genes. The expression analysis was simplified by selecting the longest isoform. GenoToolBox (https://github.com/aubombarely/GenoToolBox) was used for basic transcriptome stats and sequence filtering.

### Functional annotation and GO analysis

Sma3s [19] was used for functional annotation of the resulting transcriptome composed of 109,125 unigenes based on protein sequence similarity with UniProt-annotated plant sequences. The gene ontology (GO) terms retrieved by Sma3s were loaded in REVIGO software (http://revigo.irb.hr/; [20]).

GO analysis was carried out using Blast2GO software (https://www.blast2go.com) [21]. The gene-associated GO terms were loaded in the ‘Blast2GO’ interface and GO-term enrichment statistical analysis was performed. ‘Blast2GO’ integrated the Gossip package for statistical assessment of differences in GO-term abundance between two sets of sequences [22], using Fisher’s exact test and correcting for multiple testing. A one-tailed Fisher’s exact test was performed using a *P*-value with a filter value of < 0.05. Results were saved in a Microsoft Excel datasheet and charts were generated.

### Time-series expression analysis

The gene-expression study was analyzed with the DNAStar (ArrayStar 14) Qseq software for RNAseq analysis (www.dnastar.com). For mapping, we used parameters k-mer = 63 and 95% of matches and used the default-normalization method of ‘reads per kilobase per million mapped reads’, RPKM. For the time-series expression analysis the one-month time samples were compared to the rest of samples one by one. Any gene that showed an 8-fold change (8FC) at 95% of significance, in at least one of the comparisons was selected as a differentially expressed gene (DEG) during the early developmental stages of olive seedlings. A k-means analysis of the whole number of DEGs was later made.

### Reverse transcriptase quantitative polymerase-chain-reaction analysis

The real-time quantitative polymerase chain reaction (Q-RT-PCR) was performed as described in [9]. Briefly, first-strand cDNA was synthesized using the Transcriptor First-Strand cDNA Synthesis Kit (Roche, Basel, Switzerland) and amplifications using the master mix SsoFast EvaGreen Supermix (Bio-Rad Laboratories, Hercules, CA). Amplification of 10 ng of cDNA in 10 μL of reaction mixture were performed under the following conditions: A step of 95 °C for 30 s, followed by 40 cycles at 95 °C for 3 s, and at 60 °C for 7 s. At the end of the Q-RT-PCR it was added a melting step from 65 to 95 °C. Primers used for amplification are shown in Table 1. Three biological different pooled samples were analyzed at each time point by Q-RT-PCR.

**Table 1:** Primers for Q-RT-PCR

Unigene	Primer name	Primer sequence	Amplicon size (bp)	Primer design
27,994	27994F	5′ TGG ATC TAT TTC CTT TCA GAC GGT TCA GG 3′	165	This work
	27994R	5′ GAA GAA GGC CAA TAT CCA TCA ATG CAA G 3′		
65,000	65000F	5’ GCA TTT CAA TCT TAA TTT CCT CCA AAA ACA G 3′	161	This work
	65000R	5′ ATG CCT AAT TAC TGC TGA GCG TTG AGC 3’		
24,435	24435F	5’ TCT AAG CAG AAA AGA GGC AGA ATT TGT TCC 3’	140	This work
	24435R	5’ TCT GAA CAG AGT TGA CAC ACA AAC AAG AGC 3’		
33,184	33184F	5′ AAA TAT GCA GCA GTT AAA GCA AGA GAC AGC 3’	144	This work
	33184R	5’ CGC TCC AAC TGC TGT TCT AGC TGT TGT AG 3’		
73,363	73363F	5′ AAG AAT AGA GAA CAA GAT CAA TAG GCA GGT GAC 3’	140	This work
	73363R	5’ CGT ATA GCT TTC CAC GAC TGG AAA AGA TG 3’		
31,742	31742F	5′ TTG CCC TTT CAT TCA AGA AAC TGT TAA AGA G 3’	169	This work
	31742R	5’ CAT TCT GAT GGC AAG GAT TTA TGA TAA TTC C 3’		
61,861	61861F	5′ GAG AAG ATT CAG ATC AAG AAA ATC GAG AAC G 3’	162	This work
	61861R	5′ ATA CTC AAA GAG CTT GTC AGT GGA GGA GAA G 3’		
62,741	62741F	5’ CAT AAT TTT GCT TTG TGG AGC TGA TTT GC 3’	179	This work
	62741R	5′ TTA GCT TTC TTT AAC AGT CCT TTT CTT CTC TTG G 3’		
32,154	32154F	5’ CTA TAA AAT CCA ATG GAA AGG AAG CTG TCA C 3’	177	This work
	32154R	5′ GAA AGC TTT TAG CAT CAA TGT TCA TAT TTT GG 3’		
11,388	11388F	5’ CCG AGT ACC GGG AAC AAA TTC TTG AG 3’	152	This work
	11388R	5′ TGA AGC CAA ATC AGA TAG ATT TGT GGT CTC 3’		
34,438	34438F	5′ AGG ACA AGG CAT TGC AAG ATG AAA ACA C 3’	201	This work
	34438R	5′ TGA TCA TCC ATG GAG GTA TTA CTG CAT TAG C 3’		
30,294	30294F	5’ TCA AGA AAA AGG AAT CCG AAT TAA GGG AAG 3’	184	[3]
	30294R	5’ GCT CGA TTA TAC CAT TTC TTC TGC CTT CTG 3′		
Actin	Actin40370F	5’ CCC TTG ACT ATG AGC AGG AGC TTG AGA C 3′	143	[3]
	Actin40370R	5′ GAT CAT TGA AGG CTG GTA AAG AAC CTC AG 3′		

## Results

### Seedling growth

The size of the seedlings was obtained at three and six months from the induction of germination. The three-month-old seedlings had an average of 7.2 nodes (ranging from 3 to 9) and 5.6 cm of internode length, and 9/10 seedlings had 6 or more nodes. The 6-month-old seedlings had an average of 10 nodes (ranging from 7 to 14) with 9.2 cm of internode length.

### MRNA profile during the seedling growth of identified genes coding for transcription factors involved in plant development

**Fig. 2:**
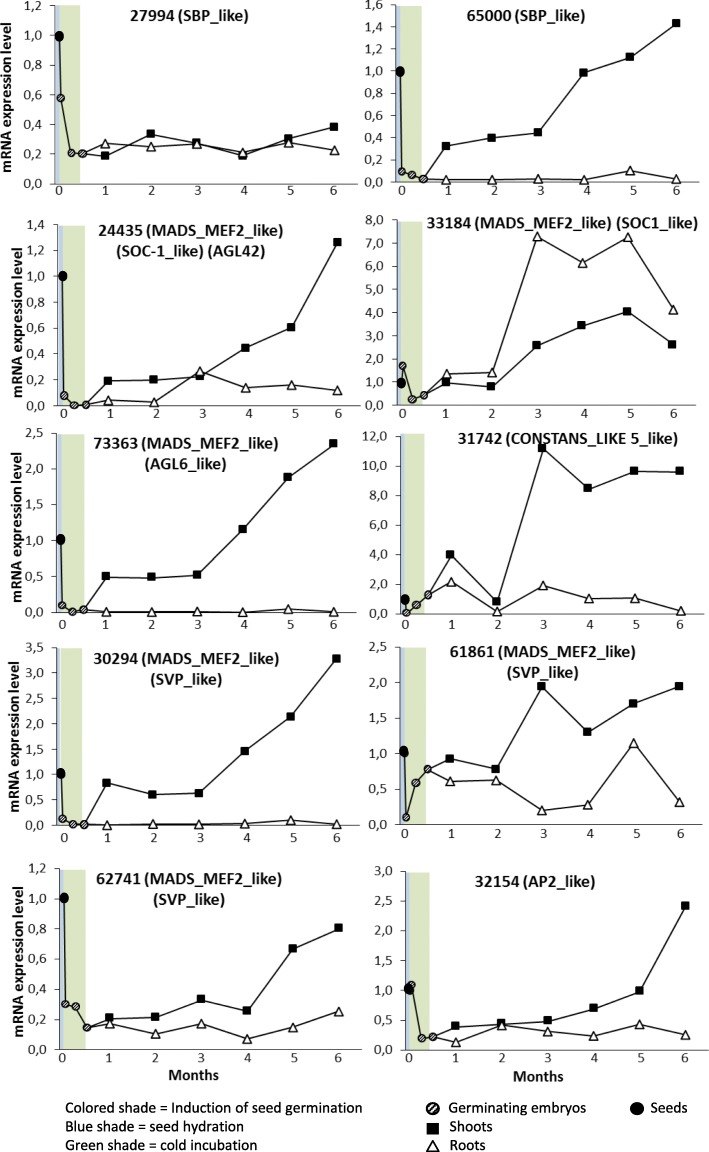
MRNA level of genes identified as likely involved in the olive-tree development during germination up to 6 months. Data represent the average of three samples taken from seed to 6-month-old juveniles, analyzed in triplicate by Q-RT-PCR

Samples were taken from the whole seed or activated germinating embryo in the first two weeks of plant growth. After the first month, the shoots and the roots were separated and RNA was sampled separately. At each time point of the study, the RNA from 10 seeds, germinating embryos or seedlings was pooled prior to analysis by Q-RT-PCR. The mRNA level was determined in seeds at time 0, before inducing germination; at 1 day, just after the water imbibition; in germinating embryos at one and two weeks during vernalization; and in seedlings every month up to the end of the follow-up at 6 month. Ten genes previously shown to code for olive transcription factors [2] and a gene previously identified as likely being involve in olive plant development (contig 30,294) [3] were analyzed for gene expression during early development (Fig. 2).

An apparent downregulation was detected during water imbibition, but this is due to the change from seed to embryo between times 0 to time 1. However, in contigs 33,184 (*SOC1*-like) and 32,154 (*APL2*-like) the down regulation occurred during the vernalization incubation. In general the genes were upregulated later during development, except for the contig 27,994 (*SBP*-like), which remained low for the 6 months of follow-up. The upregulation was detected in the shoots but not in the roots, except for contig 33,184 (*SOC1*-like), in which case the roots and shoots registered higher mRNA levels three months after germination. Two more genes, contigs 31,742 (*CONSTANS-LIKE 5*-like) and 61,861 (*SVP*-like), were upregulated after the first two months, just after the seedlings were potted. However, most genes were upregulated after the third month from germination, just when the plants reached a size of around 7 nodes or higher.

**Fig. 3:**
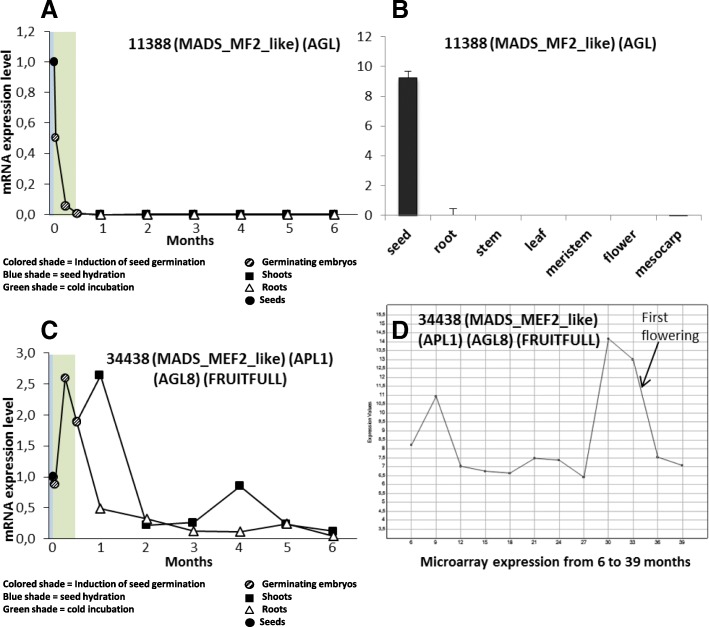
MRNA level analysis of a putative germination repressor gene and an APL1-like gene. The data represented in (**a**) and (**c**) are the average of three samples from seed to six-month-old juveniles, and from different tissues in (**b**). All samples were run in triplicate for Q-RT-PCR. Microarray data from 6-month-old to 39-month-old juveniles is represented in (**d**)

Of special interest, an *AGAMOUS*-like gene, contig 11,388, was strongly repressed during germination and its mRNA was undetected during the rest of the follow-up (Fig. 3a). The analysis of mRNA by Q-RT-PCR in different tissues showed that this gene was present only in seeds (Fig. 3b). The microarray data of meristems from plants 6 to 39 months old (available from [3]) confirmed that no expression of this gene was detectable during plant development except in dormant seeds (data not shown). Therefore, this gene could be involved in the regulation of seed dormancy, and its expression is repressed probably to allow the seed to be released from dormancy and to germinate. In addition, contig 34,438 (*AGL8*, *FRUITFULL*, *APL1*-like) was surprisingly upregulated during the seed germination and remained elevated for the first month, to be downregulated for the rest of the follow-up period (Fig. 3c). According to its annotation and the flowering-expression pattern observed in microarray transcriptomic study (Fig. 3d), this gene is likely part of the A genes, exhibiting a type-A function in the ABC model of flower-pattern formation. However, it must have another role during the germination process. The contig numbers, the DNA sequences, and the microarray data were obtained from material available in [3].

### RNAseq and transcriptome assembly

After trimming and cleaning, we obtained 342,049,597 paired-end reads of 101 bp length and 36.6 Phred quality (34.5 Gb). Trinity assembled all reads into 337,404 transcripts, and 26,393 unigenes were multi-isoform. After the removal of short contigs, contaminants, and non-coding transcripts, and after the selection of the longest isoform for clustered transcripts, the resulting transcriptome was composed of 109,125 unigenes (N50 = 1490 bp, average length = 839). A total number of 83,502 transcripts (76.52%) had an ortholog in Uniprot database of plant proteins. Sma3s software assigned functional annotations for 77,442 transcripts (67.3% of the transcriptome). The reads were mapped to this transcriptome for the time-series-expression analysis.

The RNAseq results were confirmed by a different technique —that is, the results of mRNA accumulation determined by Q-RT-PCR from the previous section were compared with RNAseq data. A great similarity of mRNA profiles was found in 8 of 10 unigenes and only partial differences in just two unigenes (Additional file 1: Figure S1).

### Time-series-expression analysis of RNAseq data

**Fig. 4:**
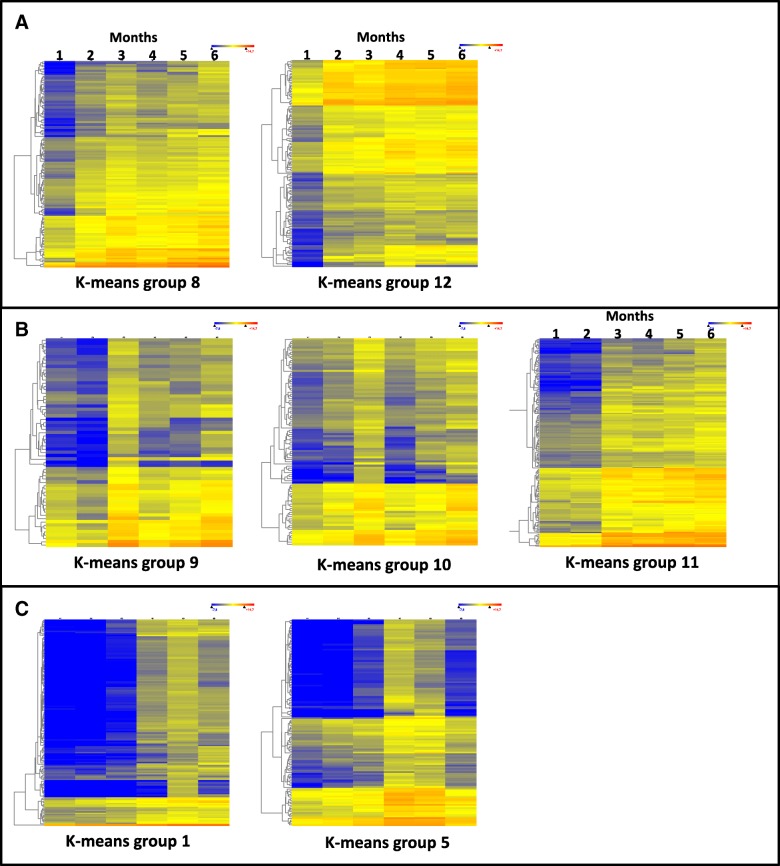
Heat maps of up-regulated genes included in k-means groups selected in the study. The k-means groups are included in the **a**, **b** and **c** clusters

**Fig. 5:**
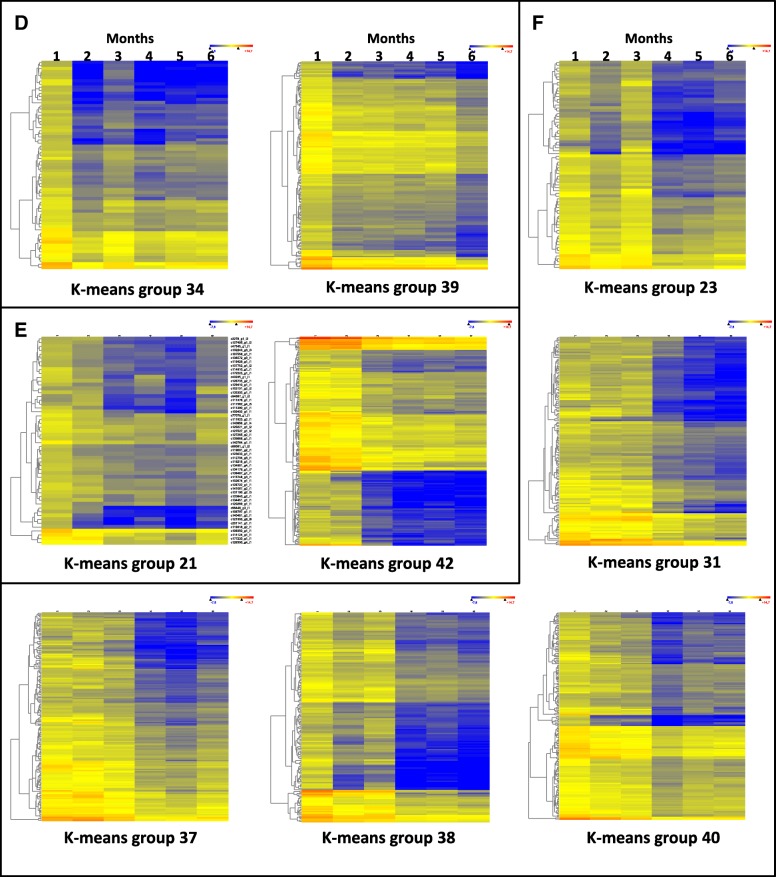
Heat maps of down-regulated genes included in k-means groups selected in the study. The k-means groups are included in the **d**, **e** and **f** clusters

The gene-expression data from the first time point (month one) was compared one by one with the rest of time points, from month two to six, and the genes that had at least a 8-fold change in any of the five comparisons with a 99% significance were selected. This analysis selected 4633 unigenes that were grouped according to their expression pattern in 42 k-mean groups (Additional file 2: Figure S2). For each group the enriched GO-terms were established and then subjected to a semantic association of Gene Ontology (GO) terms by a REVIGO analysis [20]. Of the k-means groups, 26 contained many GO terms related to stress responses but no GO terms related to plant development, and thus they were removed from the analysis. Seven of the 16 groups with relevant information about plant development corresponded to unigenes upregulated during the 6-month period studied (Fig. 4), and 9 corresponded to downregulated unigenes (Fig. 5). Additionally, k-means groups with similar patterns were grouped into clusters and the results of REVIGO analysis are shown in (Additional file 3: Table S1).

Cluster A was composed of groups 8 (133 unigenes) and 12 (136 unigenes) (Fig. 4a). In this cluster gene-expression increased over the six months of follow-up and, according to the REVIGO analysis, there were over-represented GO terms related to plant development such as: plant-type secondary cell wall biogenesis and cell wall organization and biogenesis; lignin catabolic process (biosynthetic); regulation of vernalization; formation of plant organ boundary; plant organ development; development maturation; multicellular organism growth; leaf pavement cell development; epidermal cell differentiation; negative regulation of multicellular organismal process (basically, this category contains GO terms related to breaking of seed dormancy); regulation of gene expression by genetic imprinting; genetic imprinting; DNA methylation on cytosine within a CG sequence; maintenance of DNA methylation; and response to cytokinin (including response to gibberellin and other hormones).

Cluster B was composed of groups 9 (63 unigenes), 10 (98 unigenes), and 11 (173 unigenes) (Fig. 4b). In this cluster, gene expression was upregulated after two months from onset of germination, just after the seedlings were potted. In this cluster the over-represented REVIGO GO terms related to plant development referred to: shoot system development; plant-type secondary cell wall biogenesis; and cell wall organization and biogenesis; secondary metabolite biosynthetic process (including regulation of lignin biosynthetic process); positive regulation of developmental process, positive regulation of multicellular organismal process; plant septum development; development maturation; tissue development, leaf pavement cell development; positive regulation of stomatal complex development; regionalization (stomatal complex paterning); formation of plant organ boundary; phloem or xylem histogenesis; vernalization response; fruit ripening (this large category containing GO terms related to seed dormancy and leaf senescence); negative regulation of multicellular organismal process (this category basically containing GO terms related to the breakage of seed dormancy; acropetal auxin transport (this category being related mainly to GO terms of IAA biosynthesis and metabolism); ethylene biosynthetic process; response to gibberellins; regulation of hormone biosynthetic process (a large category including biosynthetic processes of brassinosteroids and auxins); regulation of steroid metabolic process (especially including terms of abscisic acid biosynthetic process); cellular response to hormone stimuli and lipids (two large categories including many terms related to cellular response to hormone stimuli), cell-cell signaling involved in cell fate commitment (a large category with many terms of epidermis development); and negative regulation of cell differentiation (a large category of extremely diverse GO terms).

Cluster C was made up of groups 1 (146 unigenes) and 5 (126 unigenes) (Fig. 4c), and corresponded to upregulated genes after three months from germination. The REVIGO GO terms were over-represented and related to: plant development in this cluster regulation of growth rate; organ growth; regulation of secondary growth; regulation of vernalization response; programmed cell death; cell death; negative regulation of seed dormancy (basically release of seed from dormancy); positive regulation of leaf senescence (a large category including other plant organ senescence and development terms); brassinosteroid metabolic process (category including GO terms related to flavone biosynthetic process and phytosteroid metabolic process); and isoprenoid metabolic process. This last REVIGO group contained a very large group of GO terms related to the metabolism of isoprenoids that are required in primary and secondary metabolic processes with function in photosynthesis (carotenoids, chlorophylls, and plastoquinone), respiration (ubiquinone), membrane fluidity (sterols), and regulation of growth and development by hormones such as cytokinins, brassinosteroids, gibberelins, abscisic acid, and strigolactones.

Cluster D was composed of groups 34 (67 unigenes) and 39 (142 unigenes) (Fig. 5d), and corresponds to genes down-regulated the first month from germination and, according to the REVIGO analysis, there were over-represented GO terms related to plant development such as: leaf shaping; plant septum development; internode patterning (including xylem and phloem pattern formation, embryonic root morphogenesis, and others); positive regulation of developmental growth; negative regulation of leaf senescence; tissue development; positive regulation of abscisic acid-activated signaling pathway (a large category representing GO terms of a different set of genes of abscisic signaling pathway to those expressed from the second month of cluster B); response to ethylene (this category also including response to other hormones as gibberellin and cytokinin, again corresponding to a different set of genes than in cluster B); and internode patterning (this category including GO terms related to xylem and phloem pattern formation and leaf vascular tissue pattern formation).

Cluster E was constituted of groups 21 (54 unigenes) and 42 (171 unigenes) (Fig. 5e), corresponding to genes that had been downregulated just after the first two months, when the seedlings were potted. According to the REVIGO analysis, there were over-represented GO terms related to: plant development as dormancy process; positive regulation of seed germination; negative regulation of programmed cell death; cell fate specification; specification of plant organ identity; developmental process and multicellular organismal process; negative regulation of ethylene-activated signaling pathway (a very large category of GO terms including many factors and positive response to gibberellin hormone, representing GO terms of a different set of genes of response to gibberellins to those expressed from the second month of cluster B); response to gibberellin (a small category reinforcing the previous category on the previous point); auxin metabolic process (again seeming to be a change in the set of genes involved in hormone regulation and/or transport); negative regulation of cytokinin-activated signaling pathway (same comment as for the previous points; isoprenoid biosynthetic process (again the same comment as for the previous points); cellular response to endogenous stimuli (most GO terms related to responses to different hormones; same comment as for previous points); M specification of plant organ identity (a category of GO terms indicating a switch-off of genes that had been working in the first two months of development, after the seedlings had been potted); release of seed from dormancy (this category including GO terms of repressed genes related to seed exit from dormancy in agreement with the negative regulation of these kinds of genes in Cluster A and B).

Cluster F contained groups 23 (73 unigenes), 31 (138 unigenes), 37 (165 unigenes), 38 (158 unigenes) and 40 (171 unigenes), corresponding to genes that were downregulated after the first three months following germination induction. This time, three months of growth appeared to be important because there were many changes in the expression of genes related to development, and therefore, according to the REVIGO analysis, there were over-represented GO terms related to plant development such as: negative regulation of stomatal complex development (the name of this category of GO terms being a bit confusing because it includes GO terms such as stomatal complex morphogenesis, guard cell differentiation or regulation of stomatal complex development), and positive regulation of stomatal complex development (a large category including GO terms related to development); regulation of secondary growth; lateral growth; cell wall modification involved in multidimensional cell growth; growth; cell death; leaf vascular tissue pattern formation; embryonic morphogenesis (including post-embryonic plant morphogenesis), formation of plant organ boundary (this category including shoot system morphogenesis activated one month earlier, [Cluster B], and a pattern of increasing expression through the 6 months of follow-up [Cluster A], vegetative to reproductive phase transition of meristem and regulation of timing of transition from vegetative to reproductive phase (two categories with a large number of GO terms mostly related to reproduction or development); embryonic meristem development; cell fate specification; apical cell fate commitment; cell differentiation; centrolateral axis specification; seed germination (including seedling development); response to gibberellin and response to auxin (these categories affecting other hormones and thus causing a strong response to hormones in the third month that ended in the fourth); cellular response to hormone stimuli; negative regulation of response to stimuli (in agreement with the previous comment); regulation of developmental process (with genes involved in development being repressed after the third month of growth); plant organ morphogenesis and anatomical structure morphogenesis (a very large category that is consistent with the previous comments); and development process.

## Discussion

Tree development is a poorly studied process. Phase change in woody perennials is associated with the plant reaching a certain size [23]. In olive trees, the size to change from juvenile to adult tree is around 30–45 nodes [3]. However, prior to this phase transition the germinating seedling must develop into a juvenile tree, and this is the developmental process analyzed by a transcriptomic approach in this work.

The results of expression analysis of genes coding for transcription factors identified as likely being involved in plant development, indicated a change in gene expression two months after induction of germination (IG), which coincided with the transplanting of the seedlings to pots. Probably more significant was the change in gene expression after the first three months of growth. In fact, the analysis of the RNAseq data by time series showed that in the first three months there are major changes in expression of genes involved in plant development. Meanwhile, after this three-month period, the gene expression of many plant development related genes became substantially more stable, indicating that the development of the juvenile tree from the seed was completed and that the juvenile entered a growth period that would eventually transition to the adult phase. The three-month-old plants averaged 7.2 nodes per plant, most having 6 or more nodes. Therefore, 6–7 nodes appear to mark the size at which the seedling becomes a juvenile tree.

**Fig. 6:**
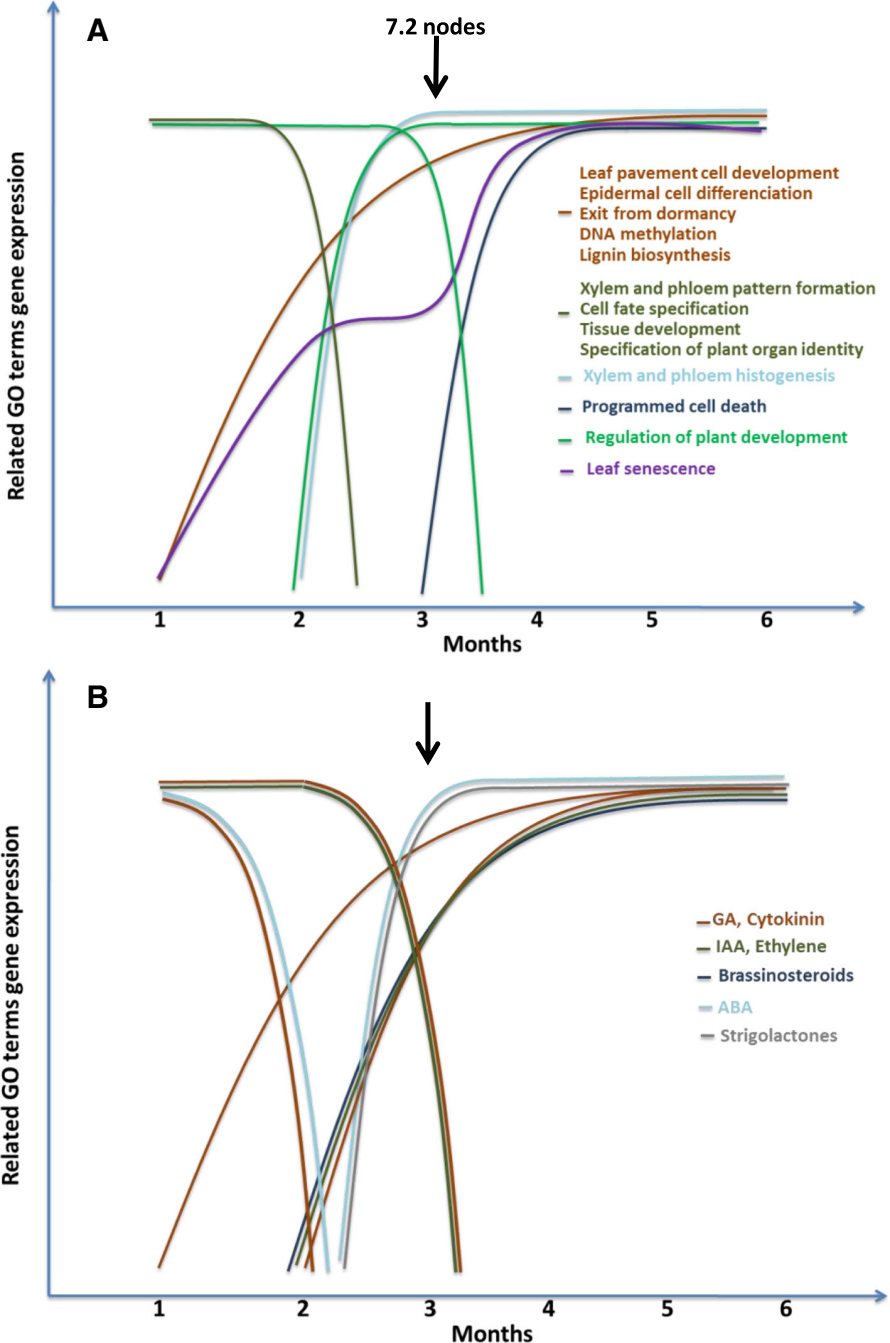
A model for gene expression of GO terms related to hormone regulation and plant development during the germination to the juvenile tree developmental process. Plant development (**a**); plant-hormone biosynthesis and regulated genes (**b**)

Due to the high complexity of the changes of gene expression in GO terms related to plant development, a model of the main changes was built (Fig. 6). In fact, the model is separated into two classes, genes involved directly in plant development (Fig. 6a) and genes involved in hormone biosynthesis or genes regulated by hormones (Fig. 6b). There are genes that increase their expression during the six-month follow-up, as is the case of genes involved in DNA methylation, first increasing de novo DNA methylation and then its maintenance. This is consistent with the fact that one-month-old samples had embryonic structures; in fact, the distinction between shoots and roots was not very clear. In the samples two-month-old and older, structures became more defined and cell differentiation increased, so that DNA methylation must take place as cells differentiate. Thus it is consistent with the other type of genes that increase their expression with the same pattern, as genes for leaf pavement cell development and epidermal cell differentiation. In addition, in this same pattern the genes involved in release of dormancy increased as do the lignin biosynthesis genes. At the second month, the genes for xylem and phloem pattern formation were downregulated while the xylem and phloem histogenesis genes were induced and maintained high throughout the follow-up period. Simultaneously, genes for cell fate specification, tissue development, and specification of plant organ identity are also repressed. These results indicate that the main structures were established and the cells were committed to their fate by this time. At the second and third month of development, key changes of gene expression were found. Thus, a set of genes regulating plant development were induced, but one month later a different set of genes of the same GO term were repressed. Also at the second month after germination, leaf senescence genes were up-regulated and at the third month programmed cell death genes were upregulated, too. The programmed cell death is considered as the end point of leaf senescence but can also be involved in leaf maturation, so these results indicate that by this time the final stages in the development of certain major plant structures were complete.

The model for hormone-related genes is quite complex because most hormones are involved in different processes (Fig. 6b). Nevertheless, a key observation was that sets of genes under hormonal control or genes for hormone biosynthesis or degradation themselves were up- or downregulated during the first three months to finally reach a more stable state. This finding agrees with the observations made for the genes directly involved in plant development in Fig. 6a. Therefore, all the findings in this transcriptomic study point towards the conclusion of that embryonic development finishes when the seedling reaches a size of 6–7 nodes. At this time, all the plant structures and the cell and organ differentiation of a juvenile tree have developed. In addition to this finding, we detected sets of genes activated by gibberellin or abscisic acid (GA and ABA, respectively) in the transcriptome of germinating olives that are switched off after two or three months from IG. GA and ABA are involved in the germination of cereals by controlling in an opposing way the expression of a number of genes, for instance, starch degradation and the function of the endosperm after germination. Several hormones, such as GA, auxin (IAA), cytokinins, ethylene, and brassinosteroids, promote stem and/or leaf growth by stimulating cell elongation and/or division. In addition, we found that sets of genes regulated by different hormones are induced after the first two months, when the germinating process appears to finish and the plant begins a phase of vegetative growth.

In addition to the general examination of gene changes during the early stages of plant development, we focused special attention on two genes. First, an AGL-gene (contig_11,388) from the transcriptome described in [2] was found to be expressed only in seeds (Fig. 3a, b) and rapidly downregulated during the induction of the germination. The repression of this gene is very strong, and expression levels remain low throughout plant development from seedling emergence to the adult tree. These results suggest that this gene might be involved in seed dormancy and could be a repressor of germination. Further work is required to confirm this hypothesis. Finally, another gene (contig_34,438) described in an earlier transcriptome [2], according to its annotation and expression profile is likely the *APL1*-like gene of olive, a gene exhibiting type-A function during flower development (Fig. 3c, d). This gene is over-expressed during flowering, but we found that it was also expressed during the cold incubation in the activation of embryo germination. This expression declines at two months after IG, just when the results of this work indicate that the embryo cells were committed to their fate and the seedlings had determined the main structures. This finding indicates that the *APL1*-like gene in olive may play a role during the embryonic development.

## Conclusions

In conclusion, this work provides a transcriptomic analysis of the early stages of the olive-seedling development, revealing that, in the first month after the induction of the germination, embryonic structures appeared, but they reached a more differentiated state in two-month-old seedlings. Once the plants were between three and four months old and reached a size of around 6–7 nodes, the first developmental stages appeared to be complete and the seedling becomes a juvenile plant. In addition, a putative seed-dormancy transcription factor was identified and the *APL1*-like olive gene was also found to have a probable role in embryonic development.

## Additional files

Additional file 1: **Figure S1.** RNAseq validation by comparing the RNAseq mRNA profiles of 10 genes and the Q-RT-PCR from the same samples. (PPTX 175 kb)

Additional file 2: **Figure S2.** MRNA-level profile of the 42 k-means groups obtained from the time-series analysis. (PPTX 976 kb)

Additional file 3: **Table S1.** REVIGO analysis of the enriched GO-terms of the 42 k-means groups obtained from the time-series analysis. (PDF 1838 kb)

## Abbreviations

8FC: 8-fold change
DEG: Differentially expressed gene
FLN: Full-LengtherNext
GO: The gene ontology
NCBI: National Center for Biotechnology Information
Q-RT-PCR: The reverse transcriptase quantitative polymerase chain reaction
RPKM: Reads per kilobase per million mapped reads
SRA: Sequence Read Archive
