# Supplementary figures and images for: Transcriptomic time-series analysis of early development in olive from germinated embryos to juvenile tree
Source: BMC Genomics. 2018 Nov 19;19:824. doi: 10.1186/s12864-018-5232-6 (PMC6245754; doi:10.1186/s12864-018-5232-6)

## Slide 1
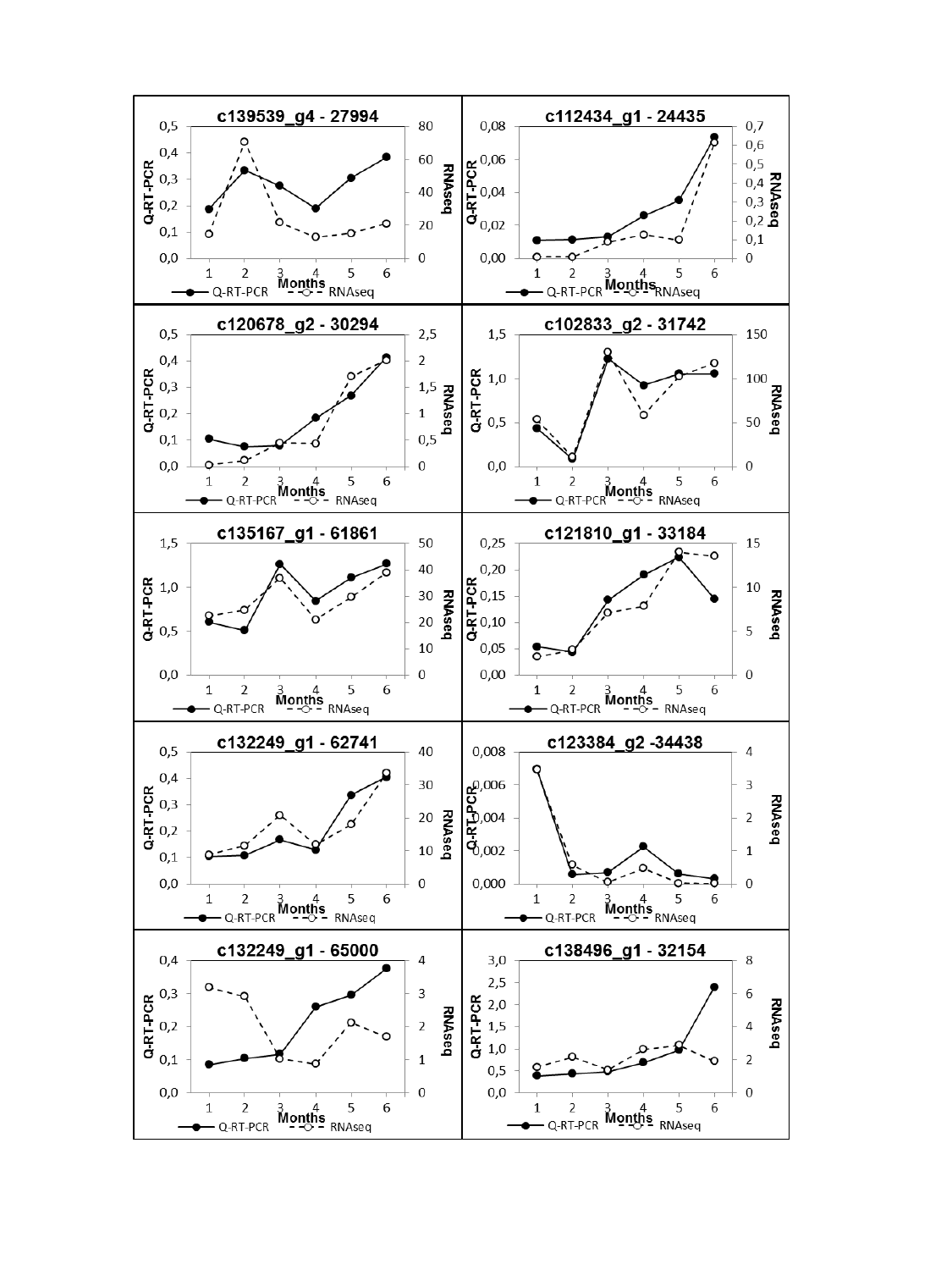

Supplement: Supplementary file 1 — Figure S1. RNAseq validation by comparing the RNAseq mRNA profiles of 10 genes and the Q-RT-PCR from the same samples. (PPTX 175 kb) [file 12864_2018_5232_MOESM1_ESM.pptx]

## Slide 1
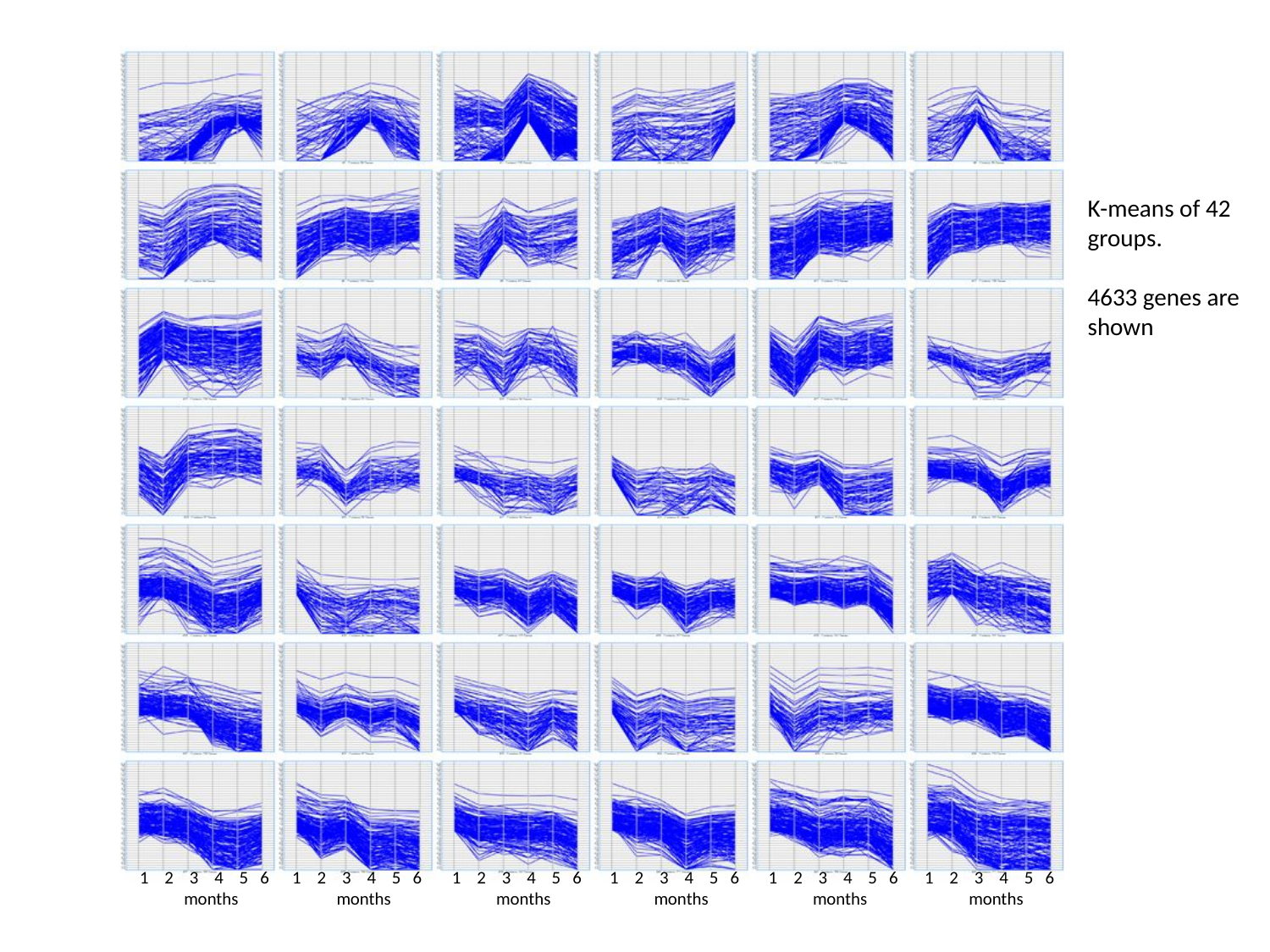

1 2 3 4 5 6
 months
1 2 3 4 5 6
 months
1 2 3 4 5 6
 months
1 2 3 4 5 6
 months
1 2 3 4 5 6
 months
1 2 3 4 5 6
 months
K-means of 42 groups.
4633 genes are shown

Supplement: Supplementary file 2 — Figure S2. MRNA-level profile of the 42 k-means groups obtained from the time-series analysis. (PPTX 976 kb) [file 12864_2018_5232_MOESM2_ESM.pptx]
