# Supplementary material for: Transcriptomic time-series analysis of early development in olive from germinated embryos to juvenile tree
Source: BMC Genomics. 2018 Nov 19;19:824. doi: 10.1186/s12864-018-5232-6 (PMC6245754; doi:10.1186/s12864-018-5232-6)
Supplement: Supplementary file 3 — Table S1. REVIGO analysis of the enriched GO-terms of the 42 k-means groups obtained from the time-series analysis. (PDF 1838 kb) [file 12864_2018_5232_MOESM3_ESM.pdf]

Biological Process (127) Tag Clouds

Scatterplot & Table Interactive Graph TreeMap

GROUP 1

Hide/show dispensable GO terms

Export results to text table (CSV)

Make R script for plotting

term ID	description	frequency	pin?	log_{10} p-value	uniqueness	dispensability
GO:0006412	translation	3.310 %		-13.0978	0.78	0.00
GO:0006518	*peptide metabolic process*	3.509 %		-12.6327	0.82	0.93
GO:0009059	*macromolecule biosynthetic process*	17.035 %		-6.9829	0.84	0.64
GO:0044249	*cellular biosynthetic process*	22.844 %		-3.7342	0.84	0.66
GO:0044271	*cellular nitrogen compound biosynthetic process*	17.018 %		-7.0979	0.82	0.64
GO:0044267	*cellular protein metabolic process*	17.229 %		-6.9904	0.85	0.52
GO:1901576	*organic substance biosynthetic process*	22.969 %		-3.7026	0.85	0.72
GO:0006414	*translational elongation*	0.363 %		-1.1741	0.82	0.73
GO:1901566	*organonitrogen compound biosynthetic process*	6.073 %		-8.1400	0.82	0.72
GO:0043604	*amide biosynthetic process*	3.591 %		-12.8673	0.81	0.93
GO:0034250	*positive regulation of cellular amide metabolic process*	0.095 %		-1.1874	0.71	0.69
GO:0043043	*peptide biosynthetic process*	3.328 %		-12.9538	0.80	0.95
GO:0045727	*positive regulation of translation*	0.095 %		-1.1874	0.67	0.63
GO:0034645	*cellular macromolecule biosynthetic process*	16.737 %		-7.1906	0.82	0.57
GO:0010269	response to selenium ion	0.009 %		-2.1888	0.92	0.00
GO:0040011	locomotion	0.164 %		-1.1133	0.98	0.00
GO:1900057	positive regulation of leaf senescence	0.039 %		-4.1221	0.63	0.00
GO:2000024	*regulation of leaf development*	0.224 %		-2.3532	0.65	0.75
GO:0023056	*positive regulation of signaling*	0.332 %		-1.3506	0.74	0.78

GO:0046620	regulation of organ growth	0.082 %	-1.2615	0.68	0.86
GO:0046622	positive regulation of organ growth	0.017 %	-1.6482	0.67	0.81
GO:0051240	positive regulation of multicellular organismal process	0.401 %	-3.6956	0.64	0.58
GO:0048831	regulation of shoot system development	0.850 %	-1.2431	0.62	0.75
GO:0048584	positive regulation of response to stimulus	0.902 %	-1.3801	0.71	0.59
GO:0048582	positive regulation of post-embryonic development	0.319 %	-1.1242	0.61	0.81
GO:0048827	phyllome development	1.921 %	-1.5042	0.73	0.78
GO:0048367	shoot system development	3.599 %	-1.2700	0.73	0.64
GO:0048366	leaf development	1.368 %	-2.1799	0.73	0.64
GO:0010647	positive regulation of cell communication	0.350 %	-1.3260	0.75	0.78
GO:0010150	leaf senescence	0.462 %	-3.4852	0.74	0.81
GO:0009787	regulation of abscisic acid-activated signaling pathway	0.350 %	-1.1351	0.69	0.78
GO:0048639	positive regulation of developmental growth	0.026 %	-1.6482	0.68	0.67
GO:0010338	leaf formation	0.022 %	-1.1741	0.79	0.52
GO:0090693	plant organ senescence	0.462 %	-3.4816	0.75	0.96
GO:0051094	positive regulation of developmental process	0.436 %	-3.5291	0.66	0.54
GO:0009967	positive regulation of signal transduction	0.332 %	-1.3506	0.67	0.69
GO:0010030	positive regulation of seed germination	0.069 %	-1.0808	0.65	0.84
GO:0009939	positive regulation of gibberellic acid mediated signaling pathway	0.026 %	-1.4813	0.70	0.81
GO:1901419	regulation of response to alcohol	0.350 %	-1.1351	0.76	0.69
GO:1900055	regulation of leaf senescence	0.104 %	-2.6529	0.66	0.87
GO:0010111	glyoxysome organization	0.004 %	-1.6482	0.96	0.03
GO:0043335	protein unfolding	0.004 %	-1.6482	0.97	0.03
GO:0000060	protein import into nucleus, translocation	0.039 %	-1.7929	0.91	0.06
GO:0007338	single fertilization	0.060 %	-1.3727	0.89	0.07
GO:0010183	pollen tube guidance	0.117 %	-1.2228	0.70	0.60
GO:0050918	positive chemotaxis	0.117 %	-1.2228	0.87	1.00
GO:0010483	pollen tube reception	0.026 %	-1.2697	0.84	0.52
GO:0042330	taxis	0.117 %	-1.2228	0.88	0.95
GO:0006935	chemotaxis	0.117 %	-1.2228	0.87	1.00
GO:0001887	selenium compound metabolic process	0.017 %	-1.6091	0.95	0.07
GO:0009058	biosynthetic process	24.432 %	-3.7868	0.92	0.11
GO:0043462	regulation of ATPase activity	0.026 %	-1.5248	0.85	0.11
GO:0016131	brassinosteroid metabolic process	0.177 %	-1.1306	0.78	0.13
GO:0051555	flavonol biosynthetic process	0.078 %	-1.1306	0.82	0.64
GO:0051553	flavone biosynthetic process	0.082 %	-1.1306	0.83	0.98
GO:0051554	flavonol metabolic process	0.082 %	-1.1248	0.84	0.99
GO:0051552	flavone metabolic process	0.091 %	-1.1248	0.86	0.77
GO:0016128	phytosteroid metabolic process	0.194 %	-1.1306	0.85	0.86
GO:0040009	regulation of growth rate	0.013 %	-1.7144	0.85	0.13
GO:0009644	response to high light intensity	0.324 %	-1.8385	0.90	0.13
GO:0009642	response to light intensity	0.587 %	-1.3969	0.89	0.64
GO:0006807	nitrogen compound metabolic process	27.303 %	-1.4506	0.92	0.19
GO:0000184	nuclear-transcribed mRNA catabolic process, nonsense-mediated decay	0.073 %	-1.5098	0.90	0.19
GO:0048518	positive regulation of biological process	4.398 %	-2.2168	0.82	0.19
GO:0010266	response to vitamin B1	0.009 %	-1.8891	0.89	0.20
GO:0033273	response to vitamin	0.026 %	-1.4813	0.89	0.87
GO:0007584	response to nutrient	0.047 %	-1.0605	0.89	0.59
GO:0018283	iron incorporation into metallo-sulfur cluster	0.009 %	-2.4891	0.88	0.23
GO:0006534	cysteine metabolic process	0.134 %	-1.0556	0.82	0.53
GO:0018282	metal incorporation into metallo-sulfur cluster	0.009 %	-2.4891	0.91	0.70
GO:0010467	gene expression	18.304 %	-6.8895	0.89	0.23
GO:0010076	maintenance of floral meristem identity	0.026 %	-1.6282	0.80	0.25
GO:0010582	floral meristem determinacy	0.052 %	-1.1874	0.75	0.71
GO:0010073	meristem maintenance	0.427 %	-1.0945	0.77	0.67
GO:0010077	maintenance of inflorescence meristem identity	0.039 %	-1.4953	0.80	0.80
GO:0010022	meristem determinacy	0.056 %	-1.1874	0.79	0.72
GO:0006817	phosphate ion transport	0.160 %	-1.1487	0.92	0.27
GO:0043170	macromolecule metabolic process	38.032 %	-1.2666	0.90	0.27
GO:0007568	aging	0.544 %	-2.9319	0.81	0.28
GO:0043603	cellular amide metabolic process	3.897 %	-12.3510	0.88	0.31
GO:0007031	peroxisome organization	0.147 %	-1.0053	0.95	0.31
GO:0009955	adaxial/abaxial pattern specification	0.151 %	-1.9295	0.81	0.32
GO:0010219	regulation of vernalization response	0.022 %	-1.8226	0.78	0.33
GO:0010220	positive regulation of vernalization response	0.013 %	-1.8226	0.74	0.84
GO:1901564	organonitrogen compound metabolic process	8.541 %	-6.2337	0.88	0.33
GO:0019538	protein metabolic process	19.012 %	-6.6363	0.89	0.36
GO:0071294	cellular response to zinc ion	0.025 %	-2.1888	0.89	0.36
GO:0009814	defense response, incompatible interaction	0.725 %	-1.2992	0.88	0.36
GO:0048569	post-embryonic animal organ development	0.572 %	-1.8891	0.76	0.37
GO:0050793	regulation of developmental process	2.577 %	-1.6775	0.71	0.41
GO:0008284	positive regulation of cell proliferation	0.129 %	-1.0053	0.76	0.41
GO:0051703	intraspecies interaction between organisms	0.026 %	-1.2697	0.96	0.42

GO:0051239	regulation of multicellular organismal process	1.972 %	-1.6832	0.71	0.42
GO:0044260	cellular macromolecule metabolic process	35.028 %	-1.5940	0.86	0.44
GO:0042542	response to hydrogen peroxide	0.268 %	-1.0612	0.88	0.45
GO:0006635	fatty acid beta-oxidation	0.147 %	-1.0184	0.86	0.45
GO:0035265	organ growth	0.117 %	-1.1487	0.79	0.46
GO:1902039	negative regulation of seed dormancy process	0.009 %	-1.6912	0.64	0.46
GO:2000034	*regulation of seed maturation*	*0.060 %*	*-1.1487*	*0.67*	*0.83*
GO:2000033	*regulation of seed dormancy process*	*0.039 %*	*-1.4295*	*0.66*	*0.90*
GO:0048838	*release of seed from dormancy*	*0.009 %*	*-1.5248*	*0.78*	*0.70*
GO:2000692	*negative regulation of seed maturation*	*0.017 %*	*-1.5098*	*0.63*	*0.82*
GO:0097438	*exit from dormancy*	*0.009 %*	*-1.5248*	*0.83*	*0.70*
GO:0009266	response to temperature stimulus	2.287 %	-1.6799	0.89	0.49
GO:0010048	*vernalization response*	*0.065 %*	*-1.3835*	*0.89*	*0.64*
GO:0009409	*response to cold*	*1.580 %*	*-1.3809*	*0.87*	*0.59*
GO:0009408	*response to heat*	*0.859 %*	*-1.0598*	*0.88*	*0.83*
GO:0034641	cellular nitrogen compound metabolic process	25.404 %	-1.6787	0.85	0.49
GO:0006469	negative regulation of protein kinase activity	0.134 %	-1.4175	0.66	0.50
GO:0001933	*negative regulation of protein phosphorylation*	*0.134 %*	*-1.4175*	*0.69*	*0.99*
GO:0042326	*negative regulation of phosphorylation*	*0.138 %*	*-1.4059*	*0.72*	*0.97*
GO:0034763	*negative regulation of transmembrane transport*	*0.017 %*	*-1.4059*	*0.75*	*0.95*
GO:0010360	*negative regulation of anion channel activity*	*0.017 %*	*-1.4059*	*0.71*	*0.59*
GO:0034766	*negative regulation of ion transmembrane transport*	*0.017 %*	*-1.4059*	*0.74*	*1.00*
GO:1903960	*negative regulation of anion transmembrane transport*	*0.017 %*	*-1.4059*	*0.74*	*1.00*
GO:0045936	*negative regulation of phosphate metabolic process*	*0.164 %*	*-1.2535*	*0.72*	*0.99*
GO:0051051	*negative regulation of transport*	*0.026 %*	*-1.1676*	*0.78*	*0.60*
GO:0050790	*regulation of catalytic activity*	*2.257 %*	*-1.0531*	*0.79*	*0.70*
GO:0043086	*negative regulation of catalytic activity*	*1.019 %*	*-1.0967*	*0.79*	*0.86*
GO:0051348	*negative regulation of transferase activity*	*0.151 %*	*-1.4059*	*0.82*	*0.80*
GO:0032410	*negative regulation of transporter activity*	*0.017 %*	*-1.4059*	*0.72*	*0.95*
GO:0032413	*negative regulation of ion transmembrane transporter activity*	*0.017 %*	*-1.4059*	*0.71*	*1.00*
GO:1903792	*negative regulation of anion transport*	*0.017 %*	*-1.4059*	*0.74*	*0.95*
GO:0010563	*negative regulation of phosphorus metabolic process*	*0.164 %*	*-1.2535*	*0.73*	*0.79*
GO:0033673	*negative regulation of kinase activity*	*0.134 %*	*-1.4175*	*0.69*	*0.99*
GO:0043271	*negative regulation of ion transport*	*0.026 %*	*-1.1676*	*0.74*	*0.98*
GO:0031400	*negative regulation of protein modification process*	*0.220 %*	*-1.1426*	*0.71*	*0.78*

Biological Process (213) Tag Clouds

Scatterplot & Table Interactive Graph TreeMap

GROUP2

Tip: your resulting list of GO terms seems to be quite long. If you want to reduce it further, press the Back button in your browser and choose a different setting for the "allowed similarity" parameter.

Hide/show dispensable GO terms

Export results to text table (CSV)

Make R script for plotting

term ID	description	frequency	pin?	$\log_{10}$ p-value	uniqueness	dispensability
GO:0009833	plant-type primary cell wall biogenesis	0.091 %		-6.4235	0.77	0.00
GO:0000281	*mitotic cytokinesis*	*0.311 %*		*-4.3526*	*0.82*	*0.89*
GO:0000278	*mitotic cell cycle*	*1.152 %*		*-3.5151*	*0.81*	*0.80*
GO:0042546	*cell wall biogenesis*	*0.928 %*		*-2.8764*	*0.86*	*0.80*
GO:0042545	*cell wall modification*	*0.561 %*		*-1.3249*	*0.87*	*0.80*
GO:0044723	*single-organism carbohydrate metabolic process*	*2.283 %*		*-2.5454*	*0.72*	*0.75*
GO:0016051	*carbohydrate biosynthetic process*	*1.442 %*		*-2.8428*	*0.68*	*0.71*
GO:0000271	*polysaccharide biosynthetic process*	*0.868 %*		*-3.5696*	*0.67*	*0.83*
GO:0051274	*beta-glucan biosynthetic process*	*0.272 %*		*-5.2816*	*0.67*	*0.95*
GO:0051273	*beta-glucan metabolic process*	*0.393 %*		*-4.9337*	*0.80*	*0.82*
GO:0044264	*cellular polysaccharide metabolic process*	*1.187 %*		*-3.1054*	*0.79*	*0.86*
GO:0044262	*cellular carbohydrate metabolic process*	*1.817 %*		*-3.1647*	*0.81*	*0.60*
GO:0044036	*cell wall macromolecule metabolic process*	*0.665 %*		*-2.4746*	*0.83*	*0.77*
GO:0044042	*glucan metabolic process*	*0.962 %*		*-3.5696*	*0.81*	*0.75*
GO:0071669	*plant-type cell wall organization or biogenesis*	*1.152 %*		*-2.4716*	*0.87*	*0.82*

GO:0030244	cellulose biosynthetic process	0.216 %	-5.8983	0.67	0.93
GO:0030243	cellulose metabolic process	0.337 %	-5.4396	0.80	0.60
GO:0000910	cytokinesis	0.384 %	-4.0716	0.83	0.66
GO:0052386	cell wall thickening	0.134 %	-1.8907	0.87	0.69
GO:0009664	plant-type cell wall organization	0.652 %	-2.4383	0.86	0.87
GO:0045488	pectin metabolic process	0.647 %	-2.4274	0.82	0.83
GO:0061640	cytoskeleton-dependent cytokinesis	0.332 %	-4.1177	0.82	0.97
GO:0071555	cell wall organization	2.430 %	-3.5267	0.85	0.62
GO:0009250	glucan biosynthetic process	0.509 %	-4.4754	0.65	0.86
GO:0052545	callose localization	0.155 %	-1.8742	0.94	0.93
GO:0052546	cell wall pectin metabolic process	0.095 %	-3.7246	0.76	0.67
GO:0052542	defense response by callose deposition	0.091 %	-1.9607	0.81	0.95
GO:0052543	callose deposition in cell wall	0.117 %	-1.9868	0.83	0.96
GO:0052544	defense response by callose deposition in cell wall	0.069 %	-2.0748	0.71	0.74
GO:0009832	plant-type cell wall biogenesis	0.626 %	-3.0562	0.86	0.84
GO:0009834	plant-type secondary cell wall biogenesis	0.263 %	-4.2365	0.87	0.78
GO:0010400	rhamnogalacturonan I side chain metabolic process	0.009 %	-5.6548	0.79	0.76
GO:0010395	rhamnogalacturonan I metabolic process	0.013 %	-5.6548	0.78	0.55
GO:0010393	galacturonan metabolic process	0.652 %	-2.4059	0.82	0.80
GO:0006073	cellular glucan metabolic process	0.937 %	-3.5696	0.79	0.92
GO:0052482	defense response by cell wall thickening	0.069 %	-2.0748	0.74	0.91
GO:1903047	mitotic cell cycle process	1.083 %	-3.6795	0.81	0.79
GO:0022402	cell cycle process	1.713 %	-2.6281	0.81	0.84
GO:0034637	cellular carbohydrate biosynthetic process	0.915 %	-3.3287	0.65	0.89
GO:0005976	polysaccharide metabolic process	1.899 %	-2.5086	0.83	0.75
GO:0033692	cellular polysaccharide biosynthetic process	0.729 %	-3.7677	0.65	0.92
GO:0030104	water homeostasis	0.052 %	-2.6734	0.95	0.00
GO:0042592	homeostatic process	2.637 %	-1.0703	0.95	0.69
GO:0048878	chemical homeostasis	1.765 %	-1.4832	0.94	0.61
GO:0042742	defense response to bacterium	1.476 %	-4.7996	0.79	0.00
GO:0043207	response to external biotic stimulus	4.981 %	-2.6298	0.80	0.94
GO:0051707	response to other organism	4.968 %	-2.6298	0.79	0.92
GO:0031348	negative regulation of defense response	0.203 %	-1.9042	0.83	0.50
GO:0048585	negative regulation of response to stimulus	0.699 %	-1.1092	0.85	0.64
GO:0050832	defense response to fungus	2.236 %	-2.2372	0.78	0.81
GO:0009627	systemic acquired resistance	0.281 %	-1.6584	0.82	0.81
GO:0009620	response to fungus	2.521 %	-1.7311	0.81	0.83
GO:0009617	response to bacterium	1.748 %	-4.2242	0.82	0.69
GO:0098542	defense response to other organism	4.044 %	-3.2374	0.77	0.79
GO:0009814	defense response, incompatible interaction	0.725 %	-1.0015	0.80	0.76
GO:0009817	defense response to fungus, incompatible interaction	0.207 %	-1.9681	0.82	0.59
GO:0044699	single-organism process	41.709 %	-2.0243	0.99	0.00
GO:0050896	response to stimulus	25.943 %	-2.2591	0.99	0.00
GO:0051704	multi-organism process	3.362 %	-1.4786	0.99	0.00
GO:0080170	hydrogen peroxide transmembrane transport	0.022 %	-5.1423	0.96	0.00
GO:0042436	indole-containing compound catabolic process	0.039 %	-2.9290	0.84	0.03
GO:0071554	cell wall organization or biogenesis	3.168 %	-3.1867	0.95	0.04
GO:0005975	carbohydrate metabolic process	4.670 %	-2.0749	0.91	0.07
GO:0006091	generation of precursor metabolites and energy	1.459 %	-2.0432	0.91	0.07
GO:0010191	mucilage metabolic process	0.142 %	-1.0216	0.92	0.08
GO:0009765	photosynthesis, light harvesting	0.160 %	-2.1409	0.91	0.08
GO:0009768	photosynthesis, light harvesting in photosystem I	0.095 %	-1.7448	0.91	0.79
GO:0019684	photosynthesis, light reaction	0.505 %	-1.3600	0.90	0.77
GO:0010150	leaf senescence	0.462 %	-1.2497	0.91	0.08
GO:0090693	plant organ senescence	0.462 %	-1.2483	0.91	0.96
GO:0009812	flavonoid metabolic process	0.414 %	-1.4342	0.93	0.08
GO:0015979	photosynthesis	1.057 %	-1.0150	0.91	0.09
GO:1901615	organic hydroxy compound metabolic process	1.265 %	-1.2183	0.93	0.10
GO:1901135	carbohydrate derivative metabolic process	3.375 %	-1.4794	0.92	0.11
GO:0071941	nitrogen cycle metabolic process	0.207 %	-1.2808	0.93	0.13
GO:0044710	single-organism metabolic process	18.390 %	-4.0891	0.86	0.14
GO:0071366	cellular response to indolebutyric acid stimulus	0.009 %	-3.1460	0.82	0.15
GO:1901699	cellular response to nitrogen compound	0.470 %	-1.5526	0.78	0.86
GO:0080026	response to indolebutyric acid	0.022 %	-2.7002	0.84	0.54
GO:0071365	cellular response to auxin stimulus	0.932 %	-1.2090	0.77	0.61
GO:0071417	cellular response to organonitrogen compound	0.147 %	-2.4992	0.79	0.67
GO:0010243	response to organonitrogen compound	0.768 %	-1.5931	0.81	0.77
GO:2001057	reactive nitrogen species metabolic process	0.203 %	-1.2542	0.93	0.15
GO:0009058	biosynthetic process	24.432 %	-1.5900	0.92	0.16
GO:0010196	nonphotochemical quenching	0.043 %	-1.6170	0.87	0.17
GO:0009699	phenylpropanoid biosynthetic process	0.337 %	-3.3504	0.69	0.18
GO:0010116	positive regulation of abscisic acid biosynthetic process	0.017 %	-1.9033	0.69	0.54

GO:0010115	regulation of abscisic acid biosynthetic process	0.030 %	-1.3796	0.68	0.91
GO:1901141	regulation of lignin biosynthetic process	0.030 %	-3.0172	0.72	0.76
GO:1901617	organic hydroxy compound biosynthetic process	0.824 %	-1.7069	0.83	0.64
GO:0044550	secondary metabolite biosynthetic process	1.183 %	-2.6979	0.71	0.80
GO:1901599	(-)-pinoresinol biosynthetic process	0.009 %	-2.1574	0.73	0.70
GO:1901598	(-)-pinoresinol metabolic process	0.009 %	-2.0785	0.75	0.90
GO:0009688	abscisic acid biosynthetic process	0.091 %	-1.1749	0.70	0.93
GO:0009687	abscisic acid metabolic process	0.125 %	-1.0333	0.73	0.98
GO:0009698	phenylpropanoid metabolic process	0.505 %	-2.9476	0.73	0.70
GO:2000762	regulation of phenylpropanoid metabolic process	0.069 %	-2.6474	0.74	0.83
GO:0043455	regulation of secondary metabolic process	0.173 %	-3.2315	0.78	0.64
GO:0018958	phenol-containing compound metabolic process	0.190 %	-1.3335	0.84	0.78
GO:0046189	phenol-containing compound biosynthetic process	0.104 %	-1.3335	0.81	0.77
GO:0051555	flavonol biosynthetic process	0.078 %	-1.3151	0.75	0.72
GO:0051553	flavone biosynthetic process	0.082 %	-1.3151	0.78	0.98
GO:0051554	flavonol metabolic process	0.082 %	-1.3092	0.79	0.99
GO:0051552	flavone metabolic process	0.091 %	-1.3092	0.82	0.77
GO:0009806	lignan metabolic process	0.022 %	-1.9542	0.77	0.74
GO:0009808	lignin metabolic process	0.285 %	-2.6132	0.73	0.91
GO:0009807	lignan biosynthetic process	0.022 %	-1.9542	0.74	0.95
GO:0009809	lignin biosynthetic process	0.181 %	-2.9317	0.70	0.88
GO:0046148	pigment biosynthetic process	0.639 %	-1.2183	0.80	0.80
GO:1900376	regulation of secondary metabolite biosynthetic process	0.078 %	-2.4781	0.74	0.88
GO:0010192	mucilage biosynthetic process	0.121 %	-1.2149	0.88	0.18
GO:0051301	cell division	1.528 %	-2.2940	0.84	0.23
GO:0015850	organic hydroxy compound transport	0.086 %	-3.1460	0.90	0.25
GO:0033037	polysaccharide localization	0.168 %	-1.8742	0.95	0.27
GO:0019748	secondary metabolic process	1.938 %	-2.1238	0.80	0.29
GO:0007049	cell cycle	2.525 %	-2.0357	0.83	0.29
GO:0010035	response to inorganic substance	3.699 %	-2.3376	0.80	0.29
GO:1901700	response to oxygen-containing compound	6.504 %	-3.4953	0.79	0.61
GO:0010033	response to organic substance	8.580 %	-1.3763	0.78	0.70
GO:0001101	response to acid chemical	5.011 %	-2.8445	0.79	0.64
GO:0045229	external encapsulating structure organization	2.577 %	-3.0999	0.90	0.31
GO:0042440	pigment metabolic process	0.785 %	-1.8657	0.82	0.31
GO:1901140	p-coumaryl alcohol transport	0.004 %	-3.2618	0.91	0.33
GO:0042430	indole-containing compound metabolic process	0.384 %	-1.7365	0.84	0.34
GO:0009750	response to fructose	0.069 %	-1.0216	0.85	0.35
GO:0010027	thylakoid membrane organization	0.164 %	-1.0159	0.83	0.35
GO:0009668	plastid membrane organization	0.168 %	-1.0159	0.83	0.73
GO:0007568	aging	0.544 %	-1.0367	0.92	0.35
GO:0009095	aromatic amino acid family biosynthetic process, prephenate pathway	0.047 %	-1.5288	0.71	0.36
GO:0016053	organic acid biosynthetic process	2.296 %	-1.0419	0.66	0.81
GO:0009073	aromatic amino acid family biosynthetic process	0.207 %	-1.1502	0.68	0.85
GO:0009072	aromatic amino acid family metabolic process	0.302 %	-1.0245	0.72	0.64
GO:0009082	branched-chain amino acid biosynthetic process	0.104 %	-1.3727	0.71	0.88
GO:0009081	branched-chain amino acid metabolic process	0.173 %	-1.2594	0.74	0.56
GO:0009094	L-phenylalanine biosynthetic process	0.039 %	-1.6170	0.71	0.94
GO:0009097	isoleucine biosynthetic process	0.056 %	-1.4395	0.72	0.76
GO:1901607	alpha-amino acid biosynthetic process	0.833 %	-1.3422	0.66	0.75
GO:0006571	tyrosine biosynthetic process	0.022 %	-1.6423	0.73	0.77
GO:0006570	tyrosine metabolic process	0.047 %	-1.4908	0.75	0.76
GO:0006558	L-phenylalanine metabolic process	0.073 %	-1.4160	0.74	0.96
GO:0006549	isoleucine metabolic process	0.056 %	-1.4395	0.75	0.85
GO:0044273	sulfur compound catabolic process	0.173 %	-2.6822	0.87	0.37
GO:0016145	S-glycoside catabolic process	0.104 %	-2.9411	0.76	0.63
GO:0016143	S-glycoside metabolic process	0.514 %	-2.6389	0.76	0.72
GO:1901658	glycosyl compound catabolic process	0.211 %	-2.4177	0.78	0.87
GO:0042344	indole glucosinolate catabolic process	0.017 %	-2.9411	0.71	0.82
GO:0042343	indole glucosinolate metabolic process	0.125 %	-2.6389	0.69	0.77
GO:0006950	response to stress	14.156 %	-3.4387	0.82	0.38
GO:1901136	carbohydrate derivative catabolic process	0.337 %	-2.0790	0.88	0.38
GO:1901657	glycosyl compound metabolic process	1.912 %	-1.4975	0.78	0.68
GO:0071452	cellular response to singlet oxygen	0.022 %	-1.6423	0.79	0.39
GO:0000304	response to singlet oxygen	0.039 %	-1.4730	0.83	0.61
GO:0036473	cell death in response to oxidative stress	0.026 %	-1.5818	0.73	0.64
GO:0097468	programmed cell death in response to reactive oxygen species	0.026 %	-1.5818	0.72	0.74
GO:0010343	singlet oxygen-mediated programmed cell death	0.013 %	-1.7448	0.73	0.96
GO:0009409	response to cold	1.580 %	-2.1362	0.80	0.40
GO:0097305	response to alcohol	2.469 %	-1.0307	0.79	0.64
GO:0009582	detection of abiotic stimulus	0.311 %	-1.0841	0.84	0.94

GO:0009581	detection of external stimulus	0.311 %		-1.0841	0.84	0.93
GO:0009583	detection of light stimulus	0.268 %		-1.2919	0.81	0.72
GO:0050826	response to freezing	0.086 %		-1.3335	0.84	0.66
GO:0010038	response to metal ion	1.938 %		-1.3664	0.80	0.77
GO:0009651	response to salt stress	2.270 %		-1.5235	0.80	0.62
GO:0009753	response to jasmonic acid	0.919 %		-1.5943	0.81	0.63
GO:0009266	response to temperature stimulus	2.287 %		-1.4874	0.82	0.62
GO:0009737	response to abscisic acid	2.443 %		-1.0376	0.79	0.71
GO:0009725	response to hormone	6.871 %		-1.6212	0.77	0.76
GO:0070417	cellular response to cold	0.147 %		-1.0130	0.79	0.69
GO:0009314	response to radiation	2.892 %		-1.2679	0.81	0.65
GO:0046686	response to cadmium ion	1.368 %		-1.1802	0.81	0.74
GO:0006970	response to osmotic stress	2.512 %		-1.9263	0.80	0.60
GO:0009414	response to water deprivation	1.390 %		-1.6520	0.75	0.55
GO:0009415	response to water	1.416 %		-1.6250	0.77	0.74
GO:0009416	response to light stimulus	2.771 %		-1.2929	0.81	0.64
GO:0071497	cellular response to freezing	0.013 %		-1.7617	0.83	0.56
GO:0009607	response to biotic stimulus	5.158 %		-2.5723	0.84	0.41
GO:0042128	nitrate assimilation	0.181 %		-1.4315	0.76	0.41
GO:0042126	nitrate metabolic process	0.181 %		-1.4315	0.76	0.98
GO:0006082	organic acid metabolic process	5.015 %		-1.5080	0.71	0.52
GO:0006413	translational initiation	0.501 %		-1.0245	0.81	0.41
GO:0006833	water transport	0.194 %		-2.4242	0.89	0.42
GO:0055085	transmembrane transport	5.175 %		-1.0656	0.94	0.64
GO:0044765	single-organism transport	5.287 %		-1.1619	0.86	0.60
GO:1902578	single-organism localization	5.481 %		-1.1165	0.86	0.63
GO:0044711	single-organism biosynthetic process	7.549 %		-3.3628	0.75	0.42
GO:0009059	macromolecule biosynthetic process	17.035 %		-1.4058	0.82	0.66
GO:0044249	cellular biosynthetic process	22.844 %		-1.9184	0.80	0.52
GO:1901576	organic substance biosynthetic process	22.969 %		-1.9002	0.82	0.72
GO:0019438	aromatic compound biosynthetic process	14.247 %		-1.1120	0.79	0.62
GO:1901362	organic cyclic compound biosynthetic process	14.769 %		-1.0643	0.81	0.63
GO:0034645	cellular macromolecule biosynthetic process	16.737 %		-1.4414	0.79	0.65
GO:0009605	response to external stimulus	6.366 %		-2.3322	0.84	0.42
GO:0009719	response to endogenous stimulus	7.359 %		-1.2621	0.83	0.44
GO:0009628	response to abiotic stimulus	7.946 %		-2.5557	0.83	0.45
GO:0042044	fluid transport	0.194 %		-2.4242	0.89	0.45
GO:1990066	energy quenching	0.043 %		-1.6170	0.87	0.45
GO:0033554	cellular response to stress	3.772 %		-1.0744	0.77	0.45
GO:0006952	defense response	6.090 %		-2.3463	0.80	0.55
GO:1901698	response to nitrogen compound	1.174 %		-1.8927	0.82	0.47
GO:0046700	heterocycle catabolic process	0.785 %		-1.5009	0.87	0.47
GO:0044270	cellular nitrogen compound catabolic process	0.785 %		-1.4851	0.85	0.55
GO:0019439	aromatic compound catabolic process	0.898 %		-1.3390	0.85	0.56
GO:1901565	organonitrogen compound catabolic process	0.811 %		-1.3327	0.84	0.48
GO:0044712	single-organism catabolic process	2.676 %		-1.0309	0.76	0.56
GO:1901361	organic cyclic compound catabolic process	0.937 %		-1.2638	0.86	0.51
GO:0051480	regulation of cytosolic calcium ion concentration	0.017 %		-1.7129	0.84	0.49
GO:0055074	calcium ion homeostasis	0.060 %		-1.1232	0.94	0.81
GO:0006874	cellular calcium ion homeostasis	0.047 %		-1.3212	0.83	0.91
GO:0010617	circadian regulation of calcium ion oscillation	0.013 %		-1.7286	0.84	0.87
GO:0072503	cellular divalent inorganic cation homeostasis	0.099 %		-1.0675	0.82	0.91
GO:0042221	response to chemical	12.434 %		-3.2402	0.82	0.49
GO:0009733	response to auxin	1.709 %		-1.2250	0.81	0.50
GO:0014070	response to organic cyclic compound	1.606 %		-1.0399	0.81	0.55

Biological Process (188) Tag Clouds

Scatterplot & Table Interactive Graph TreeMap

GROUP 3

Tip: your resulting list of GO terms seems to be quite long. If you want to reduce it further, press the Back button in your browser and choose a different setting for the "allowed similarity" parameter.

Hide/show dispensable GO terms

Export results to text table (CSV)

Make R script for plotting

term ID	description	frequency	pin?	log_{10} p-value	uniqueness	dispensability
GO:0006412	translation	3.310 %		-27.6632	0.74	0.00
GO:0032270	positive regulation of cellular protein metabolic process	0.699 %		-1.0583	0.80	0.82
GO:0002181	cytoplasmic translation	0.207 %		-2.1977	0.80	0.69

GO:0006518	peptide metabolic process	3.509 %	-26.5330	0.80	0.93
GO:1901566	organonitrogen compound biosynthetic process	6.073 %	-17.9681	0.79	0.72
GO:0043604	amide biosynthetic process	3.591 %	-27.1027	0.80	0.93
GO:0034250	positive regulation of cellular amide metabolic process	0.095 %	-1.7761	0.81	0.69
GO:0043043	peptide biosynthetic process	3.328 %	-27.3131	0.77	0.95
GO:0045727	positive regulation of translation	0.095 %	-1.7761	0.75	0.63
GO:0008152	metabolic process	56.090 %	-5.4659	1.00	0.00
GO:0009644	response to high light intensity	0.324 %	-22.3820	0.80	0.00
GO:0010196	nonphotochemical quenching	0.043 %	-2.2540	0.83	0.52
GO:1990066	energy quenching	0.043 %	-2.2540	0.83	0.52
GO:0010218	response to far red light	0.250 %	-4.4194	0.80	0.59
GO:0010114	response to red light	0.259 %	-3.2286	0.80	0.79
GO:0009642	response to light intensity	0.587 %	-18.2580	0.79	0.64

GO:0009637	response to blue light	0.341 %	-2.6023	0.80	0.64
GO:0009639	response to red or far red light	0.872 %	-2.4324	0.78	0.83
GO:0009416	response to light stimulus	2.771 %	-13.7458	0.76	0.79
GO:0071493	cellular response to UV-B	0.030 %	-1.0014	0.80	0.50
GO:0009987	cellular process	58.826 %	-5.5653	1.00	0.00
GO:0010029	regulation of seed germination	0.298 %	-2.6328	0.84	0.00
GO:0010187	negative regulation of seed germination	0.073 %	-2.2755	0.84	0.88
GO:0048580	regulation of post-embryonic development	1.303 %	-1.0386	0.83	0.78
GO:1900140	regulation of seedling development	0.315 %	-2.4665	0.84	0.86
GO:0009845	seed germination	0.600 %	-1.3183	0.89	0.92
GO:0050896	response to stimulus	25.943 %	-7.7148	0.99	0.00
GO:0051704	multi-organism process	3.362 %	-1.5405	0.99	0.00
GO:0043335	protein unfolding	0.004 %	-2.6936	0.97	0.03
GO:0051259	protein oligomerization	0.224 %	-1.8121	0.92	0.05
GO:0080158	chloroplast ribulose bisphosphate carboxylase complex biogenesis	0.017 %	-1.3042	0.96	0.58
GO:0016032	viral process	0.315 %	-4.2730	0.93	0.05
GO:0043900	regulation of multi-organism process	0.190 %	-1.1696	0.90	0.55
GO:0044419	interspecies interaction between organisms	0.423 %	-3.1013	0.96	0.60
GO:0044764	multi-organism cellular process	0.414 %	-3.4034	0.93	0.58
GO:0044403	symbiosis, encompassing mutualism through parasitism	0.406 %	-3.2455	0.96	0.97
GO:0006457	protein folding	0.764 %	-5.1240	0.95	0.05
GO:0015809	arginine transport	0.017 %	-1.0014	0.95	0.07
GO:0015977	carbon fixation	0.104 %	-2.0419	0.88	0.08
GO:0090332	stomatal closure	0.121 %	-1.5588	0.91	0.08
GO:0019684	photosynthesis, light reaction	0.505 %	-8.2740	0.79	0.09
GO:0009780	photosynthetic NADP+ reduction	0.004 %	-1.6571	0.73	0.59
GO:0042548	regulation of photosynthesis, light reaction	0.108 %	-1.9971	0.74	0.92
GO:0009768	photosynthesis, light harvesting in photosystem I	0.095 %	-5.8558	0.81	0.79
GO:0009767	photosynthetic electron transport chain	0.224 %	-2.1230	0.74	0.84
GO:0009765	photosynthesis, light harvesting	0.160 %	-6.8488	0.80	0.77
GO:0019253	reductive pentose-phosphate cycle	0.086 %	-2.6145	0.73	0.73
GO:0043467	regulation of generation of precursor metabolites and energy	0.117 %	-1.8776	0.80	0.69
GO:0010205	photoinhibition	0.060 %	-1.2834	0.58	0.95
GO:0010207	photosystem II assembly	0.086 %	-1.8168	0.78	0.78
GO:0010110	regulation of photosynthesis, dark reaction	0.013 %	-1.4264	0.72	0.88
GO:0010109	regulation of photosynthesis	0.160 %	-2.3530	0.77	0.77
GO:0043155	negative regulation of photosynthesis, light reaction	0.060 %	-1.2834	0.74	0.97
GO:0022900	electron transport chain	0.479 %	-1.5779	0.78	0.78
GO:0042549	photosystem II stabilization	0.030 %	-1.2100	0.76	0.84
GO:0042550	photosystem I stabilization	0.013 %	-1.4635	0.77	0.80
GO:0019685	photosynthesis, dark reaction	0.086 %	-2.6145	0.74	0.73
GO:1905156	negative regulation of photosynthesis	0.073 %	-2.1037	0.76	0.72
GO:0006740	NADPH regeneration	0.004 %	-1.6571	0.81	0.65
GO:0080153	negative regulation of reductive pentose-phosphate cycle	0.013 %	-1.4264	0.71	0.88
GO:0080152	regulation of reductive pentose-phosphate cycle	0.013 %	-1.4264	0.72	1.00
GO:0010270	photosystem II oxygen evolving complex assembly	0.013 %	-1.5042	0.80	0.63
GO:0015979	photosynthesis	1.057 %	-11.2961	0.91	0.10
GO:0009058	biosynthetic process	24.432 %	-7.2179	0.91	0.11
GO:0006091	generation of precursor metabolites and energy	1.459 %	-5.8681	0.91	0.11
GO:0050821	protein stabilization	0.060 %	-1.0014	0.94	0.14
GO:0080065	4-alpha-methyl-delta7-sterol oxidation	0.009 %	-1.6571	0.88	0.17
GO:0042542	response to hydrogen peroxide	0.268 %	-20.1305	0.79	0.17
GO:0000302	response to reactive oxygen species	0.678 %	-14.9874	0.79	0.71
GO:0071241	cellular response to inorganic substance	0.246 %	-1.1936	0.79	0.53
GO:0046686	response to cadmium ion	1.368 %	-6.8228	0.80	0.77
GO:0010038	response to metal ion	1.938 %	-7.1723	0.80	0.64
GO:0009615	response to virus	0.337 %	-5.3537	0.83	0.17
GO:0043207	response to external biotic stimulus	4.981 %	-2.8060	0.78	0.94
GO:0051707	response to other organism	4.968 %	-2.8060	0.78	0.81
GO:0009617	response to bacterium	1.748 %	-5.2070	0.80	0.59
GO:0098542	defense response to other organism	4.044 %	-2.0540	0.76	0.92
GO:0080142	regulation of salicylic acid biosynthetic process	0.026 %	-2.3574	0.76	0.20
GO:0072330	monocarboxylic acid biosynthetic process	1.101 %	-1.0549	0.76	0.59
GO:1900384	regulation of flavonol biosynthetic process	0.022 %	-1.4635	0.78	0.57
GO:0010337	regulation of salicylic acid metabolic process	0.056 %	-1.8030	0.77	0.89
GO:0033517	myo-inositol hexakisphosphate metabolic process	0.035 %	-1.0548	0.82	0.53
GO:0009696	salicylic acid metabolic process	0.121 %	-1.1725	0.81	0.88
GO:0009697	salicylic acid biosynthetic process	0.052 %	-2.0607	0.78	0.87
GO:0032958	inositol phosphate biosynthetic process	0.047 %	-1.0407	0.78	0.91
GO:0010264	myo-inositol hexakisphosphate biosynthetic process	0.035 %	-1.0548	0.79	0.91
GO:0045912	negative regulation of carbohydrate metabolic process	0.035 %	-1.2786	0.84	0.20

GO:0044237	cellular metabolic process	46.461 %	-5.9068	0.89	0.23
GO:0019538	protein metabolic process	19.012 %	-18.3106	0.88	0.24
GO:0006807	nitrogen compound metabolic process	27.303 %	-1.8512	0.91	0.25
GO:0006950	response to stress	14.156 %	-9.2297	0.81	0.26
GO:1901430	positive regulation of syringal lignin biosynthetic process	0.009 %	-1.4264	0.78	0.27
GO:1900378	positive regulation of secondary metabolite biosynthetic process	0.017 %	-1.1165	0.80	0.79
GO:1901064	syringal lignin metabolic process	0.009 %	-1.4264	0.87	0.62
GO:1901066	syringal lignin biosynthetic process	0.009 %	-1.4264	0.83	1.00
GO:1901428	regulation of syringal lignin biosynthetic process	0.009 %	-1.4264	0.79	1.00
GO:0090506	axillary shoot meristem initiation	0.017 %	-1.4635	0.91	0.28
GO:2000032	regulation of secondary shoot formation	0.030 %	-1.3042	0.85	0.81
GO:0060688	regulation of morphogenesis of a branching structure	0.030 %	-1.3042	0.86	0.82
GO:1900618	regulation of shoot system morphogenesis	0.073 %	-1.0845	0.85	0.70
GO:1905428	regulation of plant organ formation	0.047 %	-1.0845	0.88	0.67
GO:0016485	protein processing	0.220 %	-1.3178	0.91	0.29
GO:0055114	oxidation-reduction process	7.462 %	-2.0144	0.83	0.29
GO:0043170	macromolecule metabolic process	38.032 %	-2.6439	0.88	0.29
GO:0051604	protein maturation	0.272 %	-1.2074	0.91	0.30
GO:0043603	cellular amide metabolic process	3.897 %	-25.8498	0.86	0.31
GO:0007067	mitotic nuclear division	0.699 %	-1.0776	0.88	0.32
GO:0071454	cellular response to anoxia	0.013 %	-1.4264	0.78	0.32
GO:0036293	response to decreased oxygen levels	0.281 %	-1.1794	0.81	0.93
GO:0036294	cellular response to decreased oxygen levels	0.129 %	-1.1956	0.76	0.93
GO:0071453	cellular response to oxygen levels	0.129 %	-1.1956	0.76	0.75
GO:0080053	response to phenylalanine	0.004 %	-1.3609	0.86	0.32
GO:0043201	response to leucine	0.004 %	-1.3609	0.86	0.66
GO:0080052	response to histidine	0.004 %	-1.3609	0.86	0.66
GO:0009955	adaxial/abaxial pattern specification	0.151 %	-1.0776	0.92	0.33
GO:1901564	organonitrogen compound metabolic process	8.541 %	-13.8798	0.87	0.33
GO:0006979	response to oxidative stress	1.908 %	-12.0499	0.82	0.34
GO:0048480	stigma development	0.004 %	-1.0845	0.92	0.34
GO:0071704	organic substance metabolic process	49.271 %	-3.0049	0.90	0.34
GO:0044238	primary metabolic process	46.772 %	-2.6150	0.90	0.35
GO:0010467	gene expression	18.304 %	-8.6359	0.88	0.36
GO:0031647	regulation of protein stability	0.073 %	-1.0014	0.94	0.36
GO:0048569	post-embryonic animal organ development	0.572 %	-1.4264	0.90	0.37
GO:0042425	choline biosynthetic process	0.004 %	-1.6571	0.84	0.39
GO:0006576	cellular biogenic amine metabolic process	0.255 %	-1.0489	0.84	0.83
GO:0006598	polyamine catabolic process	0.017 %	-1.2100	0.86	0.95
GO:0009310	amine catabolic process	0.039 %	-1.1895	0.87	0.67
GO:0042402	cellular biogenic amine catabolic process	0.039 %	-1.1895	0.86	0.67
GO:0006656	phosphatidylcholine biosynthetic process	0.060 %	-1.2786	0.77	0.75
GO:0019695	choline metabolic process	0.013 %	-1.4264	0.92	0.64
GO:0006124	ferredoxin metabolic process	1.917 %	-1.5042	0.87	0.40
GO:0010035	response to inorganic substance	3.699 %	-11.9064	0.80	0.40
GO:1901700	response to oxygen-containing compound	6.504 %	-6.3602	0.79	0.61
GO:0006972	hyperosmotic response	0.263 %	-4.9691	0.80	0.40
GO:0009651	response to salt stress	2.270 %	-1.0079	0.76	0.74
GO:0070482	response to oxygen levels	0.285 %	-1.1689	0.82	0.40
GO:0009607	response to biotic stimulus	5.158 %	-3.4400	0.83	0.41
GO:0006006	glucose metabolic process	0.199 %	-1.2074	0.83	0.41
GO:0071277	cellular response to calcium ion	0.009 %	-3.6813	0.83	0.42
GO:0071248	cellular response to metal ion	0.216 %	-1.4321	0.79	0.77
GO:0009605	response to external stimulus	6.366 %	-1.4769	0.82	0.42
GO:0016567	protein ubiquitination	3.837 %	-5.7829	0.86	0.44
GO:0070647	protein modification by small protein conjugation or removal	4.294 %	-4.7675	0.86	0.56
GO:0044267	cellular protein metabolic process	17.229 %	-19.3524	0.84	0.54
GO:0032446	protein modification by small protein conjugation	3.966 %	-5.4274	0.86	0.93
GO:0044260	cellular macromolecule metabolic process	35.028 %	-3.3461	0.84	0.44
GO:0009408	response to heat	0.859 %	-15.9846	0.78	0.44
GO:0009266	response to temperature stimulus	2.287 %	-9.7512	0.78	0.64
GO:0009314	response to radiation	2.892 %	-13.5204	0.78	0.56
GO:0006970	response to osmotic stress	2.512 %	-3.1733	0.76	0.65
GO:0009628	response to abiotic stimulus	7.946 %	-8.8015	0.82	0.45
GO:0010112	regulation of systemic acquired resistance	0.082 %	-3.0041	0.75	0.45
GO:0002682	regulation of immune system process	0.453 %	-1.1490	0.86	0.80
GO:0045088	regulation of innate immune response	0.397 %	-1.2889	0.74	0.86
GO:0045087	innate immune response	1.364 %	-1.1352	0.78	0.91
GO:0009816	defense response to bacterium, incompatible interaction	0.177 %	-2.2533	0.79	0.73
GO:0009814	defense response, incompatible interaction	0.725 %	-1.9358	0.76	0.75
GO:0032101	regulation of response to external stimulus	0.315 %	-1.3996	0.77	0.62
GO:0009627	systemic acquired resistance	0.281 %	-1.0619	0.78	0.80
GO:0050776	regulation of immune response	0.419 %	-1.2399	0.76	0.98
GO:0002831	regulation of response to biotic stimulus	0.293 %	-1.4908	0.77	0.55

GO:0006955	immune response	1.398 %	-1.0994	0.80	0.96
GO:0042742	defense response to bacterium	1.476 %	-1.3575	0.78	0.75
GO:0044271	cellular nitrogen compound biosynthetic process	17.018 %	-10.0850	0.79	0.45
GO:0009059	macromolecule biosynthetic process	17.035 %	-8.7620	0.82	0.66
GO:0044249	cellular biosynthetic process	22.844 %	-6.4315	0.81	0.72
GO:1901576	organic substance biosynthetic process	22.969 %	-8.3418	0.83	0.66
GO:0034641	cellular nitrogen compound metabolic process	25.404 %	-2.4943	0.83	0.60
GO:0034645	cellular macromolecule biosynthetic process	16.737 %	-9.2482	0.80	0.64
GO:0010335	response to non-ionic osmotic stress	0.004 %	-1.4635	0.85	0.45
GO:0006542	glutamine biosynthetic process	0.030 %	-1.1336	0.79	0.46
GO:0042274	ribosomal small subunit biogenesis	0.410 %	-1.3609	0.96	0.46
GO:0070271	protein complex biogenesis	1.334 %	-1.1827	0.96	0.55
GO:0045471	response to ethanol	0.613 %	-3.3526	0.82	0.46
GO:0042819	vitamin B6 biosynthetic process	0.039 %	-1.2544	0.77	0.47
GO:0042823	pyridoxal phosphate biosynthetic process	0.043 %	-1.2316	0.75	0.89
GO:0042822	pyridoxal phosphate metabolic process	0.043 %	-1.2316	0.78	0.67
GO:0042816	vitamin B6 metabolic process	0.039 %	-1.2544	0.80	0.73
GO:0051592	response to calcium ion	0.052 %	-2.9048	0.84	0.47
GO:0010043	response to zinc ion	0.186 %	-1.1586	0.83	0.56
GO:0009867	jasmonic acid mediated signaling pathway	0.319 %	-1.1023	0.72	0.47
GO:0071395	cellular response to jasmonic acid stimulus	0.337 %	-1.0469	0.79	0.81
GO:0006346	methylation-dependent chromatin silencing	0.056 %	-1.0140	0.75	0.47
GO:0006982	response to lipid hydroperoxide	0.004 %	-1.4635	0.86	0.49
GO:0033194	response to hydroperoxide	0.004 %	-1.4635	0.86	0.49
GO:0090351	seedling development	0.660 %	-1.2032	0.90	0.49
GO:0042221	response to chemical	12.434 %	-7.4301	0.81	0.49
GO:0006952	defense response	6.090 %	-1.2821	0.80	0.50

Biological Process (153) Tag Clouds

Scatterplot & Table Interactive Graph TreeMap

GROUP 4

Tip: your resulting list of GO terms seems to be quite long. If you want to reduce it further, press the Back button in your browser and choose a different setting for the "allowed similarity" parameter.

Hide/show dispensable GO terms   Export results to text table (CSV)   Make R script for plotting

term ID	description	frequency	pin?	$\log_{10}$ p-value	uniqueness	dispensability
GO:0002376	immune system process	1.532 %		-1.5770	0.99	0.00
GO:0006720	isoprenoid metabolic process	0.850 %		-6.8480	0.69	0.00
GO:0008299	isoprenoid biosynthetic process	0.738 %		-7.3932	0.64	0.94
GO:0033384	geranyl diphosphate biosynthetic process	0.056 %		-1.5706	0.69	0.78
GO:0033383	geranyl diphosphate metabolic process	0.056 %		-1.4751	0.70	0.78
GO:0033386	geranylgeranyl diphosphate biosynthetic process	0.047 %		-1.7448	0.69	0.77
GO:0033385	geranylgeranyl diphosphate metabolic process	0.047 %		-1.7448	0.71	0.77
GO:0006638	neutral lipid metabolic process	0.147 %		-1.0410	0.73	0.59
GO:0044255	cellular lipid metabolic process	3.224 %		-4.5589	0.68	0.82
GO:0045338	farnesyl diphosphate metabolic process	0.078 %		-1.3027	0.70	0.80
GO:0045337	farnesyl diphosphate biosynthetic process	0.056 %		-1.5706	0.69	0.78
GO:0016106	sesquiterpenoid biosynthetic process	0.138 %		-1.4345	0.67	0.84
GO:0016109	tetraterpenoid biosynthetic process	0.147 %		-1.0264	0.67	0.97
GO:0016114	terpenoid biosynthetic process	0.591 %		-6.7807	0.64	0.67
GO:0016117	carotenoid biosynthetic process	0.147 %		-1.0264	0.67	0.85
GO:1901336	lactone biosynthetic process	0.026 %		-1.5046	0.88	1.00

GO:1901601	strigolactone biosynthetic process	0.026 %	-1.5046	0.69	0.85
GO:1901600	strigolactone metabolic process	0.026 %	-1.5046	0.70	1.00
GO:0008610	lipid biosynthetic process	2.464 %	-5.8662	0.68	0.69
GO:0006714	sesquiterpenoid metabolic process	0.181 %	-1.3319	0.68	0.86
GO:0006721	terpenoid metabolic process	0.699 %	-6.2208	0.66	0.96
GO:0023052	signaling	8.908 %	-1.2179	0.99	0.00
GO:0040011	locomotion	0.164 %	-1.0936	0.99	0.00
GO:0044699	single-organism process	41.709 %	-1.3798	0.99	0.00
GO:0050896	response to stimulus	25.943 %	-1.6541	0.99	0.00
GO:0010111	glyoxysome organization	0.004 %	-1.6279	0.96	0.03
GO:0042425	choline biosynthetic process	0.004 %	-2.1016	0.82	0.06
GO:0006576	cellular biogenic amine metabolic process	0.255 %	-1.8583	0.82	0.72
GO:0006598	polyamine catabolic process	0.017 %	-1.6488	0.83	0.95
GO:0006595	polyamine metabolic process	0.095 %	-1.0717	0.83	0.89
GO:0009309	amine biosynthetic process	0.186 %	-1.0034	0.81	0.85
GO:0009310	amine catabolic process	0.039 %	-1.6279	0.84	0.76
GO:0042402	cellular biogenic amine catabolic process	0.039 %	-1.6279	0.82	0.83
GO:0042401	cellular biogenic amine biosynthetic process	0.186 %	-1.0034	0.80	0.94
GO:0006656	phosphatidylcholine biosynthetic process	0.060 %	-1.7187	0.65	0.75
GO:0046470	phosphatidylcholine metabolic process	0.099 %	-1.1289	0.66	0.86
GO:0019695	choline metabolic process	0.013 %	-1.8687	0.92	0.64
GO:0044106	cellular amine metabolic process	0.306 %	-1.6897	0.83	0.89
GO:1901334	lactone metabolic process	0.026 %	-1.5046	0.92	0.06
GO:0009862	systemic acquired resistance, salicylic acid mediated signaling pathway	0.052 %	-4.0178	0.64	0.08
GO:0071229	cellular response to acid chemical	2.158 %	-1.4086	0.70	0.75
GO:1901701	cellular response to oxygen-containing compound	2.788 %	-1.3426	0.69	0.70
GO:0097305	response to alcohol	2.469 %	-1.6595	0.74	0.69
GO:0009751	response to salicylic acid	0.876 %	-3.4629	0.74	0.66
GO:0009737	response to abscisic acid	2.443 %	-1.6714	0.73	0.67
GO:0009725	response to hormone	6.871 %	-1.0966	0.72	0.76
GO:0010204	defense response signaling pathway, resistance gene-independent	0.052 %	-1.0664	0.73	0.68
GO:0045087	innate immune response	1.364 %	-1.7840	0.79	0.83
GO:0009814	defense response, incompatible interaction	0.725 %	-1.9498	0.77	0.91
GO:0002764	immune response-regulating signaling pathway	0.022 %	-1.4345	0.75	0.53
GO:0071446	cellular response to salicylic acid stimulus	0.242 %	-2.2603	0.72	0.76
GO:0009627	systemic acquired resistance	0.281 %	-2.2080	0.79	0.73
GO:0009863	salicylic acid mediated signaling pathway	0.211 %	-2.4119	0.66	0.88
GO:0071407	cellular response to organic cyclic compound	0.798 %	-1.1835	0.71	0.85
GO:0009414	response to water deprivation	1.390 %	-1.0069	0.71	0.71
GO:0006955	immune response	1.398 %	-1.7371	0.80	0.96
GO:0071310	cellular response to organic substance	4.601 %	-1.0854	0.68	0.84
GO:0097164	ammonium ion metabolic process	0.216 %	-1.0993	0.93	0.12
GO:0009650	UV protection	0.013 %	-1.8687	0.85	0.12
GO:0034644	cellular response to UV	0.073 %	-1.2497	0.79	0.83
GO:0071482	cellular response to light stimulus	0.561 %	-1.1724	0.76	0.77
GO:0071478	cellular response to radiation	0.583 %	-1.1724	0.76	0.91
GO:0071493	cellular response to UV-B	0.030 %	-1.4345	0.80	0.64
GO:0006556	S-adenosylmethionine biosynthetic process	0.017 %	-1.4896	0.89	0.12
GO:0009086	methionine biosynthetic process	0.112 %	-1.0717	0.74	0.64
GO:0046500	S-adenosylmethionine metabolic process	0.043 %	-1.3421	0.92	0.53
GO:2000603	regulation of secondary growth	0.009 %	-1.1813	0.90	0.13
GO:2000605	positive regulation of secondary growth	0.004 %	-1.2029	0.90	0.87
GO:0044070	regulation of anion transport	0.164 %	-1.9647	0.83	0.15
GO:0006820	anion transport	1.524 %	-1.4191	0.89	0.57
GO:0034762	regulation of transmembrane transport	0.216 %	-1.8076	0.85	0.81
GO:0010359	regulation of anion channel activity	0.047 %	-1.9707	0.84	0.82
GO:0034765	regulation of ion transmembrane transport	0.207 %	-1.8076	0.83	0.92
GO:0098656	anion transmembrane transport	0.902 %	-1.7464	0.88	0.74
GO:1903959	regulation of anion transmembrane transport	0.129 %	-1.9707	0.83	0.95
GO:0022898	regulation of transmembrane transporter activity	0.060 %	-1.8325	0.85	1.00
GO:0051049	regulation of transport	0.609 %	-1.3238	0.84	0.89
GO:0032412	regulation of ion transmembrane transporter activity	0.060 %	-1.8325	0.84	0.98
GO:0032409	regulation of transporter activity	0.060 %	-1.8325	0.86	0.73
GO:0043269	regulation of ion transport	0.302 %	-1.6026	0.84	0.85
GO:0015706	nitrate transport	0.108 %	-1.3219	0.90	0.62
GO:0000018	regulation of DNA recombination	0.060 %	-1.5531	0.87	0.16
GO:0045910	negative regulation of DNA recombination	0.035 %	-1.5531	0.86	0.72
GO:0051053	negative regulation of DNA metabolic process	0.086 %	-1.2103	0.85	0.77
GO:0044710	single-organism metabolic process	18.390 %	-3.2569	0.87	0.16

GO:0009058	biosynthetic process	24.432 %	-1.2223	0.93	0.16
GO:0043446	cellular alkane metabolic process	0.017 %	-1.6941	0.85	0.21
GO:0043447	alkane biosynthetic process	0.017 %	-1.6941	0.82	0.21
GO:0045595	regulation of cell differentiation	0.250 %	-1.1485	0.82	0.21
GO:0006097	glyoxylate cycle	0.030 %	-1.4094	0.81	0.21
GO:0046487	*glyoxylate metabolic process*	*0.035 %*	*-1.3857*	*0.83*	*0.72*
GO:0012501	programmed cell death	0.600 %	-1.6777	0.85	0.23
GO:0008219	cell death	0.734 %	-1.2769	0.85	0.23
GO:0022900	electron transport chain	0.479 %	-1.2163	0.81	0.27
GO:0019915	lipid storage	0.112 %	-1.3857	0.91	0.28
GO:0006979	response to oxidative stress	1.908 %	-1.7754	0.83	0.29
GO:0009308	amine metabolic process	0.522 %	-1.2250	0.88	0.29
GO:0044763	single-organism cellular process	23.034 %	-1.2432	0.87	0.32
GO:0032879	regulation of localization	0.734 %	-1.2541	0.90	0.32
GO:0080167	response to karrikin	0.531 %	-1.1251	0.83	0.33
GO:0042221	response to chemical	12.434 %	-3.3868	0.80	0.33
GO:0006629	lipid metabolic process	4.683 %	-4.8462	0.79	0.35
GO:0010183	pollen tube guidance	0.117 %	-1.2029	0.77	0.36
GO:0050918	*positive chemotaxis*	*0.117 %*	*-1.2029*	*0.78*	*1.00*
GO:0042330	*taxis*	*0.117 %*	*-1.2029*	*0.82*	*0.95*
GO:0006935	*chemotaxis*	*0.117 %*	*-1.2029*	*0.78*	*1.00*
GO:0098581	detection of external biotic stimulus	0.030 %	-1.4611	0.83	0.36
GO:0016045	*detection of bacterium*	*0.013 %*	*-1.5888*	*0.84*	*0.95*
GO:0009595	*detection of biotic stimulus*	*0.030 %*	*-1.4611*	*0.85*	*0.67*
GO:0098543	*detection of other organism*	*0.017 %*	*-1.5888*	*0.84*	*0.97*
GO:0014070	response to organic cyclic compound	1.606 %	-2.1047	0.76	0.38
GO:0009735	*response to cytokinin*	*1.010 %*	*-1.0136*	*0.77*	*0.51*
GO:0033993	*response to lipid*	*3.267 %*	*-1.0090*	*0.75*	*0.59*
GO:0034484	raffinose catabolic process	0.004 %	-1.2754	0.82	0.41
GO:0033530	*raffinose metabolic process*	*0.013 %*	*-1.1228*	*0.83*	*0.56*
GO:0005991	*trehalose metabolic process*	*0.104 %*	*-1.0034*	*0.80*	*0.85*
GO:0005992	*trehalose biosynthetic process*	*0.095 %*	*-1.0217*	*0.77*	*0.67*
GO:0071241	cellular response to inorganic substance	0.246 %	-1.3737	0.75	0.41
GO:0042542	*response to hydrogen peroxide*	*0.268 %*	*-1.0264*	*0.78*	*0.53*
GO:0071277	cellular response to calcium ion	0.009 %	-1.3974	0.80	0.41
GO:0071249	*cellular response to nitrate*	*0.017 %*	*-1.3526*	*0.77*	*0.67*
GO:0009704	de-etiolation	0.047 %	-1.0936	0.80	0.42
GO:0009644	*response to high light intensity*	*0.324 %*	*-1.0012*	*0.83*	*0.52*
GO:0016099	monoterpenoid biosynthetic process	0.004 %	-1.6079	0.73	0.45
GO:0052651	monoacylglycerol catabolic process	0.004 %	-1.7448	0.74	0.45
GO:0046340	*diacylglycerol catabolic process*	*0.004 %*	*-1.7448*	*0.74*	*0.84*
GO:0046339	*diacylglycerol metabolic process*	*0.022 %*	*-1.6488*	*0.73*	*0.70*
GO:0006639	*acylglycerol metabolic process*	*0.147 %*	*-1.0410*	*0.70*	*0.87*
GO:0046464	*acylglycerol catabolic process*	*0.022 %*	*-1.5531*	*0.71*	*0.91*
GO:0046461	*neutral lipid catabolic process*	*0.022 %*	*-1.5531*	*0.72*	*0.77*
GO:0046462	*monoacylglycerol metabolic process*	*0.004 %*	*-1.7448*	*0.75*	*0.65*
GO:0046503	*glycerolipid catabolic process*	*0.035 %*	*-1.5531*	*0.72*	*0.64*
GO:0080053	response to phenylalanine	0.004 %	-1.8023	0.81	0.46
GO:0043201	*response to leucine*	*0.004 %*	*-1.8023*	*0.82*	*0.66*
GO:0080052	*response to histidine*	*0.004 %*	*-1.8023*	*0.81*	*0.66*
GO:0055085	transmembrane transport	5.175 %	-1.5628	0.91	0.47
GO:0009408	response to heat	0.859 %	-1.0129	0.81	0.47
GO:0051592	response to calcium ion	0.052 %	-1.1414	0.82	0.47
GO:0044711	single-organism biosynthetic process	7.549 %	-2.4709	0.76	0.49
GO:0044249	*cellular biosynthetic process*	*22.844 %*	*-1.4571*	*0.84*	*0.52*
GO:0055114	*oxidation-reduction process*	*7.462 %*	*-1.6819*	*0.78*	*0.53*
GO:1901576	*organic substance biosynthetic process*	*22.969 %*	*-1.1190*	*0.86*	*0.72*
GO:0016098	monoterpenoid metabolic process	0.004 %	-1.6079	0.75	0.49
GO:0009617	response to bacterium	1.748 %	-2.1383	0.80	0.49
GO:0010035	response to inorganic substance	3.699 %	-1.6886	0.76	0.49
GO:0001101	*response to acid chemical*	*5.011 %*	*-2.5696*	*0.75*	*0.58*
GO:0044700	*single organism signaling*	*8.899 %*	*-1.2194*	*0.87*	*0.91*
GO:1901700	*response to oxygen-containing compound*	*6.504 %*	*-2.0395*	*0.74*	*0.64*
GO:0070887	*cellular response to chemical stimulus*	*5.287 %*	*-1.7785*	*0.70*	*0.64*
GO:0007165	*signal transduction*	*8.731 %*	*-1.2290*	*0.64*	*0.67*
GO:0010033	*response to organic substance*	*8.580 %*	*-2.0284*	*0.73*	*0.70*
GO:0006012	galactose metabolic process	0.121 %	-1.1351	0.81	0.50
GO:0009311	*oligosaccharide metabolic process*	*0.466 %*	*-1.0105*	*0.80*	*0.61*

Biological Process (153) Tag Clouds

Scatterplot & Table Interactive Graph TreeMap

GROUP 5

Tip: your resulting list of GO terms seems to be quite long. If you want to reduce it further, press the Back button in your browser and choose a different setting for the "allowed similarity" parameter.

Hide/show dispensable GO terms

Export results to text table (CSV)

Make R script for plotting

term ID	description	frequency	pin?	log_{10} p-value	uniqueness	dispensability
GO:0002376	immune system process	1.532 %		-1.5770	0.99	0.00
GO:0006720	isoprenoid metabolic process	0.850 %		-6.8480	0.69	0.00
GO:0008299	isoprenoid biosynthetic process	0.738 %		-7.3932	0.64	0.94
GO:0033384	geranyl diphosphate biosynthetic process	0.056 %		-1.5706	0.69	0.78
GO:0033383	geranyl diphosphate metabolic process	0.056 %		-1.4751	0.70	0.78
GO:0033386	geranylgeranyl diphosphate biosynthetic process	0.047 %		-1.7448	0.69	0.77
GO:0033385	geranylgeranyl diphosphate metabolic process	0.047 %		-1.7448	0.71	0.77
GO:0006638	neutral lipid metabolic process	0.147 %		-1.0410	0.73	0.59
GO:0044255	cellular lipid metabolic process	3.224 %		-4.5589	0.68	0.82
GO:0045338	farnesyl diphosphate metabolic process	0.078 %		-1.3027	0.70	0.80
GO:0045337	farnesyl diphosphate biosynthetic process	0.056 %		-1.5706	0.69	0.78
GO:0016106	sesquiterpenoid biosynthetic process	0.138 %		-1.4345	0.67	0.84
GO:0016109	tetraterpenoid biosynthetic process	0.147 %		-1.0264	0.67	0.97
GO:0016114	terpenoid biosynthetic process	0.591 %		-6.7807	0.64	0.67
GO:0016117	carotenoid biosynthetic process	0.147 %		-1.0264	0.67	0.85
GO:1901336	lactone biosynthetic process	0.026 %		-1.5046	0.88	1.00

GO:1901601	strigolactone biosynthetic process	0.026 %	-1.5046	0.69	0.85
GO:1901600	strigolactone metabolic process	0.026 %	-1.5046	0.70	1.00
GO:0008610	lipid biosynthetic process	2.464 %	-5.8662	0.68	0.69
GO:0006714	sesquiterpenoid metabolic process	0.181 %	-1.3319	0.68	0.86
GO:0006721	terpenoid metabolic process	0.699 %	-6.2208	0.66	0.96
GO:0023052	signaling	8.908 %	-1.2179	0.99	0.00
GO:0040011	locomotion	0.164 %	-1.0936	0.99	0.00
GO:0044699	single-organism process	41.709 %	-1.3798	0.99	0.00
GO:0050896	response to stimulus	25.943 %	-1.6541	0.99	0.00
GO:0010111	glyoxysome organization	0.004 %	-1.6279	0.96	0.03
GO:0042425	choline biosynthetic process	0.004 %	-2.1016	0.82	0.06
GO:0006576	cellular biogenic amine metabolic process	0.255 %	-1.8583	0.82	0.72
GO:0006598	polyamine catabolic process	0.017 %	-1.6488	0.83	0.95
GO:0006595	polyamine metabolic process	0.095 %	-1.0717	0.83	0.89
GO:0009309	amine biosynthetic process	0.186 %	-1.0034	0.81	0.85
GO:0009310	amine catabolic process	0.039 %	-1.6279	0.84	0.76
GO:0042402	cellular biogenic amine catabolic process	0.039 %	-1.6279	0.82	0.83
GO:0042401	cellular biogenic amine biosynthetic process	0.186 %	-1.0034	0.80	0.94
GO:0006656	phosphatidylcholine biosynthetic process	0.060 %	-1.7187	0.65	0.75
GO:0046470	phosphatidylcholine metabolic process	0.099 %	-1.1289	0.66	0.86
GO:0019695	choline metabolic process	0.013 %	-1.8687	0.92	0.64
GO:0044106	cellular amine metabolic process	0.306 %	-1.6897	0.83	0.89
GO:1901334	lactone metabolic process	0.026 %	-1.5046	0.92	0.06
GO:0009862	systemic acquired resistance, salicylic acid mediated signaling pathway	0.052 %	-4.0178	0.64	0.08
GO:0071229	cellular response to acid chemical	2.158 %	-1.4086	0.70	0.75
GO:1901701	cellular response to oxygen-containing compound	2.788 %	-1.3426	0.69	0.70
GO:0097305	response to alcohol	2.469 %	-1.6595	0.74	0.69
GO:0009751	response to salicylic acid	0.876 %	-3.4629	0.74	0.66
GO:0009737	response to abscisic acid	2.443 %	-1.6714	0.73	0.67
GO:0009725	response to hormone	6.871 %	-1.0966	0.72	0.76
GO:0010204	defense response signaling pathway, resistance gene-independent	0.052 %	-1.0664	0.73	0.68
GO:0045087	innate immune response	1.364 %	-1.7840	0.79	0.83
GO:0009814	defense response, incompatible interaction	0.725 %	-1.9498	0.77	0.91
GO:0002764	immune response-regulating signaling pathway	0.022 %	-1.4345	0.75	0.53
GO:0071446	cellular response to salicylic acid stimulus	0.242 %	-2.2603	0.72	0.76
GO:0009627	systemic acquired resistance	0.281 %	-2.2080	0.79	0.73
GO:0009863	salicylic acid mediated signaling pathway	0.211 %	-2.4119	0.66	0.88
GO:0071407	cellular response to organic cyclic compound	0.798 %	-1.1835	0.71	0.85
GO:0009414	response to water deprivation	1.390 %	-1.0069	0.71	0.71
GO:0006955	immune response	1.398 %	-1.7371	0.80	0.96
GO:0071310	cellular response to organic substance	4.601 %	-1.0854	0.68	0.84
GO:0097164	ammonium ion metabolic process	0.216 %	-1.0993	0.93	0.12
GO:0009650	UV protection	0.013 %	-1.8687	0.85	0.12
GO:0034644	cellular response to UV	0.073 %	-1.2497	0.79	0.83
GO:0071482	cellular response to light stimulus	0.561 %	-1.1724	0.76	0.77
GO:0071478	cellular response to radiation	0.583 %	-1.1724	0.76	0.91
GO:0071493	cellular response to UV-B	0.030 %	-1.4345	0.80	0.64
GO:0006556	S-adenosylmethionine biosynthetic process	0.017 %	-1.4896	0.89	0.12
GO:0009086	methionine biosynthetic process	0.112 %	-1.0717	0.74	0.64
GO:0046500	S-adenosylmethionine metabolic process	0.043 %	-1.3421	0.92	0.53
GO:2000603	regulation of secondary growth	0.009 %	-1.1813	0.90	0.13
GO:2000605	positive regulation of secondary growth	0.004 %	-1.2029	0.90	0.87
GO:0044070	regulation of anion transport	0.164 %	-1.9647	0.83	0.15
GO:0006820	anion transport	1.524 %	-1.4191	0.89	0.57
GO:0034762	regulation of transmembrane transport	0.216 %	-1.8076	0.85	0.81
GO:0010359	regulation of anion channel activity	0.047 %	-1.9707	0.84	0.82
GO:0034765	regulation of ion transmembrane transport	0.207 %	-1.8076	0.83	0.92
GO:0098656	anion transmembrane transport	0.902 %	-1.7464	0.88	0.74
GO:1903959	regulation of anion transmembrane transport	0.129 %	-1.9707	0.83	0.95
GO:0022898	regulation of transmembrane transporter activity	0.060 %	-1.8325	0.85	1.00
GO:0051049	regulation of transport	0.609 %	-1.3238	0.84	0.89
GO:0032412	regulation of ion transmembrane transporter activity	0.060 %	-1.8325	0.84	0.98
GO:0032409	regulation of transporter activity	0.060 %	-1.8325	0.86	0.73
GO:0043269	regulation of ion transport	0.302 %	-1.6026	0.84	0.85
GO:0015706	nitrate transport	0.108 %	-1.3219	0.90	0.62
GO:0000018	regulation of DNA recombination	0.060 %	-1.5531	0.87	0.16
GO:0045910	negative regulation of DNA recombination	0.035 %	-1.5531	0.86	0.72
GO:0051053	negative regulation of DNA metabolic process	0.086 %	-1.2103	0.85	0.77
GO:0044710	single-organism metabolic process	18.390 %	-3.2569	0.87	0.16

GO:0009058	biosynthetic process	24.432 %	-1.2223	0.93	0.16
GO:0043446	cellular alkane metabolic process	0.017 %	-1.6941	0.85	0.21
GO:0043447	alkane biosynthetic process	0.017 %	-1.6941	0.82	0.21
GO:0045595	regulation of cell differentiation	0.250 %	-1.1485	0.82	0.21
GO:0006097	glyoxylate cycle	0.030 %	-1.4094	0.81	0.21
GO:0046487	*glyoxylate metabolic process*	*0.035 %*	*-1.3857*	*0.83*	*0.72*
GO:0012501	programmed cell death	0.600 %	-1.6777	0.85	0.23
GO:0008219	cell death	0.734 %	-1.2769	0.85	0.23
GO:0022900	electron transport chain	0.479 %	-1.2163	0.81	0.27
GO:0019915	lipid storage	0.112 %	-1.3857	0.91	0.28
GO:0006979	response to oxidative stress	1.908 %	-1.7754	0.83	0.29
GO:0009308	amine metabolic process	0.522 %	-1.2250	0.88	0.29
GO:0044763	single-organism cellular process	23.034 %	-1.2432	0.87	0.32
GO:0032879	regulation of localization	0.734 %	-1.2541	0.90	0.32
GO:0080167	response to karrikin	0.531 %	-1.1251	0.83	0.33
GO:0042221	response to chemical	12.434 %	-3.3868	0.80	0.33
GO:0006629	lipid metabolic process	4.683 %	-4.8462	0.79	0.35
GO:0010183	pollen tube guidance	0.117 %	-1.2029	0.77	0.36
GO:0050918	*positive chemotaxis*	*0.117 %*	*-1.2029*	*0.78*	*1.00*
GO:0042330	*taxis*	*0.117 %*	*-1.2029*	*0.82*	*0.95*
GO:0006935	*chemotaxis*	*0.117 %*	*-1.2029*	*0.78*	*1.00*
GO:0098581	detection of external biotic stimulus	0.030 %	-1.4611	0.83	0.36
GO:0016045	*detection of bacterium*	*0.013 %*	*-1.5888*	*0.84*	*0.95*
GO:0009595	*detection of biotic stimulus*	*0.030 %*	*-1.4611*	*0.85*	*0.67*
GO:0098543	*detection of other organism*	*0.017 %*	*-1.5888*	*0.84*	*0.97*
GO:0014070	response to organic cyclic compound	1.606 %	-2.1047	0.76	0.38
GO:0009735	*response to cytokinin*	*1.010 %*	*-1.0136*	*0.77*	*0.51*
GO:0033993	*response to lipid*	*3.267 %*	*-1.0090*	*0.75*	*0.59*
GO:0034484	raffinose catabolic process	0.004 %	-1.2754	0.82	0.41
GO:0033530	*raffinose metabolic process*	*0.013 %*	*-1.1228*	*0.83*	*0.56*
GO:0005991	*trehalose metabolic process*	*0.104 %*	*-1.0034*	*0.80*	*0.85*
GO:0005992	*trehalose biosynthetic process*	*0.095 %*	*-1.0217*	*0.77*	*0.67*
GO:0071241	cellular response to inorganic substance	0.246 %	-1.3737	0.75	0.41
GO:0042542	*response to hydrogen peroxide*	*0.268 %*	*-1.0264*	*0.78*	*0.53*
GO:0071277	cellular response to calcium ion	0.009 %	-1.3974	0.80	0.41
GO:0071249	*cellular response to nitrate*	*0.017 %*	*-1.3526*	*0.77*	*0.67*
GO:0009704	de-etiolation	0.047 %	-1.0936	0.80	0.42
GO:0009644	*response to high light intensity*	*0.324 %*	*-1.0012*	*0.83*	*0.52*
GO:0016099	monoterpenoid biosynthetic process	0.004 %	-1.6079	0.73	0.45
GO:0052651	monoacylglycerol catabolic process	0.004 %	-1.7448	0.74	0.45
GO:0046340	*diacylglycerol catabolic process*	*0.004 %*	*-1.7448*	*0.74*	*0.84*
GO:0046339	*diacylglycerol metabolic process*	*0.022 %*	*-1.6488*	*0.73*	*0.70*
GO:0006639	*acylglycerol metabolic process*	*0.147 %*	*-1.0410*	*0.70*	*0.87*
GO:0046464	*acylglycerol catabolic process*	*0.022 %*	*-1.5531*	*0.71*	*0.91*
GO:0046461	*neutral lipid catabolic process*	*0.022 %*	*-1.5531*	*0.72*	*0.77*
GO:0046462	*monoacylglycerol metabolic process*	*0.004 %*	*-1.7448*	*0.75*	*0.65*
GO:0046503	*glycerolipid catabolic process*	*0.035 %*	*-1.5531*	*0.72*	*0.64*
GO:0080053	response to phenylalanine	0.004 %	-1.8023	0.81	0.46
GO:0043201	*response to leucine*	*0.004 %*	*-1.8023*	*0.82*	*0.66*
GO:0080052	*response to histidine*	*0.004 %*	*-1.8023*	*0.81*	*0.66*
GO:0055085	transmembrane transport	5.175 %	-1.5628	0.91	0.47
GO:0009408	response to heat	0.859 %	-1.0129	0.81	0.47
GO:0051592	response to calcium ion	0.052 %	-1.1414	0.82	0.47
GO:0044711	single-organism biosynthetic process	7.549 %	-2.4709	0.76	0.49
GO:0044249	*cellular biosynthetic process*	*22.844 %*	*-1.4571*	*0.84*	*0.52*
GO:0055114	*oxidation-reduction process*	*7.462 %*	*-1.6819*	*0.78*	*0.53*
GO:1901576	*organic substance biosynthetic process*	*22.969 %*	*-1.1190*	*0.86*	*0.72*
GO:0016098	monoterpenoid metabolic process	0.004 %	-1.6079	0.75	0.49
GO:0009617	response to bacterium	1.748 %	-2.1383	0.80	0.49
GO:0010035	response to inorganic substance	3.699 %	-1.6886	0.76	0.49
GO:0001101	*response to acid chemical*	*5.011 %*	*-2.5696*	*0.75*	*0.58*
GO:0044700	*single organism signaling*	*8.899 %*	*-1.2194*	*0.87*	*0.91*
GO:1901700	*response to oxygen-containing compound*	*6.504 %*	*-2.0395*	*0.74*	*0.64*
GO:0070887	*cellular response to chemical stimulus*	*5.287 %*	*-1.7785*	*0.70*	*0.64*
GO:0007165	*signal transduction*	*8.731 %*	*-1.2290*	*0.64*	*0.67*
GO:0010033	*response to organic substance*	*8.580 %*	*-2.0284*	*0.73*	*0.70*
GO:0006012	galactose metabolic process	0.121 %	-1.1351	0.81	0.50
GO:0009311	*oligosaccharide metabolic process*	*0.466 %*	*-1.0105*	*0.80*	*0.61*

Biological Process (59) Tag Clouds

Scatterplot & Table Interactive Graph TreeMap

GROUP 6

Hide/show dispensable GO terms

Export results to text table (CSV)

Make R script for plotting

term ID	description	frequency	pin?	log_{10} p-value	uniqueness	dispensability
GO:0015706	nitrate transport	0.108 %		-1.4979	0.94	0.00
GO:0016114	terpenoid biosynthetic process	0.591 %		-6.5719	0.52	0.00
GO:0046246	terpene biosynthetic process	0.043 %		-3.2168	0.58	0.86
GO:0051762	sesquiterpene biosynthetic process	0.009 %		-3.9846	0.61	0.67
GO:0051761	sesquiterpene metabolic process	0.009 %		-3.9846	0.61	0.78
GO:0008299	isoprenoid biosynthetic process	0.738 %		-6.0109	0.52	0.96
GO:0008610	lipid biosynthetic process	2.464 %		-3.4983	0.60	0.69
GO:0009686	gibberellin biosynthetic process	0.134 %		-1.1845	0.55	0.84
GO:0009685	gibberellin metabolic process	0.138 %		-1.1404	0.55	0.97
GO:0006720	isoprenoid metabolic process	0.850 %		-5.5992	0.59	0.67
GO:0006721	terpenoid metabolic process	0.699 %		-6.0860	0.52	0.94
GO:0006644	phospholipid metabolic process	1.014 %		-1.4924	0.59	0.71
GO:0044255	cellular lipid metabolic process	3.224 %		-2.7209	0.58	0.82
GO:0045338	farnesyl diphosphate metabolic process	0.078 %		-3.2729	0.58	0.80
GO:0016099	monoterpenoid biosynthetic process	0.004 %		-1.7856	0.63	0.65
GO:0016098	monoterpenoid metabolic process	0.004 %		-1.7856	0.64	0.65
GO:0042214	terpene metabolic process	0.065 %		-2.8430	0.59	0.76
GO:0016102	diterpenoid biosynthetic process	0.147 %		-1.1799	0.55	0.97

GO:0016101	diterpenoid metabolic process	0.173 %	-1.0857	0.56	0.86
GO:0071456	cellular response to hypoxia	0.117 %	-4.0156	0.69	0.04
GO:0071453	cellular response to oxygen levels	0.129 %	-3.8901	0.70	0.87
GO:0036293	response to decreased oxygen levels	0.281 %	-2.8612	0.74	0.99
GO:0036294	cellular response to decreased oxygen levels	0.129 %	-3.8901	0.69	0.99
GO:0001666	response to hypoxia	0.250 %	-2.9590	0.74	0.92
GO:0006032	chitin catabolic process	0.073 %	-1.5860	0.72	0.06
GO:0046348	amino sugar catabolic process	0.073 %	-1.5860	0.75	0.91
GO:0006030	chitin metabolic process	0.073 %	-1.5739	0.73	0.99
GO:0006026	aminoglycan catabolic process	0.073 %	-1.5860	0.74	0.98
GO:1901071	glucosamine-containing compound metabolic process	0.078 %	-1.5397	0.76	0.91
GO:1901072	glucosamine-containing compound catabolic process	0.073 %	-1.5860	0.74	1.00
GO:0030007	cellular potassium ion homeostasis	0.022 %	-1.4335	0.75	0.17
GO:0055075	potassium ion homeostasis	0.069 %	-1.2966	0.84	0.86
GO:0055067	monovalent inorganic cation homeostasis	0.324 %	-1.1121	0.85	0.63
GO:0030004	cellular monovalent inorganic cation homeostasis	0.160 %	-1.2845	0.72	0.92
GO:0006885	regulation of pH	0.255 %	-1.4007	0.83	0.79
GO:0009820	alkaloid metabolic process	0.009 %	-1.4881	0.88	0.27
GO:0006623	protein targeting to vacuole	0.155 %	-1.1082	0.90	0.29
GO:0072666	establishment of protein localization to vacuole	0.155 %	-1.1082	0.90	1.00
GO:0072665	protein localization to vacuole	0.155 %	-1.1082	0.91	0.68
GO:0009611	response to wounding	0.816 %	-3.0835	0.85	0.29
GO:0006950	response to stress	14.156 %	-2.0281	0.82	0.30
GO:0009647	skotomorphogenesis	0.026 %	-1.5397	0.77	0.31
GO:0009642	response to light intensity	0.587 %	-1.0153	0.79	0.52
GO:0009646	response to absence of light	0.142 %	-1.1321	0.80	0.69
GO:0006629	lipid metabolic process	4.683 %	-2.0658	0.74	0.34
GO:0042744	hydrogen peroxide catabolic process	0.380 %	-1.1708	0.85	0.36
GO:0070482	response to oxygen levels	0.285 %	-2.8485	0.81	0.37
GO:0009821	alkaloid biosynthetic process	0.795 %	-1.5739	0.77	0.37
GO:0009938	negative regulation of gibberellic acid mediated signaling pathway	0.039 %	-1.5397	0.74	0.38
GO:0009937	regulation of gibberellic acid mediated signaling pathway	0.073 %	-1.1121	0.73	0.75
GO:0009753	response to jasmonic acid	0.919 %	-1.5128	0.80	0.45
GO:0071249	cellular response to nitrate	0.017 %	-1.5288	0.79	0.45
GO:1902170	cellular response to reactive nitrogen species	0.104 %	-1.0155	0.79	0.69
GO:0006022	aminoglycan metabolic process	0.086 %	-1.5183	0.80	0.46
GO:0070887	cellular response to chemical stimulus	5.287 %	-1.3480	0.76	0.48
GO:0006040	amino sugar metabolic process	0.138 %	-1.3852	0.82	0.48
GO:0044711	single-organism biosynthetic process	7.549 %	-1.0243	0.73	0.49
GO:0042221	response to chemical	12.434 %	-1.1535	0.83	0.49
GO:0030104	water homeostasis	0.052 %	-1.1531	0.87	0.50

Biological Process (147) Tag Clouds

Scatterplot & Table Interactive Graph TreeMap

GROUP 7

Hide/show dispensable GO terms

Export results to text table (CSV) Make R script for plotting

term ID	description	frequency	pin?	log_{10} p-value	uniqueness	dispensability
GO:0008299	isoprenoid biosynthetic process	0.738 %		-9.2379	0.50	0.00
GO:0051762	sesquiterpene biosynthetic process	0.009 %		-1.7053	0.60	1.38
GO:0051761	sesquiterpene metabolic process	0.009 %		-1.7053	0.63	0.86
GO:0008300	isoprenoid catabolic process	0.052 %		-2.8245	0.57	0.76
GO:0016042	lipid catabolic process	1.032 %		-1.1681	0.56	0.71
GO:0009240	isopentenyl diphosphate biosynthetic process	0.091 %		-1.3584	0.54	0.96
GO:0019288	isopentenyl diphosphate biosynthetic process, methylerythritol 4-phosphate pathway	0.047 %		-1.7053	0.52	0.75
GO:0033384	geranyl diphosphate biosynthetic process	0.056 %		-1.6269	0.56	0.76
GO:0033383	geranyl diphosphate metabolic process	0.056 %		-1.5313	0.59	0.76
GO:0033386	geranylgeranyl diphosphate biosynthetic process	0.047 %		-1.8013	0.56	0.75
GO:0033385	geranylgeranyl diphosphate metabolic process	0.047 %		-1.8013	0.59	0.75
GO:0044242	cellular lipid catabolic process	0.315 %		-1.5500	0.56	0.62
GO:0006644	phospholipid metabolic process	1.014 %		-3.2700	0.56	0.71
GO:0044255	cellular lipid metabolic process	3.224 %		-4.2515	0.56	0.82
GO:0045338	farnesyl diphosphate metabolic process	0.078 %		-4.8909	0.58	0.78
GO:0045337	farnesyl diphosphate biosynthetic process	0.056 %		-1.6269	0.55	0.90
GO:0045339	farnesyl diphosphate catabolic process	0.023 %		-4.5667	0.57	0.71
GO:0016099	monoterpenoid biosynthetic process	0.004 %		-3.6542	0.63	0.63

GO:0016098	monoterpenoid metabolic process	0.004 %	-3.6542	0.66	0.63
GO:0016115	terpenoid catabolic process	0.047 %	-2.8397	0.56	0.94
GO:0016114	terpenoid biosynthetic process	0.591 %	-5.8289	0.50	0.94
GO:0046246	terpene biosynthetic process	0.043 %	-2.9727	0.57	0.75
GO:0046490	isopentenyl diphosphate metabolic process	0.091 %	-1.3584	0.62	0.75
GO:0009395	phospholipid catabolic process	0.052 %	-3.3528	0.58	0.85
GO:0008654	phospholipid biosynthetic process	0.643 %	-1.9514	0.52	0.73
GO:0008610	lipid biosynthetic process	2.464 %	-5.4348	0.53	0.69
GO:0006720	isoprenoid metabolic process	0.850 %	-8.6158	0.58	0.69
GO:0006721	terpenoid metabolic process	0.699 %	-5.3497	0.54	0.96
GO:1901926	cadinene metabolic process	1.509 %	-2.5256	0.47	1.23
GO:1901928	cadinene biosynthetic process	0.614 %	-2.5256	0.47	2.02
GO:0042214	terpene metabolic process	0.065 %	-2.6007	0.60	0.77
GO:0080158	chloroplast ribulose bisphosphate carboxylase complex biogenesis	0.017 %	-1.8013	0.96	0.00
GO:0071474	cellular hyperosmotic response	0.013 %	-3.1753	0.86	0.03
GO:0042538	hyperosmotic salinity response	0.220 %	-1.2811	0.89	0.79
GO:0071472	cellular response to salt stress	0.147 %	-1.9994	0.84	0.80
GO:0071470	cellular response to osmotic stress	0.207 %	-1.7681	0.84	0.62
GO:0071475	cellular hyperosmotic salinity response	0.009 %	-3.1753	0.86	0.67
GO:0009812	flavonoid metabolic process	0.414 %	-1.1593	0.90	0.07
GO:0046434	organophosphate catabolic process	0.112 %	-2.2567	0.76	0.07
GO:0043473	pigmentation	0.022 %	-1.0150	0.91	0.08
GO:0009850	auxin metabolic process	0.332 %	-1.8057	0.84	0.08
GO:0080024	indolebutyric acid metabolic process	0.022 %	-1.2971	0.70	0.70
GO:0009851	auxin biosynthetic process	0.250 %	-1.1485	0.79	0.86
GO:0042445	hormone metabolic process	0.695 %	-1.3354	0.87	0.83
GO:0009684	indoleacetic acid biosynthetic process	0.073 %	-1.4085	0.62	0.76
GO:0019684	photosynthesis, light reaction	0.505 %	-1.9221	0.86	0.08
GO:0009765	photosynthesis, light harvesting	0.160 %	-1.8453	0.87	0.77
GO:0019253	reductive pentose-phosphate cycle	0.086 %	-1.6452	0.59	0.73
GO:0019685	photosynthesis, dark reaction	0.086 %	-1.6452	0.59	0.73
GO:0010078	maintenance of root meristem identity	0.060 %	-1.5037	0.85	0.08
GO:0010588	cotyledon vascular tissue pattern formation	0.060 %	-1.0150	0.85	0.76
GO:0010338	leaf formation	0.022 %	-1.2094	0.86	0.52
GO:0010073	meristem maintenance	0.427 %	-1.1576	0.85	0.72
GO:0009965	leaf morphogenesis	0.380 %	-1.0235	0.84	0.70
GO:0010016	shoot system morphogenesis	0.708 %	-1.2976	0.84	0.52
GO:0015979	photosynthesis	1.057 %	-3.0408	0.88	0.09
GO:0006124	ferredoxin metabolic process	1.917 %	-2.0042	0.86	0.10
GO:0033591	response to L-ascorbic acid	0.017 %	-1.7273	0.92	0.11
GO:0033273	response to vitamin	0.026 %	-1.5173	0.92	0.90
GO:0007584	response to nutrient	0.047 %	-1.0955	0.92	0.62
GO:0005975	carbohydrate metabolic process	4.670 %	-1.0907	0.87	0.12
GO:0040009	regulation of growth rate	0.013 %	-1.7506	0.94	0.12
GO:0046620	regulation of organ growth	0.082 %	-1.2971	0.83	0.86
GO:0046622	positive regulation of organ growth	0.017 %	-1.6843	0.84	0.81
GO:0035265	organ growth	0.117 %	-1.1839	0.84	0.54
GO:0048639	positive regulation of developmental growth	0.026 %	-1.6843	0.84	0.51
GO:0030308	negative regulation of cell growth	0.043 %	-1.2811	0.78	0.55
GO:0008284	positive regulation of cell proliferation	0.129 %	-1.0400	0.86	0.14
GO:0044710	single-organism metabolic process	18.390 %	-6.6668	0.80	0.15
GO:0009058	biosynthetic process	24.432 %	-4.5137	0.88	0.16
GO:0006556	S-adenosylmethionine biosynthetic process	0.017 %	-1.5458	0.85	0.17
GO:0044272	sulfur compound biosynthetic process	0.699 %	-1.2194	0.81	0.66
GO:0046500	S-adenosylmethionine metabolic process	0.043 %	-1.3980	0.89	0.53
GO:0016137	glycoside metabolic process	0.017 %	-2.7117	0.77	0.20
GO:0009853	photorespiration	0.242 %	-2.5132	0.84	0.21
GO:0043094	cellular metabolic compound salvage	0.419 %	-1.9671	0.83	0.22
GO:0044237	cellular metabolic process	46.461 %	-1.0784	0.86	0.23
GO:0015977	carbon fixation	0.104 %	-3.0285	0.75	0.23
GO:0009696	salicylic acid metabolic process	0.121 %	-5.0018	0.69	0.24
GO:0032787	monocarboxylic acid metabolic process	2.128 %	-2.4511	0.64	0.56
GO:0043436	oxoacid metabolic process	5.002 %	-1.4832	0.61	0.93
GO:0019752	carboxylic acid metabolic process	4.437 %	-1.5964	0.61	0.82
GO:0006082	organic acid metabolic process	5.015 %	-1.0811	0.62	0.71
GO:0046482	para-aminobenzoic acid metabolic process	0.017 %	-2.8095	0.73	0.82
GO:0009694	jasmonic acid metabolic process	0.117 %	-1.9111	0.70	0.56
GO:0018874	benzoate metabolic process	0.009 %	-3.1527	0.74	0.78
GO:0009611	response to wounding	0.816 %	-1.1274	0.92	0.24
GO:0046148	pigment biosynthetic process	0.639 %	-2.5926	0.70	0.28
GO:0009718	anthocyanin-containing compound biosynthetic process	0.121 %	-1.9566	0.66	0.82
GO:0009813	flavonoid biosynthetic process	0.332 %	-1.4041	0.82	0.91

GO:0016109	tetraterpenoid biosynthetic process	0.147 %	-1.0809	0.54	0.97
GO:0016117	carotenoid biosynthetic process	0.147 %	-1.0809	0.53	0.83
GO:0046283	anthocyanin-containing compound metabolic process	0.190 %	-1.5289	0.71	0.85
GO:0010051	xylem and phloem pattern formation	0.259 %	-1.1044	0.85	0.30
GO:0010218	response to far red light	0.250 %	-2.5387	0.90	0.31
GO:0010114	response to red light	0.259 %	-2.3936	0.90	0.79
GO:0009641	shade avoidance	0.069 %	-1.0357	0.91	0.71
GO:0009637	response to blue light	0.341 %	-2.0547	0.90	0.60
GO:0009639	response to red or far red light	0.872 %	-1.8202	0.90	0.67
GO:0044763	single-organism cellular process	23.034 %	-1.5294	0.81	0.32
GO:0006629	lipid metabolic process	4.683 %	-3.9407	0.66	0.35
GO:0009817	defense response to fungus, incompatible interaction	0.207 %	-1.6760	0.91	0.35
GO:0009627	systemic acquired resistance	0.281 %	-1.3749	0.91	0.81
GO:0042440	pigment metabolic process	0.785 %	-2.2474	0.72	0.35
GO:0009834	plant-type secondary cell wall biogenesis	0.263 %	-1.4425	0.89	0.37
GO:2000652	regulation of secondary cell wall biogenesis	0.095 %	-1.1542	0.88	0.78
GO:1903338	regulation of cell wall organization or biogenesis	0.138 %	-1.1264	0.90	0.53
GO:0006730	one-carbon metabolic process	0.164 %	-1.0109	0.71	0.37
GO:0070301	cellular response to hydrogen peroxide	0.035 %	-2.7662	0.87	0.37
GO:0042542	response to hydrogen peroxide	0.268 %	-1.1238	0.90	0.82
GO:0034599	cellular response to oxidative stress	0.453 %	-1.5349	0.85	0.58
GO:0034614	cellular response to reactive oxygen species	0.207 %	-1.7322	0.85	0.76
GO:0042819	vitamin B6 biosynthetic process	0.039 %	-1.7506	0.67	0.37
GO:0042816	vitamin B6 metabolic process	0.039 %	-1.7506	0.73	0.73
GO:0072525	pyridine-containing compound biosynthetic process	0.099 %	-1.2366	0.81	0.70
GO:0019637	organophosphate metabolic process	2.702 %	-1.1681	0.81	0.40
GO:0016139	glycoside catabolic process	0.009 %	-2.9909	0.73	0.43
GO:1901658	glycosyl compound catabolic process	0.211 %	-2.1178	0.67	0.73
GO:0009312	oligosaccharide biosynthetic process	0.255 %	-5.5762	0.57	0.44
GO:0044723	single-organism carbohydrate metabolic process	2.283 %	-2.3801	0.59	0.60
GO:0046351	disaccharide biosynthetic process	0.155 %	-6.0590	0.57	0.86
GO:0000023	maltose metabolic process	0.017 %	-3.1093	0.67	0.73
GO:0000024	maltose biosynthetic process	0.004 %	-3.3528	0.66	0.67
GO:0016051	carbohydrate biosynthetic process	1.442 %	-3.7322	0.53	0.77
GO:0009311	oligosaccharide metabolic process	0.466 %	-3.9962	0.62	0.65
GO:0044264	cellular polysaccharide metabolic process	1.187 %	-1.0631	0.70	0.86
GO:0044262	cellular carbohydrate metabolic process	1.817 %	-2.3581	0.73	0.75
GO:0005991	trehalose metabolic process	0.104 %	-3.9584	0.62	0.88
GO:0034637	cellular carbohydrate biosynthetic process	0.915 %	-3.4789	0.52	0.83
GO:0005992	trehalose biosynthetic process	0.095 %	-4.0157	0.58	0.89
GO:0005984	disaccharide metabolic process	0.285 %	-5.0240	0.60	0.90
GO:0005983	starch catabolic process	0.073 %	-2.6123	0.70	0.44
GO:0009251	glucan catabolic process	0.229 %	-2.0804	0.71	0.72
GO:0000272	polysaccharide catabolic process	0.777 %	-1.2118	0.68	0.82
GO:0044247	cellular polysaccharide catabolic process	0.229 %	-2.0804	0.68	0.87
GO:0044275	cellular carbohydrate catabolic process	0.298 %	-1.7640	0.68	0.74
GO:0010413	glucuronoxylan metabolic process	0.047 %	-1.1718	0.75	0.52
GO:0010417	glucuronoxylan biosynthetic process	0.047 %	-1.1718	0.58	0.89
GO:0005982	starch metabolic process	0.268 %	-1.6546	0.73	0.77
GO:0045492	xylan biosynthetic process	0.138 %	-1.0955	0.55	0.89
GO:1901136	carbohydrate derivative catabolic process	0.337 %	-1.7846	0.82	0.46
GO:0006972	hyperosmotic response	0.263 %	-1.1663	0.89	0.48
GO:0044711	single-organism biosynthetic process	7.549 %	-7.5650	0.63	0.49
GO:0044249	cellular biosynthetic process	22.844 %	-4.4432	0.75	0.52
GO:1901576	organic substance biosynthetic process	22.969 %	-3.9731	0.76	0.72
GO:0044712	single-organism catabolic process	2.676 %	-1.1673	0.63	0.50
GO:1901575	organic substance catabolic process	7.402 %	-1.2019	0.78	0.74

Biological Process (206) Tag Clouds

Scatterplot & Table Interactive Graph TreeMap

GROUP 8

Tip: your resulting list of GO terms seems to be quite long. If you want to reduce it further, press the Back button in your browser and choose a different setting for the "allowed similarity" parameter.

Hide/show dispensable GO terms   Export results to text table (CSV)   Make R script for plotting

term ID	description	frequency	pin?	log_{10} p-value	uniqueness	dispensability
GO:0009834	plant-type secondary cell wall biogenesis	0.263 %		-5.3398	0.89	0.00
GO:0042546	cell wall biogenesis	0.928 %		-3.3563	0.89	0.73
GO:0000271	polysaccharide biosynthetic process	0.868 %		-1.5221	0.68	0.83
GO:2000652	regulation of secondary cell wall biogenesis	0.095 %		-1.0080	0.84	0.78
GO:0044264	cellular polysaccharide metabolic process	1.187 %		-1.2113	0.78	0.86
GO:0044042	glucan metabolic process	0.962 %		-1.5221	0.81	0.80
GO:0071669	plant-type cell wall organization or biogenesis	1.152 %		-3.5661	0.90	0.64
GO:0009664	plant-type cell wall organization	0.652 %		-1.6024	0.88	0.87
GO:0045488	pectin metabolic process	0.647 %		-1.5924	0.81	0.71
GO:0052546	cell wall pectin metabolic process	0.095 %		-1.6730	0.78	0.83
GO:0009832	plant-type cell wall biogenesis	0.626 %		-3.6148	0.88	0.84
GO:0010400	rhamnogalacturonan I side chain metabolic process	0.009 %		-2.9189	0.80	0.76
GO:0010395	rhamnogalacturonan I metabolic process	0.013 %		-2.9189	0.79	0.59
GO:0010393	galacturonan metabolic process	0.652 %		-1.5726	0.81	0.77
GO:0034637	cellular carbohydrate biosynthetic process	0.915 %		-1.3590	0.66	0.89

GO:0046688	response to copper ion	0.069 %	-4.4610	0.94	0.00
GO:0046274	lignin catabolic process	0.078 %	-4.9173	0.76	0.03
GO:0044550	secondary metabolite biosynthetic process	1.183 %	-2.3224	0.76	0.80
GO:0009698	phenylpropanoid metabolic process	0.505 %	-2.6122	0.76	0.62
GO:0009699	phenylpropanoid biosynthetic process	0.337 %	-3.0867	0.72	0.91
GO:0010023	proanthocyanidin biosynthetic process	0.035 %	-4.5226	0.75	0.70
GO:0046189	phenol-containing compound biosynthetic process	0.104 %	-3.8940	0.85	0.84
GO:0009808	lignin metabolic process	0.285 %	-3.6584	0.76	0.81
GO:0009809	lignin biosynthetic process	0.181 %	-4.1798	0.73	0.87
GO:0046271	phenylpropanoid catabolic process	0.078 %	-4.8153	0.76	0.73
GO:0071554	cell wall organization or biogenesis	3.168 %	-2.2119	0.95	0.05
GO:0009056	catabolic process	8.118 %	-1.1077	0.95	0.05
GO:0010076	maintenance of floral meristem identity	0.026 %	-3.3491	0.81	0.07
GO:0019827	stem cell population maintenance	0.216 %	-2.6426	0.82	0.88
GO:0048510	regulation of timing of transition from vegetative to reproductive phase	0.164 %	-1.5548	0.78	0.63
GO:0048506	regulation of timing of meristematic phase transition	0.164 %	-1.5548	0.78	0.95
GO:0010582	floral meristem determinacy	0.052 %	-2.4519	0.78	0.69
GO:0010074	maintenance of meristem identity	0.155 %	-1.9048	0.78	0.97
GO:0010077	maintenance of inflorescence meristem identity	0.039 %	-3.0774	0.80	0.80
GO:0010022	meristem determinacy	0.056 %	-2.4519	0.81	0.72
GO:0046777	protein autophosphorylation	0.604 %	-2.0302	0.89	0.07
GO:0016310	phosphorylation	6.966 %	-1.7112	0.89	0.75
GO:0006468	protein phosphorylation	5.334 %	-1.6489	0.87	0.62
GO:0000060	protein import into nucleus, translocation	0.039 %	-3.6887	0.76	0.07
GO:0070727	cellular macromolecule localization	2.167 %	-1.8328	0.80	0.88
GO:1902593	single-organism nuclear import	0.242 %	-2.0085	0.74	0.99
GO:0070201	regulation of establishment of protein localization	0.026 %	-1.3039	0.78	0.87
GO:0006886	intracellular protein transport	1.675 %	-1.2309	0.78	0.94
GO:0044744	protein targeting to nucleus	0.246 %	-2.0085	0.79	0.98
GO:0032386	regulation of intracellular transport	0.039 %	-1.3282	0.76	0.91
GO:0032388	positive regulation of intracellular transport	0.013 %	-1.3408	0.76	0.88
GO:0034504	protein localization to nucleus	0.268 %	-1.9330	0.81	0.76
GO:1903827	regulation of cellular protein localization	0.065 %	-1.3282	0.74	0.85
GO:1903829	positive regulation of cellular protein localization	0.013 %	-1.3408	0.76	0.77
GO:0008104	protein localization	3.448 %	-1.6194	0.83	0.81
GO:0017038	protein import	0.531 %	-1.2703	0.77	0.51
GO:0006606	protein import into nucleus	0.242 %	-2.0085	0.72	0.94
GO:0006611	protein export from nucleus	0.022 %	-1.3159	0.82	0.66
GO:0034613	cellular protein localization	2.016 %	-2.1702	0.79	0.73
GO:0046822	regulation of nucleocytoplasmic transport	0.013 %	-1.3957	0.75	0.64
GO:0006913	nucleocytoplasmic transport	0.466 %	-2.2259	0.80	0.81
GO:0051170	nuclear import	0.246 %	-1.8842	0.81	0.94
GO:0051169	nuclear transport	0.470 %	-2.2259	0.82	0.56
GO:1901615	organic hydroxy compound metabolic process	1.265 %	-1.3218	0.94	0.08
GO:0009623	response to parasitic fungus	0.009 %	-1.7394	0.95	0.12
GO:0050832	defense response to fungus	2.236 %	-1.2260	0.92	0.55
GO:0009620	response to fungus	2.521 %	-1.2502	0.92	0.76
GO:0010219	regulation of vernalization response	0.022 %	-3.7505	0.87	0.13
GO:0010220	positive regulation of vernalization response	0.013 %	-3.7505	0.85	0.84
GO:0050826	response to freezing	0.086 %	-1.0373	0.92	0.57
GO:0010048	vernalization response	0.065 %	-2.8497	0.92	0.52
GO:0006349	regulation of gene expression by genetic imprinting	0.056 %	-1.0080	0.84	0.15
GO:0018958	phenol-containing compound metabolic process	0.190 %	-3.8940	0.88	0.17
GO:0010116	positive regulation of abscisic acid biosynthetic process	0.017 %	-1.5995	0.69	0.58
GO:0010115	regulation of abscisic acid biosynthetic process	0.030 %	-1.0824	0.69	0.91
GO:0016114	terpenoid biosynthetic process	0.591 %	-1.0470	0.74	0.71
GO:1901617	organic hydroxy compound biosynthetic process	0.824 %	-2.0161	0.88	0.78
GO:0042214	terpene metabolic process	0.065 %	-1.0024	0.81	0.76
GO:0000281	mitotic cytokinesis	0.311 %	-2.1285	0.81	0.17
GO:0000278	mitotic cell cycle	1.152 %	-1.5509	0.81	0.70
GO:0010069	zygote asymmetric cytokinesis in embryo sac	0.013 %	-1.5764	0.77	0.79
GO:0010070	zygote asymmetric cell division	0.017 %	-1.5764	0.85	0.57
GO:0000910	cytokinesis	0.384 %	-1.9320	0.82	0.73
GO:0061640	cytoskeleton-dependent cytokinesis	0.332 %	-1.9641	0.81	0.97
GO:0009833	plant-type primary cell wall biogenesis	0.091 %	-2.1950	0.77	0.89
GO:1903047	mitotic cell cycle process	1.083 %	-1.6622	0.80	0.86
GO:0009226	nucleotide-sugar biosynthetic process	0.173 %	-1.0253	0.87	0.18
GO:0030048	actin filament-based movement	0.086 %	-1.6501	0.86	0.18
GO:0009092	homoserine metabolic process	0.026 %	-2.0730	0.80	0.18
GO:0019279	L-methionine biosynthetic process from L-homoserine via cystathionine	0.004 %	-2.0730	0.79	0.93

GO:0019281	L-methionine biosynthetic process from homoserine via O-succinyl-L-homoserine and cystathionine	0.004 %	-2.0730	0.79	0.64
GO:0071265	L-methionine biosynthetic process	0.052 %	-1.3957	0.76	0.80
GO:0071266	'de novo' L-methionine biosynthetic process	0.009 %	-1.8113	0.78	0.78
GO:0009064	glutamine family amino acid metabolic process	0.367 %	-1.1335	0.76	0.71
GO:0006541	glutamine metabolic process	0.168 %	-1.8011	0.78	0.58
GO:0008037	cell recognition	0.220 %	-1.8254	0.85	0.19
GO:0009719	response to endogenous stimulus	7.359 %	-1.0509	0.93	0.20
GO:0030244	cellulose biosynthetic process	0.216 %	-3.2255	0.68	0.21
GO:0044723	single-organism carbohydrate metabolic process	2.283 %	-1.1495	0.73	0.75
GO:0046352	disaccharide catabolic process	0.035 %	-1.1915	0.74	0.91
GO:0016051	carbohydrate biosynthetic process	1.442 %	-1.0427	0.69	0.71
GO:0051274	beta-glucan biosynthetic process	0.272 %	-2.7589	0.67	0.95
GO:0051273	beta-glucan metabolic process	0.393 %	-2.4994	0.79	0.79
GO:0044262	cellular carbohydrate metabolic process	1.817 %	-1.1600	0.81	0.58
GO:0030243	cellulose metabolic process	0.337 %	-2.8777	0.79	0.93
GO:0000023	maltose metabolic process	0.017 %	-1.2490	0.77	0.65
GO:0000025	maltose catabolic process	0.009 %	-1.8979	0.77	0.51
GO:0009250	glucan biosynthetic process	0.509 %	-2.1629	0.66	0.86
GO:0006073	cellular glucan metabolic process	0.937 %	-1.5221	0.78	0.92
GO:0033692	cellular polysaccharide biosynthetic process	0.729 %	-1.6588	0.65	0.92
GO:0043255	regulation of carbohydrate biosynthetic process	0.117 %	-1.6139	0.70	0.65
GO:0009729	detection of brassinosteroid stimulus	0.009 %	-1.2701	0.94	0.22
GO:0071514	genetic imprinting	0.060 %	-1.0080	0.84	0.23
GO:0051301	cell division	1.528 %	-1.8631	0.83	0.23
GO:0010424	DNA methylation on cytosine within a CG sequence	0.030 %	-1.5544	0.89	0.23
GO:0032776	DNA methylation on cytosine	0.078 %	-1.3159	0.88	0.78
GO:0009910	negative regulation of flower development	0.164 %	-2.9839	0.73	0.27
GO:1902679	negative regulation of RNA biosynthetic process	1.057 %	-1.0654	0.74	0.99
GO:0051241	negative regulation of multicellular organismal process	0.445 %	-1.5653	0.78	0.65
GO:0051253	negative regulation of RNA metabolic process	1.062 %	-1.0600	0.77	0.96
GO:0048581	negative regulation of post-embryonic development	0.354 %	-1.8065	0.73	0.89
GO:0045892	negative regulation of transcription, DNA-templated	0.958 %	-1.0654	0.74	0.64
GO:0048438	floral whorl development	0.669 %	-1.3464	0.78	0.67
GO:2000242	negative regulation of reproductive process	0.220 %	-2.6161	0.85	0.71
GO:1903507	negative regulation of nucleic acid-templated transcription	1.057 %	-1.0654	0.74	0.98
GO:0040034	regulation of development, heterochronic	0.224 %	-1.3641	0.82	0.62
GO:0051093	negative regulation of developmental process	0.492 %	-1.4238	0.79	0.61
GO:0009909	regulation of flower development	0.609 %	-1.1588	0.72	0.82
GO:0031122	cytoplasmic microtubule organization	0.216 %	-1.6187	0.82	0.27
GO:0043622	cortical microtubule organization	0.112 %	-1.6380	0.83	0.81
GO:0030865	cortical cytoskeleton organization	0.129 %	-1.5952	0.92	0.71
GO:0000226	microtubule cytoskeleton organization	0.518 %	-1.0810	0.81	0.83
GO:0019748	secondary metabolic process	1.938 %	-2.9813	0.82	0.27
GO:0090691	formation of plant organ boundary	0.069 %	-2.1851	0.83	0.28
GO:0010199	organ boundary specification between lateral organs and the meristem	0.039 %	-2.3489	0.84	0.96
GO:0048448	stamen morphogenesis	0.047 %	-1.1915	0.80	0.74
GO:0048446	petal morphogenesis	0.026 %	-1.3159	0.81	0.83
GO:0048451	petal formation	0.009 %	-1.5335	0.81	0.58
GO:0048859	formation of anatomical boundary	0.069 %	-1.8447	0.83	0.67
GO:0009268	response to pH	0.030 %	-1.3539	0.93	0.28
GO:0098727	maintenance of cell number	0.216 %	-2.6426	0.89	0.29
GO:0006749	glutathione metabolic process	0.069 %	-1.0080	0.89	0.29
GO:0043693	monoterpene biosynthetic process	0.017 %	-1.7394	0.79	0.32
GO:0043692	monoterpene metabolic process	0.017 %	-1.7394	0.82	0.85
GO:0046246	terpene biosynthetic process	0.043 %	-1.1826	0.78	0.89
GO:0051641	cellular localization	3.008 %	-1.8893	0.87	0.32
GO:0033036	macromolecule localization	4.519 %	-1.1383	0.86	0.56
GO:0015688	iron chelate transport	0.009 %	-1.5995	0.85	0.32
GO:1990267	response to transition metal nanoparticle	2.017 %	-1.9619	0.93	0.33
GO:0019359	nicotinamide nucleotide biosynthetic process	0.043 %	-1.3282	0.75	0.34
GO:0019363	pyridine nucleotide biosynthetic process	0.056 %	-1.2594	0.75	0.91
GO:0019674	NAD metabolic process	0.078 %	-1.3039	0.77	0.71
GO:0072525	pyridine-containing compound biosynthetic process	0.099 %	-1.0893	0.84	0.74
GO:0009435	NAD biosynthetic process	0.030 %	-1.4590	0.76	0.94
GO:1901678	iron coordination entity transport	0.060 %	-1.5995	0.83	0.36
GO:0009888	tissue development	2.326 %	-1.4872	0.87	0.37
GO:0009736	cytokinin-activated signaling pathway	0.328 %	-1.9048	0.78	0.38
GO:0071368	cellular response to cytokinin stimulus	0.337 %	-1.7484	0.88	0.80
GO:0010017	red or far-red light signaling pathway	0.341 %	-1.5725	0.76	0.39
GO:0010161	red light signaling pathway	0.035 %	-1.1256	0.79	0.83
GO:0071482	cellular response to light stimulus	0.561 %	-1.0088	0.86	0.94
GO:0071478	cellular response to radiation	0.583 %	-1.0088	0.86	0.86

GO:0071489	cellular response to red or far red light	0.350 %	-1.5548	0.87	0.91
GO:0071491	cellular response to red light	0.043 %	-1.0963	0.88	0.84
GO:0060151	peroxisome localization	0.009 %	-1.4423	0.86	0.39
GO:0051646	mitochondrion localization	0.017 %	-1.3813	0.86	0.63
GO:0051645	Golgi localization	0.009 %	-1.4423	0.86	0.61
GO:0042538	hyperosmotic salinity response	0.220 %	-1.0166	0.92	0.40
GO:1904951	positive regulation of establishment of protein localization	0.013 %	-1.3408	0.77	0.40
GO:0051222	positive regulation of protein transport	0.013 %	-1.3408	0.77	1.00
GO:0051223	regulation of protein transport	0.026 %	-1.3039	0.77	0.99
GO:0032880	regulation of protein localization	0.108 %	-1.0560	0.78	0.71
GO:0090316	positive regulation of intracellular protein transport	0.013 %	-1.3408	0.74	0.95
GO:0051050	positive regulation of transport	0.043 %	-1.2701	0.80	0.67
GO:0033157	regulation of intracellular protein transport	0.022 %	-1.3282	0.74	0.95
GO:0060341	regulation of cellular localization	0.099 %	-1.3282	0.77	0.61
GO:0046824	positive regulation of nucleocytoplasmic transport	0.004 %	-1.4107	0.76	0.89
GO:0046827	positive regulation of protein export from nucleus	0.004 %	-1.4107	0.74	0.87
GO:0046825	regulation of protein export from nucleus	0.004 %	-1.4107	0.75	0.89
GO:0021700	developmental maturation	0.794 %	-1.3775	0.83	0.42
GO:0048544	recognition of pollen	0.211 %	-1.8317	0.78	0.42
GO:0044703	multi-organism reproductive process	1.610 %	-1.0206	0.93	0.64
GO:0009875	pollen-pistil interaction	0.237 %	-1.8222	0.86	0.74
GO:0090436	leaf pavement cell development	0.017 %	-1.4423	0.78	0.43
GO:0010054	trichoblast differentiation	0.427 %	-1.3144	0.70	0.97
GO:0010053	root epidermal cell differentiation	0.488 %	-1.1057	0.70	0.93
GO:0048469	cell maturation	0.410 %	-1.4322	0.75	0.87
GO:0048765	root hair cell differentiation	0.406 %	-1.4322	0.70	0.61
GO:0048764	trichoblast maturation	0.406 %	-1.4322	0.70	0.99
GO:0030154	cell differentiation	3.332 %	-1.0685	0.72	0.68
GO:0090627	plant epidermal cell differentiation	0.621 %	-1.0735	0.75	0.72
GO:0044712	single-organism catabolic process	2.676 %	-2.8051	0.80	0.43
GO:1901575	organic substance catabolic process	7.402 %	-1.1943	0.90	0.74
GO:0019439	aromatic compound catabolic process	0.898 %	-2.2844	0.87	0.55
GO:1901361	organic cyclic compound catabolic process	0.937 %	-2.1377	0.89	0.56
GO:0050792	regulation of viral process	0.013 %	-1.1034	0.89	0.43
GO:0000160	phosphorelay signal transduction system	1.049 %	-1.1676	0.77	0.43
GO:0044711	single-organism biosynthetic process	7.549 %	-1.1539	0.79	0.45
GO:0010216	maintenance of DNA methylation	0.065 %	-1.0560	0.89	0.46
GO:0006284	base-excision repair	0.155 %	-1.0195	0.82	0.51
GO:0006109	regulation of carbohydrate metabolic process	0.199 %	-1.3879	0.79	0.47
GO:0009913	epidermal cell differentiation	0.069 %	-1.9330	0.76	0.47
GO:0008544	epidermis development	0.069 %	-1.8133	0.87	0.50
GO:0060429	epithelium development	0.082 %	-1.6991	0.86	0.51
GO:0030855	epithelial cell differentiation	0.069 %	-1.9330	0.76	0.98
GO:0009735	response to cytokinin	1.010 %	-1.5532	0.92	0.48
GO:0009739	response to gibberellin	0.626 %	-1.1898	0.93	0.52
GO:0009725	response to hormone	6.871 %	-1.1117	0.91	0.66
GO:0050691	regulation of defense response to virus by host	0.017 %	-1.7075	0.87	0.50
GO:0050688	regulation of defense response to virus	0.039 %	-1.3282	0.87	0.95
GO:0002697	regulation of immune effector process	0.039 %	-1.3282	0.92	0.72

Biological Process (248) Tag Clouds

Scatterplot & Table Interactive Graph TreeMap

GROUP 9

Tip: your resulting list of GO terms seems to be quite long. If you want to reduce it further, press the Back button in your browser and choose a different setting for the "allowed similarity" parameter.

Hide/show dispensable GO terms

Export results to text table (CSV)

Make R script for plotting

term ID	description	frequency	pin?	$\log_{10}$ p-value	uniqueness	dispensability
GO:0000003	reproduction	6.655 %		-1.2588	1.00	0.00
GO:0006200	(obsolete) ATP catabolic process	0.562 %		-3.5745	0.99	0.00
GO:0009414	response to water deprivation	1.390 %		-3.3643	0.74	0.00
GO:0042542	*response to hydrogen peroxide*	*0.268 %*		*-1.4589*	*0.80*	*0.71*
GO:1901699	*cellular response to nitrogen compound*	*0.470 %*		*-1.6157*	*0.75*	*0.70*
GO:0010167	*response to nitrate*	*0.078 %*		*-2.2995*	*0.81*	*0.56*
GO:0000302	*response to reactive oxygen species*	*0.678 %*		*-1.6151*	*0.79*	*0.55*
GO:0071241	*cellular response to inorganic substance*	*0.246 %*		*-1.8287*	*0.77*	*0.62*
GO:0080026	*response to indolebutyric acid*	*0.022 %*		*-1.2295*	*0.81*	*0.56*
GO:0071214	*cellular response to abiotic stimulus*	*0.889 %*		*-2.0413*	*0.78*	*0.52*
GO:0070301	*cellular response to hydrogen peroxide*	*0.035 %*		*-1.4128*	*0.78*	*0.71*
GO:1902170	*cellular response to reactive nitrogen species*	*0.104 %*		*-1.0778*	*0.78*	*0.80*
GO:0071417	*cellular response to organonitrogen compound*	*0.147 %*		*-1.1304*	*0.76*	*0.74*
GO:0009415	*response to water*	*1.416 %*		*-3.3202*	*0.75*	*0.74*
GO:0009835	fruit ripening	0.043 %		-3.3297	0.81	0.00
GO:2000692	*negative regulation of seed maturation*	*0.017 %*		*-1.7314*	*0.76*	*0.82*

GO:0010162	seed dormancy process	0.142 %	-1.1375	0.80	0.91
GO:0010150	leaf senescence	0.462 %	-1.3102	0.80	0.82
GO:0097438	exit from dormancy	0.009 %	-1.7465	0.87	0.70
GO:0071695	anatomical structure maturation	0.043 %	-3.3297	0.85	0.62
GO:1900056	negative regulation of leaf senescence	0.043 %	-1.4809	0.76	0.67
GO:1900055	regulation of leaf senescence	0.104 %	-1.3923	0.77	0.87
GO:2000024	regulation of leaf development	0.224 %	-1.2431	0.76	0.68
GO:2000034	regulation of seed maturation	0.060 %	-1.3666	0.77	0.83
GO:2000033	regulation of seed dormancy process	0.039 %	-1.6505	0.77	0.90
GO:1902039	negative regulation of seed dormancy process	0.009 %	-1.9137	0.77	0.56
GO:0048838	release of seed from dormancy	0.009 %	-1.7465	0.83	0.70
GO:0090693	plant organ senescence	0.462 %	-1.3088	0.80	0.96
GO:0023052	signaling	8.908 %	-1.6266	0.99	0.00
GO:0032501	multicellular organismal process	11.360 %	-1.1035	0.99	0.00
GO:0042430	indole-containing compound metabolic process	0.384 %	-3.0269	0.90	0.00
GO:0050896	response to stimulus	25.943 %	-1.5068	0.99	0.00
GO:0051179	localization	12.352 %	-1.3139	0.99	0.00
GO:0071840	cellular component organization or biogenesis	14.454 %	-1.3538	0.99	0.00
GO:0006914	autophagy	0.268 %	-1.1596	0.96	0.04
GO:0010166	wax metabolic process	0.082 %	-1.0048	0.97	0.04
GO:0042545	cell wall modification	0.561 %	-2.3903	0.92	0.04
GO:0052386	cell wall thickening	0.134 %	-1.9557	0.92	0.61
GO:0071555	cell wall organization	2.430 %	-1.7653	0.91	0.80
GO:0052482	defense response by cell wall thickening	0.069 %	-2.1405	0.79	0.74
GO:0071554	cell wall organization or biogenesis	3.168 %	-1.1166	0.95	0.05
GO:1900673	olefin metabolic process	0.125 %	-1.2295	0.97	0.06
GO:0010541	acropetal auxin transport	0.013 %	-3.0587	0.81	0.06
GO:0052314	phytoalexin metabolic process	0.052 %	-1.2295	0.82	0.79
GO:0052315	phytoalexin biosynthetic process	0.052 %	-1.2295	0.79	1.00
GO:0052317	camalexin metabolic process	0.047 %	-1.2573	0.77	0.99
GO:0010120	camalexin biosynthetic process	0.047 %	-1.2573	0.76	0.71
GO:0080024	indolebutyric acid metabolic process	0.022 %	-1.4809	0.77	0.51
GO:0009700	indole phytoalexin biosynthetic process	0.052 %	-1.2295	0.77	0.99
GO:0046217	indole phytoalexin metabolic process	0.052 %	-1.2295	0.79	1.00
GO:0009403	toxin biosynthetic process	0.052 %	-1.2295	0.80	0.79
GO:0042436	indole-containing compound catabolic process	0.039 %	-1.3423	0.88	0.68
GO:0043473	pigmentation	0.022 %	-1.1951	0.93	0.06
GO:0009773	photosynthetic electron transport in photosystem I	0.065 %	-1.5063	0.87	0.07
GO:0007154	cell communication	9.698 %	-2.0741	0.95	0.08
GO:0005975	carbohydrate metabolic process	4.670 %	-1.6885	0.96	0.08
GO:0006790	sulfur compound metabolic process	1.420 %	-1.7178	0.94	0.08
GO:0044272	sulfur compound biosynthetic process	0.699 %	-1.5597	0.87	0.09
GO:0019758	glycosinolate biosynthetic process	0.194 %	-1.0341	0.75	1.00
GO:0019761	glucosinolate biosynthetic process	0.194 %	-1.0341	0.75	0.81
GO:0044273	sulfur compound catabolic process	0.173 %	-1.2206	0.90	0.74
GO:0016145	S-glycoside catabolic process	0.104 %	-1.3482	0.82	0.71
GO:0016143	S-glycoside metabolic process	0.514 %	-1.1992	0.81	0.82
GO:1901658	glycosyl compound catabolic process	0.211 %	-1.0903	0.84	0.87
GO:0042344	indole glucosinolate catabolic process	0.017 %	-1.3482	0.78	0.82
GO:0042343	indole glucosinolate metabolic process	0.125 %	-1.1992	0.76	0.77
GO:0042435	indole-containing compound biosynthetic process	0.237 %	-1.0510	0.85	0.87
GO:0010143	cutin biosynthetic process	0.091 %	-1.1910	0.95	0.18
GO:0009693	ethylene biosynthetic process	0.125 %	-1.2340	0.90	0.20
GO:1900674	olefin biosynthetic process	0.125 %	-1.2295	0.92	1.00
GO:0009692	ethylene metabolic process	0.125 %	-1.2340	0.92	1.00
GO:0043450	alkene biosynthetic process	0.125 %	-1.2340	0.90	1.00
GO:0043449	cellular alkene metabolic process	0.125 %	-1.2340	0.92	1.00
GO:0000160	phosphorelay signal transduction system	1.049 %	-1.7897	0.70	0.23
GO:0019748	secondary metabolic process	1.938 %	-1.4543	0.85	0.24
GO:0048583	regulation of response to stimulus	2.521 %	-1.3967	0.80	0.26
GO:0000045	autophagosome assembly	0.065 %	-2.1499	0.93	0.28
GO:1905037	autophagosome organization	0.065 %	-2.1499	0.92	0.98
GO:0016236	macroautophagy	0.073 %	-2.1499	0.95	0.81
GO:0007568	aging	0.544 %	-2.9102	0.85	0.29
GO:0006820	anion transport	1.524 %	-2.0681	0.84	0.29
GO:0055085	transmembrane transport	5.175 %	-1.1455	0.85	0.74
GO:0051234	establishment of localization	11.817 %	-1.0968	0.84	0.86
GO:0006810	transport	11.649 %	-1.1061	0.84	0.71
GO:0006811	ion transport	4.174 %	-1.1107	0.85	0.52
GO:0006857	oligopeptide transport	0.155 %	-1.0313	0.87	0.66
GO:0042886	amide transport	0.220 %	-1.0259	0.88	0.64
GO:0015833	peptide transport	0.177 %	-1.0313	0.87	0.95

GO:0015718	monocarboxylic acid transport	0.142 %	-1.0935	0.79	0.62
GO:0015706	nitrate transport	0.108 %	-1.5622	0.86	0.55
GO:1901140	p-coumaryl alcohol transport	0.004 %	-1.5063	0.85	0.29
GO:0080168	abscisic acid transport	0.017 %	-1.2478	0.80	0.75
GO:0022611	dormancy process	0.142 %	-1.1375	0.91	0.31
GO:0010084	specification of animal organ axis polarity	0.030 %	-2.1499	0.84	0.31
GO:0065001	specification of axis polarity	0.112 %	-1.2250	0.85	0.84
GO:0009887	animal organ morphogenesis	0.004 %	-1.0871	0.86	1.11
GO:0042218	1-aminocyclopropane-1-carboxylate biosynthetic process	0.035 %	-2.0776	0.82	0.33
GO:0008652	cellular amino acid biosynthetic process	0.915 %	-1.4965	0.77	0.62
GO:0018871	1-aminocyclopropane-1-carboxylate metabolic process	0.035 %	-1.8129	0.83	0.53
GO:0006520	cellular amino acid metabolic process	1.869 %	-1.3310	0.79	0.69
GO:0006561	proline biosynthetic process	0.043 %	-1.3138	0.81	0.74
GO:0006560	proline metabolic process	0.065 %	-1.2820	0.83	0.65
GO:0009312	oligosaccharide biosynthetic process	0.255 %	-2.0598	0.82	0.34
GO:0045490	pectin catabolic process	0.423 %	-1.0074	0.93	0.51
GO:0006012	galactose metabolic process	0.121 %	-1.3729	0.84	0.58
GO:0009311	oligosaccharide metabolic process	0.466 %	-1.4417	0.84	0.65
GO:0010325	raffinose family oligosaccharide biosynthetic process	0.004 %	-1.6758	0.86	0.67
GO:0005991	trehalose metabolic process	0.104 %	-1.2385	0.84	0.85
GO:0005992	trehalose biosynthetic process	0.095 %	-1.2573	0.82	0.83
GO:0052545	callose localization	0.155 %	-1.9391	0.87	0.34
GO:0052542	defense response by callose deposition	0.091 %	-2.0260	0.77	0.95
GO:0052543	callose deposition in cell wall	0.117 %	-2.0522	0.80	0.96
GO:0052544	defense response by callose deposition in cell wall	0.069 %	-2.1405	0.69	0.93
GO:0051716	cellular response to stimulus	12.637 %	-3.4693	0.80	0.35
GO:0010451	floral meristem growth	0.013 %	-2.0776	0.84	0.35
GO:0010432	bract development	0.017 %	-2.0456	0.85	0.36
GO:0021700	developmental maturation	0.794 %	-1.6675	0.84	0.37
GO:0042493	response to drug	0.367 %	-1.5151	0.83	0.37
GO:0019432	triglyceride biosynthetic process	0.104 %	-1.5524	0.81	0.37
GO:0045017	glycerolipid biosynthetic process	0.445 %	-1.0840	0.80	0.79
GO:0046463	acylglycerol biosynthetic process	0.117 %	-1.5151	0.81	0.99
GO:0046460	neutral lipid biosynthetic process	0.117 %	-1.5151	0.81	0.96
GO:0006639	acylglycerol metabolic process	0.147 %	-1.2770	0.83	0.99
GO:0006641	triglyceride metabolic process	0.129 %	-1.3729	0.83	0.97
GO:0010117	photoprotection	0.026 %	-1.7465	0.86	0.37
GO:0009642	response to light intensity	0.587 %	-1.1272	0.83	0.68
GO:0009644	response to high light intensity	0.324 %	-1.4316	0.83	0.64
GO:0010018	far-red light signaling pathway	0.065 %	-1.6041	0.71	0.82
GO:0071462	cellular response to water stimulus	0.129 %	-1.0259	0.73	0.79
GO:0071472	cellular response to salt stress	0.147 %	-1.0313	0.78	0.80
GO:0071482	cellular response to light stimulus	0.561 %	-1.6157	0.76	0.52
GO:0071484	cellular response to light intensity	0.043 %	-1.5723	0.80	0.79
GO:0071478	cellular response to radiation	0.583 %	-1.6157	0.76	0.91
GO:0071490	cellular response to far red light	0.073 %	-1.5622	0.79	0.83
GO:0009718	anthocyanin-containing compound biosynthetic process	0.121 %	-1.0100	0.85	0.38
GO:0006855	drug transmembrane transport	0.358 %	-1.5622	0.66	0.39
GO:0015893	drug transport	0.367 %	-1.5622	0.67	0.99
GO:0010817	regulation of hormone levels	1.109 %	-1.8498	0.91	0.39
GO:0045229	external encapsulating structure organization	2.577 %	-1.5212	0.92	0.40
GO:0016043	cellular component organization	13.263 %	-1.4714	0.91	0.58
GO:0043481	anthocyanin accumulation in tissues in response to UV light	0.017 %	-1.2162	0.75	0.41
GO:0043478	pigment accumulation in response to UV light	0.017 %	-1.2162	0.80	0.97
GO:0043480	pigment accumulation in tissues	0.017 %	-1.2162	0.78	0.97
GO:0043479	pigment accumulation in tissues in response to UV light	0.017 %	-1.2162	0.75	1.00
GO:0043476	pigment accumulation	0.022 %	-1.2162	0.83	0.99
GO:1901698	response to nitrogen compound	1.174 %	-1.2862	0.81	0.42
GO:0051262	protein tetramerization	0.056 %	-1.4493	0.94	0.42
GO:0009658	chloroplast organization	0.803 %	-1.3675	0.92	0.42
GO:0009657	plastid organization	1.032 %	-1.0658	0.92	0.58
GO:0070925	organelle assembly	1.385 %	-1.2525	0.92	0.56
GO:0009719	response to endogenous stimulus	7.359 %	-2.6075	0.84	0.43
GO:0015692	lead ion transport	0.004 %	-1.2872	0.89	0.43
GO:0009628	response to abiotic stimulus	7.946 %	-1.4634	0.83	0.43
GO:0018874	benzoate metabolic process	0.009 %	-1.6041	0.85	0.43
GO:0046482	para-aminobenzoic acid metabolic process	0.017 %	-1.4343	0.84	0.70
GO:0009696	salicylic acid metabolic process	0.121 %	-1.0840	0.83	0.82
GO:0015850	organic hydroxy compound transport	0.086 %	-1.4493	0.82	0.44
GO:0031668	cellular response to extracellular stimulus	0.691 %	-1.0108	0.79	0.44
GO:0042631	cellular response to water deprivation	0.129 %	-1.0259	0.72	0.84
GO:0042538	hyperosmotic salinity response	0.220 %	-1.6244	0.84	0.45
GO:0071475	cellular hyperosmotic salinity response	0.009 %	-1.6152	0.81	0.77

GO:0071474	cellular hyperosmotic response	0.013 %	-1.6152	0.81	0.79
GO:0006972	hyperosmotic response	0.263 %	-1.5038	0.84	0.60
GO:0071705	nitrogen compound transport	1.515 %	-1.3645	0.86	0.45
GO:0048367	shoot system development	3.599 %	-1.6440	0.78	0.45
GO:0044707	single-multicellular organism process	10.699 %	-1.4020	0.80	0.79
GO:0044702	single organism reproductive process	5.939 %	-1.6483	0.85	0.86
GO:0099402	plant organ development	3.763 %	-1.3336	0.78	0.74
GO:0003006	developmental process involved in reproduction	5.611 %	-1.1035	0.85	0.89
GO:0010154	fruit development	2.443 %	-1.1833	0.77	0.86
GO:0048608	reproductive structure development	4.674 %	-1.2404	0.76	0.77
GO:0007275	multicellular organism development	10.423 %	-1.1649	0.78	0.86
GO:0048827	phyllome development	1.921 %	-1.2816	0.79	0.67
GO:0048366	leaf development	1.368 %	-1.1074	0.79	0.78
GO:0022414	reproductive process	6.629 %	-1.2687	0.93	0.91
GO:0061458	reproductive system development	4.674 %	-1.2404	0.78	0.80
GO:0048731	system development	7.225 %	-2.0846	0.78	0.68
GO:0051241	negative regulation of multicellular organismal process	0.445 %	-1.4537	0.76	0.46
GO:0080050	regulation of seed development	0.129 %	-1.1634	0.77	0.64
GO:0048585	negative regulation of response to stimulus	0.699 %	-1.1681	0.77	0.62
GO:0010030	positive regulation of seed germination	0.069 %	-1.2976	0.80	0.61
GO:0048831	regulation of shoot system development	0.850 %	-1.0197	0.74	0.76
GO:0051093	negative regulation of developmental process	0.492 %	-1.3524	0.80	0.59
GO:0044550	secondary metabolite biosynthetic process	1.183 %	-1.8751	0.78	0.46
GO:1901141	regulation of lignin biosynthetic process	0.030 %	-1.3857	0.77	0.62
GO:2000762	regulation of phenylpropanoid metabolic process	0.069 %	-1.2034	0.78	0.83
GO:1900376	regulation of secondary metabolite biosynthetic process	0.078 %	-1.1200	0.78	0.88
GO:0009404	toxin metabolic process	0.250 %	-1.1065	0.81	0.74
GO:0033037	polysaccharide localization	0.168 %	-1.9391	0.88	0.46
GO:0070727	cellular macromolecule localization	2.167 %	-1.2096	0.85	0.58
GO:0072666	establishment of protein localization to vacuole	0.155 %	-1.1711	0.87	1.00
GO:0072665	protein localization to vacuole	0.155 %	-1.1711	0.87	0.68
GO:0006623	protein targeting to vacuole	0.155 %	-1.1711	0.86	0.66
GO:0010646	regulation of cell communication	1.139 %	-1.7653	0.86	0.47
GO:0010360	negative regulation of anion channel activity	0.017 %	-1.6267	0.78	0.47
GO:0032412	regulation of ion transmembrane transporter activity	0.060 %	-1.0022	0.79	0.98
GO:0032409	regulation of transporter activity	0.060 %	-1.0022	0.81	0.65
GO:0032410	negative regulation of transporter activity	0.017 %	-1.6267	0.79	0.95
GO:0032413	negative regulation of ion transmembrane transporter activity	0.017 %	-1.6267	0.78	1.00
GO:0044070	regulation of anion transport	0.164 %	-1.0686	0.78	0.89
GO:1903792	negative regulation of anion transport	0.017 %	-1.6267	0.78	0.95
GO:0034763	negative regulation of transmembrane transport	0.017 %	-1.6267	0.79	0.95
GO:0010359	regulation of anion channel activity	0.047 %	-1.0717	0.79	0.91
GO:0034766	negative regulation of ion transmembrane transport	0.017 %	-1.6267	0.78	1.00
GO:1903960	negative regulation of anion transmembrane transport	0.017 %	-1.6267	0.78	1.00
GO:1903959	regulation of anion transmembrane transport	0.129 %	-1.0717	0.78	0.84
GO:0022898	regulation of transmembrane transporter activity	0.060 %	-1.0022	0.80	1.00
GO:0051051	negative regulation of transport	0.026 %	-1.3857	0.81	0.60
GO:0043271	negative regulation of ion transport	0.026 %	-1.3857	0.78	0.98
GO:0042221	response to chemical	12.434 %	-1.5836	0.82	0.48
GO:0032870	cellular response to hormone stimulus	3.871 %	-3.0750	0.69	0.49
GO:0071229	cellular response to acid chemical	2.158 %	-1.0478	0.72	0.79
GO:0044700	single organism signaling	8.899 %	-1.6293	0.85	0.91
GO:1901701	cellular response to oxygen-containing compound	2.788 %	-1.2267	0.71	0.82
GO:1901700	response to oxygen-containing compound	6.504 %	-1.1922	0.77	0.70
GO:0097305	response to alcohol	2.469 %	-1.1286	0.77	0.65
GO:0097306	cellular response to alcohol	1.170 %	-1.0447	0.72	0.88
GO:0060918	auxin transport	0.406 %	-1.4946	0.76	0.97
GO:0071215	cellular response to abscisic acid stimulus	1.170 %	-1.0447	0.71	0.88
GO:0071365	cellular response to auxin stimulus	0.932 %	-2.2091	0.73	0.78
GO:0071369	cellular response to ethylene stimulus	0.906 %	-1.5966	0.73	0.91
GO:0070887	cellular response to chemical stimulus	5.287 %	-3.5244	0.73	0.59
GO:0010540	basipetal auxin transport	0.078 %	-2.1032	0.79	0.77
GO:0071310	cellular response to organic substance	4.601 %	-2.5826	0.70	0.88
GO:0007165	signal transduction	8.731 %	-1.6473	0.63	0.67
GO:0010033	response to organic substance	8.580 %	-2.1361	0.76	0.68
GO:0010035	response to inorganic substance	3.699 %	-2.5820	0.78	0.56
GO:0001101	response to acid chemical	5.011 %	-1.4854	0.78	0.62
GO:0009755	hormone-mediated signaling pathway	3.617 %	-1.9920	0.59	0.94
GO:0009737	response to abscisic acid	2.443 %	-1.1357	0.75	0.72

GO:0009734	auxin-activated signaling pathway	0.868 %	-1.4069	0.64	0.85
GO:0009733	response to auxin	1.709 %	-2.0415	0.78	0.68
GO:0009735	response to cytokinin	1.010 %	-1.4451	0.79	0.64
GO:0009725	response to hormone	6.871 %	-2.9143	0.74	0.82
GO:0010928	regulation of auxin mediated signaling pathway	0.173 %	-2.8847	0.67	0.64
GO:0010315	auxin efflux	0.086 %	-3.1119	0.60	0.70
GO:0009873	ethylene-activated signaling pathway	0.811 %	-1.8345	0.64	0.77
GO:0009966	regulation of signal transduction	1.109 %	-1.7857	0.68	0.82
GO:0009926	auxin polar transport	0.337 %	-1.5637	0.77	0.86
GO:0009937	regulation of gibberellic acid mediated signaling pathway	0.073 %	-1.1750	0.68	0.73
GO:0009939	positive regulation of gibberellic acid mediated signaling pathway	0.026 %	-1.7027	0.70	0.54
GO:0009914	hormone transport	0.436 %	-1.4801	0.77	0.71
GO:0071495	cellular response to endogenous stimulus	3.988 %	-3.0102	0.80	0.75
GO:0023051	regulation of signaling	1.126 %	-1.7720	0.89	0.49
GO:0071249	cellular response to nitrate	0.017 %	-1.5932	0.79	0.49
GO:0071366	cellular response to indolebutyric acid stimulus	0.009 %	-1.4493	0.79	0.59
GO:0006638	neutral lipid metabolic process	0.147 %	-1.2770	0.84	0.50
GO:0006950	response to stress	14.156 %	-1.4659	0.82	0.50

Biological Process (98) Tag Clouds

Scatterplot & Table

GROUP 10

Hide/show dispensable GO terms

Export results to text table (CSV)

Make R script for plotting

term ID	description	frequency	pin?	$\log_{10}$ p-value	uniqueness	dispensability
GO:0006829	zinc II ion transport	0.129 %		-2.2030	0.93	0.00
GO:0006814	*sodium ion transport*	*0.121 %*		*-1.3866*	*0.93*	*0.61*
GO:0006812	*cation transport*	*2.542 %*		*-1.1033*	*0.92*	*0.78*
GO:0030001	*metal ion transport*	*1.588 %*		*-1.4692*	*0.92*	*0.66*
GO:0009739	response to gibberellin	0.626 %		-1.5123	0.87	0.00
GO:0009741	*response to brassinosteroid*	*0.410 %*		*-1.0234*	*0.87*	*0.63*
GO:0009734	*auxin-activated signaling pathway*	*0.868 %*		*-1.0368*	*0.67*	*0.53*
GO:0009735	*response to cytokinin*	*1.010 %*		*-1.0726*	*0.88*	*0.52*
GO:0046885	regulation of hormone biosynthetic process	0.065 %		-3.1779	0.65	0.00
GO:0010422	*regulation of brassinosteroid biosynthetic process*	*0.013 %*		*-1.8070*	*0.57*	*0.89*
GO:0090354	*regulation of auxin metabolic process*	*0.056 %*		*-1.4686*	*0.67*	*0.97*
GO:0090030	*regulation of steroid hormone biosynthetic process*	*0.013 %*		*-1.8070*	*0.57*	*1.00*
GO:0032350	*regulation of hormone metabolic process*	*0.073 %*		*-2.9285*	*0.70*	*0.68*
GO:0050810	*regulation of steroid biosynthetic process*	*0.013 %*		*-1.8070*	*0.66*	*1.00*
GO:0010600	*regulation of auxin biosynthetic process*	*0.052 %*		*-1.6623*	*0.65*	*0.97*
GO:0016129	*phytosteroid biosynthetic process*	*0.173 %*		*-1.4686*	*0.69*	*0.97*
GO:0016131	*brassinosteroid metabolic process*	*0.177 %*		*-1.1443*	*0.58*	*0.98*
GO:0016132	*brassinosteroid biosynthetic process*	*0.155 %*		*-1.4686*	*0.56*	*0.84*
GO:0016128	*phytosteroid metabolic process*	*0.194 %*		*-1.1443*	*0.73*	*0.85*

GO:0009851	auxin biosynthetic process	0.250 %	-1.1270	0.67	0.88
GO:0042445	hormone metabolic process	0.695 %	-1.2955	0.69	0.87
GO:0042446	hormone biosynthetic process	0.514 %	-1.9846	0.67	0.79
GO:0009834	plant-type secondary cell wall biogenesis	0.263 %	-1.4020	0.91	0.03
GO:2000652	regulation of secondary cell wall biogenesis	0.095 %	-1.1327	0.79	0.78
GO:1903338	regulation of cell wall organization or biogenesis	0.138 %	-1.1050	0.79	0.53
GO:0071669	plant-type cell wall organization or biogenesis	1.152 %	-1.1771	0.91	0.64
GO:0071554	cell wall organization or biogenesis	3.168 %	-1.1106	0.94	0.05
GO:1901868	ecgonine methyl ester catabolic process	0.076 %	-2.9800	0.66	0.06
GO:0009821	alkaloid biosynthetic process	0.801 %	-1.4315	0.58	2.10
GO:0009822	alkaloid catabolic process	0.080 %	-2.9800	0.67	1.31
GO:0046448	tropane alkaloid metabolic process	1.686 %	-2.2819	0.58	1.67
GO:1901867	ecgonine methyl ester metabolic process	0.033 %	-2.9800	0.68	1.23
GO:1901869	ecgonine methyl ester biosynthetic process	0.009 %	-2.9800	0.68	1.13
GO:1901870	ecgonone methyl ester metabolic process	0.057 %	-2.9800	0.59	1.28
GO:1901871	ecgonone methyl ester catabolic process	0.078 %	-2.9800	0.58	1.30
GO:1901872	ecgonone methyl ester biosynthetic process	0.073 %	-2.9800	0.55	1.30
GO:0009710	tropane alkaloid biosynthetic process	0.503 %	-2.2819	0.59	2.00
GO:0051100	negative regulation of binding	0.004 %	-1.9820	0.85	0.10
GO:0043392	negative regulation of DNA binding	0.004 %	-1.9820	0.85	0.84
GO:0051101	regulation of DNA binding	0.017 %	-1.8368	0.84	0.84
GO:2000123	positive regulation of stomatal complex development	0.013 %	-2.0275	0.70	0.13
GO:2000038	regulation of stomatal complex development	0.039 %	-1.1686	0.69	0.73
GO:0015074	DNA integration	0.017 %	-1.3119	0.89	0.14
GO:0009268	response to pH	0.030 %	-1.4818	0.92	0.15
GO:0019218	regulation of steroid metabolic process	0.013 %	-1.8070	0.69	0.20
GO:0010115	regulation of abscisic acid biosynthetic process	0.030 %	-1.2080	0.61	0.70
GO:0009688	abscisic acid biosynthetic process	0.091 %	-1.0061	0.70	0.93
GO:0046890	regulation of lipid biosynthetic process	0.194 %	-1.0596	0.64	0.83
GO:0010047	fruit dehiscence	0.039 %	-1.1159	0.85	0.23
GO:0009901	anther dehiscence	0.069 %	-1.0791	0.78	0.92
GO:0009820	alkaloid metabolic process	0.009 %	-1.3461	0.88	0.27
GO:0050793	regulation of developmental process	2.577 %	-1.7491	0.70	0.29
GO:0010380	regulation of chlorophyll biosynthetic process	0.073 %	-1.0644	0.66	0.31
GO:1901463	regulation of tetrapyrrole biosynthetic process	0.078 %	-1.0548	0.70	0.96
GO:0008643	carbohydrate transport	0.630 %	-1.8736	0.86	0.32
GO:0048829	root cap development	0.065 %	-1.0997	0.81	0.33
GO:0090691	formation of plant organ boundary	0.069 %	-1.0692	0.82	0.33
GO:0010199	organ boundary specification between lateral organs and the meristem	0.039 %	-1.1503	0.82	0.96
GO:0036293	response to decreased oxygen levels	0.281 %	-1.4259	0.90	0.33
GO:0034059	response to anoxia	0.030 %	-1.4315	0.91	0.84
GO:0042182	ketone catabolic process	0.043 %	-1.6623	0.77	0.36
GO:0042181	ketone biosynthetic process	0.134 %	-1.3182	0.72	0.74
GO:0042180	cellular ketone metabolic process	0.470 %	-1.3276	0.78	0.37
GO:0006560	proline metabolic process	0.065 %	-1.0791	0.75	0.38
GO:0006561	proline biosynthetic process	0.043 %	-1.1104	0.72	0.74
GO:0031326	regulation of cellular biosynthetic process	12.339 %	-1.5949	0.66	0.39
GO:0031323	regulation of cellular metabolic process	13.906 %	-1.0686	0.69	0.80
GO:0050794	regulation of cellular process	22.244 %	-1.1772	0.72	0.61
GO:0050789	regulation of biological process	24.333 %	-1.3568	0.77	0.51
GO:0009889	regulation of biosynthetic process	12.408 %	-1.5323	0.68	0.78
GO:0070482	response to oxygen levels	0.285 %	-1.4178	0.91	0.40
GO:0051098	regulation of binding	0.039 %	-1.7531	0.85	0.40
GO:0009845	seed germination	0.600 %	-1.2597	0.78	0.41
GO:0090351	seedling development	0.660 %	-1.1888	0.77	0.53
GO:0003002	regionalization	0.617 %	-1.1935	0.78	0.41
GO:0010375	stomatal complex patterning	0.047 %	-1.1686	0.81	0.76
GO:0007389	pattern specification process	0.742 %	-1.0573	0.79	0.42
GO:0009746	response to hexose	0.220 %	-1.3993	0.83	0.46
GO:0009757	hexose mediated signaling	0.026 %	-1.3005	0.69	0.86
GO:0042593	glucose homeostasis	0.095 %	-1.0791	0.76	0.88
GO:0034284	response to monosaccharide	0.237 %	-1.3515	0.84	0.86
GO:0010255	glucose mediated signaling pathway	0.022 %	-1.3005	0.62	0.83
GO:0071331	cellular response to hexose stimulus	0.073 %	-1.1159	0.80	1.00
GO:0071326	cellular response to monosaccharide stimulus	0.073 %	-1.1159	0.81	0.90
GO:0001678	cellular glucose homeostasis	0.091 %	-1.1214	0.71	0.97
GO:0071333	cellular response to glucose stimulus	0.069 %	-1.1214	0.60	0.95
GO:0006811	ion transport	4.174 %	-1.1034	0.93	0.46
GO:0006970	response to osmotic stress	2.512 %	-1.1604	0.90	0.49
GO:0010087	phloem or xylem histogenesis	0.272 %	-1.2479	0.85	0.49
GO:0051094	positive regulation of developmental process	0.436 %	-1.7240	0.69	0.50
GO:0010455	positive regulation of cell fate commitment	0.013 %	-1.6623	0.70	0.72

L.G0:0010453	regufation of celf (ate commftment	0.047%	-t=l	-1.6232	0.69	0.54
L.G0:0051240	positive regufation o(mufticeffufar organismaf process	0.401%	·¦;;;¦	-1.0234	0.69	0.72
!..G0:00481!31	regufation of shoot system development	0.850%	·¦;;;¦	-1.2777	0.63	0.80
¦...	positive regufation of post-embryonic development	0.319%	-{:¡1	-1.1489	0.64	0.79
·	regufation of post-embryonic development	1.303%	-{:¡1	-1.2243	0.62	0.87
	positive regufation of ceU differentiation	0.030%	-{:¡1	-1.2754	0.68	0.76
·	regufation of flower devefopment	0.609%	-t=l	-1.4790	0.63	0.68

Biological Process (339) Tag Clouds

Scatterplot & Table Interactive Graph TreeMap

GROUP 11

Tip: your resulting list of GO terms seems to be quite long. If you want to reduce it further, press the Back button in your browser and choose a different setting for the "allowed similarity" parameter.

Hide/show dispensable GO terms

Export results to text table (CSV)

Make R script for plotting

term ID	description	frequency	pin?	$\log_{10}$ p-value	uniqueness	dispensability
GO:0006200	(obsolete) ATP catabolic process	0.562 %		-1.1235	0.99	0.00
GO:0022610	biological adhesion	0.060 %		-2.0612	0.99	0.00
GO:0023052	signaling	8.908 %		-3.3859	0.99	0.00
GO:0033993	response to lipid	3.267 %		-4.2578	0.83	0.00
GO:0071229	*cellular response to acid chemical*	*2.158 %*		*-1.6224*	*0.79*	*0.79*
GO:1901701	*cellular response to oxygen-containing compound*	*2.788 %*		*-2.4644*	*0.78*	*0.82*
GO:1901700	*response to oxygen-containing compound*	*6.504 %*		*-2.8746*	*0.84*	*0.70*
GO:0097305	*response to alcohol*	*2.469 %*		*-2.6429*	*0.83*	*0.63*
GO:0097306	*cellular response to alcohol*	*1.170 %*		*-2.1932*	*0.79*	*0.88*
GO:0048545	*response to steroid hormone*	*0.306 %*		*-1.4930*	*0.84*	*0.72*
GO:0032870	*cellular response to hormone stimulus*	*3.871 %*		*-3.1807*	*0.77*	*0.67*
GO:0071215	*cellular response to abscisic acid stimulus*	*1.170 %*		*-2.1932*	*0.78*	*0.98*
GO:0071367	*cellular response to brassinosteroid stimulus*	*0.315 %*		*-1.4896*	*0.80*	*0.95*
GO:0071365	*cellular response to auxin stimulus*	*0.932 %*		*-1.4585*	*0.80*	*0.78*
GO:0070887	*cellular response to chemical stimulus*	*5.287 %*		*-3.4050*	*0.80*	*0.57*
GO:0071383	*cellular response to steroid hormone stimulus*	*0.306 %*		*-1.4930*	*0.80*	*1.00*

GO:0071396	cellular response to lipid	1.770 %	-2.8215	0.78	0.81
GO:0071407	cellular response to organic cyclic compound	0.798 %	-1.0234	0.80	0.79
GO:0071310	cellular response to organic substance	4.601 %	-2.2459	0.77	0.88
GO:0010033	response to organic substance	8.580 %	-3.0963	0.83	0.68
GO:0001101	response to acid chemical	5.011 %	-2.8409	0.84	0.62
GO:0009755	hormone-mediated signaling pathway	3.617 %	-2.8298	0.59	0.94
GO:0009753	response to jasmonic acid	0.919 %	-1.8875	0.84	0.55
GO:0009742	brassinosteroid mediated signaling pathway	0.306 %	-1.4930	0.66	0.72
GO:0009741	response to brassinosteroid	0.410 %	-1.6767	0.84	0.50
GO:0009738	abscisic acid-activated signaling pathway	1.062 %	-2.0258	0.62	0.87
GO:0009737	response to abscisic acid	2.443 %	-2.6642	0.81	0.85
GO:0009734	auxin-activated signaling pathway	0.868 %	-1.0766	0.64	0.85
GO:0009725	response to hormone	6.871 %	-4.4656	0.81	0.82
GO:0043401	steroid hormone mediated signaling pathway	0.306 %	-1.4930	0.66	1.00
GO:0014070	response to organic cyclic compound	1.606 %	-1.7857	0.85	0.59
GO:0071495	cellular response to endogenous stimulus	3.988 %	-3.0737	0.85	0.75
GO:0043455	regulation of secondary metabolic process	0.173 %	-6.1454	0.75	0.00
GO:0044550	secondary metabolite biosynthetic process	1.183 %	-2.8904	0.80	0.72
GO:0009698	phenylpropanoid metabolic process	0.505 %	-1.2917	0.79	0.80
GO:0009699	phenylpropanoid biosynthetic process	0.337 %	-1.0231	0.77	0.76
GO:0010023	proanthocyanidin biosynthetic process	0.035 %	-1.0706	0.79	0.77
GO:2000762	regulation of phenylpropanoid metabolic process	0.069 %	-2.8931	0.73	0.88
GO:1900376	regulation of secondary metabolite biosynthetic process	0.078 %	-3.8630	0.75	0.57
GO:0044699	single-organism process	41.709 %	-2.2842	1.00	0.00
GO:0050896	response to stimulus	25.943 %	-2.2406	1.00	0.00
GO:0065007	biological regulation	28.554 %	-1.6337	1.00	0.00
GO:0006790	sulfur compound metabolic process	1.420 %	-2.5564	0.94	0.05
GO:0007154	cell communication	9.698 %	-3.8679	0.95	0.07
GO:0043473	pigmentation	0.022 %	-2.8683	0.92	0.07
GO:0005975	carbohydrate metabolic process	4.670 %	-1.4882	0.95	0.07
GO:0000060	protein import into nucleus, translocation	0.039 %	-3.3410	0.86	0.07
GO:1902593	single-organism nuclear import	0.242 %	-1.6786	0.84	0.99
GO:0044744	protein targeting to nucleus	0.246 %	-1.6786	0.91	0.98
GO:0034504	protein localization to nucleus	0.268 %	-1.6051	0.92	0.64
GO:0006606	protein import into nucleus	0.242 %	-1.6786	0.83	0.87
GO:0051170	nuclear import	0.246 %	-1.5577	0.92	0.89
GO:0010191	mucilage metabolic process	0.142 %	-1.4316	0.95	0.08
GO:0045168	cell-cell signaling involved in cell fate commitment	0.168 %	-5.8248	0.68	0.08
GO:0008544	epidermis development	0.069 %	-2.5401	0.81	0.57
GO:0060429	epithelium development	0.082 %	-2.3696	0.81	0.57
GO:0090558	plant epidermis development	1.001 %	-2.8953	0.77	0.71
GO:0010057	trichoblast fate specification	0.009 %	-1.7755	0.70	1.00
GO:0010054	trichoblast differentiation	0.427 %	-1.4655	0.64	0.97
GO:0010053	root epidermal cell differentiation	0.488 %	-1.2015	0.64	0.93
GO:0010063	positive regulation of trichoblast fate specification	0.009 %	-1.7755	0.61	0.85
GO:0010061	regulation of trichoblast fate specification	0.009 %	-1.7755	0.62	1.00
GO:0042660	positive regulation of cell fate specification	0.009 %	-1.7248	0.65	0.96
GO:0042659	regulation of cell fate specification	0.017 %	-1.7248	0.65	0.75
GO:0010090	trichome morphogenesis	0.246 %	-3.9345	0.67	0.80
GO:0010091	trichome branching	0.121 %	-2.1290	0.69	0.89
GO:0048468	cell development	1.217 %	-2.2268	0.68	0.65
GO:0000902	cell morphogenesis	1.467 %	-1.2343	0.67	0.91
GO:0000904	cell morphogenesis involved in differentiation	0.880 %	-1.9032	0.67	0.81
GO:0010026	trichome differentiation	0.341 %	-4.4514	0.68	0.54
GO:0048469	cell maturation	0.410 %	-1.6159	0.69	0.87
GO:0032989	cellular component morphogenesis	1.618 %	-1.6293	0.67	0.72
GO:0030855	epithelial cell differentiation	0.069 %	-2.7192	0.72	0.98
GO:0048765	root hair cell differentiation	0.406 %	-1.6159	0.63	0.85
GO:0045165	cell fate commitment	0.341 %	-3.5987	0.72	0.58
GO:0048764	trichoblast maturation	0.406 %	-1.6159	0.63	0.99
GO:0010440	stomatal lineage progression	0.069 %	-1.0909	0.70	0.70
GO:0010455	positive regulation of cell fate commitment	0.013 %	-1.3622	0.64	0.89
GO:0010453	regulation of cell fate commitment	0.047 %	-1.3236	0.64	0.81
GO:1903890	positive regulation of plant epidermal cell differentiation	0.009 %	-1.7248	0.66	1.00
GO:0048629	trichome patterning	0.030 %	-5.8248	0.68	0.78
GO:0009957	epidermal cell fate specification	0.026 %	-1.4048	0.71	0.94
GO:0001708	cell fate specification	0.134 %	-1.7021	0.71	0.87
GO:0090626	plant epidermis morphogenesis	0.367 %	-3.4412	0.77	0.81
GO:0090628	plant epidermal cell fate specification	0.009 %	-1.7248	0.75	0.72

GO:0090627	plant epidermal cell differentiation	0.621 %	-1.1612	0.70	0.69
GO:0009913	epidermal cell differentiation	0.069 %	-2.7192	0.72	0.57
GO:1901615	organic hydroxy compound metabolic process	1.265 %	-1.4749	0.96	0.10
GO:0044763	single-organism cellular process	23.034 %	-2.5542	0.84	0.13
GO:0048041	focal adhesion assembly	0.004 %	-3.4689	0.84	0.14
GO:0034329	cell junction assembly	0.026 %	-3.0027	0.84	0.86
GO:0034332	adherens junction organization	0.004 %	-3.4689	0.86	0.78
GO:0034333	adherens junction assembly	0.004 %	-3.4689	0.86	1.00
GO:0007160	cell-matrix adhesion	0.004 %	-3.4689	0.98	1.00
GO:0007155	cell adhesion	0.060 %	-2.0612	0.98	0.85
GO:0031589	cell-substrate adhesion	0.004 %	-3.4689	0.98	0.74
GO:0007045	cell-substrate adherens junction assembly	0.004 %	-3.4689	0.86	1.00
GO:0007044	cell-substrate junction assembly	0.004 %	-3.4689	0.86	0.82
GO:0045216	cell-cell junction organization	0.039 %	-2.6503	0.84	0.97
GO:0030002	cellular anion homeostasis	0.030 %	-1.4781	0.81	0.16
GO:0072502	cellular trivalent inorganic anion homeostasis	0.022 %	-1.4781	0.81	0.96
GO:0030643	cellular phosphate ion homeostasis	0.022 %	-1.4781	0.81	0.75
GO:0030048	actin filament-based movement	0.086 %	-1.4781	0.87	0.17
GO:0051240	positive regulation of multicellular organismal process	0.401 %	-3.1779	0.70	0.18
GO:0051241	negative regulation of multicellular organismal process	0.445 %	-1.7869	0.64	0.70
GO:0048582	positive regulation of post-embryonic development	0.319 %	-1.9189	0.63	0.68
GO:0048578	positive regulation of long-day photoperiodism, flowering	0.030 %	-1.2114	0.60	0.79
GO:1905423	positive regulation of plant organ morphogenesis	0.009 %	-1.7248	0.69	0.73
GO:1900057	positive regulation of leaf senescence	0.039 %	-1.6012	0.67	0.81
GO:0048831	regulation of shoot system development	0.850 %	-1.5423	0.61	0.76
GO:2000067	regulation of root morphogenesis	0.039 %	-1.0513	0.67	0.82
GO:0045339	farnesyl diphosphate catabolic process	0.023 %	-1.7755	0.83	0.19
GO:0033383	geranyl diphosphate metabolic process	0.056 %	-1.2114	0.82	0.62
GO:0043692	monoterpene metabolic process	0.017 %	-1.5669	0.84	0.85
GO:0043693	monoterpene biosynthetic process	0.017 %	-1.5669	0.82	0.56
GO:0045338	farnesyl diphosphate metabolic process	0.078 %	-1.0420	0.82	0.67
GO:0046246	terpene biosynthetic process	0.043 %	-1.0153	0.81	0.89
GO:0009395	phospholipid catabolic process	0.052 %	-1.1976	0.82	0.85
GO:0034330	cell junction organization	0.039 %	-2.6503	0.86	0.20
GO:0009718	anthocyanin-containing compound biosynthetic process	0.121 %	-1.3486	0.84	0.21
GO:0009963	positive regulation of flavonoid biosynthetic process	0.039 %	-1.2257	0.82	0.78
GO:0009411	response to UV	0.427 %	-3.4308	0.89	0.23
GO:0009314	response to radiation	2.892 %	-1.9378	0.88	0.52
GO:0009416	response to light stimulus	2.771 %	-1.9776	0.87	0.76
GO:1905328	plant septum development	0.009 %	-1.1467	0.87	0.23
GO:0010360	negative regulation of anion channel activity	0.017 %	-2.5519	0.72	0.23
GO:0001933	negative regulation of protein phosphorylation	0.134 %	-1.1349	0.73	0.99
GO:0051348	negative regulation of transferase activity	0.151 %	-1.1235	0.86	0.80
GO:0032412	regulation of ion transmembrane transporter activity	0.060 %	-1.3341	0.77	0.98
GO:0032409	regulation of transporter activity	0.060 %	-1.3341	0.79	0.65
GO:0032410	negative regulation of transporter activity	0.017 %	-2.5519	0.73	0.95
GO:0032413	negative regulation of ion transmembrane transporter activity	0.017 %	-2.5519	0.73	1.00
GO:0044070	regulation of anion transport	0.164 %	-1.4597	0.78	0.89
GO:1903792	negative regulation of anion transport	0.017 %	-2.5519	0.74	0.95
GO:0044092	negative regulation of molecular function	1.070 %	-1.2833	0.84	0.55
GO:0032973	amino acid export	0.082 %	-1.1976	0.84	0.70
GO:0042326	negative regulation of phosphorylation	0.138 %	-1.1235	0.73	0.97
GO:0034762	regulation of transmembrane transport	0.216 %	-1.3105	0.78	0.75
GO:0034763	negative regulation of transmembrane transport	0.017 %	-2.5519	0.74	0.95
GO:0010359	regulation of anion channel activity	0.047 %	-1.4655	0.78	0.91
GO:0034765	regulation of ion transmembrane transport	0.207 %	-1.3105	0.77	0.95
GO:0010362	negative regulation of anion channel activity by blue light	0.009 %	-1.4781	0.73	0.96
GO:0010361	regulation of anion channel activity by blue light	0.009 %	-1.4781	0.80	0.85
GO:0034766	negative regulation of ion transmembrane transport	0.017 %	-2.5519	0.73	1.00
GO:0098656	anion transmembrane transport	0.902 %	-1.2528	0.90	0.62
GO:1903960	negative regulation of anion transmembrane transport	0.017 %	-2.5519	0.73	1.00
GO:1903959	regulation of anion transmembrane transport	0.129 %	-1.4655	0.78	0.84
GO:0022898	regulation of transmembrane transporter activity	0.060 %	-1.3341	0.78	1.00
GO:0051051	negative regulation of transport	0.026 %	-2.0740	0.75	0.60
GO:0006469	negative regulation of protein kinase activity	0.134 %	-1.1349	0.71	0.65
GO:0033673	negative regulation of kinase activity	0.134 %	-1.1349	0.71	0.99
GO:0043271	negative regulation of ion transport	0.026 %	-2.0740	0.73	0.98
GO:0043269	regulation of ion transport	0.302 %	-1.1184	0.78	0.85
GO:0048519	negative regulation of biological process	3.716 %	-3.1044	0.83	0.24
GO:0009611	response to wounding	0.816 %	-1.2370	0.90	0.24

GO:0042762	regulation of sulfur metabolic process	0.091 %	-2.9056	0.79	0.24
GO:0009070	serine family amino acid biosynthetic process	0.177 %	-1.7347	0.78	0.91
GO:0044272	sulfur compound biosynthetic process	0.699 %	-2.7805	0.87	0.70
GO:0046500	S-adenosylmethionine metabolic process	0.043 %	-1.0807	0.91	0.57
GO:0019419	sulfate reduction	0.013 %	-1.6012	0.83	0.53
GO:0019344	cysteine biosynthetic process	0.104 %	-1.9887	0.76	0.71
GO:0006534	cysteine metabolic process	0.134 %	-1.8513	0.78	0.87
GO:0006535	cysteine biosynthetic process from serine	0.065 %	-1.0609	0.77	0.93
GO:0006556	S-adenosylmethionine biosynthetic process	0.017 %	-1.2257	0.89	0.54
GO:0000103	sulfate assimilation	0.060 %	-1.0909	0.91	0.58
GO:0010675	regulation of cellular carbohydrate metabolic process	0.125 %	-3.8494	0.79	0.25
GO:0019760	glucosinolate metabolic process	0.514 %	-2.7534	0.75	0.91
GO:0019758	glycosinolate biosynthetic process	0.194 %	-3.5198	0.75	1.00
GO:0019757	glycosinolate metabolic process	0.514 %	-2.7534	0.75	0.99
GO:0019761	glucosinolate biosynthetic process	0.194 %	-3.5198	0.75	0.86
GO:0019751	polyol metabolic process	0.246 %	-1.3735	0.84	0.82
GO:0019400	alditol metabolic process	0.117 %	-2.5519	0.79	0.89
GO:0010581	regulation of starch biosynthetic process	0.013 %	-1.3425	0.74	0.79
GO:0006067	ethanol metabolic process	0.004 %	-1.3425	0.87	0.75
GO:0006066	alcohol metabolic process	0.505 %	-1.7240	0.83	0.72
GO:0010439	regulation of glucosinolate biosynthetic process	0.026 %	-4.0031	0.69	0.83
GO:2000904	regulation of starch metabolic process	0.043 %	-1.1235	0.80	0.86
GO:0006069	ethanol oxidation	0.004 %	-1.3425	0.87	0.63
GO:0010411	xyloglucan metabolic process	0.259 %	-1.6264	0.90	0.62
GO:0006071	glycerol metabolic process	0.117 %	-2.5519	0.79	0.59
GO:0010410	hemicellulose metabolic process	0.423 %	-1.0016	0.90	0.92
GO:0009969	xyloglucan biosynthetic process	0.091 %	-1.0329	0.78	0.84
GO:0043255	regulation of carbohydrate biosynthetic process	0.117 %	-3.3144	0.73	0.92
GO:0060867	fruit abscission	0.004 %	-1.6795	0.82	0.26
GO:0010227	floral organ abscission	0.112 %	-1.3198	0.74	0.79
GO:0060862	negative regulation of floral organ abscission	0.004 %	-1.4522	0.65	0.79
GO:0060860	regulation of floral organ abscission	0.004 %	-1.4522	0.70	0.79
GO:0019748	secondary metabolic process	1.938 %	-3.1971	0.84	0.27
GO:0009767	photosynthetic electron transport chain	0.224 %	-1.0341	0.83	0.28
GO:0006805	xenobiotic metabolic process	0.009 %	-1.4048	0.85	0.29
GO:0042178	xenobiotic catabolic process	0.004 %	-1.5056	0.85	0.96
GO:0071466	cellular response to xenobiotic stimulus	0.009 %	-1.4048	0.86	0.96
GO:0043619	regulation of transcription from RNA polymerase II promoter in response to oxidative stress	0.004 %	-1.3830	0.76	0.29
GO:0043618	regulation of transcription from RNA polymerase II promoter in response to stress	0.009 %	-1.1015	0.77	0.92
GO:0098727	maintenance of cell number	0.216 %	-1.2402	0.84	0.29
GO:0010077	maintenance of inflorescence meristem identity	0.039 %	-2.7327	0.76	0.30
GO:0019827	stem cell population maintenance	0.216 %	-1.2402	0.78	0.97
GO:0048510	regulation of timing of transition from vegetative to reproductive phase	0.164 %	-1.2402	0.67	0.65
GO:0048506	regulation of timing of meristematic phase transition	0.164 %	-1.2402	0.67	0.95
GO:0010582	floral meristem determinacy	0.052 %	-2.1136	0.73	0.70
GO:0010073	meristem maintenance	0.427 %	-1.1890	0.73	0.78
GO:0010074	maintenance of meristem identity	0.155 %	-1.5777	0.74	0.86
GO:0010076	maintenance of floral meristem identity	0.026 %	-1.3425	0.77	0.80
GO:0010022	meristem determinacy	0.056 %	-2.1136	0.76	0.72
GO:0048583	regulation of response to stimulus	2.521 %	-2.4327	0.77	0.31
GO:0048354	mucilage biosynthetic process involved in seed coat development	0.056 %	-3.0435	0.73	0.31
GO:0010192	mucilage biosynthetic process	0.121 %	-1.8043	0.91	0.95
GO:0010214	seed coat development	0.168 %	-1.0557	0.75	0.53
GO:0048359	mucilage metabolic process involved in seed coat development	0.086 %	-2.0003	0.74	0.90
GO:0071249	cellular response to nitrate	0.017 %	-1.0909	0.85	0.31
GO:1901140	p-coumaryl alcohol transport	0.004 %	-1.0068	0.88	0.31
GO:0042335	cuticle development	0.082 %	-1.6337	0.80	0.32
GO:0044710	single-organism metabolic process	18.390 %	-1.5711	0.85	0.32
GO:0080051	cutin transport	0.009 %	-1.4522	0.88	0.32
GO:0010160	formation of animal organ boundary	0.055 %	-1.0068	0.77	0.33
GO:0009838	abscission	0.125 %	-1.3013	0.79	0.33
GO:0007584	response to nutrient	0.047 %	-1.8610	0.87	0.33
GO:0033273	response to vitamin	0.026 %	-1.1976	0.87	0.87
GO:0031667	response to nutrient levels	0.639 %	-1.6182	0.88	0.76
GO:0006995	cellular response to nitrogen starvation	0.073 %	-1.1467	0.83	0.79
GO:0009970	cellular response to sulfate starvation	0.052 %	-1.7687	0.84	0.76
GO:0010266	response to vitamin B1	0.009 %	-1.6012	0.86	0.59
GO:0021700	developmental maturation	0.794 %	-2.4510	0.78	0.33
GO:0009719	response to endogenous stimulus	7.359 %	-3.7963	0.88	0.33
GO:0043620	regulation of DNA-templated transcription in response to stress	0.013 %	-1.0153	0.77	0.33
GO:0051262	protein tetramerization	0.056 %	-2.1992	0.94	0.35

GO:0051259	protein oligomerization	0.224 %	-1.3293	0.93	0.63
GO:0051289	protein homotetramerization	0.030 %	-1.1588	0.94	0.78
GO:0010048	vernalization response	0.065 %	-2.5067	0.91	0.36
GO:0010219	regulation of vernalization response	0.022 %	-1.5351	0.80	0.52
GO:0010220	positive regulation of vernalization response	0.013 %	-1.5351	0.79	0.84
GO:0007267	cell-cell signaling	0.371 %	-3.8845	0.81	0.37
GO:0009605	response to external stimulus	6.366 %	-1.3445	0.88	0.37
GO:0009627	systemic acquired resistance	0.281 %	-1.4896	0.89	0.38
GO:0002764	immune response-regulating signaling pathway	0.022 %	-1.1713	0.74	0.59
GO:0046459	short-chain fatty acid metabolic process	0.013 %	-1.2558	0.83	0.38
GO:0060151	peroxisome localization	0.009 %	-1.2717	0.94	0.39
GO:0051646	mitochondrion localization	0.017 %	-1.2114	0.93	0.63
GO:0051645	Golgi localization	0.009 %	-1.2717	0.94	0.61
GO:0015749	monosaccharide transport	0.229 %	-1.3056	0.85	0.40
GO:0044765	single-organism transport	5.287 %	-1.5516	0.81	0.61
GO:1902578	single-organism localization	5.481 %	-1.5003	0.82	0.63
GO:0009069	serine family amino acid metabolic process	0.276 %	-1.1689	0.80	0.40
GO:1901607	alpha-amino acid biosynthetic process	0.833 %	-1.0726	0.76	0.80
GO:0080127	fruit septum development	0.004 %	-1.2717	0.80	0.41
GO:0002215	defense response to nematode	0.013 %	-1.3236	0.91	0.42
GO:2000068	regulation of defense response to insect	0.013 %	-1.1235	0.81	0.42
GO:0006694	steroid biosynthetic process	0.358 %	-1.4483	0.82	0.42
GO:0008202	steroid metabolic process	0.427 %	-1.0495	0.84	0.53
GO:0010646	regulation of cell communication	1.139 %	-2.0292	0.80	0.42
GO:0009888	tissue development	2.326 %	-1.8763	0.80	0.42
GO:0048869	cellular developmental process	4.148 %	-1.1434	0.70	0.52
GO:0010540	basipetal auxin transport	0.078 %	-1.1505	0.80	0.42
GO:0044711	single-organism biosynthetic process	7.549 %	-1.3074	0.80	0.42
GO:0051716	cellular response to stimulus	12.637 %	-2.5278	0.84	0.43
GO:0009628	response to abiotic stimulus	7.946 %	-2.0598	0.88	0.43
GO:0023051	regulation of signaling	1.126 %	-2.0411	0.82	0.44
GO:0044700	single organism signaling	8.899 %	-3.3932	0.83	0.64
GO:0007165	signal transduction	8.731 %	-2.1565	0.61	0.91
GO:0050794	regulation of cellular process	22.244 %	-2.1930	0.76	0.45
GO:0031326	regulation of cellular biosynthetic process	12.339 %	-1.0991	0.71	0.59
GO:0050789	regulation of biological process	24.333 %	-1.9075	0.82	0.61
GO:0009889	regulation of biosynthetic process	12.408 %	-1.0627	0.74	0.78
GO:0006109	regulation of carbohydrate metabolic process	0.199 %	-2.8552	0.81	0.45
GO:0044723	single-organism carbohydrate metabolic process	2.283 %	-1.3723	0.81	0.75
GO:0044262	cellular carbohydrate metabolic process	1.817 %	-2.2356	0.90	0.57
GO:0048438	floral whorl development	0.669 %	-2.3954	0.72	0.47
GO:0090567	reproductive shoot system development	2.110 %	-1.8142	0.70	0.72
GO:0048467	gynoecium development	0.315 %	-2.3406	0.73	0.71
GO:0048440	carpel development	0.268 %	-1.2530	0.72	0.85
GO:0048437	floral organ development	0.872 %	-1.5547	0.71	0.88
GO:2000241	regulation of reproductive process	1.092 %	-1.0502	0.79	0.53
GO:0048480	stigma development	0.004 %	-1.2558	0.79	0.51
GO:0048367	shoot system development	3.599 %	-1.0778	0.71	0.59
GO:0009908	flower development	2.059 %	-1.9415	0.69	0.85
GO:0002213	defense response to insect	0.069 %	-1.4153	0.90	0.47
GO:0090436	leaf pavement cell development	0.017 %	-1.2717	0.73	0.47
GO:0045596	negative regulation of cell differentiation	0.052 %	-3.3805	0.58	0.47
GO:0048523	negative regulation of cellular process	2.719 %	-3.2363	0.70	0.68
GO:0023057	negative regulation of signaling	0.345 %	-3.3763	0.69	0.56
GO:0031324	negative regulation of cellular metabolic process	1.929 %	-1.4160	0.68	0.85
GO:1902679	negative regulation of RNA biosynthetic process	1.057 %	-1.6969	0.67	0.99
GO:0031327	negative regulation of cellular biosynthetic process	1.429 %	-1.2834	0.67	0.98
GO:0080050	regulation of seed development	0.129 %	-1.6410	0.64	0.70
GO:0048584	positive regulation of response to stimulus	0.902 %	-1.1944	0.74	0.74
GO:0051253	negative regulation of RNA metabolic process	1.062 %	-1.6879	0.68	0.96
GO:0048581	negative regulation of post-embryonic development	0.354 %	-1.3590	0.57	0.80
GO:0048585	negative regulation of response to stimulus	0.699 %	-1.8730	0.68	0.74
GO:2000692	negative regulation of seed maturation	0.017 %	-2.7620	0.62	0.65
GO:0045934	negative regulation of nucleobase-containing compound metabolic process	1.139 %	-1.5644	0.68	0.92
GO:0010648	negative regulation of cell communication	0.345 %	-3.3763	0.72	0.53
GO:0045892	negative regulation of transcription, DNA-templated	0.958 %	-1.6969	0.67	0.77
GO:0010629	negative regulation of gene expression	1.601 %	-1.0729	0.71	0.91
GO:0010605	negative regulation of macromolecule metabolic process	2.171 %	-1.3204	0.70	0.94
GO:0097438	exit from dormancy	0.009 %	-1.2404	0.83	0.70
GO:0010558	negative regulation of macromolecule biosynthetic process	1.355 %	-1.3723	0.69	0.96
GO:1900033	negative regulation of trichome patterning	0.022 %	-1.9782	0.54	0.91
GO:1900032	regulation of trichome patterning	0.022 %	-1.9782	0.59	0.96

GO:2000243	positive regulation of reproductive process	0.246 %	-1.3765	0.78	0.74
GO:2000242	negative regulation of reproductive process	0.220 %	-2.1290	0.72	0.54
GO:0048497	maintenance of floral organ identity	0.026 %	-2.3658	0.57	0.76
GO:0010431	seed maturation	0.272 %	-1.0311	0.73	0.70
GO:2000034	regulation of seed maturation	0.060 %	-2.0363	0.65	0.83
GO:2000033	regulation of seed dormancy process	0.039 %	-1.1467	0.65	0.90
GO:1903888	regulation of plant epidermal cell differentiation	0.009 %	-1.7248	0.67	0.71
GO:0010454	negative regulation of cell fate commitment	0.022 %	-1.9782	0.59	0.91
GO:1902039	negative regulation of seed dormancy process	0.009 %	-1.4048	0.63	0.82
GO:0048838	release of seed from dormancy	0.009 %	-1.2404	0.78	0.65
GO:2000113	negative regulation of cellular macromolecule biosynthetic process	1.256 %	-1.3723	0.67	0.94
GO:0045595	regulation of cell differentiation	0.250 %	-1.3139	0.62	0.62
GO:0009787	regulation of abscisic acid-activated signaling pathway	0.350 %	-1.9400	0.63	0.93
GO:0009788	negative regulation of abscisic acid-activated signaling pathway	0.160 %	-2.9053	0.58	0.73
GO:1903507	negative regulation of nucleic acid-templated transcription	1.057 %	-1.6969	0.67	0.98
GO:0040034	regulation of development, heterochronic	0.224 %	-1.0589	0.71	0.62
GO:0009892	negative regulation of metabolic process	2.352 %	-1.1130	0.71	0.86
GO:0009890	negative regulation of biosynthetic process	1.450 %	-1.2662	0.70	0.90
GO:0090700	maintenance of plant organ identity	0.026 %	-2.3658	0.58	0.92
GO:0051094	positive regulation of developmental process	0.436 %	-2.9911	0.68	0.55
GO:0051093	negative regulation of developmental process	0.492 %	-3.0628	0.62	0.71
GO:0009968	negative regulation of signal transduction	0.332 %	-2.4696	0.61	0.93
GO:0009966	regulation of signal transduction	1.109 %	-1.4955	0.65	0.87
GO:0080154	regulation of fertilization	0.047 %	-1.8995	0.77	0.65
GO:0080155	regulation of double fertilization forming a zygote and endosperm	0.030 %	-1.8995	0.78	0.79
GO:0009939	positive regulation of gibberellic acid mediated signaling pathway	0.026 %	-1.1976	0.68	0.64
GO:0009910	negative regulation of flower development	0.164 %	-1.4540	0.57	0.89
GO:0009909	regulation of flower development	0.609 %	-1.2683	0.60	0.80
GO:1901420	negative regulation of response to alcohol	0.160 %	-2.9053	0.67	0.88
GO:0051172	negative regulation of nitrogen compound metabolic process	1.433 %	-1.2662	0.71	0.89
GO:0009991	response to extracellular stimulus	0.777 %	-1.3090	0.89	0.48
GO:0042221	response to chemical	12.434 %	-2.2676	0.87	0.48
GO:0019605	butyrate metabolic process	0.009 %	-1.2883	0.83	0.49
GO:0019379	sulfate assimilation, phosphoadenylyl sulfate reduction by phosphoadenylyl-sulfate reductase (thioredoxin)	0.004 %	-1.8995	0.84	0.49
GO:0043481	anthocyanin accumulation in tissues in response to UV light	0.017 %	-2.9312	0.77	0.49
GO:0010224	response to UV-B	0.298 %	-1.7723	0.89	0.75
GO:0043478	pigment accumulation in response to UV light	0.017 %	-2.9312	0.83	0.97
GO:0043480	pigment accumulation in tissues	0.017 %	-2.9312	0.78	0.97
GO:0043479	pigment accumulation in tissues in response to UV light	0.017 %	-2.9312	0.77	1.00
GO:0043476	pigment accumulation	0.022 %	-2.9312	0.84	0.99
GO:1901419	regulation of response to alcohol	0.350 %	-1.9400	0.73	0.49

Biological Process (238) Tag Clouds

Scatterplot & Table Interactive Graph TreeMap

GROUP 12

Tip: your resulting list of GO terms seems to be quite long. If you want to reduce it further, press the Back button in your browser and choose a different setting for the "allowed similarity" parameter.

Hide/show dispensable GO terms

Export results to text table (CSV)

Make R script for plotting

term ID	description	frequency	pin?	$\log_{10}$ p-value	uniqueness	dispensability
GO:0000003	reproduction	6.655 %		-1.7315	1.00	0.00
GO:0002376	immune system process	1.532 %		-1.4322	0.99	0.00
GO:0006184	(obsolete) GTP catabolic process	0.562 %		-2.0041	0.99	0.00
GO:0006884	cell volume homeostasis	0.009 %		-6.2084	0.83	0.00
GO:0009992	*cellular water homeostasis*	*0.004 %*		*-6.3001*	*0.84*	*0.71*
GO:0032535	*regulation of cellular component size*	*0.319 %*		*-2.5373*	*0.80*	*0.90*
GO:0008361	*regulation of cell size*	*0.099 %*		*-3.3205*	*0.81*	*0.70*
GO:0009813	flavonoid biosynthetic process	0.332 %		-3.7257	0.91	0.00
GO:0031540	*regulation of anthocyanin biosynthetic process*	*0.052 %*		*-1.1072*	*0.80*	*0.90*
GO:0009718	*anthocyanin-containing compound biosynthetic process*	*0.121 %*		*-3.8259*	*0.84*	*0.88*
GO:0009963	*positive regulation of flavonoid biosynthetic process*	*0.039 %*		*-1.3280*	*0.85*	*0.81*
GO:0009962	*regulation of flavonoid biosynthetic process*	*0.095 %*		*-1.8160*	*0.85*	*0.86*
GO:0046283	*anthocyanin-containing compound metabolic process*	*0.190 %*		*-2.9659*	*0.85*	*0.91*
GO:0023052	signaling	8.908 %		-2.7236	0.99	0.00
GO:0032501	multicellular organismal process	11.360 %		-2.0236	0.99	0.00
GO:0040007	growth	2.529 %		-2.4503	0.99	0.00
GO:0044699	single-organism process	41.709 %		-1.6907	1.00	0.00

GO:0044706	multi-multicellular organism process	1.075 %	-3.4026	0.84	0.00
GO:0044703	multi-organism reproductive process	1.610 %	-3.2514	0.89	0.76
GO:0051703	intraspecies interaction between organisms	0.026 %	-2.4829	0.95	0.51
GO:0016032	viral process	0.315 %	-1.9330	0.92	0.64
GO:0048544	recognition of pollen	0.211 %	-3.4539	0.76	0.84
GO:0044764	multi-organism cellular process	0.414 %	-1.5513	0.92	0.65
GO:0010483	pollen tube reception	0.026 %	-2.4829	0.86	0.51
GO:0044419	interspecies interaction between organisms	0.423 %	-2.0524	0.94	0.66
GO:0009875	pollen-pistil interaction	0.237 %	-3.4375	0.80	0.85
GO:0009856	pollination	1.075 %	-3.4026	0.82	0.76
GO:0044403	symbiosis, encompassing mutualism through parasitism	0.406 %	-2.1354	0.94	0.97
GO:0050896	response to stimulus	25.943 %	-2.9393	0.99	0.00
GO:0051704	multi-organism process	3.362 %	-4.8720	0.99	0.00
GO:0065007	biological regulation	28.554 %	-1.9968	0.99	0.00
GO:0070588	calcium ion transmembrane transport	0.246 %	-1.2607	0.94	0.00
GO:0006790	sulfur compound metabolic process	1.420 %	-1.2262	0.94	0.05
GO:0006032	chitin catabolic process	0.073 %	-2.8064	0.86	0.06
GO:0046348	amino sugar catabolic process	0.073 %	-2.8064	0.88	0.91
GO:1901071	glucosamine-containing compound metabolic process	0.078 %	-2.7130	0.91	0.91
GO:1901072	glucosamine-containing compound catabolic process	0.073 %	-2.8064	0.88	1.00
GO:0006030	chitin metabolic process	0.073 %	-2.7821	0.89	0.99
GO:0006026	aminoglycan catabolic process	0.073 %	-2.8064	0.87	0.98
GO:0009812	flavonoid metabolic process	0.414 %	-3.0976	0.96	0.06
GO:0007154	cell communication	9.698 %	-4.1993	0.96	0.07
GO:0009311	oligosaccharide metabolic process	0.466 %	-4.8842	0.80	0.07
GO:0044723	single-organism carbohydrate metabolic process	2.283 %	-2.1459	0.78	0.64
GO:0046351	disaccharide biosynthetic process	0.155 %	-1.4725	0.77	0.87
GO:0046352	disaccharide catabolic process	0.035 %	-1.1246	0.77	0.91
GO:0016052	carbohydrate catabolic process	1.187 %	-1.3674	0.85	0.71
GO:0000272	polysaccharide catabolic process	0.777 %	-1.5243	0.83	0.82
GO:0051275	beta-glucan catabolic process	0.112 %	-1.0444	0.82	0.90
GO:0044247	cellular polysaccharide catabolic process	0.229 %	-2.8025	0.81	0.74
GO:0044275	cellular carbohydrate catabolic process	0.298 %	-3.4383	0.83	0.52
GO:0044262	cellular carbohydrate metabolic process	1.817 %	-1.3499	0.85	0.75
GO:0030245	cellulose catabolic process	0.112 %	-1.0444	0.82	0.90
GO:0045489	pectin biosynthetic process	0.190 %	-1.3356	0.78	0.63
GO:0000023	maltose metabolic process	0.017 %	-2.6699	0.82	0.54
GO:0000024	maltose biosynthetic process	0.004 %	-1.2997	0.82	0.89
GO:0009251	glucan catabolic process	0.229 %	-2.8025	0.84	0.79
GO:0000025	maltose catabolic process	0.009 %	-1.8290	0.80	0.75
GO:0006109	regulation of carbohydrate metabolic process	0.199 %	-1.2640	0.81	0.50
GO:0009312	oligosaccharide biosynthetic process	0.255 %	-1.2953	0.78	0.90
GO:0005976	polysaccharide metabolic process	1.899 %	-1.0441	0.86	0.75
GO:0005984	disaccharide metabolic process	0.285 %	-1.9317	0.77	0.74
GO:0005983	starch catabolic process	0.073 %	-2.1791	0.83	0.87
GO:0005982	starch metabolic process	0.268 %	-1.2506	0.84	0.67
GO:0005975	carbohydrate metabolic process	4.670 %	-3.3400	0.95	0.10
GO:0007568	aging	0.544 %	-2.1281	0.85	0.10
GO:0007166	cell surface receptor signaling pathway	1.204 %	-5.7099	0.72	0.13
GO:0044700	single organism signaling	8.899 %	-2.7293	0.87	0.91
GO:0035556	intracellular signal transduction	3.112 %	-2.5514	0.69	0.56
GO:0007165	signal transduction	8.731 %	-2.7676	0.66	0.75
GO:0008037	cell recognition	0.220 %	-3.4430	0.90	0.15
GO:0010117	photoprotection	0.026 %	-3.0002	0.90	0.16
GO:0009786	regulation of asymmetric cell division	0.017 %	-1.5816	0.87	0.18
GO:0009804	coumarin metabolic process	0.017 %	-1.1818	0.87	0.20
GO:0009611	response to wounding	0.816 %	-4.4082	0.85	0.22
GO:1901698	response to nitrogen compound	1.174 %	-3.4834	0.84	0.23
GO:0042127	regulation of cell proliferation	0.293 %	-1.2087	0.86	0.23
GO:0046148	pigment biosynthetic process	0.639 %	-2.5686	0.86	0.27
GO:0051707	response to other organism	4.968 %	-3.6696	0.81	0.27
GO:0043207	response to external biotic stimulus	4.981 %	-3.6696	0.81	0.94
GO:0080027	response to herbivore	0.043 %	-1.0825	0.87	0.53
GO:0050832	defense response to fungus	2.236 %	-1.5334	0.79	0.84
GO:0009620	response to fungus	2.521 %	-1.9608	0.82	0.85
GO:0009615	response to virus	0.337 %	-2.4073	0.85	0.66
GO:0098542	defense response to other organism	4.044 %	-1.5889	0.78	0.92
GO:0009682	induced systemic resistance	0.125 %	-1.6190	0.82	0.73
GO:0002213	defense response to insect	0.069 %	-2.7236	0.84	0.55
GO:0045087	innate immune response	1.364 %	-1.6603	0.80	0.91
GO:0009814	defense response, incompatible interaction	0.725 %	-2.0432	0.80	0.72
GO:0009817	defense response to fungus, incompatible interaction	0.207 %	-2.2031	0.82	0.76

GO:0009861	jasmonic acid and ethylene-dependent systemic resistance	0.052 %	-1.0372	0.83	0.54
GO:0006955	immune response	1.398 %	-1.6083	0.84	0.96
GO:0042440	pigment metabolic process	0.785 %	-2.1600	0.88	0.28
GO:0046482	para-aminobenzoic acid metabolic process	0.017 %	-2.3734	0.86	0.28
GO:0018874	benzoate metabolic process	0.009 %	-1.2028	0.87	0.70
GO:0035264	multicellular organism growth	0.013 %	-3.2952	0.85	0.29
GO:0016049	cell growth	1.519 %	-1.3523	0.84	0.86
GO:0048589	developmental growth	1.623 %	-2.8882	0.81	0.55
GO:0060560	developmental growth involved in morphogenesis	1.157 %	-1.3477	0.80	0.90
GO:0046620	regulation of organ growth	0.082 %	-1.0825	0.76	0.86
GO:0046622	positive regulation of organ growth	0.017 %	-1.4653	0.78	0.81
GO:0009826	unidimensional cell growth	1.010 %	-1.5677	0.75	0.82
GO:0048639	positive regulation of developmental growth	0.026 %	-1.4653	0.79	0.58
GO:0006351	transcription, DNA-templated	11.709 %	-1.1646	0.87	0.29
GO:0032774	RNA biosynthetic process	11.813 %	-1.1646	0.87	0.83
GO:0097659	nucleic acid-templated transcription	11.761 %	-1.1646	0.87	0.88
GO:0033306	phytol metabolic process	0.030 %	-1.6708	0.87	0.29
GO:1903173	fatty alcohol metabolic process	0.030 %	-1.6708	0.88	0.65
GO:0033037	polysaccharide localization	0.168 %	-1.1841	0.95	0.30
GO:0048530	fruit morphogenesis	0.035 %	-1.5081	0.81	0.31
GO:0042538	hyperosmotic salinity response	0.220 %	-2.4687	0.85	0.32
GO:0071475	cellular hyperosmotic salinity response	0.009 %	-1.2138	0.85	0.77
GO:0071474	cellular hyperosmotic response	0.013 %	-1.2138	0.84	0.79
GO:0006972	hyperosmotic response	0.263 %	-2.2385	0.85	0.60
GO:0009607	response to biotic stimulus	5.158 %	-3.5663	0.85	0.33
GO:0009605	response to external stimulus	6.366 %	-2.8363	0.84	0.35
GO:0090066	regulation of anatomical structure size	0.319 %	-2.5373	0.89	0.35
GO:0055081	anion homeostasis	0.199 %	-1.6832	0.88	0.68
GO:0055082	cellular chemical homeostasis	0.902 %	-2.1305	0.79	0.80
GO:0042592	homeostatic process	2.637 %	-2.4734	0.87	0.56
GO:0019725	cellular homeostasis	1.549 %	-2.4841	0.78	0.68
GO:0048878	chemical homeostasis	1.765 %	-2.1768	0.86	0.86
GO:0042430	indole-containing compound metabolic process	0.384 %	-1.8717	0.91	0.35
GO:0031347	regulation of defense response	0.971 %	-1.6572	0.75	0.36
GO:0031348	negative regulation of defense response	0.203 %	-1.2118	0.75	0.78
GO:0048585	negative regulation of response to stimulus	0.699 %	-1.0108	0.77	0.75
GO:2000068	regulation of defense response to insect	0.013 %	-1.2250	0.80	0.63
GO:0009864	induced systemic resistance, jasmonic acid mediated signaling pathway	0.047 %	-1.0231	0.68	0.69
GO:0080134	regulation of response to stress	1.351 %	-1.4599	0.76	0.80
GO:0009939	positive regulation of gibberellic acid mediated signaling pathway	0.026 %	-1.2997	0.73	0.56
GO:0050794	regulation of cellular process	22.244 %	-2.2515	0.84	0.36
GO:0050789	regulation of biological process	24.333 %	-2.1581	0.88	0.61
GO:0034599	cellular response to oxidative stress	0.453 %	-1.9955	0.78	0.37
GO:0043619	regulation of transcription from RNA polymerase II promoter in response to oxidative stress	0.004 %	-1.4862	0.76	0.50
GO:0043618	regulation of transcription from RNA polymerase II promoter in response to stress	0.009 %	-1.2028	0.77	0.92
GO:0071369	cellular response to ethylene stimulus	0.906 %	-1.5862	0.79	0.55
GO:0070301	cellular response to hydrogen peroxide	0.035 %	-1.0163	0.81	0.76
GO:0009873	ethylene-activated signaling pathway	0.811 %	-1.0890	0.68	0.91
GO:0034614	cellular response to reactive oxygen species	0.207 %	-1.3243	0.79	0.67
GO:0009719	response to endogenous stimulus	7.359 %	-2.5640	0.84	0.37
GO:0080024	indolebutyric acid metabolic process	0.022 %	-1.0825	0.80	0.37
GO:0051241	negative regulation of multicellular organismal process	0.445 %	-1.3902	0.75	0.39
GO:2000692	negative regulation of seed maturation	0.017 %	-1.3280	0.75	0.82
GO:0097438	exit from dormancy	0.009 %	-1.3429	0.87	0.70
GO:2000033	regulation of seed dormancy process	0.039 %	-1.2484	0.75	0.90
GO:1902039	negative regulation of seed dormancy process	0.009 %	-1.5081	0.75	0.52
GO:0048838	release of seed from dormancy	0.009 %	-1.3429	0.83	0.70
GO:0051093	negative regulation of developmental process	0.492 %	-1.2533	0.77	0.59
GO:0006979	response to oxidative stress	1.908 %	-1.5060	0.83	0.39
GO:0010243	response to organonitrogen compound	0.768 %	-3.8498	0.83	0.39
GO:0071230	cellular response to amino acid stimulus	0.095 %	-1.1922	0.81	0.74
GO:0010200	response to chitin	0.544 %	-3.8066	0.82	0.87
GO:0040009	regulation of growth rate	0.013 %	-1.5312	0.90	0.40
GO:0065008	regulation of biological quality	5.991 %	-2.0506	0.89	0.41
GO:0030104	water homeostasis	0.052 %	-4.6610	0.89	0.42
GO:0055062	phosphate ion homeostasis	0.078 %	-2.2144	0.88	0.54
GO:0072506	trivalent inorganic anion homeostasis	0.078 %	-2.2144	0.88	0.87
GO:0042221	response to chemical	12.434 %	-2.4164	0.83	0.43
GO:0006434	seryl-tRNA aminoacylation	0.009 %	-2.0829	0.86	0.43
GO:1901565	organonitrogen compound catabolic process	0.811 %	-1.3093	0.88	0.43
GO:0009057	macromolecule catabolic process	5.050 %	-1.1044	0.89	0.62

GO:0019722	calcium-mediated signaling	0.302 %	-2.0516	0.75	0.44
GO:0019932	second-messenger-mediated signaling	0.319 %	-1.7017	0.75	0.58
GO:0000160	phosphorelay signal transduction system	1.049 %	-1.0487	0.72	0.65
GO:0043620	regulation of DNA-templated transcription in response to stress	0.013 %	-1.1158	0.77	0.44
GO:0019432	triglyceride biosynthetic process	0.104 %	-1.1522	0.84	0.44
GO:0046463	acylglycerol biosynthetic process	0.117 %	-1.1158	0.84	0.99
GO:0046460	neutral lipid biosynthetic process	0.117 %	-1.1158	0.84	0.96
GO:0006022	aminoglycan metabolic process	0.086 %	-2.6699	0.91	0.46
GO:0009751	response to salicylic acid	0.876 %	-3.3451	0.82	0.47
GO:1901700	response to oxygen-containing compound	6.504 %	-4.2690	0.81	0.64
GO:0097305	response to alcohol	2.469 %	-1.6608	0.81	0.64
GO:0010033	response to organic substance	8.580 %	-4.2245	0.80	0.70
GO:0001101	response to acid chemical	5.011 %	-4.9785	0.81	0.51
GO:0009753	response to jasmonic acid	0.919 %	-1.1880	0.82	0.63
GO:0009737	response to abscisic acid	2.443 %	-1.6748	0.80	0.71
GO:0009725	response to hormone	6.871 %	-1.7607	0.79	0.82
GO:0009723	response to ethylene	1.269 %	-1.2305	0.82	0.53
GO:0009414	response to water deprivation	1.390 %	-1.8387	0.79	0.63
GO:0009415	response to water	1.416 %	-1.7974	0.81	0.74
GO:0014070	response to organic cyclic compound	1.606 %	-1.8542	0.82	0.51
GO:0071495	cellular response to endogenous stimulus	3.988 %	-1.1517	0.82	0.64
GO:0022414	reproductive process	6.629 %	-1.7443	0.91	0.47
GO:0044702	single organism reproductive process	5.939 %	-1.2732	0.84	0.91
GO:1900030	regulation of pectin biosynthetic process	0.004 %	-1.8290	0.78	0.48
GO:0006040	amino sugar metabolic process	0.138 %	-2.4032	0.93	0.48
GO:0019760	glucosinolate metabolic process	0.514 %	-2.1162	0.80	0.60
GO:0019757	glycosinolate metabolic process	0.514 %	-2.1162	0.80	0.99
GO:1901136	carbohydrate derivative catabolic process	0.337 %	-2.3635	0.89	0.53
GO:1905393	plant organ formation	0.427 %	-1.2513	0.87	0.48
GO:0010360	negative regulation of anion channel activity	0.017 %	-1.2250	0.85	0.49
GO:0032410	negative regulation of transporter activity	0.017 %	-1.2250	0.85	0.95
GO:0032413	negative regulation of ion transmembrane transporter activity	0.017 %	-1.2250	0.85	1.00
GO:1903792	negative regulation of anion transport	0.017 %	-1.2250	0.85	0.95
GO:0034763	negative regulation of transmembrane transport	0.017 %	-1.2250	0.85	0.95
GO:0034766	negative regulation of ion transmembrane transport	0.017 %	-1.2250	0.85	1.00
GO:1903960	negative regulation of anion transmembrane transport	0.017 %	-1.2250	0.85	1.00
GO:0099402	plant organ development	3.763 %	-2.9390	0.77	0.49
GO:0048528	post-embryonic root development	0.501 %	-2.3734	0.78	0.93
GO:0048527	lateral root development	0.457 %	-2.6589	0.78	0.56
GO:2000280	regulation of root development	0.229 %	-1.4589	0.74	0.68
GO:0022622	root system development	1.895 %	-1.5000	0.79	0.67
GO:0010150	leaf senescence	0.462 %	-2.6452	0.78	0.59
GO:0048464	flower calyx development	0.022 %	-1.1717	0.81	0.63
GO:0048465	corolla development	0.065 %	-1.9768	0.80	0.68
GO:0048442	sepal development	0.022 %	-1.1717	0.81	0.63
GO:0048441	petal development	0.065 %	-1.9768	0.79	0.61
GO:0048448	stamen morphogenesis	0.047 %	-1.1246	0.78	0.76
GO:0048446	petal morphogenesis	0.026 %	-1.2484	0.79	0.94
GO:0048451	petal formation	0.009 %	-1.4653	0.80	0.88
GO:0009653	anatomical structure morphogenesis	3.500 %	-1.3704	0.86	0.57
GO:1900056	negative regulation of leaf senescence	0.043 %	-2.4663	0.74	0.82
GO:1900055	regulation of leaf senescence	0.104 %	-2.2900	0.74	0.87
GO:2000024	regulation of leaf development	0.224 %	-1.9948	0.73	0.74
GO:0048830	adventitious root development	0.022 %	-1.6093	0.83	0.56
GO:0048827	phyllome development	1.921 %	-1.9660	0.77	0.71
GO:0048367	shoot system development	3.599 %	-1.3235	0.77	0.74
GO:0048366	leaf development	1.368 %	-2.3640	0.77	0.80
GO:0048364	root development	1.886 %	-1.5129	0.77	0.83
GO:0090693	plant organ senescence	0.462 %	-2.6418	0.79	0.96
GO:0090696	post-embryonic plant organ development	0.686 %	-2.5951	0.79	0.61
GO:0048731	system development	7.225 %	-1.4442	0.78	0.69
GO:0006950	response to stress	14.156 %	-1.7843	0.83	0.49
GO:0006952	defense response	6.090 %	-1.9137	0.81	0.50
GO:0051716	cellular response to stimulus	12.637 %	-1.5062	0.81	0.50
GO:0010398	xylogalacturonan metabolic process	0.004 %	-1.5312	0.90	0.50
GO:0052544	defense response by callose deposition in cell wall	0.069 %	-1.3703	0.75	0.50
GO:0010215	cellulose microfibril organization	0.073 %	-1.1619	0.87	0.50
GO:0070726	cell wall assembly	0.082 %	-1.0444	0.93	0.65
GO:0052386	cell wall thickening	0.134 %	-1.1994	0.93	0.74
GO:0052545	callose localization	0.155 %	-1.1841	0.93	0.93

GO:0052542	defense response by callose deposition	0.091 %	-1.2640	0.81	0.95
GO:0052543	callose deposition in cell wall	0.117 %	-1.2882	0.88	0.96
GO:0072599	establishment of protein localization to endoplasmic reticulum	0.121 %	-1.1336	0.94	0.90
GO:0071668	plant-type cell wall assembly	0.082 %	-1.0444	0.93	0.99
GO:0006613	cotranslational protein targeting to membrane	0.091 %	-1.2733	0.84	0.90
GO:0045047	protein targeting to ER	0.121 %	-1.1336	0.93	0.97
GO:0006614	SRP-dependent cotranslational protein targeting to membrane	0.078 %	-1.2733	0.84	0.52
GO:0052482	defense response by cell wall thickening	0.069 %	-1.3703	0.79	0.91

Biological Process (304) Tag Clouds

Scatterplot & Table Interactive Graph TreeMap

GROUP 13

Tip: your resulting list of GO terms seems to be quite long. If you want to reduce it further, press the Back button in your browser and choose a different setting for the "allowed similarity" parameter.

Hide/show dispensable GO terms

Export results to text table (CSV)

Make R script for plotting

term ID	description	frequency	pin?	log_{10} p-value	uniqueness	dispensability
GO:0002376	immune system process	1.532 %		-1.1401	0.99	0.00
GO:0006200	(obsolete) ATP catabolic process	0.562 %		-1.2670	0.99	0.00
GO:0010200	response to chitin	0.544 %		-8.1196	0.81	0.00
GO:0071229	*cellular response to acid chemical*	*2.158 %*		*-2.4748*	*0.74*	*0.79*
GO:1901701	*cellular response to oxygen-containing compound*	*2.788 %*		*-1.6601*	*0.73*	*0.82*
GO:1901700	*response to oxygen-containing compound*	*6.504 %*		*-6.8400*	*0.79*	*0.61*
GO:0097305	*response to alcohol*	*2.469 %*		*-3.4871*	*0.79*	*0.65*
GO:0097306	*cellular response to alcohol*	*1.170 %*		*-1.5210*	*0.74*	*0.88*
GO:0032870	*cellular response to hormone stimulus*	*3.871 %*		*-4.1557*	*0.72*	*0.57*
GO:0071215	*cellular response to abscisic acid stimulus*	*1.170 %*		*-1.5210*	*0.74*	*0.88*
GO:0033993	*response to lipid*	*3.267 %*		*-3.4617*	*0.79*	*0.67*
GO:0009642	*response to light intensity*	*0.587 %*		*-1.0555*	*0.84*	*0.79*
GO:0009644	*response to high light intensity*	*0.324 %*		*-1.4682*	*0.84*	*0.74*
GO:0070887	*cellular response to chemical stimulus*	*5.287 %*		*-3.1701*	*0.74*	*0.64*
GO:0071369	*cellular response to ethylene stimulus*	*0.906 %*		*-2.5709*	*0.75*	*0.91*
GO:0071395	*cellular response to jasmonic acid stimulus*	*0.337 %*		*-2.7911*	*0.77*	*0.69*

GO:0071310	cellular response to organic substance	4.601 %	-3.3195	0.72	0.88
GO:0010033	response to organic substance	8.580 %	-6.7593	0.78	0.70
GO:0001101	response to acid chemical	5.011 %	-5.1630	0.79	0.64
GO:0009755	hormone-mediated signaling pathway	3.617 %	-2.4106	0.57	0.94
GO:0009753	response to jasmonic acid	0.919 %	-2.5995	0.80	0.63
GO:0009751	response to salicylic acid	0.876 %	-2.0943	0.80	0.63
GO:0009266	response to temperature stimulus	2.287 %	-2.7890	0.82	0.58
GO:0009737	response to abscisic acid	2.443 %	-3.5093	0.78	0.72
GO:0009725	response to hormone	6.871 %	-5.2854	0.76	0.82
GO:0009723	response to ethylene	1.269 %	-2.6717	0.80	0.65
GO:0009314	response to radiation	2.892 %	-1.8775	0.82	0.65
GO:0071446	cellular response to salicylic acid stimulus	0.242 %	-1.9269	0.77	0.76
GO:0009867	jasmonic acid mediated signaling pathway	0.319 %	-1.9232	0.65	0.81
GO:0009863	salicylic acid mediated signaling pathway	0.211 %	-1.1878	0.66	0.79
GO:0009864	induced systemic resistance, jasmonic acid mediated signaling pathway	0.047 %	-1.0644	0.61	0.85
GO:0009873	ethylene-activated signaling pathway	0.811 %	-3.0344	0.63	0.77
GO:0006970	response to osmotic stress	2.512 %	-1.1534	0.80	0.64
GO:0009414	response to water deprivation	1.390 %	-3.9629	0.76	0.54
GO:0009415	response to water	1.416 %	-3.8949	0.77	0.74
GO:0009416	response to light stimulus	2.771 %	-1.9233	0.81	0.63
GO:0010243	response to organonitrogen compound	0.768 %	-6.8980	0.81	0.87
GO:0014070	response to organic cyclic compound	1.606 %	-2.0785	0.81	0.61
GO:0071495	cellular response to endogenous stimulus	3.988 %	-4.0445	0.81	0.75
GO:0019953	sexual reproduction	0.596 %	-1.6355	0.95	0.00
GO:0007276	gamete generation	0.203 %	-1.3799	0.90	0.81
GO:0022412	cellular process involved in reproduction in multicellular organism	0.177 %	-1.4567	0.87	0.79
GO:1902579	multi-organism localization	0.052 %	-1.2037	0.91	0.52
GO:0048235	pollen sperm cell differentiation	0.147 %	-1.6665	0.84	0.70
GO:0048232	male gamete generation	0.177 %	-1.5472	0.90	0.96
GO:0023052	signaling	8.908 %	-1.6985	0.99	0.00
GO:0032502	developmental process	12.689 %	-1.0161	0.99	0.00
GO:0035264	multicellular organism growth	0.013 %	-5.4374	0.93	0.00
GO:0048589	developmental growth	1.623 %	-1.1674	0.91	0.55
GO:0045893	positive regulation of transcription, DNA-templated	1.864 %	-3.8665	0.64	0.00
GO:0032774	RNA biosynthetic process	11.813 %	-4.6489	0.73	0.82
GO:0048522	positive regulation of cellular process	3.582 %	-1.8940	0.74	0.87
GO:0080090	regulation of primary metabolic process	13.681 %	-4.2531	0.69	0.82
GO:0031323	regulation of cellular metabolic process	13.906 %	-4.2727	0.67	0.80
GO:1902680	positive regulation of RNA biosynthetic process	1.869 %	-3.8665	0.64	0.97
GO:0031328	positive regulation of cellular biosynthetic process	2.102 %	-3.4620	0.67	0.97
GO:0031326	regulation of cellular biosynthetic process	12.339 %	-5.5799	0.63	0.87
GO:0031325	positive regulation of cellular metabolic process	2.741 %	-3.0924	0.69	0.95
GO:0051252	regulation of RNA metabolic process	11.463 %	-4.5532	0.62	0.87
GO:2001141	regulation of RNA biosynthetic process	11.282 %	-4.7100	0.59	0.88
GO:0019219	regulation of nucleobase-containing compound metabolic process	11.700 %	-4.3378	0.63	0.84
GO:0051254	positive regulation of RNA metabolic process	1.912 %	-3.8665	0.66	0.96
GO:0045935	positive regulation of nucleobase-containing compound metabolic process	2.007 %	-3.7482	0.67	0.96
GO:0010628	positive regulation of gene expression	2.050 %	-3.6552	0.70	0.90
GO:0006355	regulation of transcription, DNA-templated	11.234 %	-4.7100	0.59	0.76
GO:0006351	transcription, DNA-templated	11.709 %	-4.6489	0.72	0.87
GO:0019222	regulation of metabolic process	14.847 %	-3.4991	0.75	0.54
GO:0050794	regulation of cellular process	22.244 %	-2.7315	0.73	0.63
GO:0050789	regulation of biological process	24.333 %	-2.5516	0.79	0.54
GO:0010604	positive regulation of macromolecule metabolic process	2.672 %	-3.1640	0.70	0.92
GO:0010556	regulation of macromolecule biosynthetic process	12.076 %	-5.0531	0.63	0.87
GO:0010557	positive regulation of macromolecule biosynthetic process	2.007 %	-3.5974	0.67	0.95
GO:0010468	regulation of gene expression	12.706 %	-3.9239	0.67	0.80
GO:2000112	regulation of cellular macromolecule biosynthetic process	12.003 %	-5.0749	0.61	0.86
GO:0060255	regulation of macromolecule metabolic process	14.091 %	-3.6789	0.67	0.83
GO:1903508	positive regulation of nucleic acid-templated transcription	1.869 %	-3.8665	0.64	0.98
GO:1903506	regulation of nucleic acid-templated transcription	11.282 %	-4.7100	0.59	0.89
GO:0097659	nucleic acid-templated transcription	11.761 %	-4.6489	0.73	0.88
GO:0009893	positive regulation of metabolic process	2.892 %	-2.8643	0.72	0.79
GO:0009891	positive regulation of biosynthetic process	2.145 %	-3.4236	0.69	0.89
GO:0009889	regulation of biosynthetic process	12.408 %	-5.4116	0.66	0.54
GO:0051173	positive regulation of nitrogen compound metabolic process	2.119 %	-3.4951	0.71	0.88
GO:0034654	nucleobase-containing compound biosynthetic process	13.056 %	-3.7891	0.75	0.80
GO:0051171	regulation of nitrogen compound metabolic process	12.430 %	-4.0516	0.68	0.80

GO:0050896	response to stimulus	25.943 %	-4.7279	1.00	0.00
GO:0051704	multi-organism process	3.362 %	-3.5740	0.99	0.00
GO:0065007	biological regulation	28.554 %	-2.1532	1.00	0.00
GO:1901804	beta-glucoside metabolic process	0.009 %	-1.6244	0.90	0.06
GO:1901038	*cyanidin 3-O-glucoside metabolic process*	*0.004 %*	*-1.6244*	*0.90*	*0.89*
GO:0071554	cell wall organization or biogenesis	3.168 %	-1.5475	0.94	0.06
GO:0007154	cell communication	9.698 %	-1.8234	0.94	0.08
GO:0009058	biosynthetic process	24.432 %	-1.3965	0.92	0.09
GO:0044262	cellular carbohydrate metabolic process	1.817 %	-2.6302	0.82	0.10
GO:0016051	*carbohydrate biosynthetic process*	*1.442 %*	*-2.0215*	*0.73*	*0.71*
GO:0044247	*cellular polysaccharide catabolic process*	*0.229 %*	*-1.7401*	*0.80*	*0.87*
GO:0044275	*cellular carbohydrate catabolic process*	*0.298 %*	*-1.4328*	*0.82*	*0.75*
GO:0044264	*cellular polysaccharide metabolic process*	*1.187 %*	*-2.2991*	*0.79*	*0.86*
GO:0044042	*glucan metabolic process*	*0.962 %*	*-2.8017*	*0.82*	*0.83*
GO:0030244	*cellulose biosynthetic process*	*0.216 %*	*-1.2072*	*0.71*	*0.85*
GO:0030243	*cellulose metabolic process*	*0.337 %*	*-1.0456*	*0.80*	*0.93*
GO:0045489	*pectin biosynthetic process*	*0.190 %*	*-2.4244*	*0.75*	*0.71*
GO:0045488	*pectin metabolic process*	*0.647 %*	*-1.5256*	*0.83*	*0.88*
GO:0009251	*glucan catabolic process*	*0.229 %*	*-1.7401*	*0.82*	*0.86*
GO:0010393	*galacturonan metabolic process*	*0.652 %*	*-1.5060*	*0.83*	*0.80*
GO:0009311	*oligosaccharide metabolic process*	*0.466 %*	*-1.4822*	*0.80*	*0.77*
GO:0006073	*cellular glucan metabolic process*	*0.937 %*	*-2.8017*	*0.79*	*0.67*
GO:0010411	*xyloglucan metabolic process*	*0.259 %*	*-1.9053*	*0.80*	*0.87*
GO:0010410	*hemicellulose metabolic process*	*0.423 %*	*-1.2570*	*0.80*	*0.92*
GO:0034637	*cellular carbohydrate biosynthetic process*	*0.915 %*	*-1.8706*	*0.71*	*0.84*
GO:0005976	*polysaccharide metabolic process*	*1.899 %*	*-2.2320*	*0.83*	*0.73*
GO:0005983	*starch catabolic process*	*0.073 %*	*-2.2628*	*0.81*	*0.77*
GO:0005982	*starch metabolic process*	*0.268 %*	*-1.3274*	*0.81*	*0.87*
GO:0051865	protein autoubiquitination	0.056 %	-1.1082	0.91	0.12
GO:0072660	maintenance of protein location in plasma membrane	0.004 %	-3.2582	0.75	0.13
GO:0090002	*establishment of protein localization to plasma membrane*	*0.013 %*	*-2.6561*	*0.81*	*1.00*
GO:0044765	*single-organism transport*	*5.287 %*	*-1.7263*	*0.83*	*0.71*
GO:1902582	*single-organism intracellular transport*	*0.790 %*	*-1.7074*	*0.83*	*0.64*
GO:0044802	*single-organism membrane organization*	*0.919 %*	*-1.1201*	*0.84*	*0.69*
GO:0072661	*protein targeting to plasma membrane*	*0.013 %*	*-2.6561*	*0.81*	*0.94*
GO:0032507	*maintenance of protein location in cell*	*0.078 %*	*-2.1649*	*0.73*	*0.83*
GO:0072657	*protein localization to membrane*	*0.259 %*	*-1.6884*	*0.78*	*0.88*
GO:0072658	*maintenance of protein location in membrane*	*0.004 %*	*-3.2582*	*0.76*	*0.71*
GO:0072659	*protein localization to plasma membrane*	*0.013 %*	*-2.6561*	*0.81*	*0.94*
GO:0006605	*protein targeting*	*1.006 %*	*-1.2900*	*0.88*	*0.78*
GO:0006612	*protein targeting to membrane*	*0.168 %*	*-1.8485*	*0.78*	*0.96*
GO:0007009	*plasma membrane organization*	*0.022 %*	*-2.5846*	*0.88*	*0.62*
GO:1902580	*single-organism cellular localization*	*0.842 %*	*-1.6665*	*0.84*	*0.71*
GO:0090150	*establishment of protein localization to membrane*	*0.259 %*	*-1.6884*	*0.78*	*0.88*
GO:0051651	*maintenance of location in cell*	*0.155 %*	*-1.9354*	*0.72*	*0.85*
GO:0045185	*maintenance of protein location*	*0.078 %*	*-2.0245*	*0.79*	*0.67*
GO:0045730	respiratory burst	0.013 %	-1.1575	0.91	0.15
GO:0050826	response to freezing	0.086 %	-5.4463	0.85	0.16
GO:0070417	*cellular response to cold*	*0.147 %*	*-1.6884*	*0.79*	*0.69*
GO:0009409	*response to cold*	*1.580 %*	*-3.5108*	*0.81*	*0.66*
GO:0071497	*cellular response to freezing*	*0.013 %*	*-3.1850*	*0.83*	*0.51*
GO:0018874	benzoate metabolic process	0.009 %	-1.2448	0.86	0.17
GO:0046482	*para-aminobenzoic acid metabolic process*	*0.017 %*	*-1.0785*	*0.85*	*0.70*
GO:0009827	plant-type cell wall modification	0.095 %	-1.9746	0.93	0.21
GO:0042545	*cell wall modification*	*0.561 %*	*-1.4054*	*0.92*	*0.60*
GO:0010467	gene expression	18.304 %	-2.0130	0.88	0.21
GO:0065008	regulation of biological quality	5.991 %	-1.3775	0.83	0.26
GO:2000280	regulation of root development	0.229 %	-1.5380	0.80	0.27
GO:0048509	*regulation of meristem development*	*0.445 %*	*-1.3367*	*0.82*	*0.78*
GO:0048510	*regulation of timing of transition from vegetative to reproductive phase*	*0.164 %*	*-1.5070*	*0.83*	*0.57*
GO:0048506	*regulation of timing of meristematic phase transition*	*0.164 %*	*-1.5070*	*0.83*	*0.95*
GO:0031348	*negative regulation of defense response*	*0.203 %*	*-1.2881*	*0.69*	*0.70*
GO:0048586	*regulation of long-day photoperiodism, flowering*	*0.086 %*	*-1.0575*	*0.69*	*0.90*
GO:0048579	*negative regulation of long-day photoperiodism, flowering*	*0.039 %*	*-1.4006*	*0.67*	*0.57*
GO:0010480	*microsporocyte differentiation*	*0.244 %*	*-1.1488*	*0.86*	*0.52*
GO:0040034	*regulation of development, heterochronic*	*0.224 %*	*-1.3174*	*0.84*	*0.59*
GO:0010036	response to boron-containing substance	0.022 %	-1.1488	0.87	0.27
GO:0000160	phosphorelay signal transduction system	1.049 %	-2.9469	0.67	0.27
GO:0044700	*single organism signaling*	*8.899 %*	*-1.7010*	*0.90*	*0.91*
GO:0035556	*intracellular signal transduction*	*3.112 %*	*-1.4209*	*0.63*	*0.55*
GO:0007165	*signal transduction*	*8.731 %*	*-1.7171*	*0.58*	*0.75*

GO:0010256	endomembrane system organization	0.311 %	-1.2945	0.93	0.28
GO:0000288	nuclear-transcribed mRNA catabolic process, deadenylation-dependent decay	0.164 %	-2.3875	0.84	0.28
GO:0000289	*nuclear-transcribed mRNA poly(A) tail shortening*	*0.043 %*	*-2.3875*	*0.86*	*0.81*
GO:0000956	*nuclear-transcribed mRNA catabolic process*	*0.306 %*	*-1.6884*	*0.83*	*0.93*
GO:0006402	*mRNA catabolic process*	*0.345 %*	*-1.6348*	*0.83*	*0.96*
GO:0006401	*RNA catabolic process*	*0.397 %*	*-1.5426*	*0.83*	*0.86*
GO:0034655	*nucleobase-containing compound catabolic process*	*0.561 %*	*-1.3585*	*0.84*	*0.90*
GO:0006470	protein dephosphorylation	0.811 %	-1.0369	0.88	0.29
GO:0048519	negative regulation of biological process	3.716 %	-1.4270	0.82	0.29
GO:0010440	stomatal lineage progression	0.069 %	-1.2341	0.87	0.30
GO:0010117	photoprotection	0.026 %	-4.9827	0.87	0.30
GO:0009268	response to pH	0.030 %	-2.9696	0.88	0.31
GO:0019747	regulation of isoprenoid metabolic process	0.099 %	-1.4508	0.74	0.32
GO:0019287	*isopentenyl diphosphate biosynthetic process, mevalonate pathway*	*0.039 %*	*-1.4006*	*0.81*	*0.64*
GO:0016104	*triterpenoid biosynthetic process*	*0.030 %*	*-1.1006*	*0.82*	*0.63*
GO:0016114	*terpenoid biosynthetic process*	*0.591 %*	*-1.0032*	*0.77*	*0.78*
GO:0009240	*isopentenyl diphosphate biosynthetic process*	*0.091 %*	*-1.1846*	*0.80*	*0.94*
GO:0010371	*regulation of gibberellin biosynthetic process*	*0.039 %*	*-1.5507*	*0.73*	*0.81*
GO:0006084	*acetyl-CoA metabolic process*	*0.108 %*	*-1.1240*	*0.91*	*0.88*
GO:0010540	basipetal auxin transport	0.078 %	-2.4244	0.76	0.32
GO:0060918	*auxin transport*	*0.406 %*	*-1.5557*	*0.73*	*0.97*
GO:0080024	*indolebutyric acid metabolic process*	*0.022 %*	*-1.1240*	*0.76*	*0.71*
GO:0032350	*regulation of hormone metabolic process*	*0.073 %*	*-1.1575*	*0.78*	*0.67*
GO:0010600	*regulation of auxin biosynthetic process*	*0.052 %*	*-1.5079*	*0.74*	*0.60*
GO:0046885	*regulation of hormone biosynthetic process*	*0.065 %*	*-1.2786*	*0.73*	*0.97*
GO:0010541	*acropetal auxin transport*	*0.013 %*	*-1.0187*	*0.79*	*0.70*
GO:0090354	*regulation of auxin metabolic process*	*0.056 %*	*-1.3154*	*0.76*	*0.96*
GO:0010315	*auxin efflux*	*0.086 %*	*-1.0442*	*0.59*	*0.86*
GO:0009850	*auxin metabolic process*	*0.332 %*	*-1.4730*	*0.78*	*0.80*
GO:0009926	*auxin polar transport*	*0.337 %*	*-1.6522*	*0.73*	*0.85*
GO:0042445	*hormone metabolic process*	*0.695 %*	*-1.0240*	*0.79*	*0.86*
GO:0009914	*hormone transport*	*0.436 %*	*-1.5355*	*0.73*	*0.71*
GO:0098542	defense response to other organism	4.044 %	-4.8089	0.78	0.33
GO:0043207	*response to external biotic stimulus*	*4.981 %*	*-4.8024*	*0.81*	*0.94*
GO:0051707	*response to other organism*	*4.968 %*	*-4.8024*	*0.81*	*0.92*
GO:0080027	*response to herbivore*	*0.043 %*	*-1.1240*	*0.87*	*0.52*
GO:0050832	*defense response to fungus*	*2.236 %*	*-3.7695*	*0.79*	*0.81*
GO:0009620	*response to fungus*	*2.521 %*	*-3.4538*	*0.82*	*0.83*
GO:0009617	*response to bacterium*	*1.748 %*	*-1.5202*	*0.83*	*0.79*
GO:0009991	*response to extracellular stimulus*	*0.777 %*	*-1.2773*	*0.84*	*0.64*
GO:0002213	*defense response to insect*	*0.069 %*	*-2.8475*	*0.85*	*0.54*
GO:0033554	*cellular response to stress*	*3.772 %*	*-1.0296*	*0.76*	*0.52*
GO:0006952	*defense response*	*6.090 %*	*-4.8734*	*0.81*	*0.56*
GO:0042742	*defense response to bacterium*	*1.476 %*	*-1.4186*	*0.80*	*0.77*
GO:0055046	microgametogenesis	0.216 %	-1.4367	0.92	0.33
GO:0048518	positive regulation of biological process	4.398 %	-1.2396	0.81	0.33
GO:0016192	vesicle-mediated transport	1.964 %	-1.3773	0.91	0.34
GO:1902578	*single-organism localization*	*5.481 %*	*-1.6432*	*0.86*	*0.54*
GO:0009719	response to endogenous stimulus	7.359 %	-7.5202	0.83	0.34
GO:0048533	sporocyte differentiation	0.004 %	-1.1488	0.90	0.35
GO:0090305	nucleic acid phosphodiester bond hydrolysis	3.276 %	-1.6939	0.83	0.35
GO:0016070	*RNA metabolic process*	*17.562 %*	*-4.1126*	*0.78*	*0.51*
GO:0009059	*macromolecule biosynthetic process*	*17.035 %*	*-1.6173*	*0.80*	*0.64*
GO:0044249	*cellular biosynthetic process*	*22.844 %*	*-1.5957*	*0.79*	*0.66*
GO:0044271	*cellular nitrogen compound biosynthetic process*	*17.018 %*	*-1.8120*	*0.77*	*0.64*
GO:1901576	*organic substance biosynthetic process*	*22.969 %*	*-1.0235*	*0.81*	*0.72*
GO:0019438	*aromatic compound biosynthetic process*	*14.247 %*	*-2.5885*	*0.77*	*0.60*
GO:0018130	*heterocycle biosynthetic process*	*14.014 %*	*-3.0074*	*0.78*	*0.61*
GO:1901362	*organic cyclic compound biosynthetic process*	*14.769 %*	*-2.7438*	*0.80*	*0.60*
GO:0034645	*cellular macromolecule biosynthetic process*	*16.737 %*	*-1.8411*	*0.76*	*0.61*
GO:0009607	response to biotic stimulus	5.158 %	-5.2543	0.84	0.36
GO:0042538	hyperosmotic salinity response	0.220 %	-3.6111	0.84	0.36
GO:0071470	*cellular response to osmotic stress*	*0.207 %*	*-1.4367*	*0.79*	*0.58*
GO:0071475	*cellular hyperosmotic salinity response*	*0.009 %*	*-1.2557*	*0.83*	*0.77*
GO:0071474	*cellular hyperosmotic response*	*0.013 %*	*-1.2557*	*0.82*	*0.79*
GO:0006972	*hyperosmotic response*	*0.263 %*	*-3.3128*	*0.84*	*0.60*
GO:0010148	transpiration	0.026 %	-2.0596	0.88	0.36
GO:0006833	*water transport*	*0.194 %*	*-1.7829*	*0.87*	*0.86*
GO:0016071	mRNA metabolic process	2.145 %	-1.2620	0.83	0.37
GO:0055062	phosphate ion homeostasis	0.078 %	-2.2982	0.85	0.37
GO:0055081	*anion homeostasis*	*0.199 %*	*-1.7642*	*0.84*	*0.64*

GO:0072506	*trivalent inorganic anion homeostasis*	0.078 %	-2.2982	0.85	0.87
GO:0045229	external encapsulating structure organization	2.577 %	-1.1319	0.92	0.37
GO:0009605	response to external stimulus	6.366 %	-4.6259	0.84	0.37
GO:1901698	response to nitrogen compound	1.174 %	-5.3721	0.82	0.38
GO:0070301	cellular response to hydrogen peroxide	0.035 %	-1.0575	0.79	0.39
GO:0080169	cellular response to boron-containing substance deprivation	0.004 %	-1.7136	0.81	0.39
GO:0033273	*response to vitamin*	0.026 %	-1.3419	0.85	0.90
GO:0080029	*cellular response to boron-containing substance levels*	0.017 %	-1.1488	0.81	0.90
GO:0033591	*response to L-ascorbic acid*	0.017 %	-1.5507	0.83	0.54
GO:0015770	sucrose transport	0.086 %	-1.8266	0.87	0.39
GO:0015772	*oligosaccharide transport*	0.091 %	-1.8266	0.87	0.72
GO:0015766	*disaccharide transport*	0.091 %	-1.8266	0.87	1.00
GO:0034219	*carbohydrate transmembrane transport*	0.332 %	-1.6244	0.86	0.79
GO:0048354	mucilage biosynthetic process involved in seed coat development	0.056 %	-1.5079	0.84	0.40
GO:1990778	protein localization to cell periphery	0.022 %	-2.6561	0.91	0.41
GO:1900030	regulation of pectin biosynthetic process	0.004 %	-1.8720	0.73	0.42
GO:0032885	*regulation of polysaccharide biosynthetic process*	0.043 %	-1.0187	0.70	0.80
GO:0002679	respiratory burst involved in defense response	0.009 %	-1.3703	0.80	0.42
GO:0009611	response to wounding	0.816 %	-3.1485	0.85	0.42
GO:0042044	fluid transport	0.194 %	-1.7829	0.87	0.42
GO:0061024	membrane organization	0.997 %	-1.0500	0.93	0.42
GO:0042221	response to chemical	12.434 %	-6.0234	0.82	0.43
GO:0080034	host response to induction by symbiont of tumor, nodule or growth in host	0.004 %	-1.4167	0.89	0.43
GO:0016137	glycoside metabolic process	0.017 %	-1.0312	0.90	0.43
GO:0051708	intracellular protein transport in other organism involved in symbiotic interaction	0.004 %	-1.4167	0.90	0.43
GO:0010185	regulation of cellular defense response	0.013 %	-3.2582	0.78	0.43
GO:0051245	*negative regulation of cellular defense response*	0.004 %	-3.2582	0.76	0.89
GO:0009626	*plant-type hypersensitive response*	0.341 %	-1.5611	0.70	0.86
GO:0060548	*negative regulation of cell death*	0.112 %	-1.5113	0.74	0.82
GO:0043069	*negative regulation of programmed cell death*	0.082 %	-1.7401	0.74	0.84
GO:0043067	*regulation of programmed cell death*	0.216 %	-1.2945	0.77	0.90
GO:0050776	*regulation of immune response*	0.419 %	-1.5060	0.72	0.98
GO:0010941	*regulation of cell death*	0.298 %	-1.0864	0.77	0.74
GO:0002682	*regulation of immune system process*	0.453 %	-1.4273	0.83	0.64
GO:0010363	*regulation of plant-type hypersensitive response*	0.060 %	-2.4290	0.66	0.56
GO:0045088	*regulation of innate immune response*	0.397 %	-1.5481	0.70	0.84
GO:0002252	*immune effector process*	0.268 %	-1.2850	0.96	0.73
GO:0034050	*host programmed cell death induced by symbiont*	0.345 %	-1.5113	0.87	0.90
GO:0080135	*regulation of cellular response to stress*	0.250 %	-1.7401	0.69	0.63
GO:0009628	response to abiotic stimulus	7.946 %	-3.0194	0.83	0.43
GO:0010225	response to UV-C	0.043 %	-1.2557	0.87	0.44
GO:0016126	sterol biosynthetic process	0.168 %	-1.0714	0.81	0.44
GO:2000113	negative regulation of cellular macromolecule biosynthetic process	1.256 %	-2.6232	0.65	0.45
GO:0048523	*negative regulation of cellular process*	2.719 %	-2.1695	0.73	0.79
GO:0060964	*regulation of gene silencing by miRNA*	0.039 %	-1.4508	0.71	0.69
GO:0060966	*regulation of gene silencing by RNA*	0.056 %	-1.3028	0.73	0.82
GO:0031324	*negative regulation of cellular metabolic process*	1.929 %	-2.0722	0.68	0.91
GO:1902679	*negative regulation of RNA biosynthetic process*	1.057 %	-2.2966	0.64	0.99
GO:0031327	*negative regulation of cellular biosynthetic process*	1.429 %	-2.4986	0.66	0.98
GO:0051253	*negative regulation of RNA metabolic process*	1.062 %	-2.2867	0.66	0.96
GO:0048585	*negative regulation of response to stimulus*	0.699 %	-1.1099	0.68	0.68
GO:0045934	*negative regulation of nucleobase-containing compound metabolic process*	1.139 %	-2.1512	0.67	0.87
GO:0045892	*negative regulation of transcription, DNA-templated*	0.958 %	-2.2966	0.64	0.94
GO:0010629	*negative regulation of gene expression*	1.601 %	-2.1974	0.69	0.88
GO:0010605	*negative regulation of macromolecule metabolic process*	2.171 %	-1.9628	0.68	0.94
GO:0060147	*regulation of posttranscriptional gene silencing*	0.060 %	-1.2448	0.73	0.84
GO:0010558	*negative regulation of macromolecule biosynthetic process*	1.355 %	-2.6232	0.66	0.96
GO:1903507	*negative regulation of nucleic acid-templated transcription*	1.057 %	-2.2966	0.64	0.98
GO:0009892	*negative regulation of metabolic process*	2.352 %	-1.7214	0.71	0.86
GO:0009890	*negative regulation of biosynthetic process*	1.450 %	-2.4742	0.68	0.85
GO:0051172	*negative regulation of nitrogen compound metabolic process*	1.433 %	-2.4742	0.70	0.86
GO:0006968	cellular defense response	0.022 %	-3.2582	0.88	0.45
GO:1902583	multi-organism intracellular transport	0.009 %	-1.4167	0.89	0.45
GO:0044766	*multi-organism transport*	0.052 %	-1.2037	0.90	0.89
GO:1902581	*multi-organism cellular localization*	0.009 %	-1.4167	0.90	0.81
GO:0030581	*symbiont intracellular protein transport in host*	0.004 %	-1.4167	0.89	0.96
GO:0010817	regulation of hormone levels	1.109 %	-2.1737	0.82	0.45
GO:0051235	*maintenance of location*	0.302 %	-1.5611	0.79	0.51
GO:0000023	maltose metabolic process	0.017 %	-2.7551	0.81	0.46
GO:0046351	*disaccharide biosynthetic process*	0.155 %	-2.6377	0.74	0.77
GO:0000024	*maltose biosynthetic process*	0.004 %	-2.9972	0.80	0.62
GO:0009312	*oligosaccharide biosynthetic process*	0.255 %	-2.3614	0.76	0.86

GO:0005986	*sucrose biosynthetic process*	*0.056 %*	*-1.1082*	*0.76*	*0.85*
GO:0005984	*disaccharide metabolic process*	*0.285 %*	*-2.0489*	*0.77*	*0.90*
GO:0046490	isopentenyl diphosphate metabolic process	0.091 %	-1.1846	0.85	0.48
GO:0015936	coenzyme A metabolic process	0.052 %	-1.1488	0.82	0.48
GO:0006950	response to stress	14.156 %	-4.7836	0.82	0.49
GO:0051716	cellular response to stimulus	12.637 %	-2.6195	0.78	0.50

Biological Process (90) Tag Clouds

Scatterplot & Table Interactive Graph TreeMap

GROUP 14

Hide/show dispensable GO terms

Export results to text table (CSV)

Make R script for plotting

term ID	description	frequency	pin?	log_{10} p-value	uniqueness	dispensability
GO:0006623	protein targeting to vacuole	0.155 %		-1.1711	0.94	0.00
GO:0072666	*establishment of protein localization to vacuole*	*0.155 %*		*-1.1711*	*0.94*	*1.00*
GO:0072665	*protein localization to vacuole*	*0.155 %*		*-1.1711*	*0.95*	*0.68*
GO:0009827	plant-type cell wall modification	0.095 %		-4.3776	0.84	0.00
GO:0042545	*cell wall modification*	*0.561 %*		*-2.3903*	*0.83*	*0.60*
GO:0071555	*cell wall organization*	*2.430 %*		*-1.7653*	*0.82*	*0.81*
GO:0071669	*plant-type cell wall organization or biogenesis*	*1.152 %*		*-1.7088*	*0.85*	*0.82*
GO:0009830	*cell wall modification involved in abscission*	*0.013 %*		*-2.7131*	*0.73*	*0.80*
GO:0009828	*plant-type cell wall loosening*	*0.022 %*		*-4.3729*	*0.85*	*0.67*
GO:0009664	*plant-type cell wall organization*	*0.652 %*		*-2.5353*	*0.82*	*0.74*
GO:0045488	pectin metabolic process	0.647 %		-5.0036	0.83	0.00
GO:1901575	*organic substance catabolic process*	*7.402 %*		*-1.4558*	*0.83*	*0.83*
GO:0016052	*carbohydrate catabolic process*	*1.187 %*		*-2.7805*	*0.80*	*0.62*
GO:0000272	*polysaccharide catabolic process*	*0.777 %*		*-3.8737*	*0.79*	*0.79*
GO:0005976	*polysaccharide metabolic process*	*1.899 %*		*-2.6607*	*0.83*	*0.70*
GO:0009057	*macromolecule catabolic process*	*5.050 %*		*-1.9358*	*0.83*	*0.65*
GO:0010393	*galacturonan metabolic process*	*0.652 %*		*-4.9664*	*0.84*	*0.77*
GO:0045490	*pectin catabolic process*	*0.423 %*		*-5.4439*	*0.79*	*0.95*
GO:0071456	cellular response to hypoxia	0.117 %		-2.5805	0.77	0.03

GO:0071453	cellular response to oxygen levels	0.129 %	-2.4974	0.79	0.87
GO:0036293	response to decreased oxygen levels	0.281 %	-1.8174	0.84	0.99
GO:0036294	cellular response to decreased oxygen levels	0.129 %	-2.4974	0.79	0.99
GO:0001666	response to hypoxia	0.250 %	-1.8819	0.82	0.92
GO:0071554	cell wall organization or biogenesis	3.168 %	-1.1166	0.93	0.04
GO:0051762	sesquiterpene biosynthetic process	0.009 %	-4.1162	0.72	0.06
GO:0046246	terpene biosynthetic process	0.043 %	-3.3476	0.69	0.86
GO:0051761	sesquiterpene metabolic process	0.009 %	-4.1162	0.73	0.78
GO:0008299	isoprenoid biosynthetic process	0.738 %	-1.4801	0.64	0.96
GO:0006720	isoprenoid metabolic process	0.850 %	-1.3509	0.69	0.71
GO:0006721	terpenoid metabolic process	0.699 %	-1.5038	0.67	0.94
GO:0006644	phospholipid metabolic process	1.014 %	-1.6136	0.69	0.60
GO:0045338	farnesyl diphosphate metabolic process	0.078 %	-3.4038	0.71	0.57
GO:0042214	terpene metabolic process	0.065 %	-2.9730	0.72	0.65
GO:0016114	terpenoid biosynthetic process	0.591 %	-1.6579	0.65	0.80
GO:0010047	fruit dehiscence	0.039 %	-1.3193	0.85	0.06
GO:0009900	dehiscence	0.095 %	-1.0627	0.85	0.69
GO:0009901	anther dehiscence	0.069 %	-1.2820	0.82	0.92
GO:0048235	pollen sperm cell differentiation	0.147 %	-1.0341	0.74	0.76
GO:0018958	phenol-containing compound metabolic process	0.190 %	-1.3666	0.86	0.06
GO:0009056	catabolic process	8.118 %	-1.8973	0.93	0.07
GO:0042744	hydrogen peroxide catabolic process	0.380 %	-1.2340	0.84	0.07
GO:0005975	carbohydrate metabolic process	4.670 %	-2.9103	0.91	0.10
GO:0006949	syncytium formation	0.043 %	-3.1541	0.81	0.13
GO:0007267	cell-cell signaling	0.371 %	-1.5333	0.80	0.17
GO:0019748	secondary metabolic process	1.938 %	-2.2449	0.78	0.21
GO:0090344	negative regulation of cell aging	0.004 %	-2.2368	0.74	0.21
GO:0090342	regulation of cell aging	0.017 %	-1.5524	0.76	0.90
GO:0007569	cell aging	0.022 %	-1.5427	0.79	0.56
GO:0009835	fruit ripening	0.043 %	-1.5063	0.82	0.64
GO:0009910	negative regulation of flower development	0.164 %	-1.0656	0.75	0.64
GO:0002215	defense response to nematode	0.013 %	-1.8312	0.88	0.21
GO:0009806	lignan metabolic process	0.022 %	-1.9879	0.70	0.22
GO:0010439	regulation of glucosinolate biosynthetic process	0.026 %	-1.5723	0.60	0.78
GO:1900376	regulation of secondary metabolite biosynthetic process	0.078 %	-1.1200	0.68	0.82
GO:0044550	secondary metabolite biosynthetic process	1.183 %	-1.0552	0.67	0.80
GO:1901599	(-)-pinoresinol biosynthetic process	0.009 %	-2.1912	0.67	0.59
GO:1901598	(-)-pinoresinol metabolic process	0.009 %	-2.1123	0.68	0.90
GO:0019760	glucosinolate metabolic process	0.514 %	-1.9727	0.64	0.56
GO:0019758	glycosinolate biosynthetic process	0.194 %	-1.0341	0.63	1.00
GO:0010675	regulation of cellular carbohydrate metabolic process	0.125 %	-1.1166	0.78	0.83
GO:0019757	glycosinolate metabolic process	0.514 %	-1.9727	0.64	0.99
GO:0019761	glucosinolate biosynthetic process	0.194 %	-1.0341	0.63	0.91
GO:0009698	phenylpropanoid metabolic process	0.505 %	-1.1727	0.66	0.73
GO:0042762	regulation of sulfur metabolic process	0.091 %	-1.2076	0.80	0.68
GO:0046189	phenol-containing compound biosynthetic process	0.104 %	-1.3666	0.83	0.77
GO:0009807	lignan biosynthetic process	0.022 %	-1.9879	0.67	0.95
GO:0046274	lignin catabolic process	0.078 %	-1.2119	0.64	0.67
GO:0046271	phenylpropanoid catabolic process	0.078 %	-1.1869	0.64	0.73
GO:0071695	anatomical structure maturation	0.043 %	-1.5063	0.86	0.24
GO:0007568	aging	0.544 %	-1.0947	0.85	0.29
GO:0009741	response to brassinosteroid	0.410 %	-1.3925	0.90	0.30
GO:0045229	external encapsulating structure organization	2.577 %	-1.5212	0.87	0.33
GO:0045824	negative regulation of innate immune response	0.060 %	-2.1499	0.76	0.36
GO:0043069	negative regulation of programmed cell death	0.082 %	-1.0717	0.74	0.84
GO:0050777	negative regulation of immune response	0.065 %	-2.1499	0.77	0.98
GO:0002683	negative regulation of immune system process	0.073 %	-2.0456	0.82	0.73
GO:0010204	defense response signaling pathway, resistance gene-independent	0.052 %	-1.3029	0.71	0.74
GO:0010363	regulation of plant-type hypersensitive response	0.060 %	-1.4198	0.66	0.74
GO:0080135	regulation of cellular response to stress	0.250 %	-1.0717	0.77	0.63
GO:0009814	defense response, incompatible interaction	0.725 %	-1.0590	0.81	0.69
GO:0034051	negative regulation of plant-type hypersensitive response	0.022 %	-2.1912	0.65	0.93
GO:0002237	response to molecule of bacterial origin	0.121 %	-1.1596	0.88	0.37
GO:0070482	response to oxygen levels	0.285 %	-1.8090	0.89	0.37
GO:0022411	cellular component disassembly	0.324 %	-1.1032	0.78	0.37
GO:0019722	calcium-mediated signaling	0.302 %	-1.2720	0.77	0.39
GO:0019932	second-messenger-mediated signaling	0.319 %	-1.0935	0.77	0.58
GO:0033554	cellular response to stress	3.772 %	-1.1737	0.81	0.40
GO:0002213	defense response to insect	0.069 %	-1.0453	0.87	0.41
GO:0045926	negative regulation of growth	0.121 %	-1.1339	0.88	0.45
GO:0044277	cell wall disassembly	0.013 %	-2.7131	0.77	0.46

Biological Process (174) Tag Clouds

Scatterplot & Table Interactive Graph TreeMap

GROUP 15

Tip: your resulting list of GO terms seems to be quite long. If you want to reduce it further, press the Back button in your browser and choose a different setting for the "allowed similarity" parameter.

Hide/show dispensable GO terms

Export results to text table (CSV)

Make R script for plotting

term ID	description	frequency	pin?	log_{10} p-value	uniqueness	dispensability
GO:0006457	protein folding	0.764 %		-2.4310	0.95	0.00
GO:0009416	response to light stimulus	2.771 %		-5.1925	0.80	0.00
GO:0009266	response to temperature stimulus	2.287 %		-1.4530	0.84	0.63
GO:0010224	response to UV-B	0.298 %		-2.1639	0.83	0.83
GO:0009582	detection of abiotic stimulus	0.311 %		-1.2335	0.84	0.94
GO:0009581	detection of external stimulus	0.311 %		-1.2335	0.88	0.93
GO:0009584	detection of visible light	0.022 %		-1.9515	0.84	0.57
GO:0009583	detection of light stimulus	0.268 %		-1.4435	0.81	0.80
GO:0009314	response to radiation	2.892 %		-5.1350	0.83	0.65
GO:0071214	cellular response to abiotic stimulus	0.889 %		-1.3778	0.82	0.56
GO:0009642	response to light intensity	0.587 %		-3.4507	0.83	0.79
GO:0009644	response to high light intensity	0.324 %		-4.1064	0.84	0.74
GO:0009638	phototropism	0.069 %		-1.4160	0.84	0.63
GO:0009650	UV protection	0.013 %		-4.8195	0.86	0.55
GO:0034605	cellular response to heat	0.160 %		-1.1881	0.83	0.66
GO:0006970	response to osmotic stress	2.512 %		-1.9923	0.83	0.64

GO:0034644	cellular response to UV	0.073 %	-3.5387	0.81	0.71
GO:0009411	response to UV	0.427 %	-1.7064	0.83	0.76
GO:0009408	response to heat	0.859 %	-2.9242	0.84	0.56
GO:0071482	cellular response to light stimulus	0.561 %	-1.8451	0.79	0.83
GO:0070370	cellular heat acclimation	0.039 %	-1.4550	0.84	0.59
GO:0071478	cellular response to radiation	0.583 %	-1.8451	0.79	0.91
GO:0071493	cellular response to UV-B	0.030 %	-3.9192	0.82	0.64
GO:0080167	response to karrikin	0.531 %	-1.1792	0.86	0.53
GO:0009987	cellular process	58.826 %	-1.0780	1.00	0.00
GO:0010187	negative regulation of seed germination	0.073 %	-4.6621	0.78	0.00
GO:2000026	regulation of multicellular organismal development	1.765 %	-1.2800	0.77	0.92
GO:0051241	negative regulation of multicellular organismal process	0.445 %	-2.8466	0.77	0.61
GO:0048581	negative regulation of post-embryonic development	0.354 %	-3.1135	0.76	0.84
GO:0048580	regulation of post-embryonic development	1.303 %	-1.5150	0.77	0.79
GO:1900140	regulation of seedling development	0.315 %	-2.8575	0.79	0.92
GO:0009845	seed germination	0.600 %	-3.1861	0.86	0.81
GO:0051093	negative regulation of developmental process	0.492 %	-2.6869	0.81	0.57
GO:0010029	regulation of seed germination	0.298 %	-2.9546	0.79	0.91
GO:0044699	single-organism process	41.709 %	-1.0977	0.99	0.00
GO:0050896	response to stimulus	25.943 %	-2.1898	0.99	0.00
GO:0043335	protein unfolding	0.004 %	-1.9908	0.97	0.03
GO:0001887	selenium compound metabolic process	0.017 %	-1.9515	0.94	0.03
GO:0061077	chaperone-mediated protein folding	0.224 %	-1.3852	0.96	0.04
GO:0009658	chloroplast organization	0.803 %	-1.5908	0.92	0.05
GO:0019750	chloroplast localization	0.134 %	-1.3952	0.82	1.00
GO:0009903	chloroplast avoidance movement	0.112 %	-1.6346	0.79	0.97
GO:0009904	chloroplast accumulation movement	0.095 %	-1.7583	0.79	0.77
GO:0009902	chloroplast relocation	0.129 %	-1.3952	0.78	0.99
GO:0051656	establishment of organelle localization	0.276 %	-1.2656	0.82	0.91
GO:0009657	plastid organization	1.032 %	-1.2784	0.93	0.55
GO:0051667	establishment of plastid localization	0.129 %	-1.3952	0.82	0.99
GO:0051644	plastid localization	0.134 %	-1.3952	0.83	0.84
GO:0018298	protein-chromophore linkage	0.203 %	-1.8090	0.90	0.06
GO:0046209	nitric oxide metabolic process	0.030 %	-1.6436	0.90	0.06
GO:1903409	reactive oxygen species biosynthetic process	0.099 %	-1.4213	0.91	0.65
GO:0006809	nitric oxide biosynthetic process	0.022 %	-1.7583	0.89	0.75
GO:0042126	nitrate metabolic process	0.181 %	-1.5840	0.79	0.98
GO:0042128	nitrate assimilation	0.181 %	-1.5840	0.79	0.86
GO:0046462	monoacylglycerol metabolic process	0.004 %	-4.5604	0.82	0.06
GO:0046340	diacylglycerol catabolic process	0.004 %	-4.5604	0.80	0.91
GO:0046339	diacylglycerol metabolic process	0.022 %	-4.3614	0.80	0.70
GO:0044242	cellular lipid catabolic process	0.315 %	-2.1379	0.76	0.66
GO:0006639	acylglycerol metabolic process	0.147 %	-3.1062	0.78	0.87
GO:0046464	acylglycerol catabolic process	0.022 %	-4.1637	0.78	0.77
GO:0046461	neutral lipid catabolic process	0.022 %	-4.1637	0.78	0.77
GO:0046503	glycerolipid catabolic process	0.035 %	-4.1637	0.79	0.54
GO:0052651	monoacylglycerol catabolic process	0.004 %	-4.5604	0.80	0.91
GO:0051186	cofactor metabolic process	1.735 %	-1.6475	0.92	0.08
GO:0044710	single-organism metabolic process	18.390 %	-1.7683	0.87	0.11
GO:0009056	catabolic process	8.118 %	-1.2284	0.94	0.12
GO:0019915	lipid storage	0.112 %	-3.8189	0.82	0.13
GO:0010876	lipid localization	0.708 %	-2.5647	0.85	0.50
GO:0071941	nitrogen cycle metabolic process	0.207 %	-1.4323	0.94	0.13
GO:0090332	stomatal closure	0.121 %	-2.3482	0.87	0.13
GO:0090333	regulation of stomatal closure	0.065 %	-1.3803	0.81	0.77
GO:0010119	regulation of stomatal movement	0.281 %	-1.6020	0.79	0.86
GO:0019354	siroheme biosynthetic process	0.009 %	-2.5334	0.83	0.14
GO:0006783	heme biosynthetic process	0.104 %	-1.6257	0.80	0.83
GO:0046156	siroheme metabolic process	0.009 %	-2.5334	0.83	0.72
GO:0006732	coenzyme metabolic process	1.191 %	-1.2331	0.89	0.71
GO:0042168	heme metabolic process	0.121 %	-1.5684	0.81	0.69
GO:0043412	macromolecule modification	15.900 %	-1.2090	0.91	0.15
GO:2001057	reactive nitrogen species metabolic process	0.203 %	-1.4054	0.94	0.15
GO:0009610	response to symbiotic fungus	0.052 %	-1.1820	0.91	0.18
GO:0048519	negative regulation of biological process	3.716 %	-1.3501	0.88	0.20
GO:0044237	cellular metabolic process	46.461 %	-1.0768	0.91	0.21
GO:0032787	monocarboxylic acid metabolic process	2.128 %	-2.2507	0.77	0.21
GO:0006559	L-phenylalanine catabolic process	0.035 %	-1.9908	0.78	0.50
GO:0006558	L-phenylalanine metabolic process	0.073 %	-1.5684	0.80	0.95
GO:0043436	oxoacid metabolic process	5.002 %	-2.4827	0.75	0.93
GO:0019752	carboxylic acid metabolic process	4.437 %	-1.8979	0.75	0.82

GO:0006082	organic acid metabolic process	5.015 %	-2.2509	0.76	0.71
GO:0009063	cellular amino acid catabolic process	0.276 %	-1.0693	0.75	0.76
GO:0009694	jasmonic acid metabolic process	0.117 %	-1.1027	0.81	0.56
GO:0016485	protein processing	0.220 %	-1.2807	0.92	0.22
GO:0044092	negative regulation of molecular function	1.070 %	-1.0905	0.86	0.23
GO:0051604	protein maturation	0.272 %	-1.2200	0.92	0.23
GO:0009611	response to wounding	0.816 %	-2.8963	0.90	0.24
GO:0010118	stomatal movement	0.440 %	-2.1555	0.87	0.24
GO:0010155	regulation of proton transport	0.030 %	-2.1363	0.69	0.25
GO:0006813	potassium ion transport	0.496 %	-1.0261	0.80	0.74
GO:0006812	cation transport	2.542 %	-1.1021	0.78	0.83
GO:0071805	potassium ion transmembrane transport	0.319 %	-1.2006	0.69	0.81
GO:0071804	cellular potassium ion transport	0.319 %	-1.2006	0.70	0.92
GO:0034762	regulation of transmembrane transport	0.216 %	-1.1052	0.72	0.81
GO:0034763	negative regulation of transmembrane transport	0.017 %	-1.7465	0.71	0.92
GO:0010360	negative regulation of anion channel activity	0.017 %	-1.7465	0.69	0.96
GO:0010359	regulation of anion channel activity	0.047 %	-1.1881	0.70	0.87
GO:0034765	regulation of ion transmembrane transport	0.207 %	-1.1052	0.69	0.92
GO:0010362	negative regulation of anion channel activity by blue light	0.009 %	-2.1084	0.70	0.66
GO:0010361	regulation of anion channel activity by blue light	0.009 %	-2.1084	0.72	0.82
GO:0034766	negative regulation of ion transmembrane transport	0.017 %	-1.7465	0.70	1.00
GO:0098656	anion transmembrane transport	0.902 %	-1.0739	0.78	0.67
GO:1903960	negative regulation of anion transmembrane transport	0.017 %	-1.7465	0.70	1.00
GO:1903959	regulation of anion transmembrane transport	0.129 %	-1.1881	0.69	0.95
GO:0010107	potassium ion import	0.022 %	-1.4729	0.74	0.52
GO:0022898	regulation of transmembrane transporter activity	0.060 %	-1.1179	0.71	1.00
GO:0051051	negative regulation of transport	0.026 %	-1.5046	0.75	0.62
GO:0032412	regulation of ion transmembrane transporter activity	0.060 %	-1.1179	0.70	0.98
GO:0032409	regulation of transporter activity	0.060 %	-1.1179	0.74	0.65
GO:0032410	negative regulation of transporter activity	0.017 %	-1.7465	0.71	0.95
GO:0032413	negative regulation of ion transmembrane transporter activity	0.017 %	-1.7465	0.69	1.00
GO:0044070	regulation of anion transport	0.164 %	-1.1851	0.70	0.80
GO:1903792	negative regulation of anion transport	0.017 %	-1.7465	0.70	0.95
GO:0015672	monovalent inorganic cation transport	1.260 %	-1.6181	0.79	0.57
GO:0034220	ion transmembrane transport	2.529 %	-1.5260	0.78	0.76
GO:0043271	negative regulation of ion transport	0.026 %	-1.5046	0.70	0.98
GO:0043269	regulation of ion transport	0.302 %	-1.0003	0.70	0.85
GO:0010493	Lewis a epitope biosynthetic process	0.009 %	-2.1363	0.85	0.26
GO:0043413	macromolecule glycosylation	0.483 %	-1.0022	0.82	0.97
GO:0009100	glycoprotein metabolic process	0.496 %	-1.0022	0.90	0.62
GO:0009101	glycoprotein biosynthetic process	0.488 %	-1.0022	0.89	0.99
GO:0006486	protein glycosylation	0.483 %	-1.0022	0.78	0.54
GO:0065008	regulation of biological quality	5.991 %	-1.2867	0.89	0.29
GO:0051640	organelle localization	0.332 %	-1.1614	0.84	0.29
GO:0000413	protein peptidyl-prolyl isomerization	0.250 %	-1.6002	0.90	0.30
GO:0018208	peptidyl-proline modification	0.341 %	-1.4213	0.89	0.67
GO:0048480	stigma development	0.004 %	-1.8823	0.91	0.31
GO:0042542	response to hydrogen peroxide	0.268 %	-2.8548	0.88	0.31
GO:0000302	response to reactive oxygen species	0.678 %	-1.9418	0.88	0.71
GO:0046686	response to cadmium ion	1.368 %	-1.6748	0.89	0.62
GO:0010038	response to metal ion	1.938 %	-1.3501	0.88	0.77
GO:0044763	single-organism cellular process	23.034 %	-1.4367	0.87	0.32
GO:0045727	positive regulation of translation	0.095 %	-1.5248	0.79	0.32
GO:0032270	positive regulation of cellular protein metabolic process	0.699 %	-1.1363	0.80	0.82
GO:0051247	positive regulation of protein metabolic process	0.721 %	-1.0281	0.82	0.63
GO:0034250	positive regulation of cellular amide metabolic process	0.095 %	-1.5248	0.84	0.69
GO:0009628	response to abiotic stimulus	7.946 %	-4.3374	0.89	0.33
GO:0051239	regulation of multicellular organismal process	1.972 %	-1.1398	0.85	0.35
GO:0009791	post-embryonic development	5.848 %	-1.7920	0.85	0.57
GO:0007275	multicellular organism development	10.423 %	-1.1618	0.84	0.78
GO:0044712	single-organism catabolic process	2.676 %	-2.1886	0.80	0.37
GO:0044248	cellular catabolic process	5.939 %	-1.1362	0.86	0.85
GO:1901575	organic substance catabolic process	7.402 %	-1.3183	0.87	0.74
GO:0055074	calcium ion homeostasis	0.060 %	-1.2731	0.91	0.37
GO:0072507	divalent inorganic cation homeostasis	0.147 %	-1.0648	0.90	0.64
GO:0006979	response to oxidative stress	1.908 %	-1.9532	0.89	0.38
GO:0002213	defense response to insect	0.069 %	-1.1614	0.90	0.38
GO:0009608	response to symbiont	0.060 %	-1.1102	0.91	0.39
GO:0010035	response to inorganic substance	3.699 %	-2.1838	0.88	0.40
GO:2000022	regulation of jasmonic acid mediated signaling pathway	0.108 %	-1.6919	0.77	0.40
GO:2000031	regulation of salicylic acid mediated signaling pathway	0.086 %	-1.5046	0.78	0.64
GO:0006638	neutral lipid metabolic process	0.147 %	-3.1062	0.80	0.40

GO:0072330	*monocarboxylic acid biosynthetic process*	*1.101 %*	*-1.2970*	*0.76*	*0.84*
GO:0016042	*lipid catabolic process*	*1.032 %*	*-1.7326*	*0.77*	*0.73*
GO:0044255	*cellular lipid metabolic process*	*3.224 %*	*-1.9988*	*0.76*	*0.59*
GO:0006631	*fatty acid metabolic process*	*1.101 %*	*-1.4017*	*0.73*	*0.79*
GO:0006633	*fatty acid biosynthetic process*	*0.682 %*	*-1.7046*	*0.73*	*0.69*
GO:0046486	*glycerolipid metabolic process*	*0.764 %*	*-1.9872*	*0.78*	*0.70*
GO:0006629	lipid metabolic process	4.683 %	-1.5523	0.81	0.41
GO:0051235	maintenance of location	0.302 %	-2.4975	0.81	0.42
GO:0042391	regulation of membrane potential	0.138 %	-2.2329	0.91	0.43
GO:0090351	seedling development	0.660 %	-3.0757	0.87	0.44
GO:0006950	response to stress	14.156 %	-1.5788	0.88	0.45
GO:0006464	cellular protein modification process	12.719 %	-1.3858	0.86	0.45
GO:0036211	*protein modification process*	*12.719 %*	*-1.3858*	*0.88*	*0.75*
GO:0046500	S-adenosylmethionine metabolic process	0.043 %	-1.7023	0.91	0.47
GO:0044281	small molecule metabolic process	7.303 %	-1.5099	0.81	0.49

Biological Process (138) Tag Clouds

Scatterplot & Table Interactive Graph TreeMap

GROUP 16

Hide/show dispensable GO terms

Export results to text table (CSV)

Make R script for plotting

term ID	description	frequency	pin?	log_{10} p-value	uniqueness	dispensability
GO:0007154	cell communication	9.698 %		-1.9190	0.91	0.00
GO:0009753	response to jasmonic acid	0.919 %		-2.1846	0.84	0.00
GO:0071229	*cellular response to acid chemical*	*2.158 %*		*-1.3400*	*0.76*	*0.79*
GO:0044700	*single organism signaling*	*8.899 %*		*-1.1863*	*0.89*	*0.91*
GO:0009755	*hormone-mediated signaling pathway*	*3.617 %*		*-1.5732*	*0.53*	*0.94*
GO:0009751	*response to salicylic acid*	*0.876 %*		*-1.5242*	*0.84*	*0.60*
GO:0009819	*drought recovery*	*0.047 %*		*-1.5603*	*0.86*	*0.70*
GO:0032870	*cellular response to hormone stimulus*	*3.871 %*		*-1.8453*	*0.75*	*0.63*
GO:0070887	*cellular response to chemical stimulus*	*5.287 %*		*-1.1902*	*0.77*	*0.67*
GO:0071396	*cellular response to lipid*	*1.770 %*		*-1.0695*	*0.77*	*0.81*
GO:0009414	*response to water deprivation*	*1.390 %*		*-2.0704*	*0.82*	*0.63*
GO:0009415	*response to water*	*1.416 %*		*-2.0365*	*0.83*	*0.74*
GO:0071310	*cellular response to organic substance*	*4.601 %*		*-1.4723*	*0.75*	*0.88*
GO:0007165	*signal transduction*	*8.731 %*		*-1.2023*	*0.53*	*0.64*
GO:0071495	*cellular response to endogenous stimulus*	*3.988 %*		*-1.7956*	*0.87*	*0.75*
GO:0010150	leaf senescence	0.462 %		-1.9838	0.88	0.00
GO:0090693	*plant organ senescence*	*0.462 %*		*-1.9817*	*0.88*	*0.96*
GO:0048467	*gynoecium development*	*0.315 %*		*-1.1866*	*0.89*	*0.58*
GO:0010604	positive regulation of macromolecule metabolic process	2.672 %		-2.0767	0.53	0.00

GO:0048522	positive regulation of cellular process	3.582 %	-1.3241	0.60	0.87
GO:0032774	RNA biosynthetic process	11.813 %	-2.9573	0.62	0.82
GO:0080090	regulation of primary metabolic process	13.681 %	-1.8928	0.55	0.82
GO:0010468	regulation of gene expression	12.706 %	-2.2795	0.53	0.79
GO:0031323	regulation of cellular metabolic process	13.906 %	-2.1474	0.54	0.80
GO:1902680	positive regulation of RNA biosynthetic process	1.869 %	-1.6321	0.47	0.97
GO:0031328	positive regulation of cellular biosynthetic process	2.102 %	-1.4619	0.50	0.97
GO:0031326	regulation of cellular biosynthetic process	12.339 %	-2.2108	0.49	0.87
GO:0031325	positive regulation of cellular metabolic process	2.741 %	-2.0347	0.53	0.95
GO:0051252	regulation of RNA metabolic process	11.463 %	-2.9725	0.47	0.87
GO:2001141	regulation of RNA biosynthetic process	11.282 %	-3.0645	0.45	0.88
GO:0019219	regulation of nucleobase-containing compound metabolic process	11.700 %	-2.8458	0.49	0.84
GO:0051254	positive regulation of RNA metabolic process	1.912 %	-1.6321	0.49	0.96
GO:0016070	RNA metabolic process	17.562 %	-2.3055	0.66	0.66
GO:2000112	regulation of cellular macromolecule biosynthetic process	12.003 %	-2.5637	0.47	0.86
GO:0045935	positive regulation of nucleobase-containing compound metabolic process	2.007 %	-1.5823	0.50	0.96
GO:0010628	positive regulation of gene expression	2.050 %	-1.5432	0.54	0.92
GO:0006355	regulation of transcription, DNA-templated	11.234 %	-3.0645	0.45	0.58
GO:0006351	transcription, DNA-templated	11.709 %	-2.9573	0.61	0.87
GO:0045893	positive regulation of transcription, DNA-templated	1.864 %	-1.6321	0.47	0.90
GO:0060255	regulation of macromolecule metabolic process	14.091 %	-1.8747	0.53	0.83
GO:1903508	positive regulation of nucleic acid-templated transcription	1.869 %	-1.6321	0.47	0.98
GO:1903506	regulation of nucleic acid-templated transcription	11.282 %	-3.0645	0.45	0.89
GO:0097659	nucleic acid-templated transcription	11.761 %	-2.9573	0.62	0.88
GO:0009893	positive regulation of metabolic process	2.892 %	-1.9007	0.57	0.83
GO:0009891	positive regulation of biosynthetic process	2.145 %	-1.4457	0.53	0.92
GO:0009889	regulation of biosynthetic process	12.408 %	-2.1401	0.53	0.78
GO:0010556	regulation of macromolecule biosynthetic process	12.076 %	-2.5529	0.49	0.86
GO:0010557	positive regulation of macromolecule biosynthetic process	2.007 %	-1.5188	0.50	0.95
GO:0051173	positive regulation of nitrogen compound metabolic process	2.119 %	-1.4758	0.54	0.92
GO:0034654	nucleobase-containing compound biosynthetic process	13.056 %	-2.4593	0.66	0.80
GO:0051171	regulation of nitrogen compound metabolic process	12.430 %	-2.3484	0.54	0.76
GO:0034645	cellular macromolecule biosynthetic process	16.737 %	-1.4801	0.66	0.74
GO:0023052	signaling	8.908 %	-1.1839	0.99	0.00
GO:0007062	sister chromatid cohesion	0.186 %	-1.5127	0.82	0.09
GO:0098813	nuclear chromosome segregation	0.436 %	-1.0206	0.87	0.92
GO:0000819	sister chromatid segregation	0.285 %	-1.2295	0.81	0.89
GO:0006869	lipid transport	0.596 %	-1.0240	0.94	0.10
GO:0018027	peptidyl-lysine dimethylation	0.022 %	-1.7099	0.72	0.10
GO:0034968	histone lysine methylation	0.298 %	-1.1074	0.62	0.79
GO:0018022	peptidyl-lysine methylation	0.350 %	-1.0991	0.67	0.93
GO:0018874	benzoate metabolic process	0.009 %	-1.5016	0.84	0.14
GO:0046482	para-aminobenzoic acid metabolic process	0.017 %	-1.3325	0.83	0.70
GO:0000959	mitochondrial RNA metabolic process	0.324 %	-1.2908	0.79	0.15
GO:0032196	transposition	0.013 %	-1.3708	0.91	0.15
GO:0031930	mitochondria-nucleus signaling pathway	0.043 %	-1.8337	0.71	0.19
GO:0071474	cellular hyperosmotic response	0.013 %	-1.5127	0.86	0.22
GO:0071475	cellular hyperosmotic salinity response	0.009 %	-1.5127	0.86	0.67
GO:0010467	gene expression	18.304 %	-1.2202	0.80	0.23
GO:0009611	response to wounding	0.816 %	-1.3302	0.90	0.24
GO:0016556	mRNA modification	0.311 %	-1.2295	0.73	0.25
GO:1900864	mitochondrial RNA modification	0.263 %	-1.3399	0.73	0.95
GO:0080156	mitochondrial mRNA modification	0.233 %	-1.3474	0.73	0.64
GO:0061647	histone H3-K9 modification	0.069 %	-1.2843	0.70	0.26
GO:1902275	regulation of chromatin organization	0.233 %	-1.1032	0.66	0.60
GO:0009628	response to abiotic stimulus	7.946 %	-1.0389	0.89	0.28
GO:0042549	photosystem II stabilization	0.030 %	-1.7883	0.74	0.28
GO:0043543	protein acylation	0.345 %	-1.0869	0.78	0.28
GO:0019438	aromatic compound biosynthetic process	14.247 %	-2.2909	0.70	0.29
GO:0018130	heterocycle biosynthetic process	14.014 %	-2.0025	0.70	0.58
GO:0009059	macromolecule biosynthetic process	17.035 %	-1.4270	0.72	0.62
GO:0044271	cellular nitrogen compound biosynthetic process	17.018 %	-1.4568	0.69	0.61
GO:1901362	organic cyclic compound biosynthetic process	14.769 %	-2.1035	0.72	0.55
GO:0009647	skotomorphogenesis	0.026 %	-1.5016	0.84	0.30
GO:0009646	response to absence of light	0.142 %	-1.0950	0.90	0.69
GO:0046685	response to arsenic-containing substance	0.060 %	-1.1762	0.89	0.30
GO:1901983	regulation of protein acetylation	0.056 %	-1.5863	0.57	0.30
GO:1901984	negative regulation of protein acetylation	0.039 %	-1.6436	0.54	0.95
GO:0031060	regulation of histone methylation	0.086 %	-1.5127	0.51	0.92
GO:0031057	negative regulation of histone modification	0.047 %	-1.6436	0.51	0.95
GO:0031058	positive regulation of histone modification	0.069 %	-1.6285	0.48	0.87

GO:0031056	regulation of histone modification	0.134 %	-1.3399	0.52	0.88
GO:2000756	regulation of peptidyl-lysine acetylation	0.056 %	-1.5863	0.53	1.00
GO:0031062	positive regulation of histone methylation	0.047 %	-1.6285	0.48	0.94
GO:0051570	regulation of histone H3-K9 methylation	0.039 %	-1.6140	0.51	0.84
GO:0051567	histone H3-K9 methylation	0.065 %	-1.2843	0.64	0.96
GO:1905269	positive regulation of chromatin organization	0.086 %	-1.5479	0.58	0.81
GO:0016573	histone acetylation	0.263 %	-1.1426	0.60	0.86
GO:1900111	positive regulation of histone H3-K9 dimethylation	0.017 %	-1.7099	0.49	0.89
GO:1900109	regulation of histone H3-K9 dimethylation	0.022 %	-1.7099	0.53	0.99
GO:1905268	negative regulation of chromatin organization	0.065 %	-1.4130	0.61	0.84
GO:0051574	positive regulation of histone H3-K9 methylation	0.022 %	-1.6592	0.49	0.92
GO:0035065	regulation of histone acetylation	0.056 %	-1.5863	0.49	0.98
GO:0035067	negative regulation of histone acetylation	0.039 %	-1.6436	0.47	0.75
GO:0006473	protein acetylation	0.319 %	-1.1246	0.70	0.97
GO:0006475	internal protein amino acid acetylation	0.281 %	-1.1426	0.70	0.97
GO:0036123	histone H3-K9 dimethylation	0.022 %	-1.7099	0.66	1.00
GO:2000757	negative regulation of peptidyl-lysine acetylation	0.039 %	-1.6436	0.52	1.00
GO:0031400	negative regulation of protein modification process	0.220 %	-1.2590	0.53	0.72
GO:0018394	peptidyl-lysine acetylation	0.263 %	-1.1426	0.65	0.97
GO:0018393	internal peptidyl-lysine acetylation	0.263 %	-1.1426	0.65	1.00
GO:0031401	positive regulation of protein modification process	0.337 %	-1.1915	0.49	0.83
GO:0009962	regulation of flavonoid biosynthetic process	0.095 %	-1.0524	0.70	0.31
GO:0048504	regulation of timing of animal organ formation	0.004 %	-1.8104	0.74	0.32
GO:2000027	regulation of organ morphogenesis	0.004 %	-1.3956	0.75	1.15
GO:0010223	secondary shoot formation	0.112 %	-1.1032	0.86	0.58
GO:0010160	formation of animal organ boundary	0.055 %	-1.4042	0.83	1.15
GO:0048859	formation of anatomical boundary	0.069 %	-1.0008	0.88	0.69
GO:0003156	regulation of animal organ formation	0.004 %	-1.5016	0.74	1.00
GO:0048645	animal organ formation	0.004 %	-1.1472	0.86	1.00
GO:0010346	shoot axis formation	0.112 %	-1.1032	0.86	0.80
GO:0010072	primary shoot apical meristem specification	0.060 %	-1.0991	0.88	0.69
GO:0009968	negative regulation of signal transduction	0.332 %	-1.6270	0.61	0.33
GO:0048523	negative regulation of cellular process	2.719 %	-1.1860	0.65	0.68
GO:0023057	negative regulation of signaling	0.345 %	-1.6132	0.70	0.78
GO:0010648	negative regulation of cell communication	0.345 %	-1.6132	0.69	0.78
GO:0009937	regulation of gibberellic acid mediated signaling pathway	0.073 %	-1.0750	0.64	0.69
GO:0009938	negative regulation of gibberellic acid mediated signaling pathway	0.039 %	-1.5016	0.62	0.83
GO:0080024	indolebutyric acid metabolic process	0.022 %	-1.3789	0.77	0.33
GO:0007568	aging	0.544 %	-1.6581	0.90	0.35
GO:0001763	morphogenesis of a branching structure	0.125 %	-1.0673	0.89	0.39
GO:0019222	regulation of metabolic process	14.847 %	-1.7676	0.66	0.39
GO:0050794	regulation of cellular process	22.244 %	-1.0096	0.63	0.63
GO:0070301	cellular response to hydrogen peroxide	0.035 %	-1.3112	0.82	0.40
GO:0010035	response to inorganic substance	3.699 %	-1.2632	0.85	0.46
GO:0001101	response to acid chemical	5.011 %	-1.8141	0.84	0.58
GO:0010529	negative regulation of transposition	0.009 %	-1.6140	0.74	0.47
GO:0010528	regulation of transposition	0.009 %	-1.6140	0.79	0.96
GO:0051782	negative regulation of cell division	0.022 %	-1.5016	0.74	0.50

Biological Process (145) Tag Clouds

Scatterplot & Table Interactive Graph TreeMap

GROUP 17

Hide/show dispensable GO terms

Export results to text table (CSV)

Make R script for plotting

term ID	description	frequency	pin?	$\log_{10}$ p-value	uniqueness	dispensability
GO:0002376	immune system process	1.532 %		-1.0569	0.99	0.00
GO:0015979	photosynthesis	1.057 %		-1.9769	0.92	0.00
GO:0042754	negative regulation of circadian rhythm	0.013 %		-3.4274	0.85	0.00
GO:0007623	*circadian rhythm*	*0.539 %*		*-1.3675*	*0.96*	*0.90*
GO:0042752	*regulation of circadian rhythm*	*0.220 %*		*-1.5315*	*0.82*	*0.69*
GO:0048511	rhythmic process	0.600 %		-1.3535	0.99	0.00
GO:0048574	long-day photoperiodism, flowering	0.121 %		-3.8603	0.75	0.00
GO:0061416	*regulation of transcription from RNA polymerase II promoter in response to salt stress*	*0.004 %*		*-1.6708*	*0.68*	*0.55*
GO:0061392	*regulation of transcription from RNA polymerase II promoter in response to osmotic stress*	*0.004 %*		*-1.6708*	*0.68*	*0.93*
GO:0048573	*photoperiodism, flowering*	*0.388 %*		*-2.0301*	*0.73*	*0.90*
GO:0048571	*long-day photoperiodism*	*0.138 %*		*-3.5949*	*0.83*	*0.82*
GO:0009785	*blue light signaling pathway*	*0.078 %*		*-2.3512*	*0.67*	*0.81*
GO:0043618	*regulation of transcription from RNA polymerase II promoter in response to stress*	*0.009 %*		*-1.3634*	*0.71*	*0.92*
GO:0009637	*response to blue light*	*0.341 %*		*-1.0749*	*0.83*	*0.74*
GO:0009638	*phototropism*	*0.069 %*		*-1.0612*	*0.82*	*0.80*
GO:0009648	*photoperiodism*	*0.427 %*		*-1.8234*	*0.82*	*0.76*
GO:0009416	*response to light stimulus*	*2.771 %*		*-2.8033*	*0.79*	*0.67*
GO:0071482	*cellular response to light stimulus*	*0.561 %*		*-1.1724*	*0.77*	*0.85*
GO:0071483	*cellular response to blue light*	*0.104 %*		*-2.1386*	*0.79*	*0.51*

GO:0071478	cellular response to radiation	0.583 %	-1.1724	0.77	0.91
GO:0065007	biological regulation	28.554 %	-1.0542	0.99	0.00
GO:0010398	xylogalacturonan metabolic process	0.004 %	-1.6941	0.93	0.03
GO:0009813	flavonoid biosynthetic process	0.332 %	-1.3014	0.89	0.05
GO:0009812	flavonoid metabolic process	0.414 %	-1.0611	0.94	0.06
GO:1901678	iron coordination entity transport	0.060 %	-3.7176	0.88	0.07
GO:0009767	photosynthetic electron transport chain	0.224 %	-2.5770	0.84	0.08
GO:0022900	electron transport chain	0.479 %	-2.1204	0.83	0.73
GO:0019684	photosynthesis, light reaction	0.505 %	-1.7692	0.90	0.79
GO:0080164	regulation of nitric oxide metabolic process	0.009 %	-1.9063	0.80	0.12
GO:0046209	nitric oxide metabolic process	0.030 %	-1.2843	0.90	0.71
GO:2001057	reactive nitrogen species metabolic process	0.203 %	-1.0509	0.91	0.12
GO:0030522	intracellular receptor signaling pathway	0.078 %	-2.3512	0.74	0.14
GO:0042547	cell wall modification involved in multidimensional cell growth	0.017 %	-1.2843	0.89	0.15
GO:0016049	cell growth	1.519 %	-1.1463	0.86	0.56
GO:0048878	chemical homeostasis	1.765 %	-2.2441	0.68	0.16
GO:0055082	cellular chemical homeostasis	0.902 %	-1.8058	0.62	0.86
GO:0055080	cation homeostasis	1.083 %	-1.6820	0.67	0.95
GO:0055076	transition metal ion homeostasis	0.678 %	-1.4245	0.68	0.91
GO:0055075	potassium ion homeostasis	0.069 %	-1.1228	0.73	0.73
GO:0055072	iron ion homeostasis	0.237 %	-2.0403	0.71	0.70
GO:0055065	metal ion homeostasis	0.842 %	-1.7573	0.68	0.93
GO:0055062	phosphate ion homeostasis	0.078 %	-1.1168	0.72	0.92
GO:0042592	homeostatic process	2.637 %	-1.5704	0.71	0.69
GO:0019725	cellular homeostasis	1.549 %	-1.1205	0.62	0.86
GO:0006884	cell volume homeostasis	0.009 %	-1.3526	0.73	0.53
GO:0006873	cellular ion homeostasis	0.790 %	-1.2250	0.62	0.92
GO:0050801	ion homeostasis	1.299 %	-2.1755	0.68	0.90
GO:0072506	trivalent inorganic anion homeostasis	0.078 %	-1.1168	0.72	0.81
GO:0072502	cellular trivalent inorganic anion homeostasis	0.022 %	-1.7448	0.69	0.96
GO:0030002	cellular anion homeostasis	0.030 %	-1.7448	0.68	0.58
GO:0030004	cellular monovalent inorganic cation homeostasis	0.160 %	-1.1109	0.65	0.83
GO:0009992	cellular water homeostasis	0.004 %	-1.3744	0.73	0.71
GO:0030643	cellular phosphate ion homeostasis	0.022 %	-1.7448	0.69	0.75
GO:0030007	cellular potassium ion homeostasis	0.022 %	-1.2581	0.69	0.57
GO:0098771	inorganic ion homeostasis	1.139 %	-2.3619	0.67	0.83
GO:0016139	glycoside catabolic process	0.009 %	-1.2843	0.89	0.18
GO:1901698	response to nitrogen compound	1.174 %	-1.7865	0.87	0.18
GO:0019509	L-methionine biosynthetic process from methylthioadenosine	0.039 %	-1.6941	0.78	0.20
GO:0071265	L-methionine biosynthetic process	0.052 %	-1.4896	0.78	0.88
GO:0071267	L-methionine salvage	0.043 %	-1.6941	0.78	0.99
GO:0009086	methionine biosynthetic process	0.112 %	-1.0717	0.77	0.92
GO:0043102	amino acid salvage	0.043 %	-1.6941	0.79	0.70
GO:0043620	regulation of DNA-templated transcription in response to stress	0.013 %	-1.2754	0.72	0.22
GO:0010266	response to vitamin B1	0.009 %	-1.8687	0.87	0.26
GO:0009753	response to jasmonic acid	0.919 %	-1.0730	0.86	0.60
GO:0009751	response to salicylic acid	0.876 %	-1.1747	0.86	0.58
GO:0033273	response to vitamin	0.026 %	-1.4611	0.87	0.87
GO:0007584	response to nutrient	0.047 %	-1.0410	0.87	0.59
GO:0010243	response to organonitrogen compound	0.768 %	-1.0719	0.87	0.62
GO:0009805	coumarin biosynthetic process	0.013 %	-1.6079	0.81	0.28
GO:0009804	coumarin metabolic process	0.017 %	-1.3421	0.84	0.60
GO:0009628	response to abiotic stimulus	7.946 %	-1.5123	0.87	0.29
GO:0008216	spermidine metabolic process	0.056 %	-1.2934	0.88	0.29
GO:0006595	polyamine metabolic process	0.095 %	-1.0717	0.88	0.82
GO:0010019	chloroplast-nucleus signaling pathway	0.039 %	-1.3634	0.75	0.30
GO:0050688	regulation of defense response to virus	0.039 %	-1.4217	0.77	0.30
GO:0002697	regulation of immune effector process	0.039 %	-1.4217	0.84	0.75
GO:0009627	systemic acquired resistance	0.281 %	-1.2726	0.86	0.60
GO:0006357	regulation of transcription from RNA polymerase II promoter	2.210 %	-1.0993	0.67	0.30
GO:0032774	RNA biosynthetic process	11.813 %	-1.0189	0.75	0.82
GO:0010468	regulation of gene expression	12.706 %	-1.2647	0.66	0.79
GO:0031323	regulation of cellular metabolic process	13.906 %	-1.2415	0.66	0.80
GO:0031326	regulation of cellular biosynthetic process	12.339 %	-1.2432	0.63	0.85
GO:0051252	regulation of RNA metabolic process	11.463 %	-1.5828	0.62	0.87
GO:2001141	regulation of RNA biosynthetic process	11.282 %	-1.6510	0.61	0.88
GO:0019219	regulation of nucleobase-containing compound metabolic process	11.700 %	-1.4897	0.63	0.84
GO:2000112	regulation of cellular macromolecule biosynthetic process	12.003 %	-1.0829	0.62	0.87
GO:0006355	regulation of transcription, DNA-templated	11.234 %	-1.6510	0.61	0.65
GO:0006351	transcription, DNA-templated	11.709 %	-1.0189	0.75	0.87
GO:0060255	regulation of macromolecule metabolic process	14.091 %	-1.0766	0.66	0.83
GO:1903506	regulation of nucleic acid-templated transcription	11.282 %	-1.6510	0.61	0.89

GO:0097659	nucleic acid-templated transcription	11.761 %	-1.0189	0.75	0.88
GO:0009889	regulation of biosynthetic process	12.408 %	-1.2161	0.65	0.78
GO:0010556	regulation of macromolecule biosynthetic process	12.076 %	-1.0804	0.63	0.87
GO:0051171	regulation of nitrogen compound metabolic process	12.430 %	-1.5292	0.65	0.76
GO:0055114	oxidation-reduction process	7.462 %	-1.0588	0.84	0.32
GO:0015827	tryptophan transport	0.004 %	-1.7187	0.85	0.32
GO:0015740	C4-dicarboxylate transport	0.099 %	-1.1956	0.83	0.82
GO:0006835	dicarboxylic acid transport	0.121 %	-1.1956	0.83	0.83
GO:0015800	acidic amino acid transport	0.035 %	-1.3634	0.83	0.68
GO:0015804	neutral amino acid transport	0.086 %	-1.3319	0.82	0.80
GO:0015801	aromatic amino acid transport	0.017 %	-1.7187	0.84	0.61
GO:0015802	basic amino acid transport	0.035 %	-1.4217	0.83	0.66
GO:1905039	carboxylic acid transmembrane transport	0.505 %	-1.3857	0.81	0.75
GO:0015809	arginine transport	0.017 %	-1.4345	0.84	0.63
GO:0015810	aspartate transport	0.009 %	-1.7187	0.84	0.56
GO:0043090	amino acid import	0.056 %	-1.0361	0.83	0.78
GO:1903825	organic acid transmembrane transport	0.505 %	-1.3857	0.81	0.94
GO:0003333	amino acid transmembrane transport	0.337 %	-1.3857	0.81	0.96
GO:0010193	response to ozone	0.129 %	-1.5383	0.87	0.33
GO:0000302	response to reactive oxygen species	0.678 %	-1.0129	0.86	0.67
GO:0015688	iron chelate transport	0.009 %	-3.7176	0.89	0.33
GO:0048477	oogenesis	0.009 %	-1.5706	0.85	0.34
GO:0007292	female gamete generation	0.022 %	-1.3319	0.88	0.70
GO:0007281	germ cell development	0.004 %	-1.5706	0.86	0.65
GO:0006071	glycerol metabolic process	0.117 %	-1.3857	0.82	0.35
GO:0051275	beta-glucan catabolic process	0.112 %	-1.2029	0.88	0.89
GO:0019400	alditol metabolic process	0.117 %	-1.3857	0.82	0.89
GO:0030245	cellulose catabolic process	0.112 %	-1.2029	0.88	0.58
GO:0050792	regulation of viral process	0.013 %	-1.1956	0.83	0.36
GO:0043903	regulation of symbiosis, encompassing mutualism through parasitism	0.026 %	-1.0361	0.84	0.73
GO:0043433	negative regulation of sequence-specific DNA binding transcription factor activity	0.022 %	-2.7268	0.77	0.36
GO:0019222	regulation of metabolic process	14.847 %	-1.1533	0.73	0.37
GO:0007166	cell surface receptor signaling pathway	1.204 %	-1.2335	0.67	0.38
GO:0009615	response to virus	0.337 %	-1.1505	0.88	0.42
GO:0014070	response to organic cyclic compound	1.606 %	-1.0154	0.86	0.43
GO:0016137	glycoside metabolic process	0.017 %	-1.1478	0.89	0.43
GO:0019722	calcium-mediated signaling	0.302 %	-1.0361	0.71	0.44
GO:0051592	response to calcium ion	0.052 %	-1.1414	0.89	0.45
GO:2000241	regulation of reproductive process	1.092 %	-1.4160	0.77	0.45
GO:0003006	developmental process involved in reproduction	5.611 %	-1.3002	0.92	0.69
GO:0010228	vegetative to reproductive phase transition of meristem	0.738 %	-1.3262	0.87	0.58
GO:0048831	regulation of shoot system development	0.850 %	-1.1937	0.75	0.80
GO:0009909	regulation of flower development	0.609 %	-1.3913	0.74	0.53
GO:0051090	regulation of sequence-specific DNA binding transcription factor activity	0.052 %	-2.4438	0.76	0.45
GO:0044092	negative regulation of molecular function	1.070 %	-1.7788	0.82	0.60
GO:0009314	response to radiation	2.892 %	-2.7508	0.82	0.45
GO:0009266	response to temperature stimulus	2.287 %	-1.1458	0.83	0.64
GO:0071214	cellular response to abiotic stimulus	0.889 %	-1.3945	0.80	0.56
GO:0009409	response to cold	1.580 %	-1.8328	0.82	0.61
GO:0010600	regulation of auxin biosynthetic process	0.052 %	-1.6279	0.69	0.46
GO:0090354	regulation of auxin metabolic process	0.056 %	-1.4345	0.70	0.96
GO:0032350	regulation of hormone metabolic process	0.073 %	-1.2754	0.72	0.67
GO:0009851	auxin biosynthetic process	0.250 %	-1.0936	0.70	0.84
GO:0046885	regulation of hormone biosynthetic process	0.065 %	-1.3974	0.68	0.97

Biological Process (95)   Tag Clouds

Scatterplot & Table   Interactive Graph   TreeMap

GROUP 18

Hide/show dispensable GO terms   Export results to text table (CSV)   Make R script for plotting

term ID	description	frequency	pin?	log_{10} p-value	uniqueness	dispensability
GO:0033365	protein localization to organelle	1.148 %		-2.7602	0.63	0.00
GO:0072666	establishment of protein localization to vacuole	0.155 %		-1.3199	0.66	1.00
GO:0072665	protein localization to vacuole	0.155 %		-1.3199	0.67	0.70
GO:0032507	maintenance of protein location in cell	0.078 %		-1.4370	0.52	0.97
GO:0034504	protein localization to nucleus	0.268 %		-2.9005	0.66	0.74
GO:0051457	maintenance of protein location in nucleus	0.013 %		-2.4408	0.55	0.57
GO:0070727	cellular macromolecule localization	2.167 %		-1.5998	0.64	0.82
GO:0006606	protein import into nucleus	0.242 %		-1.3318	0.59	0.87
GO:1902593	single-organism nuclear import	0.242 %		-1.3318	0.63	0.99
GO:0006605	protein targeting	1.006 %		-1.5833	0.63	0.84
GO:0000060	protein import into nucleus, translocation	0.039 %		-2.1684	0.65	0.70
GO:0034613	cellular protein localization	2.016 %		-1.7924	0.62	0.90
GO:0006623	protein targeting to vacuole	0.155 %		-1.3199	0.66	0.70
GO:0006886	intracellular protein transport	1.675 %		-1.1009	0.62	0.94
GO:0051651	maintenance of location in cell	0.155 %		-1.3199	0.52	0.85
GO:0044744	protein targeting to nucleus	0.246 %		-1.3318	0.63	0.98
GO:0008104	protein localization	3.448 %		-1.4749	0.66	0.82
GO:0045185	maintenance of protein location	0.078 %		-1.3655	0.58	0.72

GO:0072594	establishment of protein localization to organelle	0.924 %	-1.6821	0.62	0.84
GO:0051170	nuclear import	0.246 %	-1.2682	0.67	0.89
GO:0072595	maintenance of protein localization in organelle	0.047 %	-1.9306	0.52	0.86
GO:1900000	regulation of anthocyanin catabolic process	0.004 %	-2.3440	0.73	0.00
GO:0031537	regulation of anthocyanin metabolic process	0.095 %	-1.3788	0.73	0.74
GO:0016139	glycoside catabolic process	0.009 %	-1.6754	0.84	0.89
GO:0046149	pigment catabolic process	0.082 %	-1.4423	0.87	0.56
GO:0046284	anthocyanin-containing compound catabolic process	0.004 %	-2.3440	0.82	1.00
GO:0046275	flavonoid catabolic process	0.004 %	-2.3440	0.91	0.54
GO:1901658	glycosyl compound catabolic process	0.211 %	-1.2382	0.83	0.70
GO:0046500	S-adenosylmethionine metabolic process	0.043 %	-1.7342	0.93	0.04
GO:0006556	S-adenosylmethionine biosynthetic process	0.017 %	-1.8834	0.92	0.53
GO:0010076	maintenance of floral meristem identity	0.026 %	-2.0027	0.77	0.06
GO:0019827	stem cell population maintenance	0.216 %	-1.0979	0.81	0.97
GO:0010067	procambium histogenesis	0.039 %	-1.4021	0.78	0.57
GO:0010065	primary meristem tissue development	0.043 %	-1.3318	0.77	0.86
GO:0010582	floral meristem determinacy	0.052 %	-1.5566	0.77	0.69
GO:0048510	regulation of timing of transition from vegetative to reproductive phase	0.164 %	-1.0979	0.68	0.66
GO:0048507	meristem development	0.945 %	-1.0810	0.81	0.59
GO:0048508	embryonic meristem development	0.125 %	-1.0309	0.77	0.71
GO:0048506	regulation of timing of meristematic phase transition	0.164 %	-1.0979	0.68	0.95
GO:0010074	maintenance of meristem identity	0.155 %	-1.2788	0.75	0.86
GO:0010077	maintenance of inflorescence meristem identity	0.039 %	-1.8688	0.77	0.80
GO:0010022	meristem determinacy	0.056 %	-1.5566	0.78	0.72
GO:0030007	cellular potassium ion homeostasis	0.022 %	-1.6488	0.74	0.10
GO:0055075	potassium ion homeostasis	0.069 %	-1.5108	0.76	0.86
GO:0055067	monovalent inorganic cation homeostasis	0.324 %	-1.3238	0.74	0.63
GO:0030004	cellular monovalent inorganic cation homeostasis	0.160 %	-1.4985	0.71	0.92
GO:0006885	regulation of pH	0.255 %	-1.6158	0.74	0.79
GO:0010219	regulation of vernalization response	0.022 %	-2.1983	0.79	0.12
GO:0010048	vernalization response	0.065 %	-1.7557	0.90	0.52
GO:0010220	positive regulation of vernalization response	0.013 %	-2.1983	0.72	0.84
GO:0008356	asymmetric cell division	0.091 %	-1.3568	0.90	0.15
GO:0009739	response to gibberellin	0.626 %	-1.4585	0.92	0.15
GO:0006730	one-carbon metabolic process	0.164 %	-1.3400	0.87	0.17
GO:0045893	positive regulation of transcription, DNA-templated	1.864 %	-1.3490	0.64	0.24
GO:1903508	positive regulation of nucleic acid-templated transcription	1.869 %	-1.3490	0.64	0.98
GO:0009893	positive regulation of metabolic process	2.892 %	-1.0528	0.67	0.79
GO:0010604	positive regulation of macromolecule metabolic process	2.672 %	-1.1420	0.64	0.92
GO:0009891	positive regulation of biosynthetic process	2.145 %	-1.2188	0.65	0.89
GO:0080090	regulation of primary metabolic process	13.681 %	-1.0211	0.71	0.54
GO:1902680	positive regulation of RNA biosynthetic process	1.869 %	-1.3490	0.64	0.97
GO:0031328	positive regulation of cellular biosynthetic process	2.102 %	-1.2301	0.64	0.97
GO:0031325	positive regulation of cellular metabolic process	2.741 %	-1.1208	0.64	0.95
GO:0010557	positive regulation of macromolecule biosynthetic process	2.007 %	-1.2700	0.65	0.95
GO:0051254	positive regulation of RNA metabolic process	1.912 %	-1.3490	0.64	0.96
GO:0045935	positive regulation of nucleobase-containing compound metabolic process	2.007 %	-1.3143	0.64	0.96
GO:0051173	positive regulation of nitrogen compound metabolic process	2.119 %	-1.2399	0.65	0.88
GO:0010628	positive regulation of gene expression	2.050 %	-1.2870	0.65	0.90
GO:0009956	radial pattern formation	0.078 %	-1.3007	0.85	0.26
GO:0065008	regulation of biological quality	5.991 %	-1.3888	0.81	0.26
GO:0098727	maintenance of cell number	0.216 %	-1.0979	0.88	0.27
GO:0007034	vacuolar transport	0.293 %	-1.0328	0.78	0.36
GO:0008643	carbohydrate transport	0.630 %	-1.1361	0.70	0.38
GO:0044765	single-organism transport	5.287 %	-1.1047	0.65	0.69
GO:0010817	regulation of hormone levels	1.109 %	-1.3084	0.75	0.41
GO:0051235	maintenance of location	0.302 %	-1.1262	0.63	0.51
GO:0010540	basipetal auxin transport	0.078 %	-1.0486	0.62	0.68
GO:0009690	cytokinin metabolic process	0.168 %	-1.3278	0.75	0.80
GO:0034754	cellular hormone metabolic process	0.263 %	-1.3082	0.75	0.51
GO:0016137	glycoside metabolic process	0.017 %	-1.5363	0.87	0.41
GO:0010100	negative regulation of photomorphogenesis	0.039 %	-1.5363	0.68	0.44
GO:2000030	regulation of response to red or far red light	0.134 %	-1.2614	0.76	0.65
GO:0010099	regulation of photomorphogenesis	0.104 %	-1.3743	0.66	0.90
GO:0048575	short-day photoperiodism, flowering	0.056 %	-1.5046	0.77	0.45
GO:2000028	regulation of photoperiodism, flowering	0.190 %	-1.3880	0.64	0.80
GO:0048572	short-day photoperiodism	0.056 %	-1.5046	0.88	0.73
GO:1901136	carbohydrate derivative catabolic process	0.337 %	-1.0693	0.90	0.46
GO:0051641	cellular localization	3.008 %	-1.3422	0.74	0.46
GO:0071702	organic substance transport	5.585 %	-1.0504	0.72	0.63
GO:1902578	single-organism localization	5.481 %	-1.7125	0.68	0.61

GO:0033036	*macromolecule localization*	*4.519 %*	*-1.1808*	*0.73*	*0.56*
GO:0010167	response to nitrate	0.078 %	-1.1463	0.91	0.46
GO:0071281	cellular response to iron ion	0.177 %	-1.1386	0.89	0.47
GO:0071248	*cellular response to metal ion*	*0.216 %*	*-1.0048*	*0.89*	*0.96*
GO:0010039	*response to iron ion*	*0.237 %*	*-1.0012*	*0.91*	*0.62*
GO:0030104	water homeostasis	0.052 %	-1.3655	0.78	0.50

Biological Process (164) Tag Clouds

Scatterplot & Table Interactive Graph TreeMap

GROUP 19

Hide/show dispensable GO terms

Export results to text table (CSV)

Make R script for plotting

term ID	description	frequency	pin?	log_{10} p-value	uniqueness	dispensability
GO:0019684	photosynthesis, light reaction	0.505 %		-4.5392	0.86	0.00
GO:0009773	photosynthetic electron transport in photosystem I	0.065 %		-4.4916	0.76	0.71
GO:0042548	regulation of photosynthesis, light reaction	0.108 %		-1.8856	0.82	0.81
GO:0009767	photosynthetic electron transport chain	0.224 %		-3.6239	0.73	0.79
GO:0043467	regulation of generation of precursor metabolites and energy	0.117 %		-1.8014	0.83	0.69
GO:0010206	photosystem II repair	0.056 %		-1.0800	0.87	0.70
GO:0010207	photosystem II assembly	0.086 %		-1.1514	0.86	0.80
GO:0010109	regulation of photosynthesis	0.160 %		-1.5620	0.83	0.77
GO:0022900	electron transport chain	0.479 %		-3.0071	0.74	0.78
GO:0042754	negative regulation of circadian rhythm	0.013 %		-5.3860	0.91	0.00
GO:0007623	circadian rhythm	0.539 %		-1.9825	0.97	0.90
GO:0042752	regulation of circadian rhythm	0.220 %		-2.4990	0.91	0.69
GO:0048511	rhythmic process	0.600 %		-1.9634	0.99	0.00
GO:0034484	raffinose catabolic process	0.004 %		-4.5187	0.82	0.03
GO:0044724	single-organism carbohydrate catabolic process	0.432 %		-1.7969	0.76	0.66
GO:0033530	raffinose metabolic process	0.013 %		-4.0454	0.83	0.56
GO:0009313	oligosaccharide catabolic process	0.039 %		-3.6336	0.79	0.64
GO:0009311	oligosaccharide metabolic process	0.466 %		-1.6970	0.79	0.68
GO:0048574	long-day photoperiodism, flowering	0.121 %		-3.7464	0.70	0.06

GO:0010228	vegetative to reproductive phase transition of meristem	0.738 %	-1.2327	0.81	0.50
GO:0048573	photoperiodism, flowering	0.388 %	-1.9260	0.68	0.90
GO:0010114	response to red light	0.259 %	-1.2214	0.84	0.72
GO:0048571	long-day photoperiodism	0.138 %	-3.4816	0.84	0.82
GO:0009642	response to light intensity	0.587 %	-1.9528	0.83	0.79
GO:0009648	photoperiodism	0.427 %	-1.7216	0.83	0.76
GO:0009416	response to light stimulus	2.771 %	-2.5113	0.80	0.67
GO:0009056	catabolic process	8.118 %	-2.3730	0.94	0.07
GO:0042726	flavin-containing compound metabolic process	0.069 %	-1.2560	0.89	0.07
GO:1902356	oxaloacetate(2-) transmembrane transport	0.022 %	-2.2090	0.82	0.07
GO:0015740	C4-dicarboxylate transport	0.099 %	-1.1585	0.81	0.96
GO:0015743	malate transport	0.086 %	-1.3047	0.81	0.98
GO:0015742	alpha-ketoglutarate transport	0.009 %	-1.9548	0.83	0.75
GO:0006835	dicarboxylic acid transport	0.121 %	-1.1585	0.82	0.71
GO:0015813	L-glutamate transport	0.022 %	-2.0634	0.82	0.79
GO:0015800	acidic amino acid transport	0.035 %	-1.3259	0.83	0.72
GO:0015807	L-amino acid transport	0.078 %	-2.0634	0.82	0.64
GO:0071423	malate transmembrane transport	0.086 %	-1.8682	0.81	0.89
GO:0015729	oxaloacetate transport	0.022 %	-2.2090	0.82	0.82
GO:0019740	nitrogen utilization	0.039 %	-1.1808	0.91	0.07
GO:0007267	cell-cell signaling	0.371 %	-1.2560	0.82	0.08
GO:0015979	photosynthesis	1.057 %	-3.4617	0.92	0.08
GO:0006091	generation of precursor metabolites and energy	1.459 %	-2.4628	0.91	0.09
GO:0006805	xenobiotic metabolic process	0.009 %	-3.5929	0.83	0.12
GO:0071466	cellular response to xenobiotic stimulus	0.009 %	-3.5929	0.86	0.96
GO:0042178	xenobiotic catabolic process	0.004 %	-3.8033	0.81	0.96
GO:0010600	regulation of auxin biosynthetic process	0.052 %	-3.5043	0.81	0.13
GO:0090354	regulation of auxin metabolic process	0.056 %	-3.1081	0.83	0.96
GO:0032350	regulation of hormone metabolic process	0.073 %	-2.7836	0.85	0.67
GO:0009850	auxin metabolic process	0.332 %	-1.6266	0.83	0.90
GO:0009851	auxin biosynthetic process	0.250 %	-2.4135	0.81	0.84
GO:0046885	regulation of hormone biosynthetic process	0.065 %	-3.0324	0.81	0.97
GO:0042445	hormone metabolic process	0.695 %	-1.1666	0.84	0.73
GO:0042446	hormone biosynthetic process	0.514 %	-1.8452	0.82	0.88
GO:0044710	single-organism metabolic process	18.390 %	-3.5349	0.84	0.14
GO:0065009	regulation of molecular function	2.443 %	-1.2356	0.92	0.16
GO:0009611	response to wounding	0.816 %	-2.8556	0.89	0.17
GO:0008202	steroid metabolic process	0.427 %	-2.4938	0.77	0.18
GO:0046345	abscisic acid catabolic process	0.022 %	-1.9548	0.70	0.73
GO:0008299	isoprenoid biosynthetic process	0.738 %	-1.7521	0.70	0.96
GO:0033559	unsaturated fatty acid metabolic process	0.112 %	-1.2748	0.72	0.65
GO:0006636	unsaturated fatty acid biosynthetic process	0.108 %	-1.2748	0.70	0.60
GO:0016099	monoterpenoid biosynthetic process	0.004 %	-1.5700	0.78	0.65
GO:0016098	monoterpenoid metabolic process	0.004 %	-1.5700	0.80	0.65
GO:0016114	terpenoid biosynthetic process	0.591 %	-2.0087	0.70	0.55
GO:0008610	lipid biosynthetic process	2.464 %	-1.1629	0.72	0.69
GO:0006720	isoprenoid metabolic process	0.850 %	-1.5672	0.73	0.67
GO:0006721	terpenoid metabolic process	0.699 %	-1.7861	0.72	0.94
GO:0009410	response to xenobiotic stimulus	0.013 %	-2.5370	0.90	0.20
GO:0009696	salicylic acid metabolic process	0.121 %	-1.9220	0.73	0.23
GO:0032787	monocarboxylic acid metabolic process	2.128 %	-1.4292	0.69	0.82
GO:0006067	ethanol metabolic process	0.004 %	-1.5700	0.81	0.75
GO:0043436	oxoacid metabolic process	5.002 %	-1.2488	0.67	0.93
GO:0019752	carboxylic acid metabolic process	4.437 %	-1.5554	0.68	0.58
GO:0006082	organic acid metabolic process	5.015 %	-1.1269	0.69	0.79
GO:0034308	primary alcohol metabolic process	0.052 %	-1.1109	0.79	0.60
GO:0006069	ethanol oxidation	0.004 %	-1.5700	0.81	0.51
GO:0009694	jasmonic acid metabolic process	0.117 %	-1.7304	0.75	0.56
GO:0009904	chloroplast accumulation movement	0.095 %	-1.3599	0.86	0.24
GO:0019750	chloroplast localization	0.134 %	-1.0045	0.90	1.00
GO:0009903	chloroplast avoidance movement	0.112 %	-1.2381	0.86	0.97
GO:0009902	chloroplast relocation	0.129 %	-1.0045	0.86	0.99
GO:0051667	establishment of plastid localization	0.129 %	-1.0045	0.90	0.99
GO:0051644	plastid localization	0.134 %	-1.0045	0.90	0.84
GO:0006081	cellular aldehyde metabolic process	0.311 %	-1.6790	0.79	0.25
GO:0009628	response to abiotic stimulus	7.946 %	-2.7007	0.88	0.27
GO:0035966	response to topologically incorrect protein	0.151 %	-1.0800	0.87	0.30
GO:0006986	response to unfolded protein	0.091 %	-1.1658	0.86	0.33
GO:0035967	cellular response to topologically incorrect protein	0.112 %	-1.1658	0.83	0.94
GO:0034620	cellular response to unfolded protein	0.065 %	-1.1658	0.83	0.90
GO:0044712	single-organism catabolic process	2.676 %	-1.9060	0.75	0.34

GO:0016052	carbohydrate catabolic process	1.187 %	-1.7721	0.87	0.57
GO:1901575	organic substance catabolic process	7.402 %	-1.6149	0.87	0.74
GO:0009814	defense response, incompatible interaction	0.725 %	-1.1573	0.88	0.34
GO:0009627	systemic acquired resistance	0.281 %	-1.2049	0.89	0.78
GO:0007389	pattern specification process	0.742 %	-1.4455	0.82	0.35
GO:0016139	glycoside catabolic process	0.009 %	-2.8018	0.82	0.36
GO:1901658	glycosyl compound catabolic process	0.211 %	-1.9345	0.78	0.73
GO:0048838	release of seed from dormancy	0.009 %	-1.4669	0.85	0.36
GO:0097438	exit from dormancy	0.009 %	-1.4669	0.86	0.70
GO:0043433	negative regulation of sequence-specific DNA binding transcription factor activity	0.022 %	-4.3169	0.84	0.36
GO:1900033	negative regulation of trichome patterning	0.022 %	-2.2090	0.66	0.38
GO:0045168	cell-cell signaling involved in cell fate commitment	0.168 %	-1.8682	0.72	0.76
GO:0003002	regionalization	0.617 %	-1.6298	0.80	0.72
GO:1903888	regulation of plant epidermal cell differentiation	0.009 %	-1.9548	0.73	0.67
GO:0010455	positive regulation of cell fate commitment	0.013 %	-1.5900	0.69	0.89
GO:0010454	negative regulation of cell fate commitment	0.022 %	-2.2090	0.69	0.91
GO:0010453	regulation of cell fate commitment	0.047 %	-1.5510	0.69	0.81
GO:1903890	positive regulation of plant epidermal cell differentiation	0.009 %	-1.9548	0.71	1.00
GO:1900457	regulation of brassinosteroid mediated signaling pathway	0.017 %	-1.3259	0.75	0.52
GO:1900459	positive regulation of brassinosteroid mediated signaling pathway	0.013 %	-1.3259	0.73	0.66
GO:0045597	positive regulation of cell differentiation	0.030 %	-1.2044	0.70	0.72
GO:0045596	negative regulation of cell differentiation	0.052 %	-1.0921	0.70	0.76
GO:0010057	trichoblast fate specification	0.009 %	-2.0057	0.74	1.00
GO:0048629	trichome patterning	0.030 %	-1.8682	0.73	0.78
GO:0010063	positive regulation of trichoblast fate specification	0.009 %	-2.0057	0.67	0.86
GO:0010061	regulation of trichoblast fate specification	0.009 %	-2.0057	0.68	1.00
GO:0042660	positive regulation of cell fate specification	0.009 %	-1.9548	0.69	0.96
GO:0042659	regulation of cell fate specification	0.017 %	-1.9548	0.69	0.89
GO:0010051	xylem and phloem pattern formation	0.259 %	-1.6921	0.81	0.88
GO:1900032	regulation of trichome patterning	0.022 %	-2.2090	0.67	0.96
GO:0090628	plant epidermal cell fate specification	0.009 %	-1.9548	0.76	0.64
GO:0006814	sodium ion transport	0.121 %	-1.3151	0.92	0.40
GO:0019509	L-methionine biosynthetic process from methylthioadenosine	0.039 %	-1.6561	0.72	0.41
GO:0006547	histidine metabolic process	0.047 %	-1.3718	0.74	1.00
GO:0071265	L-methionine biosynthetic process	0.052 %	-1.4519	0.72	0.88
GO:0071267	L-methionine salvage	0.043 %	-1.6561	0.72	0.99
GO:0000105	histidine biosynthetic process	0.047 %	-1.3718	0.73	0.61
GO:0009086	methionine biosynthetic process	0.112 %	-1.0351	0.71	0.92
GO:0019676	ammonia assimilation cycle	0.026 %	-1.4099	0.76	0.53
GO:0043102	amino acid salvage	0.043 %	-1.6561	0.73	0.70
GO:0009723	response to ethylene	1.269 %	-1.0183	0.85	0.41
GO:0006629	lipid metabolic process	4.683 %	-1.5412	0.77	0.41
GO:0009414	response to water deprivation	1.390 %	-3.1884	0.79	0.42
GO:0001101	response to acid chemical	5.011 %	-1.7711	0.84	0.50
GO:1901700	response to oxygen-containing compound	6.504 %	-1.0552	0.84	0.64
GO:0009753	response to jasmonic acid	0.919 %	-2.2626	0.85	0.63
GO:0009751	response to salicylic acid	0.876 %	-1.0846	0.84	0.63
GO:0009739	response to gibberellin	0.626 %	-1.3286	0.85	0.61
GO:0009269	response to desiccation	0.073 %	-2.2765	0.84	0.73
GO:0009314	response to radiation	2.892 %	-2.4607	0.83	0.60
GO:0071214	cellular response to abiotic stimulus	0.889 %	-1.2996	0.81	0.52
GO:0009415	response to water	1.416 %	-3.1342	0.80	0.74
GO:0016137	glycoside metabolic process	0.017 %	-2.5238	0.83	0.43
GO:0009657	plastid organization	1.032 %	-1.1666	0.93	0.44
GO:0046471	phosphatidylglycerol metabolic process	0.047 %	-2.0057	0.78	0.45
GO:0006950	response to stress	14.156 %	-2.0450	0.87	0.45
GO:1905423	positive regulation of plant organ morphogenesis	0.009 %	-1.9548	0.77	0.45
GO:2000067	regulation of root morphogenesis	0.039 %	-1.2748	0.76	0.82
GO:1905421	regulation of plant organ morphogenesis	0.086 %	-1.1808	0.76	0.64
GO:1900057	positive regulation of leaf senescence	0.039 %	-1.8306	0.76	0.64
GO:1900055	regulation of leaf senescence	0.104 %	-1.1173	0.76	0.83
GO:0009231	riboflavin biosynthetic process	0.052 %	-1.2748	0.73	0.45
GO:0042727	flavin-containing compound biosynthetic process	0.056 %	-1.2748	0.86	0.97
GO:0006771	riboflavin metabolic process	0.052 %	-1.2748	0.76	0.96
GO:0051090	regulation of sequence-specific DNA binding transcription factor activity	0.052 %	-3.8869	0.84	0.45
GO:0044092	negative regulation of molecular function	1.070 %	-2.8763	0.91	0.60
GO:0010117	photoprotection	0.026 %	-3.2515	0.86	0.47
GO:0071484	cellular response to light intensity	0.043 %	-2.8989	0.82	0.48
GO:0009644	response to high light intensity	0.324 %	-2.5505	0.83	0.76
GO:0071482	cellular response to light stimulus	0.561 %	-1.9476	0.79	0.79
GO:0071478	cellular response to radiation	0.583 %	-1.9476	0.79	0.91

GO:0055114	oxidation-reduction process	7.462 %	-2.2370	0.76	0.49
GO:0080167	response to karrikin	0.531 %	-1.0363	0.85	0.49
GO:1901136	carbohydrate derivative catabolic process	0.337 %	-1.6058	0.89	0.50

Biological Process (67) Tag Clouds

Scatterplot & Table Interactive Graph TreeMap

GROUP 20

Hide/show dispensable GO terms

Export results to text table (CSV)

Make R script for plotting

term ID	description	frequency	pin?	log_{10} p-value	uniqueness	dispensability
GO:0031396	regulation of protein ubiquitination	0.078 %		-2.2706	0.84	0.00
GO:1903320	*regulation of protein modification by small protein conjugation or removal*	*0.082 %*		*-2.2706*	*0.84*	*0.69*
GO:0045471	response to ethanol	0.613 %		-2.3119	0.87	0.00
GO:0006461	protein complex assembly	1.303 %		-1.1783	0.84	0.04
GO:0070271	*protein complex biogenesis*	*1.334 %*		*-1.1559*	*0.91*	*0.62*
GO:0071822	*protein complex subunit organization*	*1.528 %*		*-1.1293*	*0.86*	*0.67*
GO:0051259	*protein oligomerization*	*0.224 %*		*-1.1153*	*0.86*	*0.82*
GO:0065003	*macromolecular complex assembly*	*3.220 %*		*-1.0243*	*0.84*	*0.80*
GO:0006642	triglyceride mobilization	0.004 %		-2.2706	0.77	0.05
GO:0006639	*acylglycerol metabolic process*	*0.147 %*		*-1.3952*	*0.73*	*0.99*
GO:0006641	*triglyceride metabolic process*	*0.129 %*		*-1.4917*	*0.73*	*0.78*
GO:0006085	acetyl-CoA biosynthetic process	0.056 %		-1.9331	0.70	0.06
GO:0006732	*coenzyme metabolic process*	*1.191 %*		*-1.2331*	*0.80*	*0.67*
GO:0006637	*acyl-CoA metabolic process*	*0.151 %*		*-1.4323*	*0.71*	*0.97*
GO:0071616	*acyl-CoA biosynthetic process*	*0.082 %*		*-1.7023*	*0.70*	*0.97*
GO:0035383	*thioester metabolic process*	*0.151 %*		*-1.4323*	*0.80*	*0.60*
GO:0035384	*thioester biosynthetic process*	*0.082 %*		*-1.7023*	*0.74*	*0.89*
GO:0006086	*acetyl-CoA biosynthetic process from pyruvate*	*0.035 %*		*-2.0822*	*0.59*	*0.92*
GO:0006084	*acetyl-CoA metabolic process*	*0.108 %*		*-1.6002*	*0.72*	*0.93*

GO:0015749	monosaccharide transport	0.229 %	-1.9331	0.88	0.06
GO:0006874	cellular calcium ion homeostasis	0.047 %	-1.4729	0.78	0.13
GO:0055074	*calcium ion homeostasis*	0.060 %	-1.2731	0.89	0.86
GO:0072507	*divalent inorganic cation homeostasis*	0.147 %	-1.0648	0.90	0.63
GO:0072503	*cellular divalent inorganic cation homeostasis*	0.099 %	-1.2167	0.77	0.91
GO:0019563	glycerol catabolic process	0.017 %	-2.2706	0.63	0.15
GO:0019400	*alditol metabolic process*	0.117 %	-1.7465	0.62	0.89
GO:0019405	*alditol catabolic process*	0.017 %	-2.2706	0.63	0.85
GO:1901616	*organic hydroxy compound catabolic process*	0.121 %	-1.3474	0.77	0.56
GO:0019751	*polyol metabolic process*	0.246 %	-1.1391	0.66	0.72
GO:0046174	*polyol catabolic process*	0.060 %	-1.4550	0.64	0.92
GO:0046164	*alcohol catabolic process*	0.082 %	-1.4213	0.65	0.85
GO:0044275	*cellular carbohydrate catabolic process*	0.298 %	-1.0281	0.74	0.61
GO:0006071	*glycerol metabolic process*	0.117 %	-1.7465	0.61	0.88
GO:0010187	negative regulation of seed germination	0.073 %	-1.2807	0.88	0.15
GO:0046184	aldehyde biosynthetic process	0.052 %	-2.1662	0.73	0.17
GO:0019682	*glyceraldehyde-3-phosphate metabolic process*	0.168 %	-1.1790	0.65	0.83
GO:0046166	*glyceraldehyde-3-phosphate biosynthetic process*	0.009 %	-2.2706	0.69	0.68
GO:0009644	response to high light intensity	0.324 %	-1.6567	0.87	0.18
GO:0009642	*response to light intensity*	0.587 %	-1.3424	0.86	0.64
GO:1901137	carbohydrate derivative biosynthetic process	1.800 %	-1.1889	0.79	0.20
GO:0009100	*glycoprotein metabolic process*	0.496 %	-1.0022	0.81	0.70
GO:0009101	*glycoprotein biosynthetic process*	0.488 %	-1.0022	0.77	0.99
GO:0043413	*macromolecule glycosylation*	0.483 %	-1.0022	0.75	0.97
GO:0006486	*protein glycosylation*	0.483 %	-1.0022	0.66	0.70
GO:0031668	cellular response to extracellular stimulus	0.691 %	-1.2209	0.80	0.20
GO:0042631	*cellular response to water deprivation*	0.129 %	-1.1418	0.75	0.84
GO:0009991	*response to extracellular stimulus*	0.777 %	-1.1172	0.87	0.53
GO:0009970	*cellular response to sulfate starvation*	0.052 %	-1.3474	0.81	0.78
GO:0071496	*cellular response to external stimulus*	0.712 %	-1.2006	0.87	0.52
GO:0006081	cellular aldehyde metabolic process	0.311 %	-1.0763	0.77	0.21
GO:0080022	primary root development	0.091 %	-1.2439	0.90	0.28
GO:0080160	selenate transport	0.077 %	-1.8986	0.95	0.29
GO:0046685	response to arsenic-containing substance	0.060 %	-1.3952	0.89	0.29
GO:0046688	response to copper ion	0.069 %	-1.2167	0.88	0.30
GO:0006090	pyruvate metabolic process	0.384 %	-1.9536	0.71	0.34
GO:0071472	cellular response to salt stress	0.147 %	-1.1473	0.80	0.38
GO:0071462	*cellular response to water stimulus*	0.129 %	-1.1418	0.79	0.71
GO:0071470	*cellular response to osmotic stress*	0.207 %	-1.0302	0.80	0.74
GO:1990267	response to transition metal nanoparticle	2.017 %	-1.4520	0.87	0.41
GO:0009395	phospholipid catabolic process	0.052 %	-1.8227	0.67	0.43
GO:0006638	neutral lipid metabolic process	0.147 %	-1.3952	0.75	0.47
GO:0006094	gluconeogenesis	0.095 %	-1.5180	0.67	0.47
GO:0046364	*monosaccharide biosynthetic process*	0.237 %	-1.3565	0.66	0.80
GO:0006006	*glucose metabolic process*	0.199 %	-1.2200	0.68	0.87
GO:0019319	*hexose biosynthetic process*	0.104 %	-1.3901	0.67	0.87
GO:0046434	organophosphate catabolic process	0.112 %	-1.2769	0.78	0.49
GO:0008643	carbohydrate transport	0.630 %	-1.1052	0.88	0.50

Biological Process (171) Tag Clouds

Scatterplot & Table Interactive Graph TreeMap

GROUP 21

Hide/show dispensable GO terms

Export results to text table (CSV) Make R script for plotting

term ID	description	frequency	pin?	log_{10} p-value	uniqueness	dispensability
GO:0010105	negative regulation of ethylene-activated signaling pathway	0.073 %		-4.1611	0.53	0.00
GO:0001933	negative regulation of protein phosphorylation	0.134 %		-1.7739	0.55	0.99
GO:0048523	negative regulation of cellular process	2.719 %		-1.9746	0.57	0.74
GO:0001932	regulation of protein phosphorylation	0.432 %		-1.0558	0.62	0.98
GO:0045859	regulation of protein kinase activity	0.423 %		-1.1008	0.56	0.90
GO:0045861	negative regulation of proteolysis	0.181 %		-1.8823	0.56	0.88
GO:0032269	negative regulation of cellular protein metabolic process	0.673 %		-2.2148	0.53	0.64
GO:0023057	negative regulation of signaling	0.345 %		-3.4474	0.57	0.69
GO:0032268	regulation of cellular protein metabolic process	1.921 %		-1.2369	0.63	0.84
GO:0010466	negative regulation of peptidase activity	0.173 %		-1.8823	0.51	0.99
GO:0009740	gibberellic acid mediated signaling pathway	0.341 %		-1.1056	0.63	0.88
GO:0051248	negative regulation of protein metabolic process	0.678 %		-2.2148	0.57	0.75
GO:0010476	gibberellin mediated signaling pathway	0.345 %		-1.0600	0.63	0.99
GO:0010951	negative regulation of endopeptidase activity	0.168 %		-1.8823	0.51	0.87
GO:0052548	regulation of endopeptidase activity	0.173 %		-1.8823	0.62	0.99
GO:0042325	regulation of phosphorylation	0.462 %		-1.0350	0.67	0.95
GO:0042326	negative regulation of phosphorylation	0.138 %		-1.7621	0.58	0.97
GO:0048585	negative regulation of response to stimulus	0.699 %		-2.4392	0.59	0.59

GO:0010104	regulation of ethylene-activated signaling pathway	0.095 %	-3.5537	0.63	0.98
GO:0030162	regulation of proteolysis	0.591 %	-1.7286	0.67	0.74
GO:0010648	negative regulation of cell communication	0.345 %	-3.4474	0.58	0.69
GO:0045936	negative regulation of phosphate metabolic process	0.164 %	-1.6076	0.57	0.99
GO:0009788	negative regulation of abscisic acid-activated signaling pathway	0.160 %	-1.0077	0.52	0.93
GO:0071369	cellular response to ethylene stimulus	0.906 %	-1.8558	0.80	0.91
GO:0071370	cellular response to gibberellin stimulus	0.354 %	-1.0310	0.80	0.89
GO:2000117	negative regulation of cysteine-type endopeptidase activity	0.004 %	-2.1819	0.60	0.66
GO:1902531	regulation of intracellular signal transduction	0.229 %	-2.8240	0.64	0.76
GO:1902532	negative regulation of intracellular signal transduction	0.091 %	-4.1611	0.55	0.85
GO:0070298	negative regulation of phosphorelay signal transduction system	0.073 %	-4.1611	0.55	0.97
GO:0009873	ethylene-activated signaling pathway	0.811 %	-2.0985	0.61	0.77
GO:0070297	regulation of phosphorelay signal transduction system	0.095 %	-3.5537	0.65	0.87
GO:0000160	phosphorelay signal transduction system	1.049 %	-2.0530	0.64	0.57
GO:0010563	negative regulation of phosphorus metabolic process	0.164 %	-1.6076	0.58	0.68
GO:0006469	negative regulation of protein kinase activity	0.134 %	-1.7739	0.50	0.85
GO:0043549	regulation of kinase activity	0.427 %	-1.0799	0.59	0.98
GO:0009968	negative regulation of signal transduction	0.332 %	-3.4691	0.52	0.89
GO:0009966	regulation of signal transduction	1.109 %	-2.1637	0.60	0.87
GO:0033673	negative regulation of kinase activity	0.134 %	-1.7739	0.51	0.99
GO:0009937	regulation of gibberellic acid mediated signaling pathway	0.073 %	-1.3078	0.64	0.73
GO:0009939	positive regulation of gibberellic acid mediated signaling pathway	0.026 %	-1.8384	0.66	0.58
GO:1901420	negative regulation of response to alcohol	0.160 %	-1.0077	0.60	0.72
GO:0031400	negative regulation of protein modification process	0.220 %	-1.4946	0.57	0.89
GO:0000280	nuclear division	1.079 %	-1.8821	0.85	0.04
GO:0048285	organelle fission	1.239 %	-1.7040	0.87	0.58
GO:0007126	meiotic nuclear division	0.470 %	-1.0411	0.70	0.88
GO:0007067	mitotic nuclear division	0.699 %	-1.1625	0.73	0.91
GO:0051783	regulation of nuclear division	0.181 %	-1.4262	0.71	0.80
GO:1903046	meiotic cell cycle process	0.613 %	-1.0370	0.74	0.94
GO:0040020	regulation of meiotic nuclear division	0.047 %	-1.5917	0.62	0.71
GO:0048838	release of seed from dormancy	0.009 %	-1.8823	0.85	0.06
GO:2000034	regulation of seed maturation	0.060 %	-1.5009	0.71	0.83
GO:2000033	regulation of seed dormancy process	0.039 %	-1.7861	0.70	0.90
GO:1902039	negative regulation of seed dormancy process	0.009 %	-2.0498	0.63	0.82
GO:2000692	negative regulation of seed maturation	0.017 %	-1.8671	0.62	0.65
GO:0010162	seed dormancy process	0.142 %	-1.2700	0.82	0.91
GO:0097438	exit from dormancy	0.009 %	-1.8823	0.88	0.70
GO:0071836	nectar secretion	0.017 %	-2.5491	0.84	0.06
GO:0009395	phospholipid catabolic process	0.052 %	-1.8384	0.76	0.07
GO:0010212	response to ionizing radiation	0.129 %	-1.5404	0.92	0.14
GO:0043086	negative regulation of catalytic activity	1.019 %	-3.2132	0.68	0.15
GO:0052547	regulation of peptidase activity	0.181 %	-1.8823	0.63	0.68
GO:0034762	regulation of transmembrane transport	0.216 %	-1.1203	0.66	0.81
GO:0034763	negative regulation of transmembrane transport	0.017 %	-1.7621	0.59	0.95
GO:0010360	negative regulation of anion channel activity	0.017 %	-1.7621	0.53	0.68
GO:0010359	regulation of anion channel activity	0.047 %	-1.2034	0.60	0.91
GO:0034765	regulation of ion transmembrane transport	0.207 %	-1.1203	0.64	0.92
GO:0034766	negative regulation of ion transmembrane transport	0.017 %	-1.7621	0.58	1.00
GO:1903960	negative regulation of anion transmembrane transport	0.017 %	-1.7621	0.58	1.00
GO:1903959	regulation of anion transmembrane transport	0.129 %	-1.2034	0.65	0.95
GO:2000116	regulation of cysteine-type endopeptidase activity	0.004 %	-2.1819	0.70	0.51
GO:0022898	regulation of transmembrane transporter activity	0.060 %	-1.1331	0.61	1.00
GO:0050790	regulation of catalytic activity	2.257 %	-1.7057	0.67	0.87
GO:0051338	regulation of transferase activity	0.522 %	-1.0391	0.71	0.75
GO:0051336	regulation of hydrolase activity	0.781 %	-1.0370	0.70	0.79
GO:0051348	negative regulation of transferase activity	0.151 %	-1.7621	0.71	0.81
GO:0051346	negative regulation of hydrolase activity	0.194 %	-1.7739	0.71	0.83
GO:0032412	regulation of ion transmembrane transporter activity	0.060 %	-1.1331	0.60	0.98
GO:0032410	negative regulation of transporter activity	0.017 %	-1.7621	0.54	0.95
GO:0032413	negative regulation of ion transmembrane transporter activity	0.017 %	-1.7621	0.53	1.00
GO:0044070	regulation of anion transport	0.164 %	-1.2004	0.65	0.77
GO:1903792	negative regulation of anion transport	0.017 %	-1.7621	0.58	0.95
GO:0044092	negative regulation of molecular function	1.070 %	-2.5190	0.69	0.79
GO:0043271	negative regulation of ion transport	0.026 %	-1.5202	0.58	0.98
GO:0043269	regulation of ion transport	0.302 %	-1.0153	0.65	0.85
GO:1990778	protein localization to cell periphery	0.022 %	-1.6685	0.89	0.21
GO:0048519	negative regulation of biological process	3.716 %	-1.4006	0.75	0.23
GO:0009913	epidermal cell differentiation	0.069 %	-1.2773	0.83	0.23

GO:0008544	epidermis development	0.069 %	-1.2159	0.94	0.50
GO:0060429	epithelium development	0.082 %	-1.1570	0.94	0.51
GO:0030855	epithelial cell differentiation	0.069 %	-1.2773	0.83	0.98
GO:0065009	regulation of molecular function	2.443 %	-1.3589	0.79	0.26
GO:0022611	dormancy process	0.142 %	-1.2700	0.95	0.26
GO:0007009	plasma membrane organization	0.022 %	-1.6326	0.82	0.27
GO:0072661	protein targeting to plasma membrane	0.013 %	-1.6685	0.75	0.66
GO:0072657	protein localization to membrane	0.259 %	-1.1767	0.72	0.65
GO:0072659	protein localization to plasma membrane	0.013 %	-1.6685	0.75	0.94
GO:0090002	establishment of protein localization to plasma membrane	0.013 %	-1.6685	0.75	1.00
GO:0006612	protein targeting to membrane	0.168 %	-1.2593	0.72	0.96
GO:0090150	establishment of protein localization to membrane	0.259 %	-1.1767	0.71	0.80
GO:0000919	cell plate assembly	0.052 %	-1.4006	0.78	0.29
GO:0032506	cytokinetic process	0.203 %	-1.1279	0.78	0.80
GO:1902410	mitotic cytokinetic process	0.203 %	-1.1279	0.78	0.99
GO:0000911	cytokinesis by cell plate formation	0.194 %	-1.1570	0.78	0.90
GO:0009920	cell plate formation involved in plant-type cell wall biogenesis	0.030 %	-1.5268	0.77	0.80
GO:0006820	anion transport	1.524 %	-1.4246	0.84	0.30
GO:0051246	regulation of protein metabolic process	2.123 %	-1.1635	0.70	0.30
GO:0043467	regulation of generation of precursor metabolites and energy	0.117 %	-1.1543	0.75	0.31
GO:0048583	regulation of response to stimulus	2.521 %	-1.1683	0.73	0.31
GO:0009719	response to endogenous stimulus	7.359 %	-1.3527	0.90	0.32
GO:0010030	positive regulation of seed germination	0.069 %	-1.4315	0.74	0.32
GO:0080050	regulation of seed development	0.129 %	-1.2961	0.71	0.62
GO:2000242	negative regulation of reproductive process	0.220 %	-1.0732	0.62	0.70
GO:0010646	regulation of cell communication	1.139 %	-2.1424	0.71	0.36
GO:2000241	regulation of reproductive process	1.092 %	-1.0129	0.74	0.37
GO:0023051	regulation of signaling	1.126 %	-2.1495	0.74	0.38
GO:0035556	intracellular signal transduction	3.112 %	-1.2277	0.62	0.55
GO:0010686	tetracyclic triterpenoid biosynthetic process	0.004 %	-1.8247	0.80	0.38
GO:0010685	tetracyclic triterpenoid metabolic process	0.004 %	-1.8247	0.80	0.79
GO:0010683	tricyclic triterpenoid metabolic process	0.013 %	-1.7506	0.79	0.84
GO:0019742	pentacyclic triterpenoid metabolic process	0.004 %	-1.6502	0.80	0.84
GO:0019745	pentacyclic triterpenoid biosynthetic process	0.004 %	-1.8247	0.80	0.81
GO:0016104	triterpenoid biosynthetic process	0.030 %	-1.5917	0.78	0.93
GO:0080003	thalianol metabolic process	0.009 %	-1.8671	0.79	0.87
GO:0006722	triterpenoid metabolic process	0.035 %	-1.4423	0.80	0.55
GO:0010263	tricyclic triterpenoid biosynthetic process	0.009 %	-1.8384	0.79	0.96
GO:0080167	response to karrikin	0.531 %	-1.2071	0.91	0.39
GO:0006468	protein phosphorylation	5.334 %	-1.0032	0.81	0.40
GO:0046903	secretion	0.501 %	-1.0172	0.81	0.40
GO:0009739	response to gibberellin	0.626 %	-2.4615	0.86	0.41
GO:0009755	hormone-mediated signaling pathway	3.617 %	-1.1410	0.58	0.67
GO:0009725	response to hormone	6.871 %	-1.5253	0.84	0.81
GO:0009723	response to ethylene	1.269 %	-1.1931	0.86	0.53
GO:0033993	response to lipid	3.267 %	-1.1887	0.85	0.58
GO:0051051	negative regulation of transport	0.026 %	-1.5202	0.61	0.41
GO:0032409	regulation of transporter activity	0.060 %	-1.1331	0.63	0.65
GO:0015692	lead ion transport	0.004 %	-1.4209	0.89	0.43
GO:0001101	response to acid chemical	5.011 %	-1.1431	0.87	0.45
GO:0046352	disaccharide catabolic process	0.035 %	-1.6592	0.81	0.45
GO:0044275	cellular carbohydrate catabolic process	0.298 %	-1.0432	0.88	0.64
GO:0009313	oligosaccharide catabolic process	0.039 %	-1.3583	0.84	0.69
GO:0005987	sucrose catabolic process	0.022 %	-1.7986	0.81	0.96
GO:0005985	sucrose metabolic process	0.164 %	-1.1681	0.81	0.81
GO:0043161	proteasome-mediated ubiquitin-dependent protein catabolic process	2.231 %	-1.1709	0.81	0.47
GO:0044248	cellular catabolic process	5.939 %	-1.1744	0.87	0.70
GO:0010498	proteasomal protein catabolic process	2.240 %	-1.1462	0.81	0.86
GO:0080168	abscisic acid transport	0.017 %	-1.3813	0.80	0.47
GO:0098656	anion transmembrane transport	0.902 %	-1.0891	0.82	0.73
GO:0015718	monocarboxylic acid transport	0.142 %	-1.2255	0.78	0.65
GO:0010332	response to gamma radiation	0.043 %	-1.5996	0.92	0.48
GO:0031570	DNA integrity checkpoint	0.060 %	-1.9488	0.57	0.48
GO:0051445	regulation of meiotic cell cycle	0.060 %	-1.4647	0.65	0.64
GO:0000075	cell cycle checkpoint	0.117 %	-1.6779	0.56	0.87
GO:0000077	DNA damage checkpoint	0.043 %	-1.9864	0.55	0.89
GO:0022402	cell cycle process	1.713 %	-1.2614	0.77	0.62
GO:0010564	regulation of cell cycle process	0.384 %	-1.1435	0.64	0.74
GO:0031122	cytoplasmic microtubule organization	0.216 %	-1.1154	0.81	0.48
GO:0043622	cortical microtubule organization	0.112 %	-1.1254	0.82	0.81
GO:0030865	cortical cytoskeleton organization	0.129 %	-1.1032	0.88	0.71
GO:0006996	organelle organization	6.439 %	-1.4099	0.87	0.48
GO:1905156	negative regulation of photosynthesis	0.073 %	-1.2354	0.62	0.49

GO:0042548	regulation of photosynthesis, light reaction	0.108 %	-1.1973	0.73	0.92
GO:0010205	photoinhibition	0.060 %	-1.2773	0.55	0.95
GO:0010109	regulation of photosynthesis	0.160 %	-1.0310	0.73	0.66
GO:0043155	negative regulation of photosynthesis, light reaction	0.060 %	-1.2773	0.62	0.97
GO:0046434	organophosphate catabolic process	0.112 %	-1.2923	0.87	0.49
GO:0043069	negative regulation of programmed cell death	0.082 %	-1.2034	0.61	0.49
GO:0060548	negative regulation of cell death	0.112 %	-1.0845	0.60	0.83
GO:0009247	glycolipid biosynthetic process	0.250 %	-1.4534	0.79	0.49
GO:0046467	membrane lipid biosynthetic process	0.371 %	-1.2885	0.78	0.91
GO:0006664	glycolipid metabolic process	0.306 %	-1.2159	0.78	0.98
GO:1903509	liposaccharide metabolic process	0.315 %	-1.1681	0.84	0.56

Biological Process (147) Tag Clouds

Scatterplot & Table Interactive Graph TreeMap

GROUP 22

Hide/show dispensable GO terms

Export results to text table (CSV)

Make R script for plotting

term ID	description	frequency	pin?	log_{10} p-value	uniqueness	dispensability
GO:0009624	response to nematode	0.306 %		-2.5487	0.95	0.00
GO:0032535	regulation of cellular component size	0.319 %		-3.1692	0.63	0.00
GO:0051494	*negative regulation of cytoskeleton organization*	*0.082 %*		*-1.4853*	*0.56*	*0.91*
GO:0032272	*negative regulation of protein polymerization*	*0.078 %*		*-1.5534*	*0.56*	*0.99*
GO:0030832	*regulation of actin filament length*	*0.181 %*		*-1.2267*	*0.52*	*0.98*
GO:0030835	*negative regulation of actin filament depolymerization*	*0.047 %*		*-1.5534*	*0.48*	*0.99*
GO:0030833	*regulation of actin filament polymerization*	*0.164 %*		*-1.2510*	*0.50*	*0.94*
GO:0030834	*regulation of actin filament depolymerization*	*0.065 %*		*-1.4981*	*0.50*	*0.92*
GO:0043244	*regulation of protein complex disassembly*	*0.099 %*		*-1.3254*	*0.56*	*0.88*
GO:0043241	*protein complex disassembly*	*0.246 %*		*-1.2404*	*0.66*	*0.98*
GO:0043242	*negative regulation of protein complex disassembly*	*0.052 %*		*-1.4853*	*0.53*	*0.93*
GO:0051261	*protein depolymerization*	*0.147 %*		*-1.3086*	*0.67*	*0.95*
GO:0051016	*barbed-end actin filament capping*	*0.017 %*		*-1.8823*	*0.50*	*0.79*
GO:0007015	*actin filament organization*	*0.315 %*		*-1.0160*	*0.67*	*0.95*
GO:0043624	*cellular protein complex disassembly*	*0.237 %*		*-1.2475*	*0.66*	*0.94*
GO:0008154	*actin polymerization or depolymerization*	*0.237 %*		*-1.1337*	*0.67*	*0.90*
GO:0008064	*regulation of actin polymerization or depolymerization*	*0.181 %*		*-1.2267*	*0.52*	*0.99*
GO:0051693	*actin filament capping*	*0.047 %*		*-1.5534*	*0.47*	*0.96*

GO:0032956	regulation of actin cytoskeleton organization	0.194 %	-1.2267	0.54	0.99
GO:1901880	negative regulation of protein depolymerization	0.052 %	-1.4853	0.53	0.99
GO:1901879	regulation of protein depolymerization	0.078 %	-1.4003	0.56	0.95
GO:0030042	actin filament depolymerization	0.117 %	-1.3852	0.61	0.85
GO:0030837	negative regulation of actin filament polymerization	0.069 %	-1.5534	0.48	0.90
GO:0008361	regulation of cell size	0.099 %	-2.3000	0.66	0.90
GO:0032984	macromolecular complex disassembly	0.268 %	-1.2370	0.68	0.96
GO:0050896	response to stimulus	25.943 %	-1.4016	0.99	0.00
GO:0006261	DNA-dependent DNA replication	0.419 %	-2.4082	0.72	0.04
GO:0042023	DNA endoreduplication	0.160 %	-2.5865	0.64	0.84
GO:0006275	regulation of DNA replication	0.125 %	-1.4791	0.62	0.82
GO:0090329	regulation of DNA-dependent DNA replication	0.095 %	-1.5389	0.62	0.95
GO:2000104	negative regulation of DNA-dependent DNA replication	0.039 %	-1.8368	0.59	0.98
GO:0044786	cell cycle DNA replication	0.181 %	-2.5810	0.64	0.86
GO:0051053	negative regulation of DNA metabolic process	0.086 %	-1.5684	0.59	0.57
GO:0032876	negative regulation of DNA endoreduplication	0.030 %	-1.8823	0.55	0.87
GO:0032875	regulation of DNA endoreduplication	0.086 %	-1.6346	0.57	0.93
GO:0051052	regulation of DNA metabolic process	0.380 %	-1.1912	0.62	0.64
GO:0008156	negative regulation of DNA replication	0.039 %	-1.8368	0.59	0.88
GO:0006260	DNA replication	0.665 %	-2.0168	0.73	0.68
GO:0090467	arginine import	0.004 %	-2.0575	0.84	0.06
GO:0042886	amide transport	0.220 %	-1.1418	0.90	0.51
GO:0006865	amino acid transport	0.419 %	-1.1672	0.82	0.76
GO:0006857	oligopeptide transport	0.155 %	-1.1473	0.90	0.66
GO:0015833	peptide transport	0.177 %	-1.1473	0.89	0.95
GO:1902023	L-arginine transport	0.004 %	-2.0575	0.84	0.86
GO:0043090	amino acid import	0.056 %	-1.3901	0.83	0.62
GO:0043092	L-amino acid import	0.017 %	-2.0575	0.83	0.80
GO:0043091	L-arginine import	0.004 %	-2.0575	0.84	0.91
GO:0051938	L-glutamate import	0.013 %	-2.1363	0.83	0.85
GO:0008283	cell proliferation	0.470 %	-1.6794	0.91	0.09
GO:0061062	regulation of nematode larval development	0.009 %	-2.0341	0.73	0.12
GO:2000026	regulation of multicellular organismal development	1.765 %	-1.2800	0.63	0.92
GO:2000024	regulation of leaf development	0.224 %	-1.3611	0.67	0.76
GO:2000038	regulation of stomatal complex development	0.039 %	-1.4917	0.71	0.65
GO:0048580	regulation of post-embryonic development	1.303 %	-1.5150	0.63	0.58
GO:0010374	stomatal complex development	0.220 %	-1.0323	0.83	0.65
GO:0002119	nematode larval development	0.009 %	-2.0341	0.86	1.00
GO:0009934	regulation of meristem structural organization	0.065 %	-1.2510	0.71	0.61
GO:1900056	negative regulation of leaf senescence	0.043 %	-1.6002	0.64	0.83
GO:1900055	regulation of leaf senescence	0.104 %	-1.5113	0.69	0.71
GO:0010212	response to ionizing radiation	0.129 %	-1.5248	0.95	0.16
GO:0010467	gene expression	18.304 %	-1.4710	0.87	0.17
GO:0010948	negative regulation of cell cycle process	0.117 %	-1.6346	0.63	0.18
GO:0022402	cell cycle process	1.713 %	-1.2331	0.78	0.66
GO:0045786	negative regulation of cell cycle	0.211 %	-1.4435	0.61	0.74
GO:0010564	regulation of cell cycle process	0.384 %	-1.1283	0.67	0.81
GO:0032970	regulation of actin filament-based process	0.194 %	-1.2267	0.63	0.19
GO:0018958	phenol-containing compound metabolic process	0.190 %	-1.4853	0.84	0.19
GO:0009735	response to cytokinin	1.010 %	-1.6706	0.93	0.19
GO:0009753	response to jasmonic acid	0.919 %	-1.1399	0.92	0.58
GO:0009739	response to gibberellin	0.626 %	-1.3993	0.92	0.52
GO:0009699	phenylpropanoid biosynthetic process	0.337 %	-1.5877	0.70	0.20
GO:0046189	phenol-containing compound biosynthetic process	0.104 %	-1.4853	0.81	0.84
GO:0044550	secondary metabolite biosynthetic process	1.183 %	-1.2673	0.76	0.80
GO:0009698	phenylpropanoid metabolic process	0.505 %	-1.3897	0.73	0.70
GO:0010023	proanthocyanidin biosynthetic process	0.035 %	-1.6919	0.73	0.77
GO:0065008	regulation of biological quality	5.991 %	-1.2867	0.75	0.21
GO:0010375	stomatal complex patterning	0.047 %	-1.4917	0.86	0.24
GO:2000037	regulation of stomatal complex patterning	0.022 %	-1.6085	0.73	0.63
GO:0051090	regulation of sequence-specific DNA binding transcription factor activity	0.052 %	-1.4268	0.69	0.25
GO:0044092	negative regulation of molecular function	1.070 %	-1.0905	0.78	0.60
GO:0071705	nitrogen compound transport	1.515 %	-1.5877	0.93	0.27
GO:0002164	larval development	0.009 %	-2.0341	0.87	0.29
GO:0007033	vacuole organization	0.186 %	-1.1077	0.81	0.29
GO:0048317	seed morphogenesis	0.026 %	-1.7829	0.85	0.30
GO:0050793	regulation of developmental process	2.577 %	-1.4514	0.68	0.32
GO:0051239	regulation of multicellular organismal process	1.972 %	-1.1398	0.69	0.33
GO:0045893	positive regulation of transcription, DNA-templated	1.864 %	-1.2907	0.53	0.35
GO:0032774	RNA biosynthetic process	11.813 %	-2.3856	0.67	0.83
GO:0010468	regulation of gene expression	12.706 %	-1.1176	0.58	0.80
GO:1902680	positive regulation of RNA biosynthetic process	1.869 %	-1.2907	0.53	0.97

GO:0031328	positive regulation of cellular biosynthetic process	2.102 %	-1.1730	0.56	0.97
GO:0031326	regulation of cellular biosynthetic process	12.339 %	-1.0842	0.52	0.87
GO:0031325	positive regulation of cellular metabolic process	2.741 %	-1.0650	0.59	0.95
GO:0051252	regulation of RNA metabolic process	11.463 %	-1.4557	0.51	0.67
GO:2001141	regulation of RNA biosynthetic process	11.282 %	-1.5007	0.49	0.88
GO:0019219	regulation of nucleobase-containing compound metabolic process	11.700 %	-1.3937	0.52	0.85
GO:0051254	positive regulation of RNA metabolic process	1.912 %	-1.2907	0.55	0.96
GO:0016070	RNA metabolic process	17.562 %	-2.1464	0.71	0.67
GO:2000112	regulation of cellular macromolecule biosynthetic process	12.003 %	-1.2560	0.51	0.79
GO:0045935	positive regulation of nucleobase-containing compound metabolic process	2.007 %	-1.2564	0.55	0.96
GO:0010628	positive regulation of gene expression	2.050 %	-1.2293	0.60	0.90
GO:0006355	regulation of transcription, DNA-templated	11.234 %	-1.5007	0.49	0.87
GO:0006351	transcription, DNA-templated	11.709 %	-2.3856	0.65	0.62
GO:1903508	positive regulation of nucleic acid-templated transcription	1.869 %	-1.2907	0.53	0.98
GO:1903506	regulation of nucleic acid-templated transcription	11.282 %	-1.5007	0.49	0.89
GO:0097659	nucleic acid-templated transcription	11.761 %	-2.3856	0.66	0.88
GO:0010604	positive regulation of macromolecule metabolic process	2.672 %	-1.0859	0.60	0.92
GO:0009891	positive regulation of biosynthetic process	2.145 %	-1.1618	0.58	0.89
GO:0009889	regulation of biosynthetic process	12.408 %	-1.0498	0.56	0.78
GO:0010556	regulation of macromolecule biosynthetic process	12.076 %	-1.2508	0.53	0.86
GO:0010557	positive regulation of macromolecule biosynthetic process	2.007 %	-1.2125	0.56	0.95
GO:0051173	positive regulation of nitrogen compound metabolic process	2.119 %	-1.1827	0.59	0.88
GO:0034654	nucleobase-containing compound biosynthetic process	13.056 %	-2.0353	0.68	0.81
GO:0051171	regulation of nitrogen compound metabolic process	12.430 %	-1.1511	0.58	0.76
GO:0051128	regulation of cellular component organization	1.467 %	-1.2534	0.66	0.35
GO:0022411	cellular component disassembly	0.324 %	-1.2200	0.78	0.35
GO:0010541	acropetal auxin transport	0.013 %	-1.4917	0.75	0.36
GO:0010540	basipetal auxin transport	0.078 %	-1.0180	0.72	0.70
GO:0019438	aromatic compound biosynthetic process	14.247 %	-2.7711	0.73	0.38
GO:0018130	heterocycle biosynthetic process	14.014 %	-2.3046	0.73	0.58
GO:1901362	organic cyclic compound biosynthetic process	14.769 %	-2.5991	0.75	0.55
GO:0009718	anthocyanin-containing compound biosynthetic process	0.121 %	-1.1257	0.80	0.38
GO:0010229	inflorescence development	0.104 %	-1.0042	0.84	0.39
GO:0015849	organic acid transport	0.682 %	-1.1672	0.84	0.41
GO:0042631	cellular response to water deprivation	0.129 %	-1.1418	0.88	0.41
GO:0010182	sugar mediated signaling pathway	0.147 %	-1.1337	0.69	0.53
GO:0009756	carbohydrate mediated signaling	0.147 %	-1.1337	0.69	0.94
GO:0071462	cellular response to water stimulus	0.129 %	-1.1418	0.88	0.70
GO:0043433	negative regulation of sequence-specific DNA binding transcription factor activity	0.022 %	-1.5684	0.65	0.45
GO:0090066	regulation of anatomical structure size	0.319 %	-3.1692	0.77	0.46
GO:0010332	response to gamma radiation	0.043 %	-1.5840	0.95	0.48
GO:0001101	response to acid chemical	5.011 %	-1.0874	0.93	0.48
GO:0009736	cytokinin-activated signaling pathway	0.328 %	-1.2475	0.68	0.48
GO:0071368	cellular response to cytokinin stimulus	0.337 %	-1.1672	0.88	0.80
GO:0030308	negative regulation of cell growth	0.043 %	-1.5840	0.61	0.48
GO:0051493	regulation of cytoskeleton organization	0.289 %	-1.1501	0.58	0.69
GO:0032271	regulation of protein polymerization	0.186 %	-1.2510	0.59	0.87
GO:0031333	negative regulation of protein complex assembly	0.078 %	-1.5534	0.57	0.56
GO:0045926	negative regulation of growth	0.121 %	-1.2510	0.73	0.61
GO:0010639	negative regulation of organelle organization	0.138 %	-1.3852	0.56	0.89
GO:0051129	negative regulation of cellular component organization	0.211 %	-1.1974	0.59	0.62
GO:0030041	actin filament polymerization	0.168 %	-1.2335	0.63	0.93
GO:0043254	regulation of protein complex assembly	0.298 %	-1.2510	0.58	0.81
GO:0009888	tissue development	2.326 %	-1.4227	0.89	0.49
GO:0048364	root development	1.886 %	-1.0359	0.81	0.49
GO:0022622	root system development	1.895 %	-1.0300	0.81	0.61
GO:0009960	endosperm development	0.134 %	-1.2924	0.83	0.50

Biological Process (224) Tag Clouds

Scatterplot & Table Interactive Graph TreeMap

GROUP 23

Tip: your resulting list of GO terms seems to be quite long. If you want to reduce it further, press the Back button in your browser and choose a different setting for the "allowed similarity" parameter.

Hide/show dispensable GO terms

Export results to text table (CSV)

Make R script for plotting

term ID	description	frequency	pin?	$\log_{10}$ p-value	uniqueness	dispensability
GO:0000003	reproduction	6.655 %		-3.2441	1.00	0.00
GO:0032260	response to jasmonic acid stimulus involved in jasmonic acid and ethylene-dependent systemic resistance	0.009 %		-2.4962	0.94	0.00
GO:0009861	*jasmonic acid and ethylene-dependent systemic resistance*	*0.052 %*		*-1.2223*	*0.95*	*0.58*
GO:0032501	multicellular organismal process	11.360 %		-1.7416	0.99	0.00
GO:0032502	developmental process	12.689 %		-1.7122	0.99	0.00
GO:0036290	protein trans-autophosphorylation	0.004 %		-4.6321	0.93	0.00
GO:0044699	single-organism process	41.709 %		-3.0239	0.99	0.00
GO:0048608	reproductive structure development	4.674 %		-3.8282	0.72	0.00
GO:0044707	*single-multicellular organism process*	*10.699 %*		*-2.0985*	*0.76*	*0.86*
GO:0044702	*single organism reproductive process*	*5.939 %*		*-3.2672*	*0.80*	*0.89*
GO:0003006	*developmental process involved in reproduction*	*5.611 %*		*-3.9683*	*0.83*	*0.85*
GO:0010214	*seed coat development*	*0.168 %*		*-1.5859*	*0.79*	*0.62*
GO:0080060	*integument development*	*0.039 %*		*-3.0940*	*0.80*	*0.53*
GO:0044767	*single-organism developmental process*	*12.123 %*		*-1.9325*	*0.75*	*0.82*
GO:0010154	*fruit development*	*2.443 %*		*-3.0597*	*0.74*	*0.86*
GO:0009555	*pollen development*	*1.230 %*		*-1.9714*	*0.78*	*0.54*

GO:0080001	mucilage extrusion from seed coat	0.022 %	-1.1942	0.82	0.51
GO:0007275	multicellular organism development	10.423 %	-2.1577	0.73	0.78
GO:0048440	carpel development	0.268 %	-1.1528	0.77	0.94
GO:0048481	plant ovule development	0.190 %	-1.4574	0.77	0.88
GO:0048827	phyllome development	1.921 %	-1.0734	0.76	0.69
GO:0048367	shoot system development	3.599 %	-1.5186	0.75	0.76
GO:0048359	mucilage metabolic process involved in seed coat development	0.086 %	-1.1374	0.78	0.58
GO:0048856	anatomical structure development	11.722 %	-1.8998	0.85	0.85
GO:0048316	seed development	2.305 %	-3.3413	0.73	0.85
GO:0009790	embryo development	1.683 %	-1.3979	0.78	0.57
GO:0009791	post-embryonic development	5.848 %	-3.1900	0.75	0.69
GO:0009793	embryo development ending in seed dormancy	1.584 %	-1.8353	0.74	0.92
GO:0022414	reproductive process	6.629 %	-3.2887	0.92	0.91
GO:0035670	plant-type ovary development	0.194 %	-1.4320	0.77	0.94
GO:0048229	gametophyte development	1.653 %	-1.4793	0.78	0.57
GO:0061458	reproductive system development	4.674 %	-3.8282	0.74	0.80
GO:0048731	system development	7.225 %	-2.3756	0.74	0.82
GO:0065007	biological regulation	28.554 %	-1.6154	0.99	0.00
GO:0009902	chloroplast relocation	0.129 %	-2.4349	0.85	0.03
GO:0019750	chloroplast localization	0.134 %	-2.4349	0.89	1.00
GO:0072594	establishment of protein localization to organelle	0.924 %	-1.0157	0.90	0.81
GO:0009903	chloroplast avoidance movement	0.112 %	-1.3022	0.85	0.99
GO:0009904	chloroplast accumulation movement	0.095 %	-1.4245	0.85	0.98
GO:0051656	establishment of organelle localization	0.276 %	-2.1771	0.89	0.91
GO:0051667	establishment of plastid localization	0.129 %	-2.4349	0.89	0.99
GO:0051644	plastid localization	0.134 %	-2.4349	0.89	0.86
GO:0051640	organelle localization	0.332 %	-1.9708	0.90	0.55
GO:0051649	establishment of localization in cell	2.408 %	-1.2465	0.89	0.72
GO:0019079	viral genome replication	0.026 %	-1.3904	0.96	0.03
GO:0019058	viral life cycle	0.030 %	-1.3796	0.96	0.70
GO:0080088	spermidine hydroxycinnamate conjugate biosynthetic process	0.009 %	-3.8238	0.86	0.04
GO:1901615	organic hydroxy compound metabolic process	1.265 %	-2.4853	0.93	0.05
GO:0006790	sulfur compound metabolic process	1.420 %	-1.1681	0.92	0.06
GO:0009812	flavonoid metabolic process	0.414 %	-1.9525	0.94	0.07
GO:0051782	negative regulation of cell division	0.022 %	-3.0940	0.80	0.09
GO:0051302	regulation of cell division	0.199 %	-1.5962	0.80	0.56
GO:2000603	regulation of secondary growth	0.009 %	-1.2080	0.87	0.12
GO:2000605	positive regulation of secondary growth	0.004 %	-1.2296	0.87	0.87
GO:0080117	secondary growth	0.022 %	-1.0035	0.89	0.95
GO:0055088	lipid homeostasis	0.199 %	-1.3391	0.93	0.12
GO:0030104	water homeostasis	0.052 %	-1.0077	0.93	0.58
GO:0010268	brassinosteroid homeostasis	0.138 %	-1.4488	0.93	0.62
GO:0010114	response to red light	0.259 %	-1.3386	0.92	0.13
GO:0010161	red light signaling pathway	0.035 %	-1.2447	0.80	0.78
GO:0009637	response to blue light	0.341 %	-1.1222	0.92	0.64
GO:0009638	phototropism	0.069 %	-1.0876	0.92	0.53
GO:0009785	blue light signaling pathway	0.078 %	-1.0527	0.79	0.83
GO:0071482	cellular response to light stimulus	0.561 %	-1.2207	0.88	0.63
GO:0071478	cellular response to radiation	0.583 %	-1.2207	0.88	0.91
GO:0071491	cellular response to red light	0.043 %	-1.2151	0.90	0.79
GO:0007263	nitric oxide mediated signal transduction	0.009 %	-1.6982	0.85	0.13
GO:0019748	secondary metabolic process	1.938 %	-2.9183	0.76	0.15
GO:0044272	sulfur compound biosynthetic process	0.699 %	-2.0453	0.85	0.16
GO:0019760	glucosinolate metabolic process	0.514 %	-1.5623	0.58	0.91
GO:0019758	glycosinolate biosynthetic process	0.194 %	-1.9485	0.60	1.00
GO:0019757	glycosinolate metabolic process	0.514 %	-1.5623	0.58	0.99
GO:0019761	glucosinolate biosynthetic process	0.194 %	-1.9485	0.60	0.75
GO:0046499	S-adenosylmethioninamine metabolic process	0.017 %	-1.7214	0.71	0.71
GO:0010439	regulation of glucosinolate biosynthetic process	0.026 %	-1.3589	0.61	0.86
GO:0006557	S-adenosylmethioninamine biosynthetic process	0.017 %	-1.7214	0.69	0.62
GO:0048519	negative regulation of biological process	3.716 %	-1.1268	0.93	0.19
GO:0031221	arabinan metabolic process	0.009 %	-1.3391	0.82	0.21
GO:0045730	respiratory burst	0.013 %	-1.3022	0.83	0.21
GO:0044710	single-organism metabolic process	18.390 %	-1.6446	0.83	0.21
GO:0043446	cellular alkane metabolic process	0.017 %	-1.7214	0.81	0.22
GO:0043447	alkane biosynthetic process	0.017 %	-1.7214	0.78	0.22
GO:0009828	plant-type cell wall loosening	0.022 %	-1.7999	0.95	0.23
GO:0080170	hydrogen peroxide transmembrane transport	0.022 %	-1.2603	0.93	0.25
GO:1902123	(-)-pinoresinol catabolic process	0.081 %	-2.6720	0.56	0.25
GO:0044724	single-organism carbohydrate catabolic process	0.432 %	-1.1222	0.73	0.65
GO:0010115	regulation of abscisic acid biosynthetic process	0.030 %	-1.2011	0.57	0.68
GO:0034312	diol biosynthetic process	0.004 %	-1.9337	0.68	0.62

GO:0034311	diol metabolic process	0.004 %	-1.9337	0.71	0.54
GO:0034310	primary alcohol catabolic process	0.128 %	-2.1958	0.61	0.97
GO:0034309	primary alcohol biosynthetic process	0.009 %	-2.1958	0.68	0.52
GO:0034308	primary alcohol metabolic process	0.052 %	-1.1744	0.68	0.62
GO:1902124	(+)-pinoresinol metabolic process	1.218 %	-2.1958	0.50	1.92
GO:1902127	(-)-lariciresinol metabolic process	1.258 %	-2.3714	0.49	1.47
GO:1902128	(-)-lariciresinol catabolic process	0.087 %	-2.4962	0.56	1.48
GO:1902125	(+)-pinoresinol catabolic process	0.087 %	-2.1958	0.56	1.48
GO:0016131	brassinosteroid metabolic process	0.177 %	-2.5780	0.64	0.64
GO:1902131	(+)-lariciresinol catabolic process	0.087 %	-2.1958	0.52	1.48
GO:1902132	(+)-lariciresinol biosynthetic process	0.510 %	-2.1958	0.46	1.75
GO:1902129	(-)-lariciresinol biosynthetic process	0.542 %	-2.3714	0.50	1.76
GO:0016128	phytosteroid metabolic process	0.194 %	-2.5780	0.67	0.86
GO:1902130	(+)-lariciresinol metabolic process	1.007 %	-2.1958	0.46	1.88
GO:1901616	organic hydroxy compound catabolic process	0.121 %	-1.0207	0.77	0.66
GO:0044550	secondary metabolite biosynthetic process	1.183 %	-2.0371	0.64	0.67
GO:1901598	(-)-pinoresinol metabolic process	0.009 %	-1.8961	0.63	1.02
GO:0019336	phenol-containing compound catabolic process	0.030 %	-2.1958	0.73	0.88
GO:0009698	phenylpropanoid metabolic process	0.505 %	-1.4805	0.67	0.80
GO:1902138	(-)-secoisolariciresinol biosynthetic process	0.083 %	-2.1958	0.53	1.47
GO:1902133	(+)-secoisolariciresinol metabolic process	0.029 %	-2.4962	0.57	1.11
GO:1902136	(-)-secoisolariciresinol metabolic process	0.124 %	-2.1958	0.53	1.52
GO:1902135	(+)-secoisolariciresinol biosynthetic process	0.018 %	-2.4962	0.57	1.20
GO:0006066	alcohol metabolic process	0.505 %	-1.4867	0.64	0.74
GO:0043455	regulation of secondary metabolic process	0.173 %	-1.5893	0.68	0.72
GO:0018958	phenol-containing compound metabolic process	0.190 %	-1.1555	0.77	0.75
GO:1901503	ether biosynthetic process	1.458 %	-2.1958	0.65	1.37
GO:1901502	ether catabolic process	0.474 %	-2.1958	0.67	0.57
GO:0046189	phenol-containing compound biosynthetic process	0.104 %	-1.1555	0.72	0.89
GO:0046165	alcohol biosynthetic process	0.250 %	-1.1680	0.62	0.78
GO:0046173	polyol biosynthetic process	0.082 %	-1.2684	0.64	0.80
GO:0009806	lignan metabolic process	0.022 %	-1.7721	0.71	0.68
GO:0009805	coumarin biosynthetic process	0.013 %	-1.6352	0.69	0.65
GO:0009808	lignin metabolic process	0.285 %	-1.2006	0.66	0.81
GO:0009807	lignan biosynthetic process	0.022 %	-1.7721	0.64	1.09
GO:0046164	alcohol catabolic process	0.082 %	-1.0928	0.63	0.97
GO:0009804	coumarin metabolic process	0.017 %	-1.3691	0.71	0.67
GO:0046273	lignan catabolic process	0.468 %	-2.1958	0.55	1.73
GO:0042445	hormone metabolic process	0.695 %	-2.2241	0.89	0.79
GO:0006979	response to oxidative stress	1.908 %	-1.9021	0.94	0.25
GO:0010155	regulation of proton transport	0.030 %	-1.7999	0.79	0.26
GO:0032410	negative regulation of transporter activity	0.017 %	-1.4128	0.83	0.95
GO:0032413	negative regulation of ion transmembrane transporter activity	0.017 %	-1.4128	0.83	1.00
GO:1903792	negative regulation of anion transport	0.017 %	-1.4128	0.83	0.92
GO:0034763	negative regulation of transmembrane transport	0.017 %	-1.4128	0.83	0.95
GO:0010360	negative regulation of anion channel activity	0.017 %	-1.4128	0.83	0.96
GO:0010362	negative regulation of anion channel activity by blue light	0.009 %	-1.7721	0.84	0.66
GO:0010361	regulation of anion channel activity by blue light	0.009 %	-1.7721	0.86	0.82
GO:0034766	negative regulation of ion transmembrane transport	0.017 %	-1.4128	0.83	1.00
GO:1903960	negative regulation of anion transmembrane transport	0.017 %	-1.4128	0.83	1.00
GO:0051051	negative regulation of transport	0.026 %	-1.1744	0.86	0.62
GO:0043271	negative regulation of ion transport	0.026 %	-1.1744	0.83	0.98
GO:0018904	ether metabolic process	0.142 %	-1.1145	0.75	0.26
GO:0030522	intracellular receptor signaling pathway	0.078 %	-1.0527	0.82	0.27
GO:0008202	steroid metabolic process	0.427 %	-1.5791	0.76	0.29
GO:0009308	amine metabolic process	0.522 %	-3.2748	0.86	0.31
GO:0007569	cell aging	0.022 %	-1.3296	0.80	0.32
GO:0042221	response to chemical	12.434 %	-1.0593	0.94	0.33
GO:0006949	syncytium formation	0.043 %	-1.2080	0.80	0.35
GO:0006560	proline metabolic process	0.065 %	-2.4450	0.70	0.36
GO:0043650	dicarboxylic acid biosynthetic process	0.129 %	-1.1315	0.70	0.73
GO:0009065	glutamine family amino acid catabolic process	0.043 %	-1.2011	0.67	0.74
GO:0009064	glutamine family amino acid metabolic process	0.367 %	-1.3509	0.67	0.66
GO:0009084	glutamine family amino acid biosynthetic process	0.147 %	-1.8748	0.66	0.81
GO:1901607	alpha-amino acid biosynthetic process	0.833 %	-1.0217	0.63	0.82
GO:0006537	glutamate biosynthetic process	0.017 %	-1.4128	0.70	0.70
GO:0006561	proline biosynthetic process	0.043 %	-1.1035	0.68	0.88
GO:0006562	proline catabolic process	0.013 %	-2.2748	0.69	0.69
GO:0046777	protein autophosphorylation	0.604 %	-1.1407	0.91	0.37

GO:0016310	phosphorylation	6.966 %	-1.1234	0.90	0.54
GO:0051017	actin filament bundle assembly	0.086 %	-1.2603	0.83	0.37
GO:0061572	actin filament bundle organization	0.086 %	-1.2603	0.83	0.81
GO:2000122	negative regulation of stomatal complex development	0.022 %	-2.1291	0.76	0.38
GO:0051241	negative regulation of multicellular organismal process	0.445 %	-1.0683	0.75	0.56
GO:0048581	negative regulation of post-embryonic development	0.354 %	-1.2289	0.72	0.77
GO:0010103	stomatal complex morphogenesis	0.125 %	-1.0673	0.79	0.89
GO:0010052	guard cell differentiation	0.082 %	-1.4883	0.75	0.79
GO:2000038	regulation of stomatal complex development	0.039 %	-1.1617	0.78	0.82
GO:0090344	negative regulation of cell aging	0.004 %	-2.0204	0.76	0.57
GO:0090342	regulation of cell aging	0.017 %	-1.3391	0.77	0.90
GO:0010686	tetracyclic triterpenoid biosynthetic process	0.004 %	-1.4747	0.75	0.38
GO:0010685	tetracyclic triterpenoid metabolic process	0.004 %	-1.4747	0.78	0.79
GO:0010683	tricyclic triterpenoid metabolic process	0.013 %	-1.4014	0.76	0.84
GO:0019742	pentacyclic triterpenoid metabolic process	0.004 %	-1.3022	0.78	0.84
GO:0019745	pentacyclic triterpenoid biosynthetic process	0.004 %	-1.4747	0.75	0.81
GO:0016104	triterpenoid biosynthetic process	0.030 %	-1.2447	0.72	0.93
GO:0080003	thalianol metabolic process	0.009 %	-1.5168	0.77	0.87
GO:0006722	triterpenoid metabolic process	0.035 %	-1.0981	0.77	0.55
GO:0010263	tricyclic triterpenoid biosynthetic process	0.009 %	-1.4883	0.74	0.96
GO:0072351	tricarboxylic acid biosynthetic process	0.022 %	-2.3714	0.73	0.38
GO:0050789	regulation of biological process	24.333 %	-1.1910	0.92	0.38
GO:0009611	response to wounding	0.816 %	-1.0587	0.94	0.38
GO:0010232	vascular transport	0.065 %	-1.1201	0.79	0.38
GO:0044765	single-organism transport	5.287 %	-1.0542	0.78	0.53
GO:1902578	single-organism localization	5.481 %	-1.0037	0.79	0.63
GO:0010233	phloem transport	0.065 %	-1.1201	0.79	0.38
GO:0009739	response to gibberellin	0.626 %	-1.4939	0.93	0.39
GO:0009735	response to cytokinin	1.010 %	-1.0603	0.93	0.52
GO:0009269	response to desiccation	0.073 %	-1.0527	0.92	0.73
GO:0009414	response to water deprivation	1.390 %	-1.0844	0.90	0.61
GO:0009415	response to water	1.416 %	-1.0610	0.90	0.74
GO:0006576	cellular biogenic amine metabolic process	0.255 %	-3.1896	0.81	0.40
GO:0030418	nicotianamine biosynthetic process	0.022 %	-2.3714	0.67	0.80
GO:0030417	nicotianamine metabolic process	0.022 %	-2.3714	0.70	0.80
GO:0044106	cellular amine metabolic process	0.306 %	-2.9308	0.82	0.89
GO:0008295	spermidine biosynthetic process	0.039 %	-1.4365	0.80	0.91
GO:0006597	spermine biosynthetic process	0.026 %	-1.6551	0.80	0.88
GO:0006595	polyamine metabolic process	0.095 %	-2.4979	0.82	0.89
GO:0006596	polyamine biosynthetic process	0.065 %	-1.2935	0.80	0.94
GO:0008215	spermine metabolic process	0.035 %	-1.6551	0.82	0.90
GO:0008216	spermidine metabolic process	0.056 %	-2.9510	0.82	0.86
GO:0009309	amine biosynthetic process	0.186 %	-2.3578	0.80	0.85
GO:0042401	cellular biogenic amine biosynthetic process	0.186 %	-2.3578	0.79	0.94
GO:0080190	lateral growth	0.022 %	-1.0035	0.90	0.40
GO:0031222	arabinan catabolic process	0.004 %	-1.5168	0.79	0.41
GO:0045493	xylan catabolic process	0.017 %	-1.1555	0.88	0.52
GO:1900000	regulation of anthocyanin catabolic process	0.004 %	-1.9749	0.73	0.41
GO:0016139	glycoside catabolic process	0.009 %	-1.3112	0.76	0.89
GO:0031537	regulation of anthocyanin metabolic process	0.095 %	-1.0207	0.75	0.74
GO:0046149	pigment catabolic process	0.082 %	-1.0824	0.80	0.56
GO:0009963	positive regulation of flavonoid biosynthetic process	0.039 %	-1.5168	0.85	0.61
GO:0046284	anthocyanin-containing compound catabolic process	0.004 %	-1.9749	0.76	1.00
GO:0046275	flavonoid catabolic process	0.004 %	-1.9749	0.90	0.54
GO:0016137	glycoside metabolic process	0.017 %	-1.1744	0.80	0.41
GO:1901657	glycosyl compound metabolic process	1.912 %	-1.0497	0.73	0.52
GO:0071732	cellular response to nitric oxide	0.086 %	-1.0163	0.90	0.42
GO:0044712	single-organism catabolic process	2.676 %	-1.6115	0.72	0.43
GO:0010207	photosystem II assembly	0.086 %	-1.2151	0.92	0.44
GO:0044711	single-organism biosynthetic process	7.549 %	-1.4029	0.71	0.45
GO:0072350	tricarboxylic acid metabolic process	0.259 %	-1.3022	0.72	0.45
GO:0007568	aging	0.544 %	-1.3679	0.82	0.45
GO:0009744	response to sucrose	0.237 %	-1.3008	0.93	0.46
GO:0034285	response to disaccharide	0.242 %	-1.3008	0.93	0.87
GO:1901700	response to oxygen-containing compound	6.504 %	-1.1893	0.92	0.47
GO:0010817	regulation of hormone levels	1.109 %	-1.2879	0.93	0.49
GO:0009743	response to carbohydrate	0.518 %	-1.4905	0.93	0.49

Biological Process (106) Tag Clouds

Scatterplot & Table Interactive Graph TreeMap

GROUP 24

Hide/show dispensable GO terms

Export results to text table (CSV)

Make R script for plotting

term ID	description	frequency	pin?	log_{10} p-value	uniqueness	dispensability
GO:0010411	xyloglucan metabolic process	0.259 %		-2.2642	0.88	0.00
GO:0010383	*cell wall polysaccharide metabolic process*	*0.518 %*		*-1.3226*	*0.88*	*0.93*
GO:0010410	*hemicellulose metabolic process*	*0.423 %*		*-1.5954*	*0.88*	*0.92*
GO:0044036	*cell wall macromolecule metabolic process*	*0.665 %*		*-1.1798*	*0.89*	*0.61*
GO:0048364	root development	1.886 %		-2.2506	0.61	0.00
GO:0048528	*post-embryonic root development*	*0.501 %*		*-1.6120*	*0.62*	*0.93*
GO:0048527	*lateral root development*	*0.457 %*		*-1.7867*	*0.62*	*0.92*
GO:0099402	*plant organ development*	*3.763 %*		*-1.7557*	*0.63*	*0.67*
GO:1905392	*plant organ morphogenesis*	*1.485 %*		*-1.4864*	*0.62*	*0.77*
GO:0022622	*root system development*	*1.895 %*		*-2.2370*	*0.65*	*0.61*
GO:0010102	*lateral root morphogenesis*	*0.229 %*		*-2.3492*	*0.63*	*0.77*
GO:0010101	*post-embryonic root morphogenesis*	*0.233 %*		*-2.3492*	*0.62*	*0.90*
GO:0009791	*post-embryonic development*	*5.848 %*		*-1.1812*	*0.65*	*0.59*
GO:0090697	*post-embryonic plant organ morphogenesis*	*0.419 %*		*-1.8165*	*0.61*	*0.86*
GO:0090696	*post-embryonic plant organ development*	*0.686 %*		*-1.3306*	*0.64*	*0.71*
GO:0010015	*root morphogenesis*	*0.967 %*		*-1.5035*	*0.61*	*0.90*
GO:0009812	flavonoid metabolic process	0.414 %		-1.1727	0.94	0.06
GO:0046717	acid secretion	0.026 %		-2.0574	0.77	0.08
GO:0080149	sucrose induced translational repression	0.004 %		-2.0118	0.80	0.09

GO:0032055	negative regulation of translation in response to stress	0.004 %	-2.0118	0.80	1.00
GO:0019048	modulation by virus of host morphology or physiology	0.017 %	-1.4609	0.90	0.10
GO:0009813	flavonoid biosynthetic process	0.332 %	-1.4181	0.90	0.14
GO:0046246	terpene biosynthetic process	0.043 %	-1.3385	0.79	0.17
GO:0042214	terpene metabolic process	0.065 %	-1.1559	0.82	0.63
GO:2000072	regulation of defense response to fungus, incompatible interaction	0.013 %	-1.8368	0.87	0.18
GO:1900150	regulation of defense response to fungus	0.073 %	-1.2169	0.86	0.72
GO:0046439	L-cysteine metabolic process	0.017 %	-1.9707	0.78	0.24
GO:0006561	proline biosynthetic process	0.043 %	-1.1392	0.75	0.74
GO:0006560	proline metabolic process	0.065 %	-1.1079	0.77	0.53
GO:0019450	L-cysteine catabolic process to pyruvate	0.004 %	-1.9707	0.79	0.71
GO:0019448	L-cysteine catabolic process	0.004 %	-1.9707	0.79	0.89
GO:0009734	auxin-activated signaling pathway	0.868 %	-1.9212	0.71	0.24
GO:0009753	response to jasmonic acid	0.919 %	-1.2261	0.91	0.57
GO:0009733	response to auxin	1.709 %	-1.4395	0.91	0.57
GO:0009723	response to ethylene	1.269 %	-1.2611	0.91	0.59
GO:0071365	cellular response to auxin stimulus	0.932 %	-1.7178	0.87	0.85
GO:0010033	response to organic substance	8.580 %	-1.2218	0.91	0.51
GO:0048533	sporocyte differentiation	0.004 %	-1.3297	0.77	0.26
GO:0033238	regulation of cellular amine metabolic process	0.060 %	-1.3128	0.81	0.28
GO:0072594	establishment of protein localization to organelle	0.924 %	-1.0788	0.80	0.29
GO:0006606	protein import into nucleus	0.242 %	-1.0103	0.70	0.82
GO:1902593	single-organism nuclear import	0.242 %	-1.0103	0.71	0.99
GO:0033365	protein localization to organelle	1.148 %	-1.0240	0.80	0.84
GO:0044744	protein targeting to nucleus	0.246 %	-1.0103	0.78	0.98
GO:0002164	larval development	0.009 %	-1.7350	0.75	0.30
GO:0061062	regulation of nematode larval development	0.009 %	-1.7350	0.68	0.30
GO:0002119	nematode larval development	0.009 %	-1.7350	0.74	1.00
GO:0015669	gas transport	0.009 %	-1.7828	0.79	0.32
GO:0015670	carbon dioxide transport	0.014 %	-2.0118	0.77	0.33
GO:0010565	regulation of cellular ketone metabolic process	0.276 %	-1.5611	0.70	0.33
GO:0006521	regulation of cellular amino acid metabolic process	0.060 %	-1.4730	0.70	0.80
GO:0010115	regulation of abscisic acid biosynthetic process	0.030 %	-1.2371	0.70	0.76
GO:0009688	abscisic acid biosynthetic process	0.091 %	-1.0346	0.75	0.93
GO:0006868	glutamine transport	0.026 %	-2.0574	0.72	0.34
GO:0015804	neutral amino acid transport	0.086 %	-1.3952	0.70	0.77
GO:0015851	nucleobase transport	0.108 %	-1.5840	0.71	0.57
GO:0015837	amine transport	0.043 %	-2.0118	0.80	0.52
GO:0010585	glutamine secretion	0.004 %	-2.0574	0.71	0.77
GO:0032890	regulation of organic acid transport	0.030 %	-2.0118	0.68	0.61
GO:0032973	amino acid export	0.082 %	-1.5248	0.70	0.72
GO:0051955	regulation of amino acid transport	0.030 %	-2.0118	0.65	1.00
GO:0080143	regulation of amino acid export	0.030 %	-2.0118	0.65	0.67
GO:0051952	regulation of amine transport	0.030 %	-2.0118	0.71	0.96
GO:0019755	one-carbon compound transport	0.035 %	-1.9331	0.76	0.34
GO:0009785	blue light signaling pathway	0.078 %	-1.0883	0.78	0.37
GO:0030522	intracellular receptor signaling pathway	0.078 %	-1.0883	0.78	0.37
GO:0010087	phloem or xylem histogenesis	0.272 %	-1.3011	0.77	0.38
GO:0010067	procambium histogenesis	0.039 %	-1.0788	0.69	0.53
GO:0010065	primary meristem tissue development	0.043 %	-1.0103	0.69	0.86
GO:0009960	endosperm development	0.134 %	-1.0025	0.68	0.59
GO:0010500	transmitting tissue development	0.017 %	-1.6528	0.70	0.39
GO:0010480	microsporocyte differentiation	0.244 %	-1.3297	0.59	0.93
GO:0048653	anther development	0.224 %	-1.5372	0.63	0.67
GO:0048466	androecium development	0.345 %	-1.1851	0.64	0.85
GO:0048438	floral whorl development	0.669 %	-1.1870	0.64	0.68
GO:0048443	stamen development	0.345 %	-1.1851	0.62	0.97
GO:0051171	regulation of nitrogen compound metabolic process	12.430 %	-1.4252	0.74	0.41
GO:0010468	regulation of gene expression	12.706 %	-1.3727	0.74	0.78
GO:0031323	regulation of cellular metabolic process	13.906 %	-1.1706	0.74	0.80
GO:0031326	regulation of cellular biosynthetic process	12.339 %	-1.3206	0.72	0.78
GO:2001141	regulation of RNA biosynthetic process	11.282 %	-1.0216	0.71	0.88
GO:2000112	regulation of cellular macromolecule biosynthetic process	12.003 %	-1.1530	0.71	0.87
GO:0006355	regulation of transcription, DNA-templated	11.234 %	-1.0216	0.71	0.85
GO:0019222	regulation of metabolic process	14.847 %	-1.2650	0.80	0.54
GO:1903506	regulation of nucleic acid-templated transcription	11.282 %	-1.0216	0.70	0.89
GO:0009889	regulation of biosynthetic process	12.408 %	-1.0809	0.74	0.78
GO:0010556	regulation of macromolecule biosynthetic process	12.076 %	-1.1464	0.72	0.87
GO:0010051	xylem and phloem pattern formation	0.259 %	-1.1176	0.72	0.41
GO:0010588	cotyledon vascular tissue pattern formation	0.060 %	-1.0223	0.63	0.76
GO:0071705	nitrogen compound transport	1.515 %	-1.0479	0.82	0.42
GO:0044765	single-organism transport	5.287 %	-1.1796	0.67	0.53

GO:0071702	organic substance transport	5.585 %	-1.1013	0.80	0.65
GO:1902578	single-organism localization	5.481 %	-1.1267	0.72	0.63
GO:0090698	post-embryonic plant morphogenesis	0.716 %	-2.1323	0.73	0.42
GO:0010223	secondary shoot formation	0.112 %	-1.0305	0.67	0.79
GO:1905393	plant organ formation	0.427 %	-1.0351	0.73	0.62
GO:0010346	shoot axis formation	0.112 %	-1.0305	0.67	0.80
GO:0042180	cellular ketone metabolic process	0.470 %	-1.3816	0.79	0.43
GO:0048359	mucilage metabolic process involved in seed coat development	0.086 %	-1.1732	0.67	0.44
GO:0009845	seed germination	0.600 %	-2.0692	0.67	0.45
GO:0090351	seedling development	0.660 %	-1.9684	0.67	0.53
GO:0080001	mucilage extrusion from seed coat	0.022 %	-1.2302	0.71	0.74
GO:0043555	regulation of translation in response to stress	0.004 %	-2.0118	0.81	0.48
GO:0015853	adenine transport	0.017 %	-1.9707	0.73	0.49
GO:0006863	purine nucleobase transport	0.095 %	-1.5840	0.71	0.87
GO:0015854	guanine transport	0.017 %	-1.9707	0.73	0.80

Biological Process (166) Tag Clouds

Scatterplot & Table Interactive Graph TreeMap

GROUP 25

Tip: your resulting list of GO terms seems to be quite long. If you want to reduce it further, press the Back button in your browser and choose a different setting for the "allowed similarity" parameter.

Hide/show dispensable GO terms

Export results to text table (CSV)

Make R script for plotting

term ID	description	frequency	pin?	$\log_{10}$ p-value	uniqueness	dispensability
GO:0000003	reproduction	6.655 %		-1.3173	1.00	0.00
GO:0007623	circadian rhythm	0.539 %		-1.1114	0.99	0.00
GO:0009744	response to sucrose	0.237 %		-2.8290	0.88	0.00
GO:0034285	*response to disaccharide*	*0.242 %*		*-2.8290*	*0.88*	*0.87*
GO:0010026	trichome differentiation	0.341 %		-5.5289	0.62	0.00
GO:0045168	*cell-cell signaling involved in cell fate commitment*	*0.168 %*		*-3.9467*	*0.63*	*0.76*
GO:0045165	*cell fate commitment*	*0.341 %*		*-1.7341*	*0.66*	*0.65*
GO:0003002	*regionalization*	*0.617 %*		*-1.4143*	*0.65*	*0.72*
GO:0010454	*negative regulation of cell fate commitment*	*0.022 %*		*-4.6846*	*0.59*	*0.91*
GO:0010453	*regulation of cell fate commitment*	*0.047 %*		*-3.2889*	*0.60*	*0.81*
GO:0090558	*plant epidermis development*	*1.001 %*		*-2.6738*	*0.69*	*0.71*
GO:0048581	*negative regulation of post-embryonic development*	*0.354 %*		*-1.7784*	*0.55*	*0.80*
GO:0048586	*regulation of long-day photoperiodism, flowering*	*0.086 %*		*-1.0723*	*0.53*	*0.90*
GO:0030154	*cell differentiation*	*3.332 %*		*-1.0501*	*0.59*	*0.78*
GO:0048579	*negative regulation of long-day photoperiodism, flowering*	*0.039 %*		*-1.4158*	*0.54*	*0.66*
GO:0010160	*formation of animal organ boundary*	*0.055 %*		*-1.1637*	*0.67*	*0.77*

GO:0045596	negative regulation of cell differentiation	0.052 %	-2.3528	0.58	0.75
GO:0048629	trichome patterning	0.030 %	-3.9467	0.61	0.78
GO:0010090	trichome morphogenesis	0.246 %	-3.7553	0.60	0.80
GO:0010089	xylem development	0.151 %	-1.6584	0.73	0.60
GO:0010091	trichome branching	0.121 %	-3.7824	0.62	0.89
GO:0010087	phloem or xylem histogenesis	0.272 %	-1.0048	0.72	0.63
GO:1902182	shoot apical meristem development	0.047 %	-1.3055	0.74	0.55
GO:0048468	cell development	1.217 %	-2.1254	0.62	0.65
GO:1900033	negative regulation of trichome patterning	0.022 %	-4.6846	0.52	0.65
GO:0000904	cell morphogenesis involved in differentiation	0.880 %	-1.5536	0.60	0.81
GO:1900032	regulation of trichome patterning	0.022 %	-4.6846	0.54	0.96
GO:0090626	plant epidermis morphogenesis	0.367 %	-3.3340	0.68	0.81
GO:0010492	maintenance of shoot apical meristem identity	0.056 %	-1.1724	0.70	0.61
GO:0023052	signaling	8.908 %	-1.4535	0.99	0.00
GO:0032502	developmental process	12.689 %	-1.6463	0.99	0.00
GO:0048511	rhythmic process	0.600 %	-1.0983	0.99	0.00
GO:0017183	peptidyl-diphthamide biosynthetic process from peptidyl-histidine	0.013 %	-1.8873	0.92	0.03
GO:0017182	peptidyl-diphthamide metabolic process	0.013 %	-1.8873	0.92	0.83
GO:0007154	cell communication	9.698 %	-1.6333	0.95	0.05
GO:0031222	arabinan catabolic process	0.004 %	-3.0859	0.82	0.06
GO:0044724	single-organism carbohydrate catabolic process	0.432 %	-1.6188	0.78	0.62
GO:0010383	cell wall polysaccharide metabolic process	0.518 %	-1.0250	0.87	0.93
GO:0010410	hemicellulose metabolic process	0.423 %	-1.2844	0.87	0.89
GO:0045493	xylan catabolic process	0.017 %	-2.3528	0.86	0.52
GO:0045491	xylan metabolic process	0.173 %	-1.6481	0.88	0.72
GO:0006850	mitochondrial pyruvate transport	0.017 %	-1.9958	0.90	0.07
GO:0006848	pyruvate transport	0.026 %	-1.7633	0.90	0.77
GO:0000959	mitochondrial RNA metabolic process	0.324 %	-1.0523	0.92	0.09
GO:0031539	positive regulation of anthocyanin metabolic process	0.022 %	-1.6396	0.79	0.15
GO:0046283	anthocyanin-containing compound metabolic process	0.190 %	-1.2343	0.85	0.74
GO:0006083	acetate metabolic process	0.009 %	-1.4842	0.85	0.16
GO:0001666	response to hypoxia	0.250 %	-2.1469	0.88	0.17
GO:0036293	response to decreased oxygen levels	0.281 %	-2.0540	0.88	0.99
GO:0036294	cellular response to decreased oxygen levels	0.129 %	-1.8183	0.82	0.99
GO:0071456	cellular response to hypoxia	0.117 %	-1.8986	0.82	0.92
GO:0071453	cellular response to oxygen levels	0.129 %	-1.8183	0.83	0.92
GO:0008037	cell recognition	0.220 %	-1.0108	0.85	0.19
GO:0007267	cell-cell signaling	0.371 %	-2.6864	0.81	0.20
GO:0019220	regulation of phosphate metabolic process	0.561 %	-1.1931	0.79	0.24
GO:0035303	regulation of dephosphorylation	0.060 %	-1.0523	0.82	0.82
GO:0035304	regulation of protein dephosphorylation	0.047 %	-1.0655	0.79	0.95
GO:0010921	regulation of phosphatase activity	0.035 %	-1.1995	0.82	0.78
GO:0043666	regulation of phosphoprotein phosphatase activity	0.022 %	-1.2186	0.80	0.93
GO:0080163	regulation of protein serine/threonine phosphatase activity	0.017 %	-1.3055	0.81	0.89
GO:0051716	cellular response to stimulus	12.637 %	-1.1126	0.86	0.25
GO:0048519	negative regulation of biological process	3.716 %	-1.1926	0.85	0.25
GO:0060771	phyllotactic patterning	0.017 %	-1.3570	0.72	0.26
GO:0060774	auxin mediated signaling pathway involved in phyllotactic patterning	0.009 %	-1.4158	0.61	0.79
GO:0060772	leaf phyllotactic patterning	0.013 %	-1.3709	0.72	0.95
GO:0006839	mitochondrial transport	0.522 %	-1.0589	0.98	0.27
GO:0000957	mitochondrial RNA catabolic process	0.009 %	-2.1413	0.90	0.29
GO:0000963	mitochondrial RNA processing	0.043 %	-1.4486	0.91	0.67
GO:0048826	cotyledon morphogenesis	0.073 %	-2.9354	0.64	0.29
GO:0044702	single organism reproductive process	5.939 %	-1.4242	0.77	0.78
GO:2000032	regulation of secondary shoot formation	0.030 %	-1.6396	0.61	0.64
GO:0010228	vegetative to reproductive phase transition of meristem	0.738 %	-1.0727	0.63	0.68
GO:0060688	regulation of morphogenesis of a branching structure	0.030 %	-1.6396	0.63	0.82
GO:1905392	plant organ morphogenesis	1.485 %	-1.3075	0.61	0.71
GO:0010214	seed coat development	0.168 %	-1.3417	0.66	0.58
GO:0048825	cotyledon development	0.190 %	-2.0580	0.64	0.63
GO:0048367	shoot system development	3.599 %	-1.0038	0.61	0.76
GO:0048316	seed development	2.305 %	-1.4241	0.58	0.78
GO:0010154	fruit development	2.443 %	-1.6656	0.59	0.56
GO:0048608	reproductive structure development	4.674 %	-1.4760	0.56	0.86
GO:0048598	embryonic morphogenesis	0.125 %	-2.4329	0.68	0.58
GO:1900618	regulation of shoot system morphogenesis	0.073 %	-1.4158	0.59	0.70
GO:0022414	reproductive process	6.629 %	-1.3265	0.86	0.91
GO:1905428	regulation of plant organ formation	0.047 %	-1.4158	0.65	0.69
GO:0009965	leaf morphogenesis	0.380 %	-2.1545	0.64	0.77
GO:0061458	reproductive system development	4.674 %	-1.4760	0.60	0.72
GO:2000241	regulation of reproductive process	1.092 %	-1.0988	0.78	0.62

GO:0010016	shoot system morphogenesis	0.708 %	-2.1324	0.63	0.53
GO:0071497	cellular response to freezing	0.013 %	-1.4486	0.89	0.31
GO:0050826	response to freezing	0.086 %	-1.0272	0.91	0.51
GO:0010541	acropetal auxin transport	0.013 %	-1.0334	0.86	0.32
GO:0051093	negative regulation of developmental process	0.492 %	-3.9451	0.61	0.33
GO:2000026	regulation of multicellular organismal development	1.765 %	-3.0179	0.53	0.81
GO:0080113	regulation of seed growth	0.039 %	-1.3435	0.60	0.64
GO:0023057	negative regulation of signaling	0.345 %	-2.0419	0.74	0.58
GO:0090344	negative regulation of cell aging	0.004 %	-1.8873	0.64	0.90
GO:0051241	negative regulation of multicellular organismal process	0.445 %	-3.2596	0.64	0.59
GO:0090342	regulation of cell aging	0.017 %	-2.7247	0.64	0.51
GO:0048831	regulation of shoot system development	0.850 %	-1.5235	0.54	0.87
GO:0007569	cell aging	0.022 %	-2.7053	0.71	0.61
GO:0048585	negative regulation of response to stimulus	0.699 %	-1.1460	0.76	0.62
GO:0048580	regulation of post-embryonic development	1.303 %	-1.2719	0.54	0.92
GO:0010648	negative regulation of cell communication	0.345 %	-2.0419	0.77	0.58
GO:0045995	regulation of embryonic development	0.056 %	-1.2186	0.63	0.60
GO:2000242	negative regulation of reproductive process	0.220 %	-1.5181	0.75	0.55
GO:0009909	regulation of flower development	0.609 %	-1.1338	0.52	0.84
GO:0048518	positive regulation of biological process	4.398 %	-1.0027	0.85	0.33
GO:0051239	regulation of multicellular organismal process	1.972 %	-2.6186	0.72	0.35
GO:0046477	glycosylceramide catabolic process	0.017 %	-1.4003	0.75	0.38
GO:0030149	sphingolipid catabolic process	0.022 %	-1.3709	0.76	0.97
GO:0046466	membrane lipid catabolic process	0.026 %	-1.1637	0.78	0.68
GO:0016139	glycoside catabolic process	0.009 %	-1.1812	0.83	0.63
GO:0046479	glycosphingolipid catabolic process	0.017 %	-1.4003	0.75	1.00
GO:0006672	ceramide metabolic process	0.078 %	-1.2491	0.78	0.80
GO:0006677	glycosylceramide metabolic process	0.022 %	-1.4003	0.78	0.99
GO:0006687	glycosphingolipid metabolic process	0.022 %	-1.4003	0.78	0.73
GO:0046514	ceramide catabolic process	0.017 %	-1.4003	0.76	0.97
GO:0019377	glycolipid catabolic process	0.017 %	-1.4003	0.76	0.95
GO:0070482	response to oxygen levels	0.285 %	-2.0419	0.90	0.39
GO:0051174	regulation of phosphorus metabolic process	0.561 %	-1.1931	0.82	0.40
GO:0048544	recognition of pollen	0.211 %	-1.0148	0.69	0.40
GO:0009875	pollen-pistil interaction	0.237 %	-1.0088	0.75	0.74
GO:0031221	arabinan metabolic process	0.009 %	-2.7247	0.84	0.40
GO:1900030	regulation of pectin biosynthetic process	0.004 %	-1.8873	0.78	0.40
GO:0032885	regulation of polysaccharide biosynthetic process	0.043 %	-1.0334	0.75	0.80
GO:0050793	regulation of developmental process	2.577 %	-3.4761	0.65	0.40
GO:0046246	terpene biosynthetic process	0.043 %	-1.1724	0.81	0.41
GO:0006002	fructose 6-phosphate metabolic process	0.060 %	-1.2387	0.90	0.41
GO:0016137	glycoside metabolic process	0.017 %	-1.0459	0.86	0.41
GO:0046459	short-chain fatty acid metabolic process	0.013 %	-1.4158	0.82	0.41
GO:0010646	regulation of cell communication	1.139 %	-1.4595	0.81	0.42
GO:0009719	response to endogenous stimulus	7.359 %	-1.0289	0.90	0.43
GO:0007389	pattern specification process	0.742 %	-1.2384	0.68	0.44
GO:0023051	regulation of signaling	1.126 %	-1.4675	0.83	0.44
GO:0044700	single organism signaling	8.899 %	-1.4561	0.82	0.64
GO:0006097	glyoxylate cycle	0.030 %	-1.3055	0.80	0.44
GO:0046487	glyoxylate metabolic process	0.035 %	-1.2820	0.83	0.72
GO:0009733	response to auxin	1.709 %	-1.9683	0.86	0.44
GO:0071229	cellular response to acid chemical	2.158 %	-1.0410	0.82	0.79
GO:0009740	gibberellic acid mediated signaling pathway	0.341 %	-1.5803	0.66	0.62
GO:0009739	response to gibberellin	0.626 %	-1.8291	0.86	0.55
GO:0009725	response to hormone	6.871 %	-1.2954	0.84	0.82
GO:0010476	gibberellin mediated signaling pathway	0.345 %	-1.4930	0.66	0.99
GO:0032870	cellular response to hormone stimulus	3.871 %	-1.0432	0.80	0.69
GO:0070887	cellular response to chemical stimulus	5.287 %	-1.4603	0.83	0.52
GO:0071370	cellular response to gibberellin stimulus	0.354 %	-1.4376	0.83	0.89
GO:0071310	cellular response to organic substance	4.601 %	-1.1080	0.81	0.88
GO:0010033	response to organic substance	8.580 %	-1.4401	0.86	0.68
GO:0071495	cellular response to endogenous stimulus	3.988 %	-1.0210	0.87	0.75
GO:1902184	negative regulation of shoot apical meristem development	0.004 %	-2.3626	0.67	0.47
GO:1902183	regulation of shoot apical meristem development	0.039 %	-1.3055	0.65	0.85
GO:0009743	response to carbohydrate	0.518 %	-1.8247	0.88	0.48
GO:0018202	peptidyl-histidine modification	0.060 %	-1.4158	0.92	0.48
GO:0030308	negative regulation of cell growth	0.043 %	-1.1231	0.75	0.49
GO:0009888	tissue development	2.326 %	-3.3938	0.70	0.49
GO:0044767	single-organism developmental process	12.123 %	-1.0604	0.62	0.85
GO:0048856	anatomical structure development	11.722 %	-1.8143	0.66	0.66
GO:0009653	anatomical structure morphogenesis	3.500 %	-2.1231	0.69	0.53
GO:0048731	system development	7.225 %	-1.4116	0.60	0.76

GO:0040019	positive regulation of embryonic development	0.009 %	-1.3055	0.66	0.49
GO:0019605	butyrate metabolic process	0.009 %	-1.4486	0.82	0.49
GO:0080112	seed growth	0.047 %	-1.3435	0.68	0.50
GO:0009893	positive regulation of metabolic process	2.892 %	-1.1187	0.81	0.50
GO:0009896	*positive regulation of catabolic process*	*0.302 %*	*-1.0933*	*0.80*	*0.93*
GO:0031399	*regulation of protein modification process*	*0.699 %*	*-1.0874*	*0.79*	*0.69*
GO:0045732	*positive regulation of protein catabolic process*	*0.276 %*	*-1.0933*	*0.77*	*0.63*

Biological Process (113) Tag Clouds

Scatterplot & Table Interactive Graph TreeMap

GROUP 26

Hide/show dispensable GO terms

Export results to text table (CSV)

Make R script for plotting

term ID	description	frequency	pin?	log_{10} p-value	uniqueness	dispensability
GO:0007623	circadian rhythm	0.539 %		-1.1632	0.98	0.00
GO:0010114	response to red light	0.259 %		-2.9687	0.86	0.00
GO:0010161	*red light signaling pathway*	*0.035 %*		*-1.4686*	*0.75*	*0.83*
GO:0009639	*response to red or far red light*	*0.872 %*		*-2.5277*	*0.86*	*0.65*
GO:0071482	*cellular response to light stimulus*	*0.561 %*		*-1.6380*	*0.79*	*0.94*
GO:0071478	*cellular response to radiation*	*0.583 %*		*-1.6380*	*0.80*	*0.86*
GO:0071489	*cellular response to red or far red light*	*0.350 %*		*-2.2254*	*0.79*	*0.91*
GO:0071491	*cellular response to red light*	*0.043 %*		*-1.4387*	*0.82*	*0.84*
GO:0010017	*red or far-red light signaling pathway*	*0.341 %*		*-2.2440*	*0.72*	*0.81*
GO:0016114	terpenoid biosynthetic process	0.591 %		-2.8431	0.54	0.00
GO:0008299	*isoprenoid biosynthetic process*	*0.738 %*		*-2.5695*	*0.54*	*0.96*
GO:0010686	*tetracyclic triterpenoid biosynthetic process*	*0.004 %*		*-1.7008*	*0.62*	*0.65*
GO:0010685	*tetracyclic triterpenoid metabolic process*	*0.004 %*		*-1.7008*	*0.64*	*0.79*
GO:0010683	*tricyclic triterpenoid metabolic process*	*0.013 %*		*-1.6270*	*0.62*	*0.84*
GO:0016053	*organic acid biosynthetic process*	*2.296 %*		*-1.1496*	*0.59*	*0.54*
GO:0019742	*pentacyclic triterpenoid metabolic process*	*0.004 %*		*-1.5268*	*0.64*	*0.84*
GO:0019745	*pentacyclic triterpenoid biosynthetic process*	*0.004 %*		*-1.7008*	*0.62*	*0.81*
GO:0044255	*cellular lipid metabolic process*	*3.224 %*		*-1.6233*	*0.59*	*0.71*
GO:0016104	*triterpenoid biosynthetic process*	*0.030 %*		*-1.4686*	*0.58*	*0.93*

GO:0016102	diterpenoid biosynthetic process	0.147 %	-2.8189	0.57	0.97
GO:0016101	diterpenoid metabolic process	0.173 %	-2.6262	0.60	0.86
GO:0080003	thalianol metabolic process	0.009 %	-1.7432	0.63	0.87
GO:0006664	glycolipid metabolic process	0.306 %	-1.0954	0.59	0.90
GO:0046488	phosphatidylinositol metabolic process	0.358 %	-1.0060	0.58	0.80
GO:1903509	liposaccharide metabolic process	0.315 %	-1.0482	0.64	0.54
GO:0009686	gibberellin biosynthetic process	0.134 %	-2.8284	0.55	0.84
GO:0008610	lipid biosynthetic process	2.464 %	-1.3678	0.59	0.82
GO:0009685	gibberellin metabolic process	0.138 %	-2.7381	0.55	0.97
GO:0006720	isoprenoid metabolic process	0.850 %	-2.3695	0.61	0.67
GO:0009695	jasmonic acid biosynthetic process	0.086 %	-1.2066	0.68	0.64
GO:0006722	triterpenoid metabolic process	0.035 %	-1.3199	0.64	0.75
GO:0006721	terpenoid metabolic process	0.699 %	-2.6061	0.57	0.94
GO:0010263	tricyclic triterpenoid biosynthetic process	0.009 %	-1.7145	0.60	0.96
GO:0006505	GPI anchor metabolic process	0.095 %	-1.6502	0.59	0.55
GO:0048511	rhythmic process	0.600 %	-1.1533	0.98	0.00
GO:0010166	wax metabolic process	0.082 %	-1.0161	0.95	0.04
GO:0045493	xylan catabolic process	0.017 %	-1.3782	0.90	0.06
GO:0010411	xyloglucan metabolic process	0.259 %	-1.1673	0.88	0.74
GO:0045491	xylan metabolic process	0.173 %	-1.0213	0.89	0.88
GO:0090467	arginine import	0.004 %	-1.9488	0.79	0.07
GO:1902023	L-arginine transport	0.004 %	-1.9488	0.79	0.86
GO:0043092	L-amino acid import	0.017 %	-1.9488	0.78	0.80
GO:0043091	L-arginine import	0.004 %	-1.9488	0.78	0.91
GO:0051938	L-glutamate import	0.013 %	-2.0276	0.78	0.85
GO:0072527	pyrimidine-containing compound metabolic process	0.298 %	-1.0399	0.81	0.07
GO:0010025	wax biosynthetic process	0.078 %	-1.0482	0.90	0.17
GO:0009739	response to gibberellin	0.626 %	-2.1066	0.90	0.18
GO:0009751	response to salicylic acid	0.876 %	-1.0264	0.90	0.58
GO:0048768	root hair cell tip growth	0.078 %	-2.7212	0.66	0.18
GO:0048765	root hair cell differentiation	0.406 %	-1.3800	0.63	0.97
GO:0048767	root hair elongation	0.229 %	-1.8124	0.64	0.87
GO:0048764	trichoblast maturation	0.406 %	-1.3800	0.63	0.99
GO:1905392	plant organ morphogenesis	1.485 %	-1.5703	0.73	0.57
GO:0010054	trichoblast differentiation	0.427 %	-1.2944	0.63	0.95
GO:0010053	root epidermal cell differentiation	0.488 %	-1.1404	0.63	0.89
GO:0010338	leaf formation	0.022 %	-1.4040	0.78	0.53
GO:0080147	root hair cell development	0.319 %	-1.6146	0.64	0.93
GO:0090627	plant epidermal cell differentiation	0.621 %	-1.1163	0.68	0.54
GO:0048469	cell maturation	0.410 %	-1.3800	0.67	0.76
GO:0010015	root morphogenesis	0.967 %	-1.3888	0.72	0.73
GO:0008356	asymmetric cell division	0.091 %	-1.2192	0.79	0.19
GO:0009267	cellular response to starvation	0.496 %	-1.3388	0.79	0.19
GO:0042594	response to starvation	0.531 %	-1.3039	0.85	0.95
GO:0031668	cellular response to extracellular stimulus	0.691 %	-1.0309	0.79	0.97
GO:0031667	response to nutrient levels	0.639 %	-1.0746	0.85	0.93
GO:0009970	cellular response to sulfate starvation	0.052 %	-1.2410	0.82	0.81
GO:0031669	cellular response to nutrient levels	0.557 %	-1.1971	0.79	0.96
GO:0071496	cellular response to external stimulus	0.712 %	-1.0115	0.89	0.50
GO:0010876	lipid localization	0.708 %	-1.0239	0.91	0.24
GO:0061062	regulation of nematode larval development	0.009 %	-1.9255	0.81	0.25
GO:0002119	nematode larval development	0.009 %	-1.9255	0.82	1.00
GO:0051260	protein homooligomerization	0.151 %	-1.4245	0.92	0.26
GO:0051259	protein oligomerization	0.224 %	-1.0110	0.92	0.68
GO:0010031	circumnutation	0.026 %	-1.8247	0.83	0.26
GO:0050879	multicellular organismal movement	0.026 %	-1.8247	0.83	0.26
GO:0002164	larval development	0.009 %	-1.9255	0.83	0.29
GO:0009944	polarity specification of adaxial/abaxial axis	0.095 %	-1.2885	0.78	0.29
GO:0009943	adaxial/abaxial axis specification	0.108 %	-1.2278	0.78	0.94
GO:0009955	adaxial/abaxial pattern specification	0.151 %	-1.0426	0.78	0.75
GO:0065001	specification of axis polarity	0.112 %	-1.2365	0.78	0.91
GO:0006629	lipid metabolic process	4.683 %	-1.7874	0.70	0.34
GO:0015748	organophosphate ester transport	0.203 %	-1.6050	0.80	0.34
GO:0044205	'de novo' UMP biosynthetic process	0.026 %	-1.9997	0.55	0.36
GO:0046131	pyrimidine ribonucleoside metabolic process	0.173 %	-1.3145	0.54	0.97
GO:0046132	pyrimidine ribonucleoside biosynthetic process	0.129 %	-1.3423	0.53	0.97
GO:0009218	pyrimidine ribonucleotide metabolic process	0.125 %	-1.3782	0.55	0.86
GO:0009220	pyrimidine ribonucleotide biosynthetic process	0.125 %	-1.3782	0.52	0.96
GO:0046112	nucleobase biosynthetic process	0.099 %	-1.3782	0.60	0.89
GO:0046134	pyrimidine nucleoside biosynthetic process	0.129 %	-1.3423	0.53	0.89

GO:0009112	*nucleobase metabolic process*	0.216 %	-1.2321	0.61	0.55
GO:0009129	*pyrimidine nucleoside monophosphate metabolic process*	0.099 %	-1.5944	0.62	0.61
GO:0009130	*pyrimidine nucleoside monophosphate biosynthetic process*	0.095 %	-1.5944	0.58	0.98
GO:0072528	*pyrimidine-containing compound biosynthetic process*	0.233 %	-1.0954	0.67	0.94
GO:0046049	*UMP metabolic process*	0.078 %	-1.6502	0.54	0.99
GO:0009174	*pyrimidine ribonucleoside monophosphate biosynthetic process*	0.078 %	-1.6502	0.58	0.99
GO:0009173	*pyrimidine ribonucleoside monophosphate metabolic process*	0.082 %	-1.6502	0.60	0.97
GO:0019856	*pyrimidine nucleobase biosynthetic process*	0.056 %	-1.5739	0.56	0.86
GO:0006207	*'de novo' pyrimidine nucleobase biosynthetic process*	0.039 %	-1.6623	0.57	0.90
GO:0006206	*pyrimidine nucleobase metabolic process*	0.112 %	-1.4460	0.57	0.77
GO:0006220	*pyrimidine nucleotide metabolic process*	0.168 %	-1.2737	0.55	0.91
GO:0006213	*pyrimidine nucleoside metabolic process*	0.181 %	-1.3145	0.55	0.89
GO:0006221	*pyrimidine nucleotide biosynthetic process*	0.168 %	-1.2885	0.52	0.98
GO:0006222	*UMP biosynthetic process*	0.078 %	-1.6502	0.52	0.93
GO:0071214	cellular response to abiotic stimulus	0.889 %	-1.1822	0.83	0.44
GO:0009736	cytokinin-activated signaling pathway	0.328 %	-1.1419	0.76	0.46
GO:0071368	*cellular response to cytokinin stimulus*	0.337 %	-1.0623	0.84	0.80
GO:0080160	selenate transport	0.077 %	-1.7902	0.88	0.48
GO:0006820	*anion transport*	1.524 %	-1.1995	0.88	0.54
GO:0006865	*amino acid transport*	0.419 %	-1.0623	0.75	0.76
GO:0006869	*lipid transport*	0.596 %	-1.1349	0.78	0.79
GO:0043090	*amino acid import*	0.056 %	-1.2835	0.77	0.62
GO:0015914	*phospholipid transport*	0.065 %	-1.7583	0.77	0.57
GO:0015849	organic acid transport	0.682 %	-1.0623	0.78	0.50

Biological Process (226) Tag Clouds

Scatterplot & Table Interactive Graph TreeMap

GROUP 27

Tip: your resulting list of GO terms seems to be quite long. If you want to reduce it further, press the Back button in your browser and choose a different setting for the "allowed similarity" parameter.

Hide/show dispensable GO terms

Export results to text table (CSV)

Make R script for plotting

term ID	description	frequency	pin?	log_{10} p-value	uniqueness	dispensability
GO:0010226	response to lithium ion	0.017 %		-3.3183	0.95	0.00
GO:0022610	biological adhesion	0.060 %		-1.0154	0.99	0.00
GO:0032501	multicellular organismal process	11.360 %		-1.6284	0.99	0.00
GO:0032502	developmental process	12.689 %		-1.5715	0.99	0.00
GO:0040007	growth	2.529 %		-1.2110	0.99	0.00
GO:0044699	single-organism process	41.709 %		-4.8075	0.99	0.00
GO:0050896	response to stimulus	25.943 %		-1.5441	0.99	0.00
GO:0071824	protein-DNA complex subunit organization	0.432 %		-7.0566	0.87	0.00
GO:0071822	*protein complex subunit organization*	*1.528 %*		*-3.2516*	*0.85*	*0.69*
GO:0006325	*chromatin organization*	*1.675 %*		*-4.2066*	*0.85*	*0.60*
GO:0022607	*cellular component assembly*	*3.949 %*		*-1.7982*	*0.85*	*0.79*
GO:0065003	*macromolecular complex assembly*	*3.220 %*		*-2.8953*	*0.84*	*0.89*
GO:0034622	*cellular macromolecular complex assembly*	*2.900 %*		*-3.4765*	*0.84*	*0.75*
GO:0006461	*protein complex assembly*	*1.303 %*		*-3.4188*	*0.85*	*0.84*
GO:0071840	cellular component organization or biogenesis	14.454 %		-1.4482	0.99	0.00
GO:0010425	DNA methylation on cytosine within a CNG sequence	0.004 %		-2.1160	0.93	0.03
GO:0032776	*DNA methylation on cytosine*	*0.078 %*		*-1.2808*	*0.91*	*0.69*

GO:0009294	DNA mediated transformation	0.099 %	-1.5959	0.95	0.04
GO:0044764	multi-organism cellular process	0.414 %	-1.0441	0.94	0.53
GO:0009292	genetic transfer	0.099 %	-1.5959	0.95	0.79
GO:1903047	mitotic cell cycle process	1.083 %	-3.3132	0.72	0.04
GO:0000281	mitotic cytokinesis	0.311 %	-1.1595	0.74	0.86
GO:0000278	mitotic cell cycle	1.152 %	-3.1183	0.73	0.80
GO:0044772	mitotic cell cycle phase transition	0.190 %	-2.2788	0.74	0.82
GO:0044770	cell cycle phase transition	0.207 %	-2.2788	0.76	0.75
GO:0044839	cell cycle G2/M phase transition	0.091 %	-1.4782	0.75	0.93
GO:0044843	cell cycle G1/S phase transition	0.069 %	-1.4410	0.76	0.91
GO:0000910	cytokinesis	0.384 %	-1.0353	0.74	0.79
GO:1902749	regulation of cell cycle G2/M phase transition	0.082 %	-1.4782	0.73	0.98
GO:1901987	regulation of cell cycle phase transition	0.164 %	-1.1392	0.72	0.97
GO:1901990	regulation of mitotic cell cycle phase transition	0.147 %	-1.1392	0.72	0.96
GO:0007135	meiosis II	0.073 %	-1.5640	0.71	0.68
GO:0061640	cytoskeleton-dependent cytokinesis	0.332 %	-1.0555	0.74	0.97
GO:0000082	G1/S transition of mitotic cell cycle	0.052 %	-1.4410	0.77	0.89
GO:0010389	regulation of G2/M transition of mitotic cell cycle	0.078 %	-1.4782	0.73	0.92
GO:0000086	G2/M transition of mitotic cell cycle	0.086 %	-1.4782	0.76	0.99
GO:0022402	cell cycle process	1.713 %	-2.0922	0.72	0.84
GO:0071554	cell wall organization or biogenesis	3.168 %	-1.0542	0.94	0.05
GO:0072593	reactive oxygen species metabolic process	0.734 %	-1.6013	0.93	0.05
GO:0006833	water transport	0.194 %	-1.7640	0.86	0.09
GO:0008283	cell proliferation	0.470 %	-1.4735	0.88	0.10
GO:2000026	regulation of multicellular organismal development	1.765 %	-2.8364	0.66	0.12
GO:0044707	single-multicellular organism process	10.699 %	-2.0899	0.72	0.86
GO:0099402	plant organ development	3.763 %	-1.2138	0.68	0.67
GO:0051240	positive regulation of multicellular organismal process	0.401 %	-1.3892	0.73	0.81
GO:0044767	single-organism developmental process	12.123 %	-1.7629	0.68	0.82
GO:0022622	root system development	1.895 %	-2.1434	0.70	0.61
GO:0048580	regulation of post-embryonic development	1.303 %	-1.6719	0.66	0.92
GO:0010054	trichoblast differentiation	0.427 %	-1.9161	0.63	0.83
GO:0010053	root epidermal cell differentiation	0.488 %	-1.6283	0.63	0.94
GO:0007275	multicellular organism development	10.423 %	-2.3101	0.66	0.72
GO:2000123	positive regulation of stomatal complex development	0.013 %	-1.8621	0.74	0.59
GO:0045995	regulation of embryonic development	0.056 %	-1.1940	0.73	0.67
GO:0061062	regulation of nematode larval development	0.009 %	-1.5408	0.76	0.57
GO:0010015	root morphogenesis	0.967 %	-1.3974	0.69	0.90
GO:0048766	root hair initiation	0.047 %	-1.3461	0.64	0.67
GO:2000038	regulation of stomatal complex development	0.039 %	-1.0092	0.72	0.73
GO:1905392	plant organ morphogenesis	1.485 %	-1.2069	0.69	0.77
GO:0090344	negative regulation of cell aging	0.004 %	-1.8621	0.74	0.51
GO:0090342	regulation of cell aging	0.017 %	-1.1843	0.72	0.90
GO:0007569	cell aging	0.022 %	-1.1749	0.75	0.56
GO:0048825	cotyledon development	0.190 %	-1.1332	0.72	0.62
GO:0048364	root development	1.886 %	-2.1593	0.69	0.51
GO:0048856	anatomical structure development	11.722 %	-1.7403	0.77	0.85
GO:0009791	post-embryonic development	5.848 %	-1.5233	0.68	0.78
GO:0045596	negative regulation of cell differentiation	0.052 %	-1.0031	0.70	0.61
GO:0051094	positive regulation of developmental process	0.436 %	-1.3003	0.77	0.75
GO:0051093	negative regulation of developmental process	0.492 %	-1.3344	0.77	0.76
GO:0040019	positive regulation of embryonic development	0.009 %	-1.2808	0.76	0.60
GO:0002119	nematode larval development	0.009 %	-1.5408	0.79	1.00
GO:0048731	system development	7.225 %	-2.1516	0.67	0.82
GO:0009909	regulation of flower development	0.609 %	-1.0747	0.66	0.84
GO:1901334	lactone metabolic process	0.026 %	-1.3754	0.94	0.12
GO:0009606	tropism	0.341 %	-2.2500	0.93	0.14
GO:0030104	water homeostasis	0.052 %	-2.0054	0.94	0.15
GO:0080144	amino acid homeostasis	0.060 %	-1.0835	0.93	0.53
GO:0030155	regulation of cell adhesion	0.009 %	-1.7756	0.94	0.16
GO:0007155	cell adhesion	0.060 %	-1.0154	0.98	0.88
GO:2000603	regulation of secondary growth	0.009 %	-2.4095	0.86	0.16
GO:0045927	positive regulation of growth	0.104 %	-1.9012	0.90	0.54
GO:2000605	positive regulation of secondary growth	0.004 %	-2.4531	0.85	0.87
GO:0040008	regulation of growth	1.148 %	-1.4825	0.90	0.63
GO:0080117	secondary growth	0.022 %	-1.9968	0.87	0.95
GO:0010070	zygote asymmetric cell division	0.017 %	-1.5408	0.83	0.17
GO:0051302	regulation of cell division	0.199 %	-1.3063	0.78	0.55
GO:0010069	zygote asymmetric cytokinesis in embryo sac	0.013 %	-1.5408	0.67	0.79
GO:0009629	response to gravity	0.311 %	-1.6291	0.95	0.17

GO:0044763	single-organism cellular process	23.034 %	-2.6241	0.81	0.19
GO:0009410	response to xenobiotic stimulus	0.013 %	-1.0281	0.95	0.21
GO:2000377	regulation of reactive oxygen species metabolic process	0.190 %	-1.6533	0.89	0.21
GO:0042127	regulation of cell proliferation	0.293 %	-1.2672	0.84	0.22
GO:0007018	microtubule-based movement	0.281 %	-1.4507	0.82	0.22
GO:0006928	movement of cell or subcellular component	0.363 %	-1.3878	0.81	0.22
GO:0045176	apical protein localization	0.004 %	-1.5408	0.97	0.22
GO:0009698	phenylpropanoid metabolic process	0.505 %	-3.2651	0.75	0.23
GO:0044550	*secondary metabolite biosynthetic process*	*1.183 %*	*-2.1711*	*0.77*	*0.80*
GO:0009699	*phenylpropanoid biosynthetic process*	*0.337 %*	*-2.9244*	*0.74*	*0.70*
GO:0009808	*lignin metabolic process*	*0.285 %*	*-2.5307*	*0.76*	*0.91*
GO:0009809	*lignin biosynthetic process*	*0.181 %*	*-1.9650*	*0.75*	*0.88*
GO:0010345	*suberin biosynthetic process*	*0.060 %*	*-1.9396*	*0.77*	*0.80*
GO:0046274	*lignin catabolic process*	*0.078 %*	*-1.9968*	*0.74*	*0.82*
GO:0046271	*phenylpropanoid catabolic process*	*0.078 %*	*-1.9476*	*0.74*	*0.82*
GO:0007017	microtubule-based process	0.859 %	-1.9161	0.80	0.24
GO:1901606	alpha-amino acid catabolic process	0.263 %	-1.7095	0.72	0.25
GO:0046395	*carboxylic acid catabolic process*	*0.514 %*	*-1.0671*	*0.71*	*0.94*
GO:0009071	*serine family amino acid catabolic process*	*0.043 %*	*-1.1940*	*0.75*	*0.86*
GO:0009065	*glutamine family amino acid catabolic process*	*0.043 %*	*-1.0480*	*0.75*	*0.86*
GO:0009063	*cellular amino acid catabolic process*	*0.276 %*	*-1.4930*	*0.72*	*0.89*
GO:0006546	*glycine catabolic process*	*0.030 %*	*-1.1940*	*0.76*	*0.84*
GO:0006525	*arginine metabolic process*	*0.095 %*	*-1.0346*	*0.77*	*0.66*
GO:0006527	*arginine catabolic process*	*0.022 %*	*-1.4236*	*0.76*	*0.87*
GO:0019544	*arginine catabolic process to glutamate*	*0.009 %*	*-1.6422*	*0.77*	*0.77*
GO:0007166	cell surface receptor signaling pathway	1.204 %	-1.1064	0.78	0.25
GO:0080170	hydrogen peroxide transmembrane transport	0.022 %	-1.1064	0.97	0.26
GO:0051301	cell division	1.528 %	-3.0507	0.79	0.26
GO:0014070	response to organic cyclic compound	1.606 %	-2.0258	0.93	0.29
GO:0009751	*response to salicylic acid*	*0.876 %*	*-1.4188*	*0.93*	*0.51*
GO:0009741	*response to brassinosteroid*	*0.410 %*	*-1.3892*	*0.93*	*0.79*
GO:0009743	*response to carbohydrate*	*0.518 %*	*-1.1020*	*0.94*	*0.51*
GO:0009739	*response to gibberellin*	*0.626 %*	*-1.1051*	*0.93*	*0.58*
GO:0007049	cell cycle	2.525 %	-2.0016	0.78	0.29
GO:0009887	animal organ morphogenesis	0.004 %	-1.7516	0.79	0.29
GO:2000027	*regulation of organ morphogenesis*	*0.004 %*	*-1.1308*	*0.75*	*1.00*
GO:0048513	animal organ development	0.004 %	-1.3841	0.81	0.29
GO:0009605	response to external stimulus	6.366 %	-2.1938	0.94	0.29
GO:0019748	secondary metabolic process	1.938 %	-2.7707	0.81	0.30
GO:1901362	organic cyclic compound biosynthetic process	14.769 %	-1.2073	0.92	0.30
GO:0019438	*aromatic compound biosynthetic process*	*14.247 %*	*-1.0785*	*0.88*	*0.55*
GO:0002164	larval development	0.009 %	-1.5408	0.80	0.30
GO:0008105	asymmetric protein localization	0.004 %	-1.5408	0.97	0.32
GO:0044347	cell wall polysaccharide catabolic process	0.004 %	-1.6145	0.90	0.33
GO:0016998	*cell wall macromolecule catabolic process*	*0.112 %*	*-1.2244*	*0.89*	*0.59*
GO:0051276	chromosome organization	1.118 %	-4.2492	0.87	0.35
GO:0010229	inflorescence development	0.104 %	-2.3560	0.75	0.37
GO:0080001	mucilage extrusion from seed coat	0.022 %	-1.0413	0.77	0.38
GO:0006629	lipid metabolic process	4.683 %	-1.9455	0.79	0.39
GO:1902578	single-organism localization	5.481 %	-1.5901	0.83	0.40
GO:0080190	lateral growth	0.022 %	-1.9968	0.88	0.40
GO:0090627	plant epidermal cell differentiation	0.621 %	-1.5838	0.68	0.41
GO:0008610	lipid biosynthetic process	2.464 %	-1.9056	0.74	0.41
GO:0044242	*cellular lipid catabolic process*	*0.315 %*	*-1.2097*	*0.70*	*0.62*
GO:0044255	*cellular lipid metabolic process*	*3.224 %*	*-1.7305*	*0.70*	*0.82*
GO:0016106	*sesquiterpenoid biosynthetic process*	*0.138 %*	*-1.3057*	*0.73*	*0.84*
GO:0016114	*terpenoid biosynthetic process*	*0.591 %*	*-1.7586*	*0.70*	*0.66*
GO:0016121	*carotene catabolic process*	*0.013 %*	*-1.6145*	*0.74*	*0.67*
GO:0016119	*carotene metabolic process*	*0.026 %*	*-1.3322*	*0.76*	*0.85*
GO:1901601	*strigolactone biosynthetic process*	*0.026 %*	*-1.3754*	*0.74*	*0.85*
GO:1901600	*strigolactone metabolic process*	*0.026 %*	*-1.3754*	*0.75*	*1.00*
GO:0006714	*sesquiterpenoid metabolic process*	*0.181 %*	*-1.2039*	*0.73*	*0.86*
GO:0006720	*isoprenoid metabolic process*	*0.850 %*	*-1.3324*	*0.72*	*0.69*
GO:0006721	*terpenoid metabolic process*	*0.699 %*	*-1.5429*	*0.71*	*0.94*
GO:0000038	*very long-chain fatty acid metabolic process*	*0.065 %*	*-1.0548*	*0.76*	*0.60*
GO:0008300	*isoprenoid catabolic process*	*0.052 %*	*-1.0761*	*0.72*	*0.76*
GO:0008299	*isoprenoid biosynthetic process*	*0.738 %*	*-1.5101*	*0.69*	*0.96*
GO:0006631	*fatty acid metabolic process*	*1.101 %*	*-1.1082*	*0.69*	*0.72*
GO:0046247	*terpene catabolic process*	*0.013 %*	*-1.6145*	*0.74*	*0.84*
GO:1901336	*lactone biosynthetic process*	*0.026 %*	*-1.3754*	*0.91*	*1.00*
GO:0009395	*phospholipid catabolic process*	*0.052 %*	*-1.3322*	*0.73*	*0.70*
GO:0042761	*very long-chain fatty acid biosynthetic process*	*0.056 %*	*-1.1308*	*0.74*	*0.52*
GO:0009682	induced systemic resistance	0.125 %	-1.6809	0.94	0.42

GO:0032973	amino acid export	0.082 %	-1.3322	0.87	0.42
GO:0008272	sulfate transport	0.078 %	-1.2244	0.87	0.58
GO:1905393	plant organ formation	0.427 %	-1.3324	0.80	0.42
GO:0048646	anatomical structure formation involved in morphogenesis	0.755 %	-1.1701	0.80	0.63
GO:0010338	leaf formation	0.022 %	-1.0281	0.76	0.70
GO:0006884	cell volume homeostasis	0.009 %	-1.2244	0.78	0.42
GO:0009992	cellular water homeostasis	0.004 %	-1.2460	0.79	0.71
GO:0051211	anisotropic cell growth	0.017 %	-1.4236	0.83	0.42
GO:0009958	positive gravitropism	0.142 %	-2.4082	0.93	0.42
GO:0009630	gravitropism	0.281 %	-1.6783	0.93	0.93
GO:0016099	monoterpenoid biosynthetic process	0.004 %	-1.4782	0.78	0.42
GO:0072348	sulfur compound transport	0.147 %	-1.1567	0.86	0.44
GO:0090558	plant epidermis development	1.001 %	-1.9214	0.79	0.44
GO:0010068	protoderm histogenesis	0.004 %	-1.6145	0.78	0.50
GO:0010082	regulation of root meristem growth	0.086 %	-1.0618	0.67	0.75
GO:0010492	maintenance of shoot apical meristem identity	0.056 %	-1.1478	0.77	0.61
GO:0010374	stomatal complex development	0.220 %	-1.4225	0.73	0.69
GO:0009744	response to sucrose	0.237 %	-1.0244	0.94	0.44
GO:0034285	response to disaccharide	0.242 %	-1.0244	0.94	0.87
GO:0003002	regionalization	0.617 %	-1.9445	0.72	0.44
GO:0090057	root radial pattern formation	0.013 %	-1.9706	0.76	0.69
GO:0010222	stem vascular tissue pattern formation	0.026 %	-1.6145	0.77	0.73
GO:0060774	auxin mediated signaling pathway involved in phyllotactic patterning	0.009 %	-1.3909	0.72	0.78
GO:0060771	phyllotactic patterning	0.017 %	-1.3322	0.77	0.81
GO:0060772	leaf phyllotactic patterning	0.013 %	-1.3461	0.78	0.95
GO:0009798	axis specification	0.194 %	-1.3916	0.74	0.86
GO:0009956	radial pattern formation	0.078 %	-1.8790	0.76	0.79
GO:2000241	regulation of reproductive process	1.092 %	-1.0273	0.90	0.44
GO:0042044	fluid transport	0.194 %	-1.7640	0.86	0.45
GO:0007389	pattern specification process	0.742 %	-1.7163	0.74	0.45
GO:0043933	macromolecular complex subunit organization	4.933 %	-2.4275	0.86	0.47
GO:0016043	cellular component organization	13.263 %	-1.5132	0.85	0.66
GO:0044085	cellular component biogenesis	5.783 %	-1.2003	0.90	0.68
GO:0006996	organelle organization	6.439 %	-1.4111	0.86	0.69
GO:0019853	L-ascorbic acid biosynthetic process	0.112 %	-1.2808	0.76	0.47
GO:0019852	L-ascorbic acid metabolic process	0.112 %	-1.2808	0.78	0.85
GO:0050793	regulation of developmental process	2.577 %	-2.2086	0.77	0.47
GO:0048869	cellular developmental process	4.148 %	-1.0051	0.64	0.53
GO:0060918	auxin transport	0.406 %	-1.5302	0.82	0.47
GO:0044765	single-organism transport	5.287 %	-1.6722	0.82	0.65
GO:0009914	hormone transport	0.436 %	-1.5101	0.81	0.81
GO:0006334	nucleosome assembly	0.134 %	-7.3497	0.85	0.47
GO:0006323	DNA packaging	0.220 %	-6.6346	0.88	0.82
GO:0006333	chromatin assembly or disassembly	0.207 %	-6.5284	0.87	0.63
GO:0065004	protein-DNA complex assembly	0.393 %	-7.0566	0.86	0.91
GO:0034728	nucleosome organization	0.173 %	-7.3497	0.87	0.84
GO:0031497	chromatin assembly	0.164 %	-6.8281	0.85	0.95
GO:0061647	histone H3-K9 modification	0.069 %	-1.0217	0.86	0.64
GO:0051567	histone H3-K9 methylation	0.065 %	-1.0217	0.86	0.58
GO:0071103	DNA conformation change	0.539 %	-5.6441	0.87	0.75
GO:0031669	cellular response to nutrient levels	0.557 %	-1.7338	0.87	0.47
GO:0042594	response to starvation	0.531 %	-1.2397	0.91	0.96
GO:0009991	response to extracellular stimulus	0.777 %	-1.2475	0.93	0.53
GO:0009267	cellular response to starvation	0.496 %	-1.2867	0.87	0.96
GO:0031668	cellular response to extracellular stimulus	0.691 %	-1.4270	0.87	0.97
GO:0031667	response to nutrient levels	0.639 %	-1.5069	0.91	0.94
GO:0071496	cellular response to external stimulus	0.712 %	-1.3916	0.93	0.51
GO:0048281	inflorescence morphogenesis	0.013 %	-1.3461	0.77	0.48
GO:0010358	leaf shaping	0.035 %	-1.1749	0.76	0.58
GO:0044712	single-organism catabolic process	2.676 %	-1.4932	0.77	0.48
GO:0044248	cellular catabolic process	5.939 %	-1.4612	0.86	0.72
GO:0051239	regulation of multicellular organismal process	1.972 %	-2.4453	0.80	0.48
GO:0009888	tissue development	2.326 %	-1.1853	0.80	0.49
GO:0016098	monoterpenoid metabolic process	0.004 %	-1.4782	0.79	0.49
GO:0070271	protein complex biogenesis	1.334 %	-3.3423	0.90	0.49
GO:0009790	embryo development	1.683 %	-1.7161	0.72	0.50

Biological Process (146) Tag Clouds

Scatterplot & Table Interactive Graph TreeMap

GROUP 28

Hide/show dispensable GO terms

Export results to text table (CSV)

Make R script for plotting

term ID	description	frequency	pin?	log_{10} p-value	uniqueness	dispensability
GO:0009410	response to xenobiotic stimulus	0.013 %		-2.6124	0.95	0.00
GO:0045892	negative regulation of transcription, DNA-templated	0.958 %		-3.7253	0.54	0.00
GO:0048523	negative regulation of cellular process	2.719 %		-1.7305	0.64	0.86
GO:0032774	RNA biosynthetic process	11.813 %		-3.0186	0.66	0.83
GO:0080090	regulation of primary metabolic process	13.681 %		-2.9002	0.58	0.82
GO:0010468	regulation of gene expression	12.706 %		-2.3894	0.56	0.80
GO:0031324	negative regulation of cellular metabolic process	1.929 %		-2.6716	0.58	0.91
GO:0031323	regulation of cellular metabolic process	13.906 %		-3.0536	0.56	0.80
GO:1902679	negative regulation of RNA biosynthetic process	1.057 %		-3.7253	0.54	0.99
GO:0018130	heterocycle biosynthetic process	14.014 %		-2.3652	0.72	0.58
GO:0031327	negative regulation of cellular biosynthetic process	1.429 %		-3.1265	0.55	0.98
GO:0031326	regulation of cellular biosynthetic process	12.339 %		-2.8256	0.51	0.56
GO:0051252	regulation of RNA metabolic process	11.463 %		-2.6209	0.51	0.80
GO:2001141	regulation of RNA biosynthetic process	11.282 %		-2.7173	0.49	0.88
GO:0019219	regulation of nucleobase-containing compound metabolic process	11.700 %		-2.4887	0.52	0.85
GO:0051253	negative regulation of RNA metabolic process	1.062 %		-3.7126	0.56	0.96
GO:0016070	RNA metabolic process	17.562 %		-1.6564	0.72	0.67
GO:2000112	regulation of cellular macromolecule biosynthetic process	12.003 %		-2.1964	0.50	0.87

GO:2000113	negative regulation of cellular macromolecule biosynthetic process	1.256 %	-3.2583	0.54	0.94
GO:0045934	negative regulation of nucleobase-containing compound metabolic process	1.139 %	-3.5370	0.56	0.92
GO:0010629	negative regulation of gene expression	1.601 %	-2.8059	0.59	0.88
GO:0006355	regulation of transcription, DNA-templated	11.234 %	-2.7173	0.49	0.87
GO:0044271	cellular nitrogen compound biosynthetic process	17.018 %	-1.1429	0.71	0.61
GO:0006351	transcription, DNA-templated	11.709 %	-3.0186	0.65	0.56
GO:0060255	regulation of macromolecule metabolic process	14.091 %	-2.8725	0.57	0.83
GO:1903507	negative regulation of nucleic acid-templated transcription	1.057 %	-3.7253	0.54	0.98
GO:1903506	regulation of nucleic acid-templated transcription	11.282 %	-2.7173	0.49	0.89
GO:0097659	nucleic acid-templated transcription	11.761 %	-3.0186	0.65	0.88
GO:1901576	organic substance biosynthetic process	22.969 %	-1.1190	0.75	0.66
GO:0010605	negative regulation of macromolecule metabolic process	2.171 %	-2.5536	0.58	0.94
GO:0009892	negative regulation of metabolic process	2.352 %	-2.2909	0.60	0.76
GO:0009890	negative regulation of biosynthetic process	1.450 %	-3.1008	0.56	0.82
GO:0009889	regulation of biosynthetic process	12.408 %	-2.7300	0.54	0.80
GO:0019438	aromatic compound biosynthetic process	14.247 %	-3.0498	0.72	0.56
GO:0010556	regulation of macromolecule biosynthetic process	12.076 %	-2.1853	0.51	0.87
GO:1901362	organic cyclic compound biosynthetic process	14.769 %	-2.7896	0.73	0.56
GO:0010558	negative regulation of macromolecule biosynthetic process	1.355 %	-3.2583	0.55	0.96
GO:0034654	nucleobase-containing compound biosynthetic process	13.056 %	-2.4450	0.67	0.81
GO:0051172	negative regulation of nitrogen compound metabolic process	1.433 %	-3.1008	0.58	0.86
GO:0051171	regulation of nitrogen compound metabolic process	12.430 %	-2.4711	0.56	0.80
GO:0048645	animal organ formation	0.004 %	-2.3225	0.74	0.00
GO:2000027	regulation of organ morphogenesis	0.004 %	-1.2581	0.64	1.15
GO:0048504	regulation of timing of animal organ formation	0.004 %	-1.6708	0.63	1.00
GO:0010160	formation of animal organ boundary	0.055 %	-1.2666	0.68	1.15
GO:0003156	regulation of animal organ formation	0.004 %	-1.3634	0.63	1.00
GO:0009887	animal organ morphogenesis	0.004 %	-2.0016	0.75	1.00
GO:0006863	purine nucleobase transport	0.095 %	-1.5202	0.90	0.06
GO:0015851	nucleobase transport	0.108 %	-1.5202	0.90	0.60
GO:0015977	carbon fixation	0.104 %	-1.3027	0.85	0.07
GO:0009959	negative gravitropism	0.035 %	-1.5706	0.94	0.12
GO:0009590	detection of gravity	0.022 %	-1.4611	0.94	0.70
GO:0006884	cell volume homeostasis	0.009 %	-1.3526	0.80	0.13
GO:0009992	cellular water homeostasis	0.004 %	-1.3744	0.80	0.71
GO:0051338	regulation of transferase activity	0.522 %	-1.6489	0.78	0.18
GO:0000079	regulation of cyclin-dependent protein serine/threonine kinase activity	0.138 %	-1.0217	0.65	0.84
GO:0050790	regulation of catalytic activity	2.257 %	-1.0184	0.75	0.80
GO:1904029	regulation of cyclin-dependent protein kinase activity	0.138 %	-1.0217	0.64	0.84
GO:0051347	positive regulation of transferase activity	0.281 %	-1.1289	0.78	0.67
GO:0010467	gene expression	18.304 %	-1.1070	0.85	0.19
GO:0051262	protein tetramerization	0.056 %	-1.2103	0.94	0.21
GO:0010031	circumnutation	0.026 %	-1.5706	0.85	0.22
GO:0008544	epidermis development	0.069 %	-1.9953	0.83	0.22
GO:0010440	stomatal lineage progression	0.069 %	-1.3526	0.72	0.50
GO:0050879	multicellular organismal movement	0.026 %	-1.5706	0.85	0.24
GO:0048513	animal organ development	0.004 %	-1.6254	0.82	0.24
GO:0080140	regulation of jasmonic acid metabolic process	0.017 %	-1.4094	0.69	0.26
GO:0019217	regulation of fatty acid metabolic process	0.069 %	-1.0560	0.66	0.75
GO:0042304	regulation of fatty acid biosynthetic process	0.052 %	-1.2754	0.63	0.90
GO:0046890	regulation of lipid biosynthetic process	0.194 %	-1.0264	0.61	0.88
GO:0080141	regulation of jasmonic acid biosynthetic process	0.013 %	-1.4217	0.66	0.83
GO:1901183	positive regulation of camalexin biosynthetic process	0.009 %	-1.8342	0.61	0.26
GO:0052317	camalexin metabolic process	0.047 %	-1.0217	0.71	0.99
GO:0052318	regulation of phytoalexin metabolic process	0.009 %	-1.8342	0.67	0.81
GO:0052319	regulation of phytoalexin biosynthetic process	0.009 %	-1.8342	0.64	1.00
GO:0052320	positive regulation of phytoalexin metabolic process	0.009 %	-1.8342	0.64	1.00
GO:0052322	positive regulation of phytoalexin biosynthetic process	0.009 %	-1.8342	0.62	1.00
GO:0010120	camalexin biosynthetic process	0.047 %	-1.0217	0.67	0.90
GO:1901182	regulation of camalexin biosynthetic process	0.009 %	-1.8342	0.62	1.00
GO:1900378	positive regulation of secondary metabolite biosynthetic process	0.017 %	-1.5531	0.64	0.79
GO:0044550	secondary metabolite biosynthetic process	1.183 %	-1.2437	0.71	0.56
GO:0051176	positive regulation of sulfur metabolic process	0.035 %	-1.6488	0.70	0.81
GO:0009751	response to salicylic acid	0.876 %	-1.8455	0.92	0.27
GO:0071229	cellular response to acid chemical	2.158 %	-1.4086	0.87	0.66
GO:0071446	cellular response to salicylic acid stimulus	0.242 %	-1.3063	0.88	0.79
GO:0009863	salicylic acid mediated signaling pathway	0.211 %	-1.4043	0.70	0.75
GO:0010033	response to organic substance	8.580 %	-1.0953	0.92	0.55
GO:0014070	response to organic cyclic compound	1.606 %	-1.0154	0.93	0.51
GO:0010201	response to continuous far red light stimulus by the high-irradiance response system	0.009 %	-1.3219	0.95	0.27

GO:2000904	regulation of starch metabolic process	0.043 %	-1.3857	0.73	0.28
GO:0032881	*regulation of polysaccharide metabolic process*	0.078 %	-1.0771	0.73	0.83
GO:0010036	response to boron-containing substance	0.022 %	-1.2666	0.95	0.28
GO:2000278	regulation of DNA biosynthetic process	0.022 %	-1.8687	0.69	0.29
GO:0051973	*positive regulation of telomerase activity*	0.009 %	-1.9063	0.66	0.92
GO:2000573	*positive regulation of DNA biosynthetic process*	0.013 %	-1.9063	0.67	0.66
GO:0071897	*DNA biosynthetic process*	0.315 %	-1.6079	0.76	0.50
GO:0051972	*regulation of telomerase activity*	0.013 %	-1.9063	0.67	0.94
GO:0051054	*positive regulation of DNA metabolic process*	0.086 %	-1.5531	0.65	0.72
GO:0009553	embryo sac development	0.565 %	-1.7224	0.77	0.30
GO:0080029	cellular response to boron-containing substance levels	0.017 %	-1.2666	0.90	0.31
GO:0048457	floral whorl morphogenesis	0.004 %	-2.2472	0.77	0.32
GO:0010223	*secondary shoot formation*	0.112 %	-2.2337	0.72	0.58
GO:0010199	*organ boundary specification between lateral organs and the meristem*	0.039 %	-1.1168	0.76	0.96
GO:0090691	*formation of plant organ boundary*	0.069 %	-1.0361	0.75	0.69
GO:0010346	*shoot axis formation*	0.112 %	-2.2337	0.72	0.80
GO:0006949	*syncytium formation*	0.043 %	-1.1813	0.73	0.67
GO:0048439	*flower morphogenesis*	0.022 %	-1.8687	0.76	0.65
GO:0019222	regulation of metabolic process	14.847 %	-2.9778	0.67	0.33
GO:0050794	*regulation of cellular process*	22.244 %	-1.4795	0.65	0.63
GO:0080006	internode patterning	0.009 %	-2.2472	0.79	0.33
GO:0003002	*regionalization*	0.617 %	-1.1112	0.75	0.67
GO:0010051	*xylem and phloem pattern formation*	0.259 %	-1.0074	0.76	0.88
GO:0048638	regulation of developmental growth	0.466 %	-1.6798	0.61	0.35
GO:0045682	*regulation of epidermis development*	0.017 %	-1.3421	0.69	0.84
GO:0048589	*developmental growth*	1.623 %	-1.1811	0.75	0.83
GO:0009826	*unidimensional cell growth*	1.010 %	-1.1492	0.64	0.81
GO:0001558	*regulation of cell growth*	0.293 %	-1.1764	0.69	0.79
GO:0045604	*regulation of epidermal cell differentiation*	0.017 %	-1.3421	0.64	0.51
GO:0022603	*regulation of anatomical structure morphogenesis*	0.522 %	-1.6245	0.61	0.67
GO:0022604	*regulation of cell morphogenesis*	0.341 %	-1.3710	0.56	0.65
GO:0045595	*regulation of cell differentiation*	0.250 %	-1.1485	0.62	0.63
GO:0051510	*regulation of unidimensional cell growth*	0.168 %	-1.4666	0.57	0.87
GO:0040008	*regulation of growth*	1.148 %	-1.2236	0.73	0.73
GO:0030856	*regulation of epithelial cell differentiation*	0.017 %	-1.3421	0.64	0.84
GO:0034052	positive regulation of plant-type hypersensitive response	0.026 %	-1.5888	0.69	0.36
GO:0010942	*positive regulation of cell death*	0.104 %	-1.0993	0.70	0.81
GO:0010363	*regulation of plant-type hypersensitive response*	0.060 %	-1.1813	0.70	0.78
GO:0047484	*regulation of response to osmotic stress*	0.121 %	-1.2497	0.77	0.57
GO:0009627	*systemic acquired resistance*	0.281 %	-1.2726	0.93	0.60
GO:0043068	*positive regulation of programmed cell death*	0.069 %	-1.2029	0.71	0.88
GO:0048609	multicellular organismal reproductive process	0.587 %	-1.4291	0.83	0.37
GO:0007292	*female gamete generation*	0.022 %	-1.3319	0.82	0.75
GO:0009901	*anther dehiscence*	0.069 %	-1.0459	0.76	0.82
GO:0007281	*germ cell development*	0.004 %	-1.5706	0.76	0.67
GO:0048477	*oogenesis*	0.009 %	-1.5706	0.75	0.70
GO:0001763	morphogenesis of a branching structure	0.125 %	-2.1613	0.75	0.39
GO:0010016	*shoot system morphogenesis*	0.708 %	-1.1599	0.72	0.56
GO:0010232	vascular transport	0.065 %	-1.0936	0.83	0.39
GO:0010233	phloem transport	0.065 %	-1.0936	0.83	0.39
GO:0016117	carotenoid biosynthetic process	0.147 %	-1.0264	0.73	0.44
GO:0016109	*tetraterpenoid biosynthetic process*	0.147 %	-1.0264	0.73	0.97
GO:0048869	cellular developmental process	4.148 %	-1.5154	0.71	0.44
GO:0010076	maintenance of floral meristem identity	0.026 %	-1.6079	0.78	0.47
GO:0010073	*meristem maintenance*	0.427 %	-1.0594	0.75	0.67
GO:0010077	*maintenance of inflorescence meristem identity*	0.039 %	-1.4751	0.78	0.80
GO:0010088	phloem development	0.026 %	-1.2843	0.84	0.47
GO:0032504	multicellular organism reproduction	0.613 %	-1.3504	0.85	0.49

Biological Process (321) Tag Clouds

Scatterplot & Table Interactive Graph TreeMap

GROUP 29

Tip: your resulting list of GO terms seems to be quite long. If you want to reduce it further, press the Back button in your browser and choose a different setting for the "allowed similarity" parameter.

Hide/show dispensable GO terms

Export results to text table (CSV)

Make R script for plotting

term ID	description	frequency	pin?	log_{10} p-value	uniqueness	dispensability
GO:0007049	cell cycle	2.525 %		-11.7378	0.76	0.00
GO:0032501	multicellular organismal process	11.360 %		-1.8048	0.99	0.00
GO:0032502	developmental process	12.689 %		-2.4133	0.99	0.00
GO:0040007	growth	2.529 %		-1.3654	0.99	0.00
GO:0044699	single-organism process	41.709 %		-2.1530	1.00	0.00
GO:0051338	regulation of transferase activity	0.522 %		-7.1772	0.87	0.00
GO:0050790	*regulation of catalytic activity*	*2.257 %*		*-4.8258*	*0.85*	*0.80*
GO:0071840	cellular component organization or biogenesis	14.454 %		-7.7753	0.99	0.00
GO:0010438	cellular response to sulfur starvation	0.030 %		-3.8890	0.92	0.04
GO:0018958	phenol-containing compound metabolic process	0.190 %		-2.2127	0.89	0.04
GO:1901617	*organic hydroxy compound biosynthetic process*	*0.824 %*		*-1.0092*	*0.92*	*0.78*
GO:1901599	*(-)-pinoresinol biosynthetic process*	*0.009 %*		*-1.7702*	*0.78*	*0.77*
GO:1901598	*(-)-pinoresinol metabolic process*	*0.009 %*		*-1.6918*	*0.78*	*0.90*
GO:0010023	*proanthocyanidin biosynthetic process*	*0.035 %*		*-1.1587*	*0.76*	*0.84*
GO:0046189	*phenol-containing compound biosynthetic process*	*0.104 %*		*-2.2127*	*0.89*	*0.66*
GO:0009807	*lignan biosynthetic process*	*0.022 %*		*-1.5683*	*0.77*	*0.95*
GO:0000280	nuclear division	1.079 %		-6.5752	0.71	0.05

interaction

GO:0045185	maintenance of protein location	0.078 %	-1.9161	0.86	0.67
GO:0009699	phenylpropanoid biosynthetic process	0.337 %	-1.9294	0.73	0.24
GO:0044550	secondary metabolite biosynthetic process	1.183 %	-1.3633	0.82	0.80
GO:0009698	phenylpropanoid metabolic process	0.505 %	-1.5756	0.74	0.70
GO:0009806	lignan metabolic process	0.022 %	-1.5683	0.78	0.74
GO:0009808	lignin metabolic process	0.285 %	-1.5487	0.74	0.91
GO:0009809	lignin biosynthetic process	0.181 %	-1.8395	0.74	0.88
GO:0046274	lignin catabolic process	0.078 %	-1.9076	0.74	0.82
GO:0046271	phenylpropanoid catabolic process	0.078 %	-1.8587	0.75	0.82
GO:0006928	movement of cell or subcellular component	0.363 %	-2.2544	0.80	0.24
GO:0080088	spermidine hydroxycinnamate conjugate biosynthetic process	0.009 %	-1.5424	0.90	0.25
GO:0007059	chromosome segregation	0.531 %	-2.7282	0.79	0.25
GO:0048015	phosphatidylinositol-mediated signaling	0.022 %	-1.4729	0.82	0.25
GO:0051726	regulation of cell cycle	0.811 %	-9.8497	0.58	0.27
GO:0000281	mitotic cytokinesis	0.311 %	-1.9037	0.62	0.95
GO:0000278	mitotic cell cycle	1.152 %	-8.0968	0.62	0.80
GO:0001932	regulation of protein phosphorylation	0.432 %	-7.2965	0.69	0.98
GO:0045859	regulation of protein kinase activity	0.423 %	-7.6189	0.68	0.95
GO:0032268	regulation of cellular protein metabolic process	1.921 %	-4.8724	0.70	0.85
GO:0042023	DNA endoreduplication	0.160 %	-1.5402	0.61	0.64
GO:0098813	nuclear chromosome segregation	0.436 %	-2.9080	0.61	0.92
GO:0006261	DNA-dependent DNA replication	0.419 %	-1.3743	0.86	0.84
GO:0045839	negative regulation of mitotic nuclear division	0.043 %	-3.5234	0.48	0.97
GO:0045841	negative regulation of mitotic metaphase/anaphase transition	0.039 %	-3.5234	0.46	1.00
GO:0019220	regulation of phosphate metabolic process	0.561 %	-6.1922	0.75	0.98
GO:0044772	mitotic cell cycle phase transition	0.190 %	-6.8349	0.62	0.97
GO:0044770	cell cycle phase transition	0.207 %	-6.8349	0.65	0.65
GO:0044784	metaphase/anaphase transition of cell cycle	0.065 %	-3.1454	0.48	0.97
GO:0045930	negative regulation of mitotic cell cycle	0.108 %	-2.8307	0.55	0.90
GO:0044786	cell cycle DNA replication	0.181 %	-1.5350	0.61	0.86
GO:0071900	regulation of protein serine/threonine kinase activity	0.242 %	-8.4448	0.70	0.86
GO:0044839	cell cycle G2/M phase transition	0.091 %	-5.1369	0.64	0.91
GO:0007346	regulation of mitotic cell cycle	0.306 %	-6.1022	0.57	0.86
GO:0071174	mitotic spindle checkpoint	0.039 %	-3.5234	0.46	1.00
GO:0071173	spindle assembly checkpoint	0.039 %	-3.5234	0.59	1.00
GO:0009556	microsporogenesis	0.099 %	-3.0607	0.60	0.97
GO:0000819	sister chromatid segregation	0.285 %	-3.5407	0.53	0.90
GO:0051306	mitotic sister chromatid separation	0.065 %	-3.1454	0.55	0.86
GO:0051321	meiotic cell cycle	0.716 %	-3.4756	0.63	0.74
GO:1902099	regulation of metaphase/anaphase transition of cell cycle	0.065 %	-3.1454	0.47	1.00
GO:1902100	negative regulation of metaphase/anaphase transition of cell cycle	0.039 %	-3.5234	0.47	0.89
GO:0010564	regulation of cell cycle process	0.384 %	-6.4671	0.56	0.79
GO:0043549	regulation of kinase activity	0.427 %	-7.4692	0.75	0.98
GO:2000816	negative regulation of mitotic sister chromatid separation	0.039 %	-3.5234	0.46	1.00
GO:0000910	cytokinesis	0.384 %	-1.7119	0.63	0.79
GO:0000911	cytokinesis by cell plate formation	0.194 %	-2.6274	0.63	0.82
GO:0000912	assembly of actomyosin apparatus involved in cytokinesis	0.009 %	-3.4120	0.65	0.66
GO:0000914	phragmoplast assembly	0.009 %	-3.4120	0.64	0.51
GO:1902749	regulation of cell cycle G2/M phase transition	0.082 %	-5.1369	0.58	0.98
GO:0031399	regulation of protein modification process	0.699 %	-6.9827	0.72	0.67
GO:0030071	regulation of mitotic metaphase/anaphase transition	0.065 %	-3.1454	0.47	0.97
GO:1901983	regulation of protein acetylation	0.056 %	-1.2731	0.75	0.70
GO:1901987	regulation of cell cycle phase transition	0.164 %	-7.6251	0.56	0.95
GO:1901988	negative regulation of cell cycle phase transition	0.073 %	-3.3136	0.55	0.93
GO:1902407	assembly of actomyosin apparatus involved in mitotic cytokinesis	0.009 %	-3.4120	0.64	1.00
GO:0010965	regulation of mitotic sister chromatid separation	0.065 %	-3.1454	0.47	1.00
GO:0033047	regulation of mitotic sister chromatid segregation	0.065 %	-3.1454	0.47	0.97
GO:0033048	negative regulation of mitotic sister chromatid segregation	0.039 %	-3.5234	0.46	1.00
GO:0032506	cytokinetic process	0.203 %	-2.5423	0.64	0.89
GO:0033045	regulation of sister chromatid segregation	0.082 %	-3.0723	0.47	0.98
GO:0033046	negative regulation of sister chromatid segregation	0.039 %	-3.5234	0.47	1.00
GO:1902410	mitotic cytokinetic process	0.203 %	-2.5423	0.63	0.99
GO:1901990	regulation of mitotic cell cycle phase transition	0.147 %	-7.6251	0.56	0.80
GO:1901991	negative regulation of mitotic cell cycle phase transition	0.073 %	-3.3136	0.54	0.96
GO:0061640	cytoskeleton-dependent cytokinesis	0.332 %	-1.7432	0.63	0.97
GO:0010948	negative regulation of cell cycle process	0.117 %	-2.5083	0.55	0.91
GO:0007093	mitotic cell cycle checkpoint	0.099 %	-3.0381	0.55	0.97
GO:0007094	mitotic spindle assembly checkpoint	0.039 %	-3.5234	0.46	0.96

GO:0051445	regulation of meiotic cell cycle	0.060 %	-3.5407	0.61	0.68
GO:0042325	regulation of phosphorylation	0.462 %	-7.1482	0.76	0.95
GO:0000075	cell cycle checkpoint	0.117 %	-4.1849	0.57	0.72
GO:0000070	mitotic sister chromatid segregation	0.207 %	-4.1849	0.53	0.82
GO:0051985	negative regulation of chromosome segregation	0.039 %	-3.5234	0.66	0.90
GO:0007091	metaphase/anaphase transition of mitotic cell cycle	0.065 %	-3.1454	0.47	1.00
GO:0000079	regulation of cyclin-dependent protein serine/threonine kinase activity	0.138 %	-9.7098	0.50	0.63
GO:0000077	DNA damage checkpoint	0.043 %	-1.4322	0.58	0.89
GO:0007067	mitotic nuclear division	0.699 %	-5.1786	0.52	0.93
GO:0031577	spindle checkpoint	0.039 %	-3.5234	0.59	0.84
GO:0031570	DNA integrity checkpoint	0.060 %	-1.3952	0.58	0.88
GO:0010389	regulation of G2/M transition of mitotic cell cycle	0.078 %	-5.1369	0.58	0.94
GO:0000086	G2/M transition of mitotic cell cycle	0.086 %	-5.1369	0.64	0.99
GO:1904029	regulation of cyclin-dependent protein kinase activity	0.138 %	-9.7098	0.49	0.83
GO:1903047	mitotic cell cycle process	1.083 %	-8.5082	0.59	0.77
GO:0022402	cell cycle process	1.713 %	-6.8428	0.61	0.84
GO:0045786	negative regulation of cell cycle	0.211 %	-2.1303	0.56	0.81
GO:0040020	regulation of meiotic nuclear division	0.047 %	-2.3922	0.51	0.71
GO:0048236	plant-type sporogenesis	0.147 %	-2.7820	0.59	0.70
GO:0007017	microtubule-based process	0.859 %	-2.4973	0.78	0.27
GO:0008216	spermidine metabolic process	0.056 %	-2.5447	0.87	0.28
GO:0044106	cellular amine metabolic process	0.306 %	-1.3620	0.86	0.87
GO:0006576	cellular biogenic amine metabolic process	0.255 %	-1.5248	0.86	0.94
GO:0008295	spermidine biosynthetic process	0.039 %	-2.7798	0.87	0.91
GO:0006595	polyamine metabolic process	0.095 %	-2.0975	0.87	0.82
GO:0006596	polyamine biosynthetic process	0.065 %	-2.4906	0.86	0.94
GO:0009309	amine biosynthetic process	0.186 %	-1.9599	0.86	0.76
GO:0042401	cellular biogenic amine biosynthetic process	0.186 %	-1.9599	0.85	0.90
GO:0009445	putrescine metabolic process	0.022 %	-1.5424	0.88	0.87
GO:0009446	putrescine biosynthetic process	0.017 %	-1.5683	0.87	0.86
GO:0016049	cell growth	1.519 %	-2.1895	0.76	0.29
GO:0042547	cell wall modification involved in multidimensional cell growth	0.017 %	-1.1116	0.74	0.56
GO:0060560	developmental growth involved in morphogenesis	1.157 %	-1.2934	0.80	0.83
GO:0051301	cell division	1.528 %	-5.9222	0.77	0.29
GO:0051567	histone H3-K9 methylation	0.065 %	-2.2507	0.70	0.30
GO:0034968	histone lysine methylation	0.298 %	-1.8993	0.68	0.86
GO:0018205	peptidyl-lysine modification	0.725 %	-1.2608	0.82	0.64
GO:1900111	positive regulation of histone H3-K9 dimethylation	0.017 %	-1.3952	0.61	0.91
GO:1900109	regulation of histone H3-K9 dimethylation	0.022 %	-1.3952	0.61	0.99
GO:0061647	histone H3-K9 modification	0.069 %	-2.2507	0.73	0.64
GO:0051570	regulation of histone H3-K9 methylation	0.039 %	-1.3004	0.60	0.96
GO:0016572	histone phosphorylation	0.026 %	-1.9906	0.73	0.60
GO:0016571	histone methylation	0.354 %	-1.5939	0.68	0.97
GO:0016570	histone modification	0.893 %	-1.8395	0.68	0.89
GO:0016569	covalent chromatin modification	1.273 %	-1.7397	0.68	0.69
GO:0018027	peptidyl-lysine dimethylation	0.022 %	-1.3952	0.84	0.71
GO:0018022	peptidyl-lysine methylation	0.350 %	-1.8828	0.81	0.93
GO:0051574	positive regulation of histone H3-K9 methylation	0.022 %	-1.3451	0.60	0.92
GO:0006479	protein methylation	0.475 %	-1.4757	0.81	0.97
GO:0036123	histone H3-K9 dimethylation	0.022 %	-1.3952	0.72	1.00
GO:0010148	transpiration	0.026 %	-1.9510	0.89	0.30
GO:0006833	water transport	0.194 %	-1.6764	0.88	0.86
GO:0010185	regulation of cellular defense response	0.013 %	-3.1454	0.87	0.30
GO:0051245	negative regulation of cellular defense response	0.004 %	-3.1454	0.83	0.89
GO:0010623	programmed cell death involved in cell development	0.035 %	-1.1898	0.75	0.66
GO:0009626	plant-type hypersensitive response	0.341 %	-1.4570	0.75	0.86
GO:0060548	negative regulation of cell death	0.112 %	-1.4079	0.68	0.82
GO:0043069	negative regulation of programmed cell death	0.082 %	-1.6340	0.69	0.84
GO:0043067	regulation of programmed cell death	0.216 %	-1.1945	0.73	0.90
GO:0012501	programmed cell death	0.600 %	-1.2289	0.77	0.90
GO:0010363	regulation of plant-type hypersensitive response	0.060 %	-2.3184	0.73	0.56
GO:0034050	host programmed cell death induced by symbiont	0.345 %	-1.4079	0.77	0.90
GO:0080135	regulation of cellular response to stress	0.250 %	-1.6340	0.81	0.63
GO:0007267	cell-cell signaling	0.371 %	-1.1206	0.79	0.31
GO:1901698	response to nitrogen compound	1.174 %	-1.1322	0.97	0.31
GO:0010430	fatty acid omega-oxidation	0.004 %	-1.7292	0.82	0.32
GO:0046854	phosphatidylinositol phosphorylation	0.160 %	-1.0035	0.76	0.64
GO:0046834	lipid phosphorylation	0.173 %	-1.0035	0.76	0.83
GO:0048869	cellular developmental process	4.148 %	-3.8952	0.69	0.33

GO:0044707	single-multicellular organism process	10.699 %	-1.5566	0.84	0.86
GO:0031325	positive regulation of cellular metabolic process	2.741 %	-1.0263	0.74	0.95
GO:0044767	single-organism developmental process	12.123 %	-2.6465	0.79	0.68
GO:0010604	positive regulation of macromolecule metabolic process	2.672 %	-1.0602	0.76	0.66
GO:0010054	trichoblast differentiation	0.427 %	-1.1164	0.70	0.82
GO:0007275	multicellular organism development	10.423 %	-1.6063	0.78	0.82
GO:0048468	cell development	1.217 %	-1.7530	0.71	0.72
GO:0000902	cell morphogenesis	1.467 %	-1.9165	0.59	0.78
GO:0000904	cell morphogenesis involved in differentiation	0.880 %	-1.8073	0.61	0.91
GO:0009653	anatomical structure morphogenesis	3.500 %	-2.0226	0.89	0.55
GO:0010015	root morphogenesis	0.967 %	-1.2319	0.80	0.74
GO:0032989	cellular component morphogenesis	1.618 %	-2.1867	0.59	0.51
GO:0010455	positive regulation of cell fate commitment	0.013 %	-1.4520	0.73	0.72
GO:0010453	regulation of cell fate commitment	0.047 %	-1.4133	0.72	0.52
GO:0060776	simple leaf morphogenesis	0.022 %	-1.3004	0.85	0.51
GO:0030154	cell differentiation	3.332 %	-1.6409	0.68	0.81
GO:0009826	unidimensional cell growth	1.010 %	-1.5102	0.60	0.92
GO:0048856	anatomical structure development	11.722 %	-2.4511	0.88	0.85
GO:0045597	positive regulation of cell differentiation	0.030 %	-1.0696	0.71	0.76
GO:0048646	anatomical structure formation involved in morphogenesis	0.755 %	-1.5462	0.89	0.72
GO:0048653	anther development	0.224 %	-1.1033	0.83	0.53
GO:0051094	positive regulation of developmental process	0.436 %	-1.1877	0.78	0.54
GO:0006949	syncytium formation	0.043 %	-1.0103	0.77	0.54
GO:0048229	gametophyte development	1.653 %	-2.6225	0.82	0.51
GO:0009932	cell tip growth	0.630 %	-1.2053	0.61	0.87
GO:0051235	maintenance of location	0.302 %	-1.4570	0.87	0.33
GO:0010256	endomembrane system organization	0.311 %	-1.1945	0.80	0.34
GO:0030245	cellulose catabolic process	0.112 %	-2.3619	0.85	0.35
GO:0000272	polysaccharide catabolic process	0.777 %	-1.4899	0.88	0.75
GO:0051275	beta-glucan catabolic process	0.112 %	-2.3619	0.85	0.89
GO:0051273	beta-glucan metabolic process	0.393 %	-1.4348	0.86	0.75
GO:0044247	cellular polysaccharide catabolic process	0.229 %	-1.6340	0.85	0.90
GO:0044275	cellular carbohydrate catabolic process	0.298 %	-1.3305	0.86	0.82
GO:0044042	glucan metabolic process	0.962 %	-1.2670	0.90	0.82
GO:0030243	cellulose metabolic process	0.337 %	-1.7017	0.86	0.88
GO:0009251	glucan catabolic process	0.229 %	-1.6340	0.89	0.80
GO:0006073	cellular glucan metabolic process	0.937 %	-1.2670	0.85	0.90
GO:0005976	polysaccharide metabolic process	1.899 %	-1.0007	0.90	0.54
GO:0051246	regulation of protein metabolic process	2.123 %	-4.5449	0.77	0.36
GO:0080090	regulation of primary metabolic process	13.681 %	-2.1393	0.76	0.56
GO:0031323	regulation of cellular metabolic process	13.906 %	-1.7392	0.72	0.83
GO:0019222	regulation of metabolic process	14.847 %	-1.1733	0.79	0.63
GO:0050794	regulation of cellular process	22.244 %	-1.6796	0.76	0.61
GO:0050789	regulation of biological process	24.333 %	-1.4571	0.83	0.61
GO:0060255	regulation of macromolecule metabolic process	14.091 %	-2.1186	0.74	0.82
GO:0042044	fluid transport	0.194 %	-1.6764	0.88	0.38
GO:0042128	nitrate assimilation	0.181 %	-1.0539	0.77	0.39
GO:0042126	nitrate metabolic process	0.181 %	-1.0539	0.77	0.98
GO:0080175	phragmoplast microtubule organization	0.017 %	-3.2253	0.71	0.39
GO:0007018	microtubule-based movement	0.281 %	-2.3507	0.79	0.65
GO:0031109	microtubule polymerization or depolymerization	0.108 %	-1.0943	0.68	0.75
GO:0046785	microtubule polymerization	0.082 %	-1.2731	0.66	0.73
GO:0000226	microtubule cytoskeleton organization	0.518 %	-1.6883	0.64	0.85
GO:0080167	response to karrikin	0.531 %	-2.4271	0.97	0.39
GO:0048017	inositol lipid-mediated signaling	0.022 %	-1.4729	0.82	0.40
GO:0051128	regulation of cellular component organization	1.467 %	-4.2033	0.70	0.40
GO:1990778	protein localization to cell periphery	0.022 %	-2.5447	0.96	0.41
GO:2001251	negative regulation of chromosome organization	0.043 %	-3.4659	0.62	0.42
GO:0048523	negative regulation of cellular process	2.719 %	-1.3969	0.73	0.56
GO:2000756	regulation of peptidyl-lysine acetylation	0.056 %	-1.2731	0.73	1.00
GO:0010639	negative regulation of organelle organization	0.138 %	-2.0160	0.61	0.87
GO:0051784	negative regulation of nuclear division	0.052 %	-3.4120	0.62	0.86
GO:0030261	chromosome condensation	0.052 %	-1.6918	0.77	0.58
GO:2000757	negative regulation of peptidyl-lysine acetylation	0.039 %	-1.3297	0.68	1.00
GO:1901984	negative regulation of protein acetylation	0.039 %	-1.3297	0.70	0.95
GO:0031060	regulation of histone methylation	0.086 %	-1.2007	0.58	0.92
GO:0031057	negative regulation of histone modification	0.047 %	-1.3297	0.57	0.95
GO:0031058	positive regulation of histone modification	0.069 %	-1.3148	0.59	0.87
GO:0031056	regulation of histone modification	0.134 %	-1.0315	0.58	0.88
GO:0033044	regulation of chromosome organization	0.099 %	-2.9429	0.66	0.69

GO:0033043	*regulation of organelle organization*	*0.617 %*	*-2.9337*	*0.63*	*0.65*
GO:0031062	*positive regulation of histone methylation*	*0.047 %*	*-1.3148*	*0.59*	*0.94*
GO:0007088	*regulation of mitotic nuclear division*	*0.129 %*	*-3.1081*	*0.48*	*0.89*
GO:1905269	*positive regulation of chromatin organization*	*0.086 %*	*-1.2353*	*0.65*	*0.81*
GO:0001558	*regulation of cell growth*	*0.293 %*	*-1.5839*	*0.60*	*0.76*
GO:0007076	*mitotic chromosome condensation*	*0.022 %*	*-1.6918*	*0.58*	*0.76*
GO:1905268	*negative regulation of chromatin organization*	*0.065 %*	*-1.1029*	*0.62*	*0.88*
GO:0035065	*regulation of histone acetylation*	*0.056 %*	*-1.2731*	*0.59*	*0.98*
GO:0035067	*negative regulation of histone acetylation*	*0.039 %*	*-1.3297*	*0.56*	*0.79*
GO:0051129	*negative regulation of cellular component organization*	*0.211 %*	*-2.7913*	*0.63*	*0.74*
GO:0051258	protein polymerization	0.263 %	-1.2137	0.77	0.43
GO:0006325	*chromatin organization*	*1.675 %*	*-1.0088*	*0.74*	*0.57*
GO:0045229	external encapsulating structure organization	2.577 %	-2.6510	0.76	0.44
GO:0016043	*cellular component organization*	*13.263 %*	*-7.4841*	*0.73*	*0.58*
GO:0044085	*cellular component biogenesis*	*5.783 %*	*-1.0579*	*0.79*	*0.68*
GO:0006996	*organelle organization*	*6.439 %*	*-3.0679*	*0.74*	*0.69*
GO:0009938	negative regulation of gibberellic acid mediated signaling pathway	0.039 %	-1.1898	0.74	0.45
GO:0031348	*negative regulation of defense response*	*0.203 %*	*-1.1882*	*0.79*	*0.70*
GO:0009863	*salicylic acid mediated signaling pathway*	*0.211 %*	*-1.0897*	*0.78*	*0.54*
GO:0009647	skotomorphogenesis	0.026 %	-1.1898	0.85	0.46
GO:0010332	response to gamma radiation	0.043 %	-3.9472	0.97	0.48
GO:0044248	cellular catabolic process	5.939 %	-1.0619	0.86	0.48
GO:0009753	response to jasmonic acid	0.919 %	-1.1488	0.97	0.48
GO:0009555	pollen development	1.230 %	-2.2393	0.82	0.49
GO:0055046	*microgametogenesis*	*0.216 %*	*-1.3343*	*0.84*	*0.77*

Biological Process (200) Tag Clouds

Scatterplot & Table Interactive Graph TreeMap

GROUP 30

Hide/show dispensable GO terms

Export results to text table (CSV)

Make R script for plotting

term ID	description	frequency	pin?	log_{10} p-value	uniqueness	dispensability
GO:0009624	response to nematode	0.306 %		-3.0170	0.93	0.00
GO:0046463	acylglycerol biosynthetic process	0.117 %		-4.4648	0.69	0.00
GO:0046339	diacylglycerol metabolic process	0.022 %		-1.5931	0.75	0.86
GO:0045017	glycerolipid biosynthetic process	0.445 %		-3.1533	0.68	0.79
GO:0006651	diacylglycerol biosynthetic process	0.017 %		-2.2877	0.73	0.88
GO:0046460	neutral lipid biosynthetic process	0.117 %		-4.4648	0.70	0.96
GO:0019432	triglyceride biosynthetic process	0.104 %		-2.8231	0.70	0.99
GO:0046486	glycerolipid metabolic process	0.764 %		-2.1007	0.72	0.58
GO:0006639	acylglycerol metabolic process	0.147 %		-3.7382	0.72	0.98
GO:0006638	neutral lipid metabolic process	0.147 %		-3.7382	0.75	0.50
GO:0006641	triglyceride metabolic process	0.129 %		-2.4627	0.72	0.99
GO:0080145	cysteine homeostasis	0.004 %		-3.7097	0.86	0.00
GO:0055063	sulfate ion homeostasis	0.009 %		-1.6889	0.86	0.74
GO:0050891	multicellular organismal water homeostasis	0.013 %		-1.7464	0.79	0.55
GO:0042592	homeostatic process	2.637 %		-1.3617	0.81	0.69
GO:0050801	ion homeostasis	1.299 %		-1.2716	0.81	0.84
GO:0048878	chemical homeostasis	1.765 %		-2.0096	0.81	0.50
GO:0048871	multicellular organismal homeostasis	0.022 %		-1.7464	0.79	0.57
GO:0072505	divalent inorganic anion homeostasis	0.086 %		-1.6889	0.83	0.86

GO:0010817	regulation of hormone levels	1.109 %	-1.0878	0.82	0.62
GO:0080144	amino acid homeostasis	0.060 %	-2.6158	0.84	0.71
GO:0009399	nitrogen fixation	0.017 %	-3.6049	0.92	0.03
GO:0036369	transcription factor catabolic process	0.004 %	-1.8128	0.86	0.05
GO:0048645	animal organ formation	0.004 %	-2.2130	0.86	0.06
GO:0010160	formation of animal organ boundary	0.055 %	-1.2119	0.84	1.15
GO:2000027	regulation of organ morphogenesis	0.004 %	-1.2034	0.80	1.15
GO:0003156	regulation of animal organ formation	0.004 %	-1.3083	0.80	1.00
GO:0009887	animal organ morphogenesis	0.004 %	-1.8940	0.86	1.00
GO:0006949	syncytium formation	0.043 %	-1.1270	0.80	0.54
GO:0015671	oxygen transport	0.009 %	-4.4633	0.91	0.06
GO:0005975	carbohydrate metabolic process	4.670 %	-1.3846	0.92	0.08
GO:0090322	regulation of superoxide metabolic process	0.017 %	-1.7167	0.82	0.10
GO:0006801	superoxide metabolic process	0.078 %	-1.2872	0.91	0.62
GO:0010310	regulation of hydrogen peroxide metabolic process	0.056 %	-1.3306	0.81	0.77
GO:0071941	nitrogen cycle metabolic process	0.207 %	-2.3447	0.91	0.13
GO:0007263	nitric oxide mediated signal transduction	0.009 %	-1.6151	0.81	0.13
GO:0009643	photosynthetic acclimation	0.035 %	-1.4647	0.94	0.15
GO:0009642	response to light intensity	0.587 %	-1.2086	0.94	0.53
GO:0071484	cellular response to light intensity	0.043 %	-1.2770	0.90	0.65
GO:0071486	cellular response to high light intensity	0.026 %	-1.4343	0.91	0.63
GO:0009750	response to fructose	0.069 %	-1.8281	0.94	0.15
GO:0009746	response to hexose	0.220 %	-1.2364	0.94	0.90
GO:0034284	response to monosaccharide	0.237 %	-1.1899	0.94	0.79
GO:0051983	regulation of chromosome segregation	0.082 %	-1.4976	0.67	0.16
GO:0051304	chromosome separation	0.147 %	-1.3083	0.66	0.76
GO:0065008	regulation of biological quality	5.991 %	-1.8986	0.85	0.18
GO:0007067	mitotic nuclear division	0.699 %	-1.7836	0.61	0.19
GO:0000280	nuclear division	1.079 %	-1.0812	0.79	0.91
GO:0045839	negative regulation of mitotic nuclear division	0.043 %	-1.7167	0.55	0.97
GO:0045841	negative regulation of mitotic metaphase/anaphase transition	0.039 %	-1.7167	0.52	1.00
GO:0044772	mitotic cell cycle phase transition	0.190 %	-1.0628	0.65	0.97
GO:0044770	cell cycle phase transition	0.207 %	-1.0628	0.68	0.72
GO:0044784	metaphase/anaphase transition of cell cycle	0.065 %	-1.5333	0.54	0.97
GO:0045930	negative regulation of mitotic cell cycle	0.108 %	-1.3792	0.57	0.87
GO:0071174	mitotic spindle checkpoint	0.039 %	-1.7167	0.52	1.00
GO:0071173	spindle assembly checkpoint	0.039 %	-1.7167	0.60	1.00
GO:0000819	sister chromatid segregation	0.285 %	-1.0397	0.61	0.90
GO:0051306	mitotic sister chromatid separation	0.065 %	-1.5333	0.62	0.73
GO:0051783	regulation of nuclear division	0.181 %	-1.0024	0.70	0.79
GO:1902099	regulation of metaphase/anaphase transition of cell cycle	0.065 %	-1.5333	0.54	1.00
GO:1902100	negative regulation of metaphase/anaphase transition of cell cycle	0.039 %	-1.7167	0.53	0.90
GO:2000816	negative regulation of mitotic sister chromatid separation	0.039 %	-1.7167	0.53	1.00
GO:0030071	regulation of mitotic metaphase/anaphase transition	0.065 %	-1.5333	0.53	0.97
GO:1901987	regulation of cell cycle phase transition	0.164 %	-1.2119	0.58	0.95
GO:1901988	negative regulation of cell cycle phase transition	0.073 %	-1.6151	0.57	0.82
GO:0010965	regulation of mitotic sister chromatid separation	0.065 %	-1.5333	0.54	1.00
GO:0033047	regulation of mitotic sister chromatid segregation	0.065 %	-1.5333	0.54	0.97
GO:0033048	negative regulation of mitotic sister chromatid segregation	0.039 %	-1.7167	0.53	1.00
GO:0033045	regulation of sister chromatid segregation	0.082 %	-1.4976	0.54	0.98
GO:0033046	negative regulation of sister chromatid segregation	0.039 %	-1.7167	0.54	1.00
GO:1901990	regulation of mitotic cell cycle phase transition	0.147 %	-1.2119	0.58	0.93
GO:1901991	negative regulation of mitotic cell cycle phase transition	0.073 %	-1.6151	0.56	0.96
GO:0010948	negative regulation of cell cycle process	0.117 %	-1.2206	0.57	0.91
GO:0007093	mitotic cell cycle checkpoint	0.099 %	-1.4809	0.56	0.97
GO:0007094	mitotic spindle assembly checkpoint	0.039 %	-1.7167	0.52	0.96
GO:0000075	cell cycle checkpoint	0.117 %	-1.2478	0.58	0.90
GO:0000070	mitotic sister chromatid segregation	0.207 %	-1.2478	0.61	0.86
GO:0051985	negative regulation of chromosome segregation	0.039 %	-1.7167	0.62	0.90
GO:0007091	metaphase/anaphase transition of mitotic cell cycle	0.065 %	-1.5333	0.53	1.00
GO:0031577	spindle checkpoint	0.039 %	-1.7167	0.60	0.56
GO:0045786	negative regulation of cell cycle	0.211 %	-1.0342	0.59	0.68
GO:0009920	cell plate formation involved in plant-type cell wall biogenesis	0.030 %	-1.1001	0.68	0.69
GO:0008037	cell recognition	0.220 %	-1.0953	0.81	0.20
GO:1990778	protein localization to cell periphery	0.022 %	-1.2386	0.97	0.21
GO:0005984	disaccharide metabolic process	0.285 %	-3.2975	0.72	0.22
GO:0046351	disaccharide biosynthetic process	0.155 %	-1.6729	0.70	0.91
GO:0019310	inositol catabolic process	0.017 %	-1.9878	0.66	0.54
GO:0010115	regulation of abscisic acid biosynthetic process	0.030 %	-1.1201	0.61	0.63

GO:0044262	cellular carbohydrate metabolic process	1.817 %	-1.2962	0.86	0.59
GO:0006020	inositol metabolic process	0.069 %	-1.4198	0.69	0.75
GO:0046174	polyol catabolic process	0.060 %	-1.0454	0.70	0.92
GO:0046164	alcohol catabolic process	0.082 %	-1.0127	0.69	0.85
GO:0009311	oligosaccharide metabolic process	0.466 %	-2.5053	0.76	0.66
GO:0009312	oligosaccharide biosynthetic process	0.255 %	-1.4913	0.73	0.90
GO:0005991	trehalose metabolic process	0.104 %	-2.1946	0.74	0.85
GO:0005992	trehalose biosynthetic process	0.095 %	-2.2320	0.70	0.83
GO:0005985	sucrose metabolic process	0.164 %	-1.7945	0.73	0.88
GO:0009605	response to external stimulus	6.366 %	-1.3248	0.95	0.23
GO:0010188	response to microbial phytotoxin	0.026 %	-1.5151	0.96	0.23
GO:0048513	animal organ development	0.004 %	-1.5212	0.90	0.24
GO:0007009	plasma membrane organization	0.022 %	-1.2034	0.79	0.26
GO:0090002	establishment of protein localization to plasma membrane	0.013 %	-1.2386	0.78	1.00
GO:0072661	protein targeting to plasma membrane	0.013 %	-1.2386	0.78	0.66
GO:0072659	protein localization to plasma membrane	0.013 %	-1.2386	0.78	0.94
GO:0009856	pollination	1.075 %	-2.1096	0.92	0.26
GO:0044706	multi-multicellular organism process	1.075 %	-2.1096	0.93	0.72
GO:0044703	multi-organism reproductive process	1.610 %	-1.6309	0.95	0.76
GO:0048544	recognition of pollen	0.211 %	-1.0994	0.75	0.84
GO:0043901	negative regulation of multi-organism process	0.393 %	-1.3542	0.76	0.65
GO:0009875	pollen-pistil interaction	0.237 %	-1.0933	0.88	0.85
GO:0009846	pollen germination	0.203 %	-1.2783	0.85	0.84
GO:1901605	alpha-amino acid metabolic process	1.329 %	-3.1566	0.58	0.27
GO:0019279	L-methionine biosynthetic process from L-homoserine via cystathionine	0.004 %	-2.1122	0.65	0.93
GO:0019281	L-methionine biosynthetic process from homoserine via O-succinyl-L-homoserine and cystathionine	0.004 %	-2.1122	0.65	0.71
GO:0071265	L-methionine biosynthetic process	0.052 %	-1.4343	0.61	0.85
GO:0071266	'de novo' L-methionine biosynthetic process	0.009 %	-1.8503	0.64	0.78
GO:0019760	glucosinolate metabolic process	0.514 %	-1.4086	0.60	0.59
GO:0016053	organic acid biosynthetic process	2.296 %	-1.3179	0.60	0.73
GO:0019757	glycosinolate metabolic process	0.514 %	-1.4086	0.60	0.99
GO:0009074	aromatic amino acid family catabolic process	0.065 %	-1.7783	0.60	0.67
GO:0009070	serine family amino acid biosynthetic process	0.177 %	-2.1416	0.60	0.91
GO:0009069	serine family amino acid metabolic process	0.276 %	-2.6392	0.62	0.76
GO:0009066	aspartate family amino acid metabolic process	0.293 %	-1.6729	0.62	0.77
GO:0009067	aspartate family amino acid biosynthetic process	0.220 %	-1.9867	0.59	0.75
GO:0009064	glutamine family amino acid metabolic process	0.367 %	-2.0969	0.61	0.79
GO:0009086	methionine biosynthetic process	0.112 %	-1.0180	0.59	0.92
GO:0009092	homoserine metabolic process	0.026 %	-2.1122	0.66	0.75
GO:1901607	alpha-amino acid biosynthetic process	0.833 %	-3.3644	0.57	0.86
GO:0008652	cellular amino acid biosynthetic process	0.915 %	-1.7280	0.58	0.87
GO:0019344	cysteine biosynthetic process	0.104 %	-2.4016	0.60	0.93
GO:0006541	glutamine metabolic process	0.168 %	-1.8755	0.63	0.73
GO:0070982	L-asparagine metabolic process	0.013 %	-2.1122	0.66	0.88
GO:0070981	L-asparagine biosynthetic process	0.013 %	-2.1122	0.65	0.77
GO:0006534	cysteine metabolic process	0.134 %	-2.2612	0.61	0.84
GO:0006535	cysteine biosynthetic process from serine	0.065 %	-2.8430	0.61	0.67
GO:0006529	asparagine biosynthetic process	0.039 %	-1.7464	0.62	0.93
GO:0006528	asparagine metabolic process	0.039 %	-1.6151	0.65	0.83
GO:0006520	cellular amino acid metabolic process	1.869 %	-2.2486	0.61	0.72
GO:0006572	tyrosine catabolic process	0.022 %	-1.8128	0.62	0.74
GO:0006570	tyrosine metabolic process	0.047 %	-1.2295	0.65	0.77
GO:0006563	L-serine metabolic process	0.129 %	-2.1416	0.62	0.89
GO:0006561	proline biosynthetic process	0.043 %	-1.0233	0.64	0.79
GO:0044283	small molecule biosynthetic process	2.827 %	-1.5191	0.69	0.62
GO:0050878	regulation of body fluid levels	0.013 %	-1.6151	0.87	0.28
GO:0015669	gas transport	0.009 %	-3.6557	0.91	0.30
GO:0010222	stem vascular tissue pattern formation	0.026 %	-1.6889	0.88	0.30
GO:0009270	response to humidity	0.009 %	-1.6629	0.95	0.31
GO:0033528	S-methylmethionine cycle	0.004 %	-1.9369	0.82	0.31
GO:0033477	S-methylmethionine metabolic process	0.004 %	-1.9369	0.82	0.58
GO:0006749	glutathione metabolic process	0.069 %	-1.0454	0.79	0.67
GO:0010037	response to carbon dioxide	0.030 %	-1.4492	0.95	0.33
GO:0001560	regulation of cell growth by extracellular stimulus	0.009 %	-1.9878	0.68	0.34
GO:0043617	cellular response to sucrose starvation	0.017 %	-1.6151	0.90	0.72
GO:0031668	cellular response to extracellular stimulus	0.691 %	-1.6671	0.87	0.69
GO:0006032	chitin catabolic process	0.073 %	-1.3542	0.74	0.39
GO:0046348	amino sugar catabolic process	0.073 %	-1.3542	0.83	0.91
GO:1901071	glucosamine-containing compound metabolic process	0.078 %	-1.3083	0.89	0.91
GO:1901072	glucosamine-containing compound catabolic process	0.073 %	-1.3542	0.83	1.00

GO:0006030	chitin metabolic process	0.073 %	-1.3423	0.79	0.99
GO:0006026	aminoglycan catabolic process	0.073 %	-1.3542	0.75	0.98
GO:0009691	cytokinin biosynthetic process	0.091 %	-2.9274	0.69	0.40
GO:0009690	cytokinin metabolic process	0.168 %	-2.0760	0.69	0.88
GO:0010541	acropetal auxin transport	0.013 %	-1.0810	0.80	0.56
GO:0034754	cellular hormone metabolic process	0.263 %	-2.0375	0.78	0.76
GO:0042445	hormone metabolic process	0.695 %	-1.1354	0.79	0.82
GO:0042446	hormone biosynthetic process	0.514 %	-1.8111	0.76	0.88
GO:0044711	single-organism biosynthetic process	7.549 %	-1.4638	0.75	0.40
GO:0010189	vitamin E biosynthetic process	0.043 %	-1.6151	0.68	0.40
GO:0006775	fat-soluble vitamin metabolic process	0.047 %	-1.6151	0.77	0.73
GO:0042362	fat-soluble vitamin biosynthetic process	0.047 %	-1.6151	0.69	0.99
GO:0042360	vitamin E metabolic process	0.043 %	-1.6151	0.71	0.99
GO:1900425	negative regulation of defense response to bacterium	0.043 %	-1.7464	0.77	0.42
GO:0031348	negative regulation of defense response	0.203 %	-1.4053	0.77	0.71
GO:0002832	negative regulation of response to biotic stimulus	0.065 %	-1.3542	0.78	0.77
GO:1900424	regulation of defense response to bacterium	0.129 %	-1.1951	0.82	0.83
GO:0032102	negative regulation of response to external stimulus	0.065 %	-1.3542	0.77	0.76
GO:0032436	positive regulation of proteasomal ubiquitin-dependent protein catabolic process	0.207 %	-1.8128	0.70	0.46
GO:0045862	positive regulation of proteolysis	0.272 %	-1.8128	0.74	0.84
GO:0031331	positive regulation of cellular catabolic process	0.289 %	-1.8128	0.70	0.98
GO:1901800	positive regulation of proteasomal protein catabolic process	0.259 %	-1.8128	0.69	0.98
GO:0032434	regulation of proteasomal ubiquitin-dependent protein catabolic process	0.211 %	-1.8128	0.70	0.96
GO:0061136	regulation of proteasomal protein catabolic process	0.268 %	-1.8128	0.69	0.99
GO:1903364	positive regulation of cellular protein catabolic process	0.263 %	-1.8128	0.69	0.99
GO:1903362	regulation of cellular protein catabolic process	0.272 %	-1.8128	0.70	0.95
GO:1901485	positive regulation of transcription factor catabolic process	0.004 %	-1.8128	0.75	0.76
GO:1901483	regulation of transcription factor catabolic process	0.004 %	-1.8128	0.75	1.00
GO:0030162	regulation of proteolysis	0.591 %	-1.2976	0.76	0.67
GO:0009896	positive regulation of catabolic process	0.302 %	-1.1412	0.73	0.91
GO:1903052	positive regulation of proteolysis involved in cellular protein catabolic process	0.263 %	-1.8128	0.69	1.00
GO:1903050	regulation of proteolysis involved in cellular protein catabolic process	0.272 %	-1.8128	0.69	1.00
GO:0045732	positive regulation of protein catabolic process	0.276 %	-1.1412	0.71	0.99
GO:0006022	aminoglycan metabolic process	0.086 %	-1.2872	0.81	0.46
GO:0009308	amine metabolic process	0.522 %	-1.1268	0.82	0.47
GO:0006040	amino sugar metabolic process	0.138 %	-1.1559	0.90	0.48
GO:0071496	cellular response to external stimulus	0.712 %	-1.6299	0.93	0.48
GO:0009991	response to extracellular stimulus	0.777 %	-1.4782	0.93	0.53
GO:2001251	negative regulation of chromosome organization	0.043 %	-1.6889	0.66	0.49
GO:0051784	negative regulation of nuclear division	0.052 %	-1.6629	0.67	0.86
GO:0033044	regulation of chromosome organization	0.099 %	-1.4343	0.72	0.69
GO:0007088	regulation of mitotic nuclear division	0.129 %	-1.5151	0.55	0.89

Biological Process (223) Tag Clouds

Scatterplot & Table Interactive Graph TreeMap

GROUP 31

Tip: your resulting list of GO terms seems to be quite long. If you want to reduce it further, press the Back button in your browser and choose a different setting for the "allowed similarity" parameter.

Hide/show dispensable GO terms Export results to text table (CSV) Make R script for plotting

term ID	description	frequency	pin?	log_{10} p-value	uniqueness	dispensability
GO:0019748	secondary metabolic process	1.938 %		-5.1206	0.77	0.00
GO:0034755	iron ion transmembrane transport	0.073 %		-2.1320	0.96	0.00
GO:0006826	*iron ion transport*	*0.121 %*		*-1.0214*	*0.96*	*0.81*
GO:0006828	*manganese ion transport*	*0.082 %*		*-1.0702*	*0.96*	*0.78*
GO:0010163	*high-affinity potassium ion import*	*0.013 %*		*-1.6954*	*0.84*	*0.53*
GO:0034220	*ion transmembrane transport*	*2.529 %*		*-1.0782*	*0.96*	*0.56*
GO:0042391	regulation of membrane potential	0.138 %		-1.6580	0.94	0.00
GO:0044699	single-organism process	41.709 %		-2.7187	0.99	0.00
GO:0050896	response to stimulus	25.943 %		-2.3628	0.99	0.00
GO:0051704	multi-organism process	3.362 %		-1.4037	0.99	0.00
GO:0080167	response to karrikin	0.531 %		-4.4662	0.87	0.00
GO:0009821	alkaloid biosynthetic process	0.795 %		-2.6883	0.87	0.06
GO:0018027	peptidyl-lysine dimethylation	0.022 %		-1.3618	0.87	0.06
GO:0048513	animal organ development	0.004 %		-2.1546	0.86	0.07
GO:0009056	catabolic process	8.118 %		-2.3805	0.94	0.08
GO:0019439	aromatic compound catabolic process	0.898 %		-2.6541	0.82	0.09
GO:0044712	*single-organism catabolic process*	*2.676 %*		*-2.6265*	*0.72*	*0.55*

GO:1901575	organic substance catabolic process	7.402 %	-1.6808	0.84	0.74
GO:1901361	organic cyclic compound catabolic process	0.937 %	-2.4733	0.83	0.56
GO:2000379	positive regulation of reactive oxygen species metabolic process	0.047 %	-1.4186	0.87	0.13
GO:0010529	negative regulation of transposition	0.009 %	-1.2672	0.81	0.14
GO:0010528	regulation of transposition	0.009 %	-1.2672	0.83	0.96
GO:0030198	extracellular matrix organization	0.233 %	-1.0843	0.80	0.15
GO:0010215	cellulose microfibril organization	0.073 %	-1.1160	0.81	0.91
GO:0032196	transposition	0.013 %	-1.0291	0.86	0.15
GO:0030522	intracellular receptor signaling pathway	0.078 %	-1.9413	0.79	0.16
GO:0009624	response to nematode	0.306 %	-1.6339	0.90	0.18
GO:0043207	response to external biotic stimulus	4.981 %	-1.1886	0.87	0.94
GO:0051707	response to other organism	4.968 %	-1.1886	0.87	0.65
GO:0044763	single-organism cellular process	23.034 %	-1.5945	0.84	0.19
GO:0009947	centrolateral axis specification	0.004 %	-1.8325	0.86	0.22
GO:0003002	regionalization	0.617 %	-1.6276	0.81	0.64
GO:0097353	centrolateral pattern formation	0.004 %	-1.8325	0.86	0.50
GO:0009955	adaxial/abaxial pattern specification	0.151 %	-1.4933	0.83	0.84
GO:0090691	formation of plant organ boundary	0.069 %	-1.9605	0.83	0.22
GO:0090451	cotyledon boundary formation	0.009 %	-1.6954	0.85	0.87
GO:0010016	shoot system morphogenesis	0.708 %	-1.1577	0.80	0.53
GO:0048859	formation of anatomical boundary	0.069 %	-1.6255	0.84	0.67
GO:1990267	response to transition metal nanoparticle	2.017 %	-2.2570	0.87	0.22
GO:1901700	response to oxygen-containing compound	6.504 %	-1.6834	0.86	0.61
GO:0010035	response to inorganic substance	3.699 %	-1.1254	0.86	0.51
GO:0001101	response to acid chemical	5.011 %	-1.0972	0.86	0.64
GO:0043693	monoterpene biosynthetic process	0.017 %	-1.6237	0.73	0.22
GO:0051762	sesquiterpene biosynthetic process	0.009 %	-1.4394	0.74	0.86
GO:0051761	sesquiterpene metabolic process	0.009 %	-1.4394	0.75	0.81
GO:0010686	tetracyclic triterpenoid biosynthetic process	0.004 %	-1.2400	0.74	0.52
GO:0010685	tetracyclic triterpenoid metabolic process	0.004 %	-1.2400	0.75	0.79
GO:0010683	tricyclic triterpenoid metabolic process	0.013 %	-1.1677	0.74	0.84
GO:0043692	monoterpene metabolic process	0.017 %	-1.6237	0.74	0.85
GO:0019742	pentacyclic triterpenoid metabolic process	0.004 %	-1.0702	0.75	0.84
GO:0019745	pentacyclic triterpenoid biosynthetic process	0.004 %	-1.2400	0.74	0.81
GO:0045338	farnesyl diphosphate metabolic process	0.078 %	-1.0970	0.72	0.80
GO:0016099	monoterpenoid biosynthetic process	0.004 %	-1.3988	0.75	0.52
GO:0016098	monoterpenoid metabolic process	0.004 %	-1.3988	0.77	0.51
GO:0016104	triterpenoid biosynthetic process	0.030 %	-1.0138	0.71	0.93
GO:0016106	sesquiterpenoid biosynthetic process	0.138 %	-1.2270	0.70	0.84
GO:0016114	terpenoid biosynthetic process	0.591 %	-1.5485	0.67	0.71
GO:0006714	sesquiterpenoid metabolic process	0.181 %	-1.1258	0.71	0.86
GO:0006721	terpenoid metabolic process	0.699 %	-1.3401	0.69	0.94
GO:0008299	isoprenoid biosynthetic process	0.738 %	-1.3084	0.67	0.96
GO:0046246	terpene biosynthetic process	0.043 %	-1.0702	0.71	0.89
GO:0010263	tricyclic triterpenoid biosynthetic process	0.009 %	-1.2534	0.73	0.87
GO:1901983	regulation of protein acetylation	0.056 %	-1.2400	0.82	0.23
GO:0032269	negative regulation of cellular protein metabolic process	0.673 %	-1.1108	0.80	0.77
GO:2000756	regulation of peptidyl-lysine acetylation	0.056 %	-1.2400	0.81	1.00
GO:0051248	negative regulation of protein metabolic process	0.678 %	-1.1108	0.82	0.60
GO:1900111	positive regulation of histone H3-K9 dimethylation	0.017 %	-1.3618	0.79	0.89
GO:1900109	regulation of histone H3-K9 dimethylation	0.022 %	-1.3618	0.79	0.99
GO:2000757	negative regulation of peptidyl-lysine acetylation	0.039 %	-1.2964	0.80	1.00
GO:1901984	negative regulation of protein acetylation	0.039 %	-1.2964	0.80	0.95
GO:0031060	regulation of histone methylation	0.086 %	-1.1677	0.78	0.92
GO:0031057	negative regulation of histone modification	0.047 %	-1.2964	0.78	0.95
GO:0031058	positive regulation of histone modification	0.069 %	-1.2816	0.78	0.87
GO:0031062	positive regulation of histone methylation	0.047 %	-1.2816	0.78	0.94
GO:0051570	regulation of histone H3-K9 methylation	0.039 %	-1.2672	0.79	0.87
GO:1905269	positive regulation of chromatin organization	0.086 %	-1.2023	0.84	0.81
GO:1905268	negative regulation of chromatin organization	0.065 %	-1.0702	0.83	0.84
GO:0051574	positive regulation of histone H3-K9 methylation	0.022 %	-1.3118	0.79	0.92
GO:0035065	regulation of histone acetylation	0.056 %	-1.2400	0.78	0.98
GO:0035067	negative regulation of histone acetylation	0.039 %	-1.2964	0.77	0.75
GO:0036123	histone H3-K9 dimethylation	0.022 %	-1.3618	0.84	1.00
GO:0043062	extracellular structure organization	0.233 %	-1.0843	0.81	0.29
GO:0009607	response to biotic stimulus	5.158 %	-2.0630	0.89	0.29
GO:0048508	embryonic meristem development	0.125 %	-2.1210	0.81	0.29
GO:0010214	seed coat development	0.168 %	-1.1543	0.81	0.56
GO:0010067	procambium histogenesis	0.039 %	-1.9224	0.82	0.64
GO:0010065	primary meristem tissue development	0.043 %	-1.7864	0.82	0.86

GO:0009698	phenylpropanoid metabolic process	0.505 %	-5.4016	0.68	0.30
GO:0090487	secondary metabolite catabolic process	0.199 %	-1.2400	0.69	0.67
GO:1902123	(-)-pinoresinol catabolic process	0.081 %	-2.4322	0.56	1.00
GO:1902124	(+)-pinoresinol metabolic process	1.218 %	-1.9567	0.50	1.92
GO:1902127	(-)-lariciresinol metabolic process	1.258 %	-2.1320	0.50	1.47
GO:1902128	(-)-lariciresinol catabolic process	0.087 %	-2.2565	0.56	1.48
GO:1902125	(+)-pinoresinol catabolic process	0.087 %	-1.9567	0.56	1.48
GO:1902131	(+)-lariciresinol catabolic process	0.087 %	-1.9567	0.53	1.48
GO:1902132	(+)-lariciresinol biosynthetic process	0.510 %	-1.9567	0.48	1.75
GO:1902129	(-)-lariciresinol biosynthetic process	0.542 %	-2.1320	0.51	1.76
GO:1902130	(+)-lariciresinol metabolic process	1.007 %	-1.9567	0.47	1.88
GO:0044550	secondary metabolite biosynthetic process	1.183 %	-3.2292	0.66	0.80
GO:1901598	(-)-pinoresinol metabolic process	0.009 %	-1.6580	0.64	1.02
GO:0009699	phenylpropanoid biosynthetic process	0.337 %	-4.3041	0.63	0.82
GO:1902138	(-)-secoisolariciresinol biosynthetic process	0.083 %	-1.9567	0.54	1.47
GO:1902133	(+)-secoisolariciresinol metabolic process	0.029 %	-2.2565	0.58	1.11
GO:1902136	(-)-secoisolariciresinol metabolic process	0.124 %	-1.9567	0.54	1.52
GO:1902135	(+)-secoisolariciresinol biosynthetic process	0.018 %	-2.2565	0.58	1.20
GO:0009717	isoflavonoid biosynthetic process	0.527 %	-1.6954	0.61	1.10
GO:0009806	lignan metabolic process	0.022 %	-1.5347	0.70	0.67
GO:0009808	lignin metabolic process	0.285 %	-3.1203	0.65	0.91
GO:0009807	lignan biosynthetic process	0.022 %	-1.5347	0.64	1.09
GO:0009809	lignin biosynthetic process	0.181 %	-2.6396	0.64	0.88
GO:0009801	cinnamic acid ester metabolic process	0.009 %	-1.6580	0.72	0.64
GO:0009407	toxin catabolic process	0.199 %	-1.2400	0.67	0.67
GO:0046287	isoflavonoid metabolic process	1.301 %	-1.6954	0.61	1.05
GO:0046273	lignan catabolic process	0.468 %	-1.9567	0.54	1.73
GO:0046274	lignin catabolic process	0.078 %	-3.0843	0.64	0.73
GO:0046271	phenylpropanoid catabolic process	0.078 %	-4.3554	0.65	0.62
GO:0010037	response to carbon dioxide	0.030 %	-1.2964	0.90	0.30
GO:0010378	temperature compensation of the circadian clock	0.004 %	-1.5919	0.88	0.30
GO:0006869	lipid transport	0.596 %	-1.6709	0.87	0.31
GO:0010876	lipid localization	0.708 %	-1.4574	0.97	0.59
GO:0009820	alkaloid metabolic process	0.009 %	-2.5159	0.94	0.32
GO:0071329	cellular response to sucrose stimulus	0.039 %	-1.0138	0.85	0.32
GO:0071324	cellular response to disaccharide stimulus	0.043 %	-1.0138	0.85	0.78
GO:0044710	single-organism metabolic process	18.390 %	-1.3137	0.84	0.32
GO:0019932	second-messenger-mediated signaling	0.319 %	-1.6130	0.77	0.34
GO:0045493	xylan catabolic process	0.017 %	-2.1468	0.85	0.34
GO:0000272	polysaccharide catabolic process	0.777 %	-1.4043	0.83	0.65
GO:0031222	arabinan catabolic process	0.004 %	-1.2816	0.78	0.52
GO:0045491	xylan metabolic process	0.173 %	-1.4524	0.88	0.72
GO:0010410	hemicellulose metabolic process	0.423 %	-1.0989	0.87	0.89
GO:0046838	phosphorylated carbohydrate dephosphorylation	0.030 %	-1.0214	0.90	0.35
GO:0080166	stomium development	0.004 %	-1.9567	0.78	0.35
GO:0048467	gynoecium development	0.315 %	-1.1659	0.79	0.63
GO:0048438	floral whorl development	0.669 %	-1.0068	0.78	0.71
GO:0048653	anther development	0.224 %	-1.0453	0.79	0.84
GO:0022412	cellular process involved in reproduction in multicellular organism	0.177 %	-1.2931	0.77	0.62
GO:0007389	pattern specification process	0.742 %	-1.4127	0.82	0.35
GO:0050982	detection of mechanical stimulus	0.017 %	-1.6237	0.89	0.36
GO:0032107	regulation of response to nutrient levels	0.022 %	-1.4612	0.88	0.36
GO:0032104	regulation of response to extracellular stimulus	0.022 %	-1.4612	0.88	0.68
GO:0009628	response to abiotic stimulus	7.946 %	-1.9422	0.89	0.36
GO:0010039	response to iron ion	0.237 %	-2.0363	0.88	0.37
GO:0071248	cellular response to metal ion	0.216 %	-1.1705	0.83	0.77
GO:0071241	cellular response to inorganic substance	0.246 %	-1.0034	0.83	0.53
GO:0071280	cellular response to copper ion	0.009 %	-1.7364	0.86	0.50
GO:0010043	response to zinc ion	0.186 %	-1.7455	0.88	0.63
GO:0046688	response to copper ion	0.069 %	-1.6255	0.89	0.58
GO:0010865	stipule development	0.004 %	-1.8325	0.86	0.39
GO:0007267	cell-cell signaling	0.371 %	-1.0879	0.82	0.39
GO:0071456	cellular response to hypoxia	0.117 %	-1.6982	0.81	0.39
GO:0036294	cellular response to decreased oxygen levels	0.129 %	-1.6192	0.81	0.99
GO:0071453	cellular response to oxygen levels	0.129 %	-1.6192	0.81	0.87
GO:0001666	response to hypoxia	0.250 %	-1.0507	0.86	0.92
GO:0051782	negative regulation of cell division	0.022 %	-1.1568	0.80	0.39
GO:0006629	lipid metabolic process	4.683 %	-1.0588	0.75	0.39
GO:0030418	nicotianamine biosynthetic process	0.022 %	-2.1320	0.75	0.40
GO:0072351	tricarboxylic acid biosynthetic process	0.022 %	-2.1320	0.75	0.70
GO:0030417	nicotianamine metabolic process	0.022 %	-2.1320	0.77	0.71
GO:0043620	regulation of DNA-templated transcription in response to stress	0.013 %	-1.0702	0.77	0.40

GO:0010252	auxin homeostasis	0.142 %	-1.0789	0.94	0.40
GO:0055071	manganese ion homeostasis	0.065 %	-1.0370	0.94	0.70
GO:0006879	cellular iron ion homeostasis	0.108 %	-1.0616	0.81	0.59
GO:1901616	organic hydroxy compound catabolic process	0.121 %	-1.8773	0.78	0.40
GO:0019751	polyol metabolic process	0.246 %	-1.4777	0.68	0.68
GO:0034312	diol biosynthetic process	0.004 %	-1.6954	0.71	0.51
GO:0034311	diol metabolic process	0.004 %	-1.6954	0.74	0.60
GO:0034310	primary alcohol catabolic process	0.128 %	-1.9567	0.64	0.91
GO:0034309	primary alcohol biosynthetic process	0.009 %	-1.9567	0.71	0.94
GO:0019336	phenol-containing compound catabolic process	0.030 %	-1.9567	0.73	0.58
GO:0032957	inositol trisphosphate metabolic process	0.035 %	-1.0702	0.69	0.75
GO:0046855	inositol phosphate dephosphorylation	0.026 %	-1.0214	0.65	0.95
GO:0006066	alcohol metabolic process	0.505 %	-1.0617	0.68	0.76
GO:0071545	inositol phosphate catabolic process	0.026 %	-1.0214	0.66	0.95
GO:0046173	polyol biosynthetic process	0.082 %	-1.0370	0.67	0.80
GO:0046164	alcohol catabolic process	0.082 %	-2.0214	0.66	0.97
GO:0009739	response to gibberellin	0.626 %	-2.1375	0.88	0.41
GO:0009751	response to salicylic acid	0.876 %	-1.1758	0.87	0.58
GO:0048569	post-embryonic animal organ development	0.572 %	-1.6580	0.77	0.41
GO:0048504	regulation of timing of animal organ formation	0.004 %	-1.4612	0.79	1.32
GO:0010160	formation of animal organ boundary	0.055 %	-1.0616	0.78	1.59
GO:2000027	regulation of organ morphogenesis	0.004 %	-1.0532	0.80	1.32
GO:0003156	regulation of animal organ formation	0.004 %	-1.1568	0.79	1.32
GO:0072350	tricarboxylic acid metabolic process	0.259 %	-1.0702	0.75	0.41
GO:0080027	response to herbivore	0.043 %	-1.0370	0.91	0.42
GO:0071457	cellular response to ozone	0.013 %	-1.6954	0.85	0.42
GO:0034599	cellular response to oxidative stress	0.453 %	-1.0562	0.83	0.54
GO:0034614	cellular response to reactive oxygen species	0.207 %	-1.2399	0.83	0.71
GO:0031221	arabinan metabolic process	0.009 %	-1.1064	0.81	0.43
GO:0009555	pollen development	1.230 %	-1.0807	0.82	0.44
GO:0048016	inositol phosphate-mediated signaling	0.009 %	-1.3278	0.81	0.45
GO:0006950	response to stress	14.156 %	-1.3605	0.88	0.45
GO:0009642	response to light intensity	0.587 %	-2.0096	0.85	0.45
GO:0010224	response to UV-B	0.298 %	-1.0930	0.85	0.78
GO:0048578	positive regulation of long-day photoperiodism, flowering	0.030 %	-1.2672	0.69	0.53
GO:0009644	response to high light intensity	0.324 %	-1.2440	0.85	0.64
GO:0009637	response to blue light	0.341 %	-1.3500	0.86	0.64
GO:0009647	skotomorphogenesis	0.026 %	-1.1568	0.75	0.73
GO:0009785	blue light signaling pathway	0.078 %	-1.9413	0.74	0.57
GO:0034644	cellular response to UV	0.073 %	-1.0450	0.82	0.83
GO:0071482	cellular response to light stimulus	0.561 %	-1.4910	0.80	0.85
GO:0071483	cellular response to blue light	0.104 %	-1.7337	0.82	0.81
GO:0071484	cellular response to light intensity	0.043 %	-1.1258	0.83	0.76
GO:0071478	cellular response to radiation	0.583 %	-1.4910	0.80	0.91
GO:0071493	cellular response to UV-B	0.030 %	-1.2270	0.83	0.68
GO:0010286	heat acclimation	0.207 %	-1.0901	0.88	0.45
GO:1901502	ether catabolic process	0.474 %	-1.9567	0.68	0.46
GO:1901503	ether biosynthetic process	1.458 %	-1.9567	0.68	1.37
GO:0042128	nitrate assimilation	0.181 %	-1.0214	0.75	0.46
GO:0042126	nitrate metabolic process	0.181 %	-1.0214	0.75	0.98
GO:0044703	multi-organism reproductive process	1.610 %	-1.3971	0.92	0.47
GO:0009566	fertilization	0.199 %	-1.3906	0.85	0.72
GO:0007338	single fertilization	0.060 %	-1.1462	0.86	0.75
GO:0019953	sexual reproduction	0.596 %	-1.4043	0.93	0.71
GO:2000122	negative regulation of stomatal complex development	0.022 %	-1.8901	0.79	0.49
GO:0010052	guard cell differentiation	0.082 %	-1.2534	0.75	0.79
GO:0010654	apical cell fate commitment	0.004 %	-1.8325	0.79	0.49
GO:0009653	anatomical structure morphogenesis	3.500 %	-1.0587	0.89	0.49
GO:0030154	cell differentiation	3.332 %	-1.0447	0.73	0.53
GO:0048479	style development	0.004 %	-1.7364	0.84	0.49
GO:0048480	stigma development	0.004 %	-1.3118	0.84	0.59
GO:0006720	isoprenoid metabolic process	0.850 %	-1.1381	0.71	0.49
GO:0008610	lipid biosynthetic process	2.464 %	-1.0887	0.69	0.69
GO:0015749	monosaccharide transport	0.229 %	-1.3618	0.88	0.50

Biological Process (249) Tag Clouds

Scatterplot & Table Interactive Graph TreeMap

GROUP 32

Tip: your resulting list of GO terms seems to be quite long. If you want to reduce it further, press the Back button in your browser and choose a different setting for the "allowed similarity" parameter.

Hide/show dispensable GO terms Export results to text table (CSV) Make R script for plotting

term ID	description	frequency	pin?	$\log_{10}$ p-value	uniqueness	dispensability
GO:0009653	anatomical structure morphogenesis	3.500 %		-3.2123	0.87	0.00
GO:0044707	*single-multicellular organism process*	10.699 %		-1.3706	0.81	0.70
GO:0099402	*plant organ development*	3.763 %		-1.2774	0.78	0.67
GO:0044767	*single-organism developmental process*	12.123 %		-2.2229	0.78	0.66
GO:0022622	*root system development*	1.895 %		-1.8554	0.79	0.52
GO:0007275	*multicellular organism development*	10.423 %		-1.2071	0.77	0.86
GO:0030154	*cell differentiation*	3.332 %		-1.2241	0.70	0.53
GO:0048856	*anatomical structure development*	11.722 %		-1.7977	0.86	0.85
GO:0009793	*embryo development ending in seed dormancy*	1.584 %		-1.1492	0.78	0.60
GO:0045596	*negative regulation of cell differentiation*	0.052 %		-1.1225	0.73	0.56
GO:0090696	*post-embryonic plant organ development*	0.686 %		-1.8214	0.80	0.54
GO:0009682	induced systemic resistance	0.125 %		-3.1983	0.97	0.00
GO:0009866	*induced systemic resistance, ethylene mediated signaling pathway*	0.013 %		-1.9863	0.83	0.54
GO:0032501	multicellular organismal process	11.360 %		-1.3026	0.99	0.00
GO:0032502	developmental process	12.689 %		-1.9932	0.99	0.00
GO:0040007	growth	2.529 %		-3.1631	0.99	0.00

GO:0044699	single-organism process	41.709 %	-4.4207	1.00	0.00
GO:0051301	cell division	1.528 %	-11.1410	0.79	0.00
GO:0051338	regulation of transferase activity	0.522 %	-2.7693	0.89	0.00
GO:0050790	regulation of catalytic activity	2.257 %	-1.7931	0.88	0.80
GO:0051704	multi-organism process	3.362 %	-1.5958	0.99	0.00
GO:0065007	biological regulation	28.554 %	-1.0336	0.99	0.00
GO:0071840	cellular component organization or biogenesis	14.454 %	-6.1136	0.99	0.00
GO:0010425	DNA methylation on cytosine within a CNG sequence	0.004 %	-4.8848	0.80	0.03
GO:0006305	DNA alkylation	0.250 %	-1.8405	0.77	0.96
GO:0006306	DNA methylation	0.250 %	-1.8405	0.74	0.91
GO:0032776	DNA methylation on cytosine	0.078 %	-3.1200	0.77	0.69
GO:0034968	histone lysine methylation	0.298 %	-2.2293	0.70	0.86
GO:0045814	negative regulation of gene expression, epigenetic	0.311 %	-1.8049	0.82	0.85
GO:0018205	peptidyl-lysine modification	0.725 %	-1.5719	0.83	0.64
GO:0006333	chromatin assembly or disassembly	0.207 %	-1.7710	0.80	0.76
GO:0044728	DNA methylation or demethylation	0.298 %	-1.8200	0.76	0.98
GO:0006342	chromatin silencing	0.298 %	-1.8669	0.59	0.79
GO:0061647	histone H3-K9 modification	0.069 %	-2.5861	0.75	0.64
GO:0051567	histone H3-K9 methylation	0.065 %	-2.5861	0.73	0.53
GO:0043414	macromolecule methylation	0.928 %	-1.2562	0.82	0.65
GO:0016572	histone phosphorylation	0.026 %	-2.1617	0.75	0.60
GO:0016571	histone methylation	0.354 %	-1.9169	0.70	0.97
GO:0016570	histone modification	0.893 %	-2.3088	0.70	0.89
GO:0016569	covalent chromatin modification	1.273 %	-2.2043	0.71	0.69
GO:0018022	peptidyl-lysine methylation	0.350 %	-2.2125	0.81	0.93
GO:0006479	protein methylation	0.475 %	-1.7951	0.81	0.97
GO:0032259	methylation	1.834 %	-1.2333	0.95	0.04
GO:0009294	DNA mediated transformation	0.099 %	-3.0658	0.94	0.04
GO:0044764	multi-organism cellular process	0.414 %	-1.3417	0.93	0.53
GO:0009292	genetic transfer	0.099 %	-3.0658	0.94	0.79
GO:0051276	chromosome organization	1.118 %	-3.8411	0.80	0.05
GO:0000280	nuclear division	1.079 %	-1.1669	0.79	0.67
GO:0006323	DNA packaging	0.220 %	-3.0273	0.80	0.78
GO:0098813	nuclear chromosome segregation	0.436 %	-2.0544	0.66	0.92
GO:0006265	DNA topological change	0.065 %	-1.5823	0.70	0.71
GO:1902589	single-organism organelle organization	1.675 %	-1.2720	0.68	0.60
GO:0006334	nucleosome assembly	0.134 %	-2.0341	0.75	0.82
GO:0000819	sister chromatid segregation	0.285 %	-2.4755	0.57	0.80
GO:0051783	regulation of nuclear division	0.181 %	-1.0497	0.75	0.80
GO:0030261	chromosome condensation	0.052 %	-1.8621	0.82	0.86
GO:0065004	protein-DNA complex assembly	0.393 %	-1.9401	0.79	0.91
GO:0048285	organelle fission	1.239 %	-1.0057	0.79	0.61
GO:0034728	nucleosome organization	0.173 %	-2.0341	0.80	0.84
GO:0031497	chromatin assembly	0.164 %	-1.8669	0.76	0.95
GO:0000070	mitotic sister chromatid segregation	0.207 %	-1.2962	0.57	0.90
GO:0007076	mitotic chromosome condensation	0.022 %	-1.8621	0.62	0.65
GO:0071103	DNA conformation change	0.539 %	-3.7260	0.79	0.53
GO:0040020	regulation of meiotic nuclear division	0.047 %	-1.2115	0.58	0.71
GO:0071669	plant-type cell wall organization or biogenesis	1.152 %	-5.0734	0.87	0.05
GO:0042547	cell wall modification involved in multidimensional cell growth	0.017 %	-1.2778	0.74	0.60
GO:0042546	cell wall biogenesis	0.928 %	-3.8434	0.78	0.73
GO:0010215	cellulose microfibril organization	0.073 %	-1.3155	0.71	0.77
GO:0070726	cell wall assembly	0.082 %	-1.1965	0.80	0.71
GO:2000652	regulation of secondary cell wall biogenesis	0.095 %	-1.0929	0.74	0.78
GO:1903338	regulation of cell wall organization or biogenesis	0.138 %	-1.0654	0.82	0.61
GO:0009664	plant-type cell wall organization	0.652 %	-1.8364	0.78	0.87
GO:0045492	xylan biosynthetic process	0.138 %	-1.0346	0.64	0.74
GO:0071555	cell wall organization	2.430 %	-1.7819	0.76	0.82
GO:0009832	plant-type cell wall biogenesis	0.626 %	-4.1089	0.77	0.84
GO:0009834	plant-type secondary cell wall biogenesis	0.263 %	-4.5820	0.79	0.64
GO:0071668	plant-type cell wall assembly	0.082 %	-1.1965	0.79	0.99
GO:0009828	plant-type cell wall loosening	0.022 %	-1.7660	0.82	0.52
GO:0071554	cell wall organization or biogenesis	3.168 %	-4.1799	0.93	0.06
GO:0030243	cellulose metabolic process	0.337 %	-1.2446	0.86	0.09
GO:0000272	polysaccharide catabolic process	0.777 %	-1.1011	0.88	0.74
GO:0051275	beta-glucan catabolic process	0.112 %	-1.1965	0.86	0.89
GO:0051273	beta-glucan metabolic process	0.393 %	-1.0636	0.86	0.82
GO:0030245	cellulose catabolic process	0.112 %	-1.1965	0.86	0.88
GO:0005976	polysaccharide metabolic process	1.899 %	-1.0782	0.89	0.60
GO:0072348	sulfur compound transport	0.147 %	-1.2778	0.91	0.10

GO:0019748	secondary metabolic process	1.938 %	-3.5219	0.85	0.13
GO:0009821	alkaloid biosynthetic process	0.795 %	-3.0955	0.86	0.14
GO:0080144	amino acid homeostasis	0.060 %	-1.2039	0.93	0.14
GO:1901361	organic cyclic compound catabolic process	0.937 %	-1.6114	0.89	0.15
GO:0010212	response to ionizing radiation	0.129 %	-2.6265	0.97	0.15
GO:0043412	macromolecule modification	15.900 %	-1.5955	0.90	0.16
GO:0051174	regulation of phosphorus metabolic process	0.561 %	-2.3587	0.81	0.17
GO:0009723	response to ethylene	1.269 %	-1.0911	0.97	0.18
GO:0044763	single-organism cellular process	23.034 %	-5.3156	0.82	0.19
GO:0065009	regulation of molecular function	2.443 %	-1.3129	0.91	0.20
GO:0008356	asymmetric cell division	0.091 %	-5.2546	0.79	0.20
GO:0051302	*regulation of cell division*	*0.199 %*	*-1.5329*	*0.74*	*0.62*
GO:0010069	*zygote asymmetric cytokinesis in embryo sac*	*0.013 %*	*-3.6563*	*0.64*	*0.79*
GO:0010070	*zygote asymmetric cell division*	*0.017 %*	*-3.6563*	*0.81*	*0.53*
GO:0009786	*regulation of asymmetric cell division*	*0.017 %*	*-1.7382*	*0.78*	*0.81*
GO:0034434	sterol esterification	0.009 %	-1.7660	0.81	0.21
GO:0016127	*sterol catabolic process*	*0.009 %*	*-1.7660*	*0.83*	*0.58*
GO:0006706	*steroid catabolic process*	*0.009 %*	*-1.7660*	*0.85*	*0.58*
GO:0034433	*steroid esterification*	*0.009 %*	*-1.7660*	*0.81*	*0.58*
GO:0007018	microtubule-based movement	0.281 %	-8.5529	0.82	0.22
GO:0080175	*phragmoplast microtubule organization*	*0.017 %*	*-1.6213*	*0.76*	*0.65*
GO:0006928	movement of cell or subcellular component	0.363 %	-8.3121	0.82	0.23
GO:0007059	chromosome segregation	0.531 %	-1.9342	0.81	0.24
GO:0007017	microtubule-based process	0.859 %	-5.3312	0.80	0.25
GO:0080170	hydrogen peroxide transmembrane transport	0.022 %	-1.2271	0.99	0.25
GO:0016049	cell growth	1.519 %	-3.3973	0.78	0.27
GO:0001558	*regulation of cell growth*	*0.293 %*	*-1.1649*	*0.68*	*0.72*
GO:2000603	*regulation of secondary growth*	*0.009 %*	*-1.1749*	*0.86*	*0.53*
GO:2000605	*positive regulation of secondary growth*	*0.004 %*	*-1.1965*	*0.86*	*0.87*
GO:0040008	*regulation of growth*	*1.148 %*	*-1.2076*	*0.88*	*0.83*
GO:0009605	response to external stimulus	6.366 %	-1.0300	0.97	0.28
GO:0007049	cell cycle	2.525 %	-10.1603	0.78	0.29
GO:0022402	cell cycle process	1.713 %	-8.6847	0.64	0.29
GO:0000281	*mitotic cytokinesis*	*0.311 %*	*-6.0377*	*0.64*	*0.86*
GO:0000278	*mitotic cell cycle*	*1.152 %*	*-8.4754*	*0.65*	*0.84*
GO:0001932	*regulation of protein phosphorylation*	*0.432 %*	*-2.8191*	*0.74*	*0.98*
GO:0045859	*regulation of protein kinase activity*	*0.423 %*	*-2.9537*	*0.73*	*0.95*
GO:0051726	*regulation of cell cycle*	*0.811 %*	*-5.0871*	*0.62*	*0.81*
GO:0006279	*premeiotic DNA replication*	*0.004 %*	*-2.9386*	*0.66*	*0.51*
GO:0019220	*regulation of phosphate metabolic process*	*0.561 %*	*-2.3587*	*0.78*	*0.98*
GO:0044772	*mitotic cell cycle phase transition*	*0.190 %*	*-1.1104*	*0.66*	*0.97*
GO:0044770	*cell cycle phase transition*	*0.207 %*	*-1.1104*	*0.68*	*0.70*
GO:0045930	*negative regulation of mitotic cell cycle*	*0.108 %*	*-1.4280*	*0.63*	*0.84*
GO:0071900	*regulation of protein serine/threonine kinase activity*	*0.242 %*	*-3.2987*	*0.74*	*0.86*
GO:0044839	*cell cycle G2/M phase transition*	*0.091 %*	*-1.6014*	*0.68*	*0.87*
GO:0044774	*mitotic DNA integrity checkpoint*	*0.043 %*	*-2.2406*	*0.64*	*0.61*
GO:0007346	*regulation of mitotic cell cycle*	*0.306 %*	*-2.2378*	*0.62*	*0.86*
GO:0051321	*meiotic cell cycle*	*0.716 %*	*-1.2177*	*0.66*	*0.80*
GO:0010564	*regulation of cell cycle process*	*0.384 %*	*-1.8405*	*0.61*	*0.79*
GO:0043549	*regulation of kinase activity*	*0.427 %*	*-2.8912*	*0.78*	*0.98*
GO:0000910	*cytokinesis*	*0.384 %*	*-5.6179*	*0.65*	*0.74*
GO:0000911	*cytokinesis by cell plate formation*	*0.194 %*	*-3.1220*	*0.65*	*0.95*
GO:0000912	*assembly of actomyosin apparatus involved in cytokinesis*	*0.009 %*	*-3.7552*	*0.65*	*0.66*
GO:0000914	*phragmoplast assembly*	*0.009 %*	*-3.7552*	*0.64*	*0.53*
GO:1902749	*regulation of cell cycle G2/M phase transition*	*0.082 %*	*-1.6014*	*0.64*	*0.98*
GO:1901987	*regulation of cell cycle phase transition*	*0.164 %*	*-1.2602*	*0.62*	*0.95*
GO:1902407	*assembly of actomyosin apparatus involved in mitotic cytokinesis*	*0.009 %*	*-3.7552*	*0.64*	*1.00*
GO:0032506	*cytokinetic process*	*0.203 %*	*-3.0349*	*0.66*	*0.93*
GO:1902410	*mitotic cytokinetic process*	*0.203 %*	*-3.0349*	*0.65*	*0.99*
GO:1901990	*regulation of mitotic cell cycle phase transition*	*0.147 %*	*-1.2602*	*0.61*	*0.94*
GO:0061640	*cytoskeleton-dependent cytokinesis*	*0.332 %*	*-5.6868*	*0.64*	*0.97*
GO:0007093	*mitotic cell cycle checkpoint*	*0.099 %*	*-1.5298*	*0.62*	*0.94*
GO:0051445	*regulation of meiotic cell cycle*	*0.060 %*	*-1.0873*	*0.66*	*0.78*
GO:0042325	*regulation of phosphorylation*	*0.462 %*	*-2.7571*	*0.79*	*0.95*
GO:0000075	*cell cycle checkpoint*	*0.117 %*	*-1.2962*	*0.64*	*0.90*
GO:0000079	*regulation of cyclin-dependent protein serine/threonine kinase activity*	*0.138 %*	*-3.8259*	*0.56*	*0.67*
GO:0009833	*plant-type primary cell wall biogenesis*	*0.091 %*	*-1.0346*	*0.56*	*0.89*
GO:0007067	*mitotic nuclear division*	*0.699 %*	*-1.8777*	*0.55*	*0.93*
GO:0031570	*DNA integrity checkpoint*	*0.060 %*	*-1.5641*	*0.65*	*0.89*
GO:0010389	*regulation of G2/M transition of mitotic cell cycle*	*0.078 %*	*-1.6014*	*0.63*	*0.77*
GO:0000086	*G2/M transition of mitotic cell cycle*	*0.086 %*	*-1.6014*	*0.68*	*0.99*

GO:1904029	regulation of cyclin-dependent protein kinase activity	0.138 %	-3.8259	0.55	0.83
GO:1903047	mitotic cell cycle process	1.083 %	-8.8521	0.63	0.83
GO:1903046	meiotic cell cycle process	0.613 %	-1.6326	0.65	0.78
GO:0045786	negative regulation of cell cycle	0.211 %	-1.0817	0.63	0.81
GO:0009698	phenylpropanoid metabolic process	0.505 %	-3.0087	0.74	0.30
GO:0019760	glucosinolate metabolic process	0.514 %	-1.4992	0.73	0.80
GO:0019757	glycosinolate metabolic process	0.514 %	-1.4992	0.73	0.99
GO:0044550	secondary metabolite biosynthetic process	1.183 %	-1.9196	0.80	0.80
GO:0009699	phenylpropanoid biosynthetic process	0.337 %	-2.5328	0.73	0.91
GO:0010023	proanthocyanidin biosynthetic process	0.035 %	-1.3254	0.76	0.79
GO:2000029	regulation of proanthocyanidin biosynthetic process	0.009 %	-2.6378	0.74	0.53
GO:0018958	phenol-containing compound metabolic process	0.190 %	-1.1225	0.87	0.55
GO:0046189	phenol-containing compound biosynthetic process	0.104 %	-1.1225	0.87	0.84
GO:0009808	lignin metabolic process	0.285 %	-2.9908	0.74	0.69
GO:0009809	lignin biosynthetic process	0.181 %	-2.3088	0.74	0.86
GO:0046274	lignin catabolic process	0.078 %	-2.2378	0.75	0.87
GO:0046271	phenylpropanoid catabolic process	0.078 %	-2.1879	0.75	0.81
GO:0090057	root radial pattern formation	0.013 %	-2.0950	0.84	0.31
GO:0009820	alkaloid metabolic process	0.009 %	-2.9215	0.94	0.32
GO:0019222	regulation of metabolic process	14.847 %	-1.1352	0.84	0.32
GO:0006839	mitochondrial transport	0.522 %	-1.1545	0.98	0.32
GO:0031399	regulation of protein modification process	0.699 %	-2.1896	0.75	0.33
GO:0032268	regulation of cellular protein metabolic process	1.921 %	-1.1491	0.75	0.85
GO:0016458	gene silencing	0.773 %	-1.0315	0.70	0.34
GO:0008213	protein alkylation	0.475 %	-1.7951	0.85	0.35
GO:0070417	cellular response to cold	0.147 %	-1.9055	0.92	0.35
GO:0040029	regulation of gene expression, epigenetic	0.691 %	-1.0136	0.83	0.37
GO:0051246	regulation of protein metabolic process	2.123 %	-1.0522	0.80	0.39
GO:0080090	regulation of primary metabolic process	13.681 %	-1.0641	0.80	0.56
GO:0060255	regulation of macromolecule metabolic process	14.091 %	-1.0528	0.78	0.82
GO:0080167	response to karrikin	0.531 %	-1.1096	0.97	0.40
GO:0018193	peptidyl-amino acid modification	1.813 %	-1.2199	0.83	0.40
GO:0016310	phosphorylation	6.966 %	-1.5356	0.85	0.75
GO:0036211	protein modification process	12.719 %	-1.9097	0.84	0.75
GO:0006464	cellular protein modification process	12.719 %	-1.9097	0.79	0.59
GO:0006468	protein phosphorylation	5.334 %	-2.0023	0.78	0.72
GO:0008272	sulfate transport	0.078 %	-1.3461	0.91	0.41
GO:0006325	chromatin organization	1.675 %	-3.0533	0.79	0.41
GO:0071824	protein-DNA complex subunit organization	0.432 %	-1.9401	0.81	0.60
GO:0006636	unsaturated fatty acid biosynthetic process	0.108 %	-1.3057	0.78	0.41
GO:0033559	unsaturated fatty acid metabolic process	0.112 %	-1.3057	0.79	0.65
GO:0051128	regulation of cellular component organization	1.467 %	-1.2089	0.74	0.42
GO:0045229	external encapsulating structure organization	2.577 %	-1.4796	0.80	0.45
GO:0016043	cellular component organization	13.263 %	-3.8731	0.77	0.66
GO:0022607	cellular component assembly	3.949 %	-1.3336	0.77	0.56
GO:0044085	cellular component biogenesis	5.783 %	-3.1283	0.83	0.68
GO:0043933	macromolecular complex subunit organization	4.933 %	-1.3929	0.79	0.53
GO:0006996	organelle organization	6.439 %	-1.4072	0.78	0.69
GO:0048646	anatomical structure formation involved in morphogenesis	0.755 %	-2.9926	0.86	0.46
GO:0048532	anatomical structure arrangement	0.289 %	-1.1792	0.81	0.60
GO:0048528	post-embryonic root development	0.501 %	-1.4293	0.79	0.93
GO:0048527	lateral root development	0.457 %	-1.5991	0.79	0.92
GO:0090558	plant epidermis development	1.001 %	-1.8564	0.87	0.61
GO:0010102	lateral root morphogenesis	0.229 %	-2.1502	0.78	0.86
GO:0010101	post-embryonic root morphogenesis	0.233 %	-2.1502	0.78	0.90
GO:0010026	trichome differentiation	0.341 %	-1.0653	0.73	0.71
GO:0010492	maintenance of shoot apical meristem identity	0.056 %	-1.2689	0.84	0.62
GO:0010015	root morphogenesis	0.967 %	-2.5496	0.77	0.68
GO:1905392	plant organ morphogenesis	1.485 %	-2.3029	0.77	0.73
GO:0048364	root development	1.886 %	-1.8682	0.77	0.90
GO:0010311	lateral root formation	0.168 %	-1.5364	0.78	0.97
GO:0090697	post-embryonic plant organ morphogenesis	0.419 %	-2.4764	0.77	0.82
GO:0090698	post-embryonic plant morphogenesis	0.716 %	-2.6652	0.86	0.66
GO:0009933	meristem structural organization	0.246 %	-1.2703	0.80	0.87
GO:0009934	regulation of meristem structural organization	0.065 %	-2.0822	0.77	0.53
GO:0090626	plant epidermis morphogenesis	0.367 %	-1.0381	0.85	0.81
GO:0006260	DNA replication	0.665 %	-4.5614	0.78	0.47
GO:0006302	double-strand break repair	0.483 %	-1.0346	0.78	0.77
GO:0006304	DNA modification	0.311 %	-1.8200	0.77	0.66
GO:0090068	positive regulation of cell cycle process	0.104 %	-1.3057	0.63	0.82
GO:0042023	DNA endoreduplication	0.160 %	-1.8615	0.58	0.86

GO:0006270	DNA replication initiation	0.086 %	-1.7957	0.79	0.79
GO:0006275	regulation of DNA replication	0.125 %	-1.1164	0.73	0.82
GO:0006261	DNA-dependent DNA replication	0.419 %	-2.8513	0.77	0.68
GO:0044786	cell cycle DNA replication	0.181 %	-3.1057	0.58	0.84
GO:0032877	positive regulation of DNA endoreduplication	0.022 %	-2.2406	0.57	0.75
GO:0032875	regulation of DNA endoreduplication	0.086 %	-1.2689	0.54	0.93
GO:0090329	regulation of DNA-dependent DNA replication	0.095 %	-1.1749	0.73	0.95
GO:2000105	positive regulation of DNA-dependent DNA replication	0.022 %	-2.2406	0.74	0.98
GO:0051054	positive regulation of DNA metabolic process	0.086 %	-1.5466	0.74	0.59
GO:0033260	nuclear DNA replication	0.022 %	-2.9386	0.63	0.72
GO:0000725	recombinational repair	0.350 %	-1.2517	0.78	0.88
GO:0045740	positive regulation of DNA replication	0.026 %	-2.2406	0.74	0.84
GO:0000724	double-strand break repair via homologous recombination	0.345 %	-1.2517	0.78	0.67
GO:0010332	response to gamma radiation	0.043 %	-2.7450	0.97	0.48
GO:0048829	root cap development	0.065 %	-1.0601	0.83	0.48
GO:0007349	cellularization	0.030 %	-3.1200	0.83	0.50
GO:0009558	embryo sac cellularization	0.022 %	-1.4546	0.83	0.91
GO:0010103	stomatal complex morphogenesis	0.125 %	-1.0346	0.79	0.78
GO:0010376	stomatal complex formation	0.013 %	-2.1617	0.82	0.57
GO:0010342	endosperm cellularization	0.009 %	-2.3373	0.84	0.56
GO:0006949	syncytium formation	0.043 %	-1.1749	0.76	0.61

Biological Process (191) Tag Clouds

Scatterplot & Table Interactive Graph TreeMap

GROUP 33

Tip: your resulting list of GO terms seems to be quite long. If you want to reduce it further, press the Back button in your browser and choose a different setting for the "allowed similarity" parameter.

Hide/show dispensable GO terms

Export results to text table (CSV)

Make R script for plotting

term ID	description	frequency	pin?	$\log_{10}$ p-value	uniqueness	dispensability
GO:0000003	reproduction	6.655 %		-2.7262	1.00	0.00
GO:0009624	response to nematode	0.306 %		-4.0798	0.88	0.00
GO:0043207	*response to external biotic stimulus*	*4.981 %*		*-1.8201*	*0.85*	*0.94*
GO:0051707	*response to other organism*	*4.968 %*		*-1.8201*	*0.85*	*0.65*
GO:0009991	*response to extracellular stimulus*	*0.777 %*		*-1.4539*	*0.87*	*0.66*
GO:0071496	*cellular response to external stimulus*	*0.712 %*		*-1.5757*	*0.88*	*0.65*
GO:0009820	alkaloid metabolic process	0.009 %		-5.2618	0.93	0.00
GO:0032501	multicellular organismal process	11.360 %		-4.1912	0.99	0.00
GO:0032502	developmental process	12.689 %		-4.4014	0.99	0.00
GO:0044699	single-organism process	41.709 %		-3.2742	0.99	0.00
GO:0048511	rhythmic process	0.600 %		-1.0037	0.99	0.00
GO:0050896	response to stimulus	25.943 %		-1.3068	0.99	0.00
GO:0051704	multi-organism process	3.362 %		-1.3370	0.99	0.00
GO:0061062	regulation of nematode larval development	0.009 %		-6.3917	0.73	0.00
GO:2000026	*regulation of multicellular organismal development*	*1.765 %*		*-2.6834*	*0.62*	*0.92*
GO:2000028	*regulation of photoperiodism, flowering*	*0.190 %*		*-1.1657*	*0.57*	*0.83*
GO:2000024	*regulation of leaf development*	*0.224 %*		*-1.1703*	*0.67*	*0.76*

GO:0048586	regulation of long-day photoperiodism, flowering	0.086 %	-1.3391	0.59	0.70
GO:0048574	long-day photoperiodism, flowering	0.121 %	-1.1891	0.69	0.95
GO:0048580	regulation of post-embryonic development	1.303 %	-3.1648	0.62	0.58
GO:0048571	long-day photoperiodism	0.138 %	-1.1025	0.90	0.85
GO:0051094	positive regulation of developmental process	0.436 %	-1.1933	0.70	0.73
GO:0002119	nematode larval development	0.009 %	-6.3917	0.81	1.00
GO:1900056	negative regulation of leaf senescence	0.043 %	-1.4070	0.69	0.83
GO:1900055	regulation of leaf senescence	0.104 %	-1.3188	0.69	0.71
GO:0065007	biological regulation	28.554 %	-1.4365	0.99	0.00
GO:0009058	biosynthetic process	24.432 %	-1.2580	0.94	0.05
GO:0007338	single fertilization	0.060 %	-3.3553	0.86	0.06
GO:0009566	fertilization	0.199 %	-2.1078	0.85	0.75
GO:0019953	sexual reproduction	0.596 %	-1.4132	0.90	0.65
GO:0015977	carbon fixation	0.104 %	-1.4686	0.88	0.07
GO:0008283	cell proliferation	0.470 %	-1.3170	0.91	0.08
GO:0051457	maintenance of protein location in nucleus	0.013 %	-2.2128	0.77	0.10
GO:0032507	maintenance of protein location in cell	0.078 %	-1.2140	0.74	0.97
GO:0072595	maintenance of protein localization in organelle	0.047 %	-1.7038	0.75	0.86
GO:0034504	protein localization to nucleus	0.268 %	-1.0725	0.95	0.59
GO:0051651	maintenance of location in cell	0.155 %	-1.0986	0.73	0.85
GO:0045185	maintenance of protein location	0.078 %	-1.1435	0.84	0.72
GO:0045912	negative regulation of carbohydrate metabolic process	0.035 %	-1.8869	0.82	0.12
GO:0019253	reductive pentose-phosphate cycle	0.086 %	-1.7566	0.81	0.88
GO:0010110	regulation of photosynthesis, dark reaction	0.013 %	-2.0373	0.76	0.79
GO:1905156	negative regulation of photosynthesis	0.073 %	-1.0313	0.79	0.80
GO:0080153	negative regulation of reductive pentose-phosphate cycle	0.013 %	-2.0373	0.75	0.73
GO:0080152	regulation of reductive pentose-phosphate cycle	0.013 %	-2.0373	0.76	1.00
GO:0071454	cellular response to anoxia	0.013 %	-2.0373	0.86	0.14
GO:0036294	cellular response to decreased oxygen levels	0.129 %	-1.0248	0.85	0.85
GO:0034059	response to anoxia	0.030 %	-1.5641	0.90	0.69
GO:0071453	cellular response to oxygen levels	0.129 %	-1.0248	0.85	0.88
GO:0010467	gene expression	18.304 %	-1.0488	0.91	0.16
GO:0065008	regulation of biological quality	5.991 %	-1.1458	0.84	0.17
GO:0051301	cell division	1.528 %	-1.3855	0.83	0.17
GO:0035445	borate transmembrane transport	0.017 %	-1.8389	0.96	0.21
GO:0098661	inorganic anion transmembrane transport	0.203 %	-1.7757	0.96	0.72
GO:0046713	borate transport	0.022 %	-1.8389	0.96	0.62
GO:0080110	sporopollenin biosynthetic process	0.030 %	-3.6674	0.66	0.23
GO:0010208	pollen wall assembly	0.164 %	-1.9264	0.68	0.98
GO:0010927	cellular component assembly involved in morphogenesis	0.168 %	-1.9264	0.71	0.67
GO:0010152	pollen maturation	0.069 %	-1.0653	0.79	0.69
GO:0085029	extracellular matrix assembly	0.164 %	-1.9264	0.82	0.97
GO:0009555	pollen development	1.230 %	-2.5786	0.74	0.65
GO:0010584	pollen exine formation	0.121 %	-2.3049	0.68	0.86
GO:0030198	extracellular matrix organization	0.233 %	-1.7760	0.82	0.95
GO:0010093	specification of floral organ identity	0.056 %	-1.2993	0.73	0.70
GO:0090701	specification of plant organ identity	0.060 %	-1.2993	0.77	0.73
GO:0048449	floral organ formation	0.104 %	-1.0346	0.72	0.88
GO:0007064	mitotic sister chromatid cohesion	0.095 %	-2.3374	0.80	0.23
GO:0000278	mitotic cell cycle	1.152 %	-1.3069	0.79	0.84
GO:0098813	nuclear chromosome segregation	0.436 %	-1.0479	0.79	0.92
GO:0044772	mitotic cell cycle phase transition	0.190 %	-1.2808	0.80	0.91
GO:0044770	cell cycle phase transition	0.207 %	-1.2808	0.81	0.70
GO:0000070	mitotic sister chromatid segregation	0.207 %	-1.4686	0.79	0.94
GO:0000082	G1/S transition of mitotic cell cycle	0.052 %	-1.7382	0.83	0.83
GO:0007062	sister chromatid cohesion	0.186 %	-1.5409	0.79	0.88
GO:0044843	cell cycle G1/S phase transition	0.069 %	-1.7382	0.82	0.56
GO:0000819	sister chromatid segregation	0.285 %	-1.2573	0.78	0.89
GO:1903047	mitotic cell cycle process	1.083 %	-1.3846	0.78	0.83
GO:0022402	cell cycle process	1.713 %	-1.6233	0.78	0.65
GO:0009605	response to external stimulus	6.366 %	-2.5147	0.90	0.23
GO:0032922	circadian regulation of gene expression	0.017 %	-1.7382	0.75	0.24
GO:0007623	circadian rhythm	0.539 %	-1.0132	0.98	0.73
GO:0009739	response to gibberellin	0.626 %	-1.8672	0.90	0.26
GO:0009741	response to brassinosteroid	0.410 %	-1.2570	0.90	0.63
GO:0009735	response to cytokinin	1.010 %	-1.3086	0.90	0.52
GO:0043062	extracellular structure organization	0.233 %	-1.7760	0.84	0.27
GO:0010036	response to boron-containing substance	0.022 %	-1.4323	0.93	0.27
GO:0002164	larval development	0.009 %	-6.3917	0.82	0.29
GO:0007049	cell cycle	2.525 %	-1.2040	0.82	0.29
GO:0050793	regulation of developmental process	2.577 %	-2.4836	0.68	0.31
GO:0048518	positive regulation of biological process	4.398 %	-1.3474	0.82	0.32

GO:0009821	alkaloid biosynthetic process	0.795 %	-3.4473	0.84	0.32
GO:0051239	regulation of multicellular organismal process	1.972 %	-2.3949	0.73	0.32
GO:0044707	single-multicellular organism process	10.699 %	-4.7191	0.76	0.86
GO:0044702	single organism reproductive process	5.939 %	-2.6020	0.81	0.86
GO:0003006	developmental process involved in reproduction	5.611 %	-1.7334	0.74	0.89
GO:0090567	reproductive shoot system development	2.110 %	-1.0928	0.68	0.84
GO:0044767	single-organism developmental process	12.123 %	-4.5934	0.72	0.82
GO:0048359	mucilage metabolic process involved in seed coat development	0.086 %	-1.2748	0.73	0.58
GO:0048856	anatomical structure development	11.722 %	-4.5158	0.77	0.85
GO:0048316	seed development	2.305 %	-1.0762	0.68	0.85
GO:0009791	post-embryonic development	5.848 %	-3.6742	0.71	0.78
GO:0048608	reproductive structure development	4.674 %	-2.2969	0.67	0.55
GO:0048869	cellular developmental process	4.148 %	-1.4296	0.69	0.59
GO:0007275	multicellular organism development	10.423 %	-4.8876	0.69	0.75
GO:0022414	reproductive process	6.629 %	-2.7608	0.89	0.91
GO:0048229	gametophyte development	1.653 %	-2.0427	0.75	0.57
GO:0061458	reproductive system development	4.674 %	-2.2969	0.71	0.80
GO:0048731	system development	7.225 %	-2.4370	0.70	0.82
GO:0080119	ER body organization	0.017 %	-1.7207	0.93	0.33
GO:0080001	mucilage extrusion from seed coat	0.022 %	-1.3322	0.78	0.34
GO:0009956	radial pattern formation	0.078 %	-1.0798	0.79	0.35
GO:0009607	response to biotic stimulus	5.158 %	-1.7735	0.91	0.35
GO:0042436	indole-containing compound catabolic process	0.039 %	-1.2689	0.88	0.35
GO:0045229	external encapsulating structure organization	2.577 %	-1.3301	0.91	0.36
GO:0043433	negative regulation of sequence-specific DNA binding transcription factor activity	0.022 %	-1.3755	0.80	0.39
GO:0051782	negative regulation of cell division	0.022 %	-1.5298	0.79	0.39
GO:0008356	asymmetric cell division	0.091 %	-1.1350	0.86	0.53
GO:0019685	photosynthesis, dark reaction	0.086 %	-1.7566	0.82	0.39
GO:1901672	positive regulation of systemic acquired resistance	0.017 %	-2.5131	0.78	0.39
GO:0010112	regulation of systemic acquired resistance	0.082 %	-1.3391	0.76	0.83
GO:0032103	positive regulation of response to external stimulus	0.108 %	-1.8869	0.75	0.74
GO:0002833	positive regulation of response to biotic stimulus	0.095 %	-2.2706	0.77	0.74
GO:0048878	chemical homeostasis	1.765 %	-1.3780	0.81	0.41
GO:0055080	cation homeostasis	1.083 %	-1.2266	0.81	0.92
GO:0055072	iron ion homeostasis	0.237 %	-1.0346	0.84	0.83
GO:0055065	metal ion homeostasis	0.842 %	-1.2775	0.82	0.80
GO:0030104	water homeostasis	0.052 %	-1.1435	0.86	0.61
GO:0050801	ion homeostasis	1.299 %	-1.0427	0.81	0.87
GO:0098771	inorganic ion homeostasis	1.139 %	-1.1339	0.81	0.95
GO:0043902	positive regulation of multi-organism process	0.017 %	-2.2706	0.87	0.41
GO:0006355	regulation of transcription, DNA-templated	11.234 %	-2.5703	0.64	0.42
GO:0032774	RNA biosynthetic process	11.813 %	-2.5374	0.78	0.82
GO:0080090	regulation of primary metabolic process	13.681 %	-2.0641	0.72	0.82
GO:0010468	regulation of gene expression	12.706 %	-2.4618	0.71	0.79
GO:0031323	regulation of cellular metabolic process	13.906 %	-2.3439	0.70	0.80
GO:0018130	heterocycle biosynthetic process	14.014 %	-1.7028	0.81	0.58
GO:0031326	regulation of cellular biosynthetic process	12.339 %	-1.8367	0.66	0.87
GO:0051252	regulation of RNA metabolic process	11.463 %	-2.4910	0.65	0.87
GO:2001141	regulation of RNA biosynthetic process	11.282 %	-2.5703	0.64	0.88
GO:0019219	regulation of nucleobase-containing compound metabolic process	11.700 %	-2.3820	0.66	0.84
GO:0016070	RNA metabolic process	17.562 %	-1.5479	0.81	0.66
GO:2000112	regulation of cellular macromolecule biosynthetic process	12.003 %	-2.1394	0.65	0.86
GO:0044249	cellular biosynthetic process	22.844 %	-1.1217	0.82	0.66
GO:0044271	cellular nitrogen compound biosynthetic process	17.018 %	-1.5889	0.80	0.61
GO:0006351	transcription, DNA-templated	11.709 %	-2.5374	0.77	0.87
GO:0060255	regulation of macromolecule metabolic process	14.091 %	-2.0454	0.72	0.83
GO:0019222	regulation of metabolic process	14.847 %	-2.4563	0.77	0.52
GO:0050794	regulation of cellular process	22.244 %	-1.5297	0.74	0.63
GO:1903506	regulation of nucleic acid-templated transcription	11.282 %	-2.5703	0.64	0.89
GO:0050789	regulation of biological process	24.333 %	-1.1311	0.80	0.54
GO:0097659	nucleic acid-templated transcription	11.761 %	-2.5374	0.77	0.88
GO:0009889	regulation of biosynthetic process	12.408 %	-2.3186	0.69	0.76
GO:0019438	aromatic compound biosynthetic process	14.247 %	-2.5099	0.81	0.55
GO:0010556	regulation of macromolecule biosynthetic process	12.076 %	-2.1302	0.68	0.86
GO:1901362	organic cyclic compound biosynthetic process	14.769 %	-1.2524	0.84	0.55
GO:0034654	nucleobase-containing compound biosynthetic process	13.056 %	-2.1014	0.78	0.80
GO:0051171	regulation of nitrogen compound metabolic process	12.430 %	-1.9547	0.70	0.78
GO:0045165	cell fate commitment	0.341 %	-2.2626	0.73	0.44
GO:0010103	stomatal complex morphogenesis	0.125 %	-1.2039	0.75	0.89
GO:0048646	anatomical structure formation involved in morphogenesis	0.755 %	-1.3877	0.80	0.54

GO:0010052	guard cell differentiation	0.082 %	-1.6282	0.71	0.51
GO:0019760	glucosinolate metabolic process	0.514 %	-1.8286	0.75	0.45
GO:0019757	glycosinolate metabolic process	0.514 %	-1.8286	0.75	0.99
GO:0042344	indole glucosinolate catabolic process	0.017 %	-1.2748	0.79	0.75
GO:0042343	indole glucosinolate metabolic process	0.125 %	-1.1266	0.76	0.88
GO:0009804	coumarin metabolic process	0.017 %	-1.5084	0.82	0.55
GO:0044273	sulfur compound catabolic process	0.173 %	-1.1478	0.89	0.72
GO:0016145	S-glycoside catabolic process	0.104 %	-1.2748	0.79	0.86
GO:0016143	S-glycoside metabolic process	0.514 %	-1.1266	0.78	0.80
GO:1901658	glycosyl compound catabolic process	0.211 %	-1.0185	0.84	0.87
GO:0048863	stem cell differentiation	0.013 %	-2.2128	0.79	0.45
GO:0009625	response to insect	0.108 %	-1.3057	0.89	0.45
GO:0051090	regulation of sequence-specific DNA binding transcription factor activity	0.052 %	-1.2351	0.79	0.45
GO:0031669	cellular response to nutrient levels	0.557 %	-1.8583	0.81	0.47
GO:0009267	cellular response to starvation	0.496 %	-1.1835	0.81	0.96
GO:0016036	cellular response to phosphate starvation	0.259 %	-1.4636	0.82	0.90
GO:0080029	cellular response to boron-containing substance levels	0.017 %	-1.4323	0.84	0.72
GO:0042594	response to starvation	0.531 %	-1.1496	0.85	0.96
GO:0031668	cellular response to extracellular stimulus	0.691 %	-1.6053	0.81	0.97
GO:0031667	response to nutrient levels	0.639 %	-1.6719	0.85	0.94
GO:0001709	cell fate determination	0.030 %	-2.0373	0.76	0.48
GO:0010158	abaxial cell fate specification	0.030 %	-1.6876	0.76	0.70
GO:0048867	stem cell fate determination	0.004 %	-2.2128	0.79	0.62
GO:0048865	stem cell fate commitment	0.004 %	-2.2128	0.79	0.89
GO:0001708	cell fate specification	0.134 %	-1.1225	0.74	0.77
GO:0045597	positive regulation of cell differentiation	0.030 %	-1.4070	0.68	0.48
GO:2000022	regulation of jasmonic acid mediated signaling pathway	0.108 %	-1.4981	0.71	0.48
GO:0031347	regulation of defense response	0.971 %	-1.4275	0.75	0.80
GO:0010104	regulation of ethylene-activated signaling pathway	0.095 %	-1.4070	0.71	0.65
GO:1902531	regulation of intracellular signal transduction	0.229 %	-1.0513	0.71	0.69
GO:0070297	regulation of phosphorelay signal transduction system	0.095 %	-1.4070	0.73	0.87
GO:0080134	regulation of response to stress	1.351 %	-1.2963	0.74	0.64

Biological Process (99) Tag Clouds

Scatterplot & Table Interactive Graph TreeMap

GROUP 34

Hide/show dispensable GO terms

Export results to text table (CSV)

Make R script for plotting

term ID	description	frequency	pin?	log_{10} p-value	uniqueness	dispensability
GO:0009269	response to desiccation	0.073 %		-2.9070	0.87	0.00
GO:0035303	regulation of dephosphorylation	0.060 %		-3.1718	0.73	0.00
GO:0035304	*regulation of protein dephosphorylation*	*0.047 %*		*-3.1993*	*0.71*	*0.95*
GO:0019220	*regulation of phosphate metabolic process*	*0.561 %*		*-1.9076*	*0.69*	*0.82*
GO:0010921	*regulation of phosphatase activity*	*0.035 %*		*-3.4776*	*0.71*	*0.67*
GO:0043666	*regulation of phosphoprotein phosphatase activity*	*0.022 %*		*-3.5172*	*0.71*	*0.93*
GO:0006470	*protein dephosphorylation*	*0.811 %*		*-1.7648*	*0.82*	*0.68*
GO:0080163	*regulation of protein serine/threonine phosphatase activity*	*0.017 %*		*-3.6971*	*0.71*	*0.89*
GO:0071705	nitrogen compound transport	1.515 %		-2.4616	0.80	0.00
GO:0006811	*ion transport*	*4.174 %*		*-1.1988*	*0.78*	*0.52*
GO:0071702	*organic substance transport*	*5.585 %*		*-1.3069*	*0.78*	*0.63*
GO:0009808	lignin metabolic process	0.285 %		-1.6633	0.84	0.06
GO:0009698	*phenylpropanoid metabolic process*	*0.505 %*		*-1.2376*	*0.83*	*0.69*
GO:0015979	photosynthesis	1.057 %		-1.1362	0.92	0.08
GO:0019048	modulation by virus of host morphology or physiology	0.017 %		-1.6747	0.83	0.11
GO:0035821	*modification of morphology or physiology of other organism*	*1.385 %*		*-1.0076*	*0.81*	*0.51*
GO:0051817	*modification of morphology or physiology of other organism involved in symbiotic interaction*	*0.129 %*		*-1.0423*	*0.83*	*0.72*
GO:0044003	*modification by symbiont of host morphology or physiology*	*0.125 %*		*-1.0502*	*0.82*	*0.87*
GO:0061062	regulation of nematode larval development	0.009 %		-1.9502	0.79	0.13

GO:0002119	nematode larval development	0.009 %	-1.9502	0.88	1.00
GO:0051336	regulation of hydrolase activity	0.781 %	-2.1828	0.80	0.15
GO:0050790	regulation of catalytic activity	2.257 %	-1.5180	0.79	0.84
GO:0009624	response to nematode	0.306 %	-2.3066	0.90	0.15
GO:0045730	respiratory burst	0.013 %	-1.5513	0.89	0.19
GO:0065009	regulation of molecular function	2.443 %	-1.1796	0.82	0.21
GO:0023051	regulation of signaling	1.126 %	-1.0520	0.79	0.22
GO:0042549	photosystem II stabilization	0.030 %	-1.9280	0.79	0.22
GO:0042548	regulation of photosynthesis, light reaction	0.108 %	-1.1008	0.77	0.90
GO:0043467	regulation of generation of precursor metabolites and energy	0.117 %	-1.0582	0.78	0.56
GO:0009605	response to external stimulus	6.366 %	-1.1270	0.89	0.23
GO:0043484	regulation of RNA splicing	0.108 %	-2.0244	0.78	0.24
GO:0010646	regulation of cell communication	1.139 %	-1.0476	0.77	0.24
GO:0001944	vasculature development	0.108 %	-1.9280	0.87	0.25
GO:0072358	cardiovascular system development	0.108 %	-1.9280	0.87	1.00
GO:0006521	regulation of cellular amino acid metabolic process	0.060 %	-1.6869	0.72	0.26
GO:0033238	regulation of cellular amine metabolic process	0.060 %	-1.5253	0.79	0.78
GO:0009395	phospholipid catabolic process	0.052 %	-1.7391	0.79	0.28
GO:0002164	larval development	0.009 %	-1.9502	0.90	0.29
GO:0009943	adaxial/abaxial axis specification	0.108 %	-1.2519	0.88	0.30
GO:0009955	adaxial/abaxial pattern specification	0.151 %	-1.0663	0.88	0.76
GO:0006979	response to oxidative stress	1.908 %	-1.0381	0.90	0.30
GO:0046717	acid secretion	0.026 %	-2.2734	0.78	0.31
GO:0051174	regulation of phosphorus metabolic process	0.561 %	-1.9076	0.74	0.33
GO:0010358	leaf shaping	0.035 %	-1.5790	0.89	0.33
GO:0010107	potassium ion import	0.022 %	-1.3902	0.73	0.33
GO:0071805	potassium ion transmembrane transport	0.319 %	-1.1192	0.69	0.81
GO:0071804	cellular potassium ion transport	0.319 %	-1.1192	0.69	0.92
GO:0031399	regulation of protein modification process	0.699 %	-1.7918	0.73	0.33
GO:0032268	regulation of cellular protein metabolic process	1.921 %	-1.0617	0.71	0.85
GO:0042538	hyperosmotic salinity response	0.220 %	-1.6934	0.90	0.35
GO:0006972	hyperosmotic response	0.263 %	-1.5720	0.90	0.60
GO:0072359	circulatory system development	0.108 %	-1.9280	0.88	0.36
GO:0042886	amide transport	0.220 %	-2.4252	0.76	0.37
GO:0006868	glutamine transport	0.026 %	-2.2734	0.70	0.57
GO:0015804	neutral amino acid transport	0.086 %	-1.6085	0.69	0.77
GO:0006865	amino acid transport	0.419 %	-1.0861	0.66	0.79
GO:0006857	oligopeptide transport	0.155 %	-2.4363	0.75	0.66
GO:0015833	peptide transport	0.177 %	-2.4363	0.75	0.95
GO:0015837	amine transport	0.043 %	-2.2278	0.77	0.60
GO:0010585	glutamine secretion	0.004 %	-2.2734	0.70	0.77
GO:0032890	regulation of organic acid transport	0.030 %	-2.2278	0.64	0.61
GO:0030001	metal ion transport	1.588 %	-1.2169	0.75	0.58
GO:0032973	amino acid export	0.082 %	-1.7391	0.69	0.72
GO:0051955	regulation of amino acid transport	0.030 %	-2.2278	0.61	1.00
GO:0080143	regulation of amino acid export	0.030 %	-2.2278	0.61	0.67
GO:0051952	regulation of amine transport	0.030 %	-2.2278	0.65	0.96
GO:0007584	response to nutrient	0.047 %	-1.3127	0.89	0.38
GO:0031667	response to nutrient levels	0.639 %	-1.1175	0.89	0.76
GO:0006606	protein import into nucleus	0.242 %	-1.2184	0.70	0.39
GO:1902593	single-organism nuclear import	0.242 %	-1.2184	0.72	0.99
GO:0015849	organic acid transport	0.682 %	-1.0861	0.72	0.51
GO:0044744	protein targeting to nucleus	0.246 %	-1.2184	0.75	0.98
GO:0034504	protein localization to nucleus	0.268 %	-1.1802	0.80	0.74
GO:0051170	nuclear import	0.246 %	-1.1554	0.79	0.89
GO:0006102	isocitrate metabolic process	0.043 %	-1.5886	0.86	0.39
GO:0009789	positive regulation of abscisic acid-activated signaling pathway	0.147 %	-2.2336	0.63	0.41
GO:0023056	positive regulation of signaling	0.332 %	-1.8356	0.72	0.73
GO:0097306	cellular response to alcohol	1.170 %	-1.1078	0.80	0.80
GO:0009738	abscisic acid-activated signaling pathway	1.062 %	-1.3011	0.62	0.79
GO:0048584	positive regulation of response to stimulus	0.902 %	-1.1796	0.71	0.69
GO:0010647	positive regulation of cell communication	0.350 %	-1.8095	0.74	0.73
GO:0071215	cellular response to abscisic acid stimulus	1.170 %	-1.1078	0.79	0.98
GO:0009787	regulation of abscisic acid-activated signaling pathway	0.350 %	-1.6064	0.61	0.93
GO:0009967	positive regulation of signal transduction	0.332 %	-1.8356	0.64	0.93
GO:0009966	regulation of signal transduction	1.109 %	-1.0608	0.62	0.87
GO:1901419	regulation of response to alcohol	0.350 %	-1.6064	0.71	0.59
GO:1901421	positive regulation of response to alcohol	0.147 %	-2.2336	0.70	0.86
GO:0010200	response to chitin	0.544 %	-1.2013	0.86	0.44
GO:0016311	dephosphorylation	1.515 %	-1.5267	0.86	0.44
GO:0072350	tricarboxylic acid metabolic process	0.259 %	-1.5513	0.84	0.45
GO:0031323	regulation of cellular metabolic process	13.906 %	-1.9446	0.68	0.47
GO:0080090	regulation of primary metabolic process	13.681 %	-1.6022	0.70	0.82

L.G0:0060255	regufation of macromolEl'cule metaboUc process	14.091%	-1.1043	0.69	0.83
L.G0:0019222	regufation of metabolic process	14.847%	-1.6338	0.74	0.55
G0:0046434	organophosphate catabolic process	0.112 %	-1.1950	0.88	0.49
G0:0009247	glycolipid biosynthetic process	0.250 %	-1.3552	0.81	0.49
.G0:0046467	membrane lfpid biosynthetic process	0.371%	-11913	0.81	0.91
·	glycolipid metabolic process	0.306%	-1.1192	0.81	0.98
L.	liposacchar ide metabolic process	0.315%	-1 0719	0.84	0.56

Biological Process (154) **Tag Clouds**

Scatterplot & Table **Interactive Graph** **TreeMap**

GROUP 35

Hide/show dispensable GO terms

Export results to text table (CSV)

Make R script for plotting

other organism involved in symbiotic interaction

interaction

term ID	description	frequency	pin?	log_{10} p-value	uniqueness	dispensability
GO:0002376	immune system process	1.532 %		-1.1001	0.99	0.00
GO:0010230	alternative respiration	0.009 %		-3.1671	0.87	0.00
GO:0009765	*photosynthesis, light harvesting*	*0.160 %*		*-2.1623*	*0.92*	*0.53*
GO:0019684	*photosynthesis, light reaction*	*0.505 %*		*-1.3799*	*0.92*	*0.77*
GO:0052173	response to defenses of other organism involved in symbiotic interaction	0.060 %		-2.7130	0.68	0.00
GO:0052572	*response to host immune response*	*0.056 %*		*-2.7130*	*0.63*	*1.00*
GO:0052031	*modulation by symbiont of host defense response*	*0.060 %*		*-2.7130*	*0.51*	*0.99*
GO:0052308	*pathogen-associated molecular pattern dependent modulation by organism of innate immune response in*	*0.043 %*		*-2.7883*	*0.50*	*1.00*
GO:0052033	*pathogen-associated molecular pattern dependent induction by symbiont of host innate immune response*	*0.043 %*		*-2.7883*	*0.48*	*0.83*
GO:0052306	*modulation by organism of innate immune response in other organism involved in symbiotic interaction*	*0.043 %*		*-2.7883*	*0.50*	*0.97*
GO:0052305	*positive regulation by organism of innate immune response in other organism involved in symbiotic*	*0.043 %*		*-2.7883*	*0.48*	*1.00*
GO:0052553	*modulation by symbiont of host immune response*	*0.056 %*		*-2.7130*	*0.49*	*1.00*
GO:0052556	*positive regulation by symbiont of host immune response*	*0.056 %*		*-2.7130*	*0.47*	*0.98*
GO:0052555	*positive regulation by organism of immune response of other organism involved in symbiotic interaction*	*0.056 %*		*-2.7130*	*0.48*	*1.00*
GO:0035821	*modification of morphology or physiology of other organism*	*1.385 %*		*-2.1999*	*0.70*	*0.56*
GO:0052552	*modulation by organism of immune response of other organism involved in symbiotic interaction*	*0.056 %*		*-2.7130*	*0.49*	*1.00*
GO:0002684	*positive regulation of immune system process*	*0.255 %*		*-1.6644*	*0.68*	*0.90*
GO:0051701	*interaction with host*	*0.177 %*		*-1.9930*	*0.74*	*0.84*

other organism involved in symbiotic interaction

GO:0009638	phototropism	0.069 %	-1.2753	0.76	0.54
GO:0009646	response to absence of light	0.142 %	-1.1730	0.82	0.57
GO:0071483	cellular response to blue light	0.104 %	-1.1338	0.77	0.80
GO:0002682	regulation of immune system process	0.453 %	-1.3605	0.74	0.16
GO:0045087	innate immune response	1.364 %	-1.2284	0.69	0.85
GO:0006955	immune response	1.398 %	-1.1994	0.72	0.96
GO:0043412	macromolecule modification	15.900 %	-1.7600	0.94	0.17
GO:0032970	regulation of actin filament-based process	0.194 %	-1.0879	0.73	0.19
GO:0010036	response to boron-containing substance	0.022 %	-1.4838	0.87	0.22
GO:0065008	regulation of biological quality	5.991 %	-1.3326	0.85	0.22
GO:0015700	arsenite transport	0.022 %	-1.4752	0.96	0.23
GO:0046685	response to arsenic-containing substance	0.060 %	-1.2547	0.86	0.23
GO:0030522	intracellular receptor signaling pathway	0.078 %	-1.2399	0.75	0.23
GO:0009071	serine family amino acid catabolic process	0.043 %	-1.5397	0.84	0.27
GO:0006546	glycine catabolic process	0.030 %	-1.5397	0.84	0.75
GO:0006544	glycine metabolic process	0.091 %	-1.2701	0.83	0.80
GO:1901606	alpha-amino acid catabolic process	0.263 %	-1.0438	0.82	0.86
GO:0006400	tRNA modification	0.293 %	-1.3904	0.89	0.28
GO:0008033	tRNA processing	0.479 %	-1.0621	0.90	0.86
GO:0031334	positive regulation of protein complex assembly	0.190 %	-1.4423	0.65	0.29
GO:0051495	positive regulation of cytoskeleton organization	0.138 %	-1.3904	0.67	0.86
GO:0051493	regulation of cytoskeleton organization	0.289 %	-1.0123	0.71	0.69
GO:0032271	regulation of protein polymerization	0.186 %	-1.1120	0.70	0.92
GO:0032273	positive regulation of protein polymerization	0.108 %	-1.4423	0.66	0.92
GO:0030832	regulation of actin filament length	0.181 %	-1.0879	0.61	0.98
GO:0030833	regulation of actin filament polymerization	0.164 %	-1.1120	0.58	0.95
GO:0010638	positive regulation of organelle organization	0.207 %	-1.1648	0.67	0.89
GO:0045010	actin nucleation	0.095 %	-1.4423	0.58	0.91
GO:0008064	regulation of actin polymerization or depolymerization	0.181 %	-1.0879	0.61	0.99
GO:0044089	positive regulation of cellular component biogenesis	0.246 %	-1.2497	0.70	0.85
GO:0032956	regulation of actin cytoskeleton organization	0.194 %	-1.0879	0.66	0.99
GO:0030041	actin filament polymerization	0.168 %	-1.0947	0.72	0.93
GO:0030838	positive regulation of actin filament polymerization	0.095 %	-1.4423	0.58	0.98
GO:0043254	regulation of protein complex assembly	0.298 %	-1.1120	0.69	0.89
GO:0016579	protein deubiquitination	0.246 %	-1.5298	0.91	0.32
GO:0070646	protein modification by small protein removal	0.328 %	-1.2807	0.90	0.51
GO:0080029	cellular response to boron-containing substance levels	0.017 %	-1.4838	0.77	0.33
GO:0009743	response to carbohydrate	0.518 %	-1.1397	0.83	0.37
GO:0044085	cellular component biogenesis	5.783 %	-1.0047	0.88	0.38
GO:0009606	tropism	0.341 %	-1.4067	0.80	0.39
GO:0051592	response to calcium ion	0.052 %	-1.3569	0.86	0.40
GO:0009750	response to fructose	0.069 %	-1.0321	0.85	0.41
GO:0006869	lipid transport	0.596 %	-1.1015	0.89	0.41
GO:0072657	protein localization to membrane	0.259 %	-1.0235	0.78	0.80
GO:0044765	single-organism transport	5.287 %	-1.1946	0.87	0.68
GO:0006612	protein targeting to membrane	0.168 %	-1.1049	0.78	0.96
GO:0090150	establishment of protein localization to membrane	0.259 %	-1.0235	0.78	0.53
GO:0010105	negative regulation of ethylene-activated signaling pathway	0.073 %	-1.7557	0.60	0.42
GO:0048584	positive regulation of response to stimulus	0.902 %	-1.0760	0.62	0.69
GO:0010104	regulation of ethylene-activated signaling pathway	0.095 %	-1.4584	0.61	0.98
GO:1902531	regulation of intracellular signal transduction	0.229 %	-1.1015	0.62	0.67
GO:1902532	negative regulation of intracellular signal transduction	0.091 %	-1.7557	0.61	0.85
GO:0070298	negative regulation of phosphorelay signal transduction system	0.073 %	-1.7557	0.62	0.97
GO:0070297	regulation of phosphorelay signal transduction system	0.095 %	-1.4584	0.63	0.87
GO:0031349	positive regulation of defense response	0.341 %	-1.6499	0.61	0.56
GO:0070887	cellular response to chemical stimulus	5.287 %	-1.0632	0.75	0.45
GO:0000724	double-strand break repair via homologous recombination	0.345 %	-1.4752	0.73	0.45
GO:0006302	double-strand break repair	0.483 %	-1.2547	0.73	0.77
GO:0036294	cellular response to decreased oxygen levels	0.129 %	-1.0748	0.76	0.99
GO:0071456	cellular response to hypoxia	0.117 %	-1.1155	0.74	0.51
GO:0071453	cellular response to oxygen levels	0.129 %	-1.0748	0.77	0.87
GO:0000725	recombinational repair	0.350 %	-1.4752	0.73	0.88
GO:1902578	single-organism localization	5.481 %	-1.1488	0.89	0.46
GO:0090066	regulation of anatomical structure size	0.319 %	-1.6173	0.80	0.46
GO:0031163	metallo-sulfur cluster assembly	0.142 %	-1.1049	0.85	0.48
GO:0015846	polyamine transport	0.026 %	-2.0549	0.91	0.49

Biological Process (176) Tag Clouds

Scatterplot & Table Interactive Graph TreeMap

GROUP 36

Tip: your resulting list of GO terms seems to be quite long. If you want to reduce it further, press the Back button in your browser and choose a different setting for the "allowed similarity" parameter.

Hide/show dispensable GO terms

Export results to text table (CSV) Make R script for plotting

term ID	description	frequency	pin?	log_{10} p-value	uniqueness	dispensability
GO:0006629	lipid metabolic process	4.683 %		-6.6071	0.77	0.00
GO:0009639	response to red or far red light	0.872 %		-2.6760	0.90	0.00
GO:0010114	*response to red light*	*0.259 %*		*-2.1361*	*0.91*	*0.65*
GO:0009314	*response to radiation*	*2.892 %*		*-1.1205*	*0.90*	*0.56*
GO:0009640	*photomorphogenesis*	*0.311 %*		*-1.7915*	*0.73*	*0.80*
GO:0009647	*skotomorphogenesis*	*0.026 %*		*-1.0529*	*0.77*	*0.54*
GO:0009416	*response to light stimulus*	*2.771 %*		*-1.1341*	*0.89*	*0.83*
GO:0009834	plant-type secondary cell wall biogenesis	0.263 %		-1.4558	0.92	0.00
GO:0042546	*cell wall biogenesis*	*0.928 %*		*-1.0879*	*0.91*	*0.73*
GO:0009832	*plant-type cell wall biogenesis*	*0.626 %*		*-1.2278*	*0.91*	*0.84*
GO:0009828	*plant-type cell wall loosening*	*0.022 %*		*-1.4557*	*0.93*	*0.61*
GO:0071669	*plant-type cell wall organization or biogenesis*	*1.152 %*		*-1.2117*	*0.93*	*0.64*
GO:0032502	developmental process	12.689 %		-1.0139	0.99	0.00
GO:0044699	single-organism process	41.709 %		-5.5944	0.99	0.00
GO:0048609	multicellular organismal reproductive process	0.587 %		-2.3382	0.84	0.00
GO:0007292	*female gamete generation*	*0.022 %*		*-1.0222*	*0.80*	*0.75*

GO:0007281	germ cell development	0.004 %	-1.2561	0.76	0.67
GO:0007276	gamete generation	0.203 %	-1.8290	0.77	0.89
GO:0022412	cellular process involved in reproduction in multicellular organism	0.177 %	-1.9420	0.74	0.88
GO:0048235	pollen sperm cell differentiation	0.147 %	-1.3031	0.70	0.87
GO:0048232	male gamete generation	0.177 %	-1.1896	0.77	0.96
GO:0048477	oogenesis	0.009 %	-1.2561	0.75	0.70
GO:0051179	localization	12.352 %	-1.3379	0.99	0.00
GO:0019079	viral genome replication	0.026 %	-1.0529	0.92	0.03
GO:0019058	viral life cycle	0.030 %	-1.0424	0.92	0.70
GO:0010166	wax metabolic process	0.082 %	-3.2121	0.96	0.05
GO:0018149	peptide cross-linking	0.004 %	-4.4756	0.94	0.06
GO:0009299	mRNA transcription	0.060 %	-2.5507	0.88	0.08
GO:0009056	catabolic process	8.118 %	-2.1822	0.95	0.09
GO:0009399	nitrogen fixation	0.017 %	-1.3780	0.95	0.12
GO:0006869	lipid transport	0.596 %	-4.9527	0.78	0.12
GO:0051234	establishment of localization	11.817 %	-1.5918	0.85	0.86
GO:0006810	transport	11.649 %	-1.7517	0.85	0.52
GO:0044765	single-organism transport	5.287 %	-1.3510	0.74	0.74
GO:0015849	organic acid transport	0.682 %	-1.3347	0.78	0.55
GO:0010876	lipid localization	0.708 %	-4.3953	0.88	0.59
GO:1902578	single-organism localization	5.481 %	-1.5892	0.77	0.73
GO:0010025	wax biosynthetic process	0.078 %	-2.2605	0.93	0.15
GO:0018958	phenol-containing compound metabolic process	0.190 %	-1.9393	0.87	0.16
GO:0046189	phenol-containing compound biosynthetic process	0.104 %	-1.9393	0.84	0.66
GO:0009807	lignan biosynthetic process	0.022 %	-1.4282	0.73	0.95
GO:0009802	cinnamic acid ester biosynthetic process	0.004 %	-1.6292	0.73	0.93
GO:0090431	alkyl caffeate ester biosynthetic process	0.004 %	-1.6292	0.70	0.74
GO:1901599	(-)-pinoresinol biosynthetic process	0.009 %	-1.6292	0.72	0.77
GO:1901598	(-)-pinoresinol metabolic process	0.009 %	-1.5511	0.74	0.90
GO:0033494	ferulate metabolic process	0.004 %	-1.6292	0.77	0.74
GO:0009712	catechol-containing compound metabolic process	0.004 %	-1.6292	0.88	0.74
GO:0009713	catechol-containing compound biosynthetic process	0.004 %	-1.6292	0.86	1.00
GO:0046688	response to copper ion	0.069 %	-2.4442	0.93	0.17
GO:0009624	response to nematode	0.306 %	-2.5581	0.94	0.19
GO:0010143	cutin biosynthetic process	0.091 %	-1.5998	0.91	0.20
GO:0044710	single-organism metabolic process	18.390 %	-3.1820	0.83	0.21
GO:0042401	cellular biogenic amine biosynthetic process	0.186 %	-1.6911	0.83	0.21
GO:0006576	cellular biogenic amine metabolic process	0.255 %	-1.2680	0.85	0.94
GO:0008295	spermidine biosynthetic process	0.039 %	-1.0978	0.83	0.91
GO:0006597	spermine biosynthetic process	0.026 %	-1.3126	0.83	0.86
GO:0008215	spermine metabolic process	0.035 %	-1.3126	0.85	0.84
GO:0009309	amine biosynthetic process	0.186 %	-1.6911	0.83	0.83
GO:0046219	indolalkylamine biosynthetic process	0.095 %	-1.1352	0.82	0.96
GO:0000162	tryptophan biosynthetic process	0.095 %	-1.1352	0.69	0.95
GO:0044106	cellular amine metabolic process	0.306 %	-1.1117	0.85	0.87
GO:0006083	acetate metabolic process	0.009 %	-1.2741	0.80	0.22
GO:0080175	phragmoplast microtubule organization	0.017 %	-1.3126	0.85	0.23
GO:0031539	positive regulation of anthocyanin metabolic process	0.022 %	-1.4282	0.84	0.24
GO:0048314	embryo sac morphogenesis	0.009 %	-2.1485	0.81	0.27
GO:0055046	microgametogenesis	0.216 %	-1.9126	0.79	0.52
GO:0010102	lateral root morphogenesis	0.229 %	-1.3276	0.77	0.57
GO:0010101	post-embryonic root morphogenesis	0.233 %	-1.3276	0.77	0.90
GO:0009553	embryo sac development	0.565 %	-1.5088	0.78	0.71
GO:0048598	embryonic morphogenesis	0.125 %	-1.8452	0.79	0.50
GO:0048646	anatomical structure formation involved in morphogenesis	0.755 %	-1.0486	0.86	0.64
GO:0009886	post-embryonic animal morphogenesis	0.526 %	-1.3780	0.77	0.54
GO:0010086	embryonic root morphogenesis	0.013 %	-1.1485	0.81	0.73
GO:0080159	zygote elongation	0.009 %	-1.3333	0.76	0.71
GO:0010015	root morphogenesis	0.967 %	-1.1463	0.75	0.77
GO:1901362	organic cyclic compound biosynthetic process	14.769 %	-1.3049	0.86	0.28
GO:0019438	aromatic compound biosynthetic process	14.247 %	-1.2148	0.84	0.55
GO:0032456	endocytic recycling	0.022 %	-1.4557	0.91	0.28
GO:0009739	response to gibberellin	0.626 %	-2.3286	0.89	0.30
GO:0009742	brassinosteroid mediated signaling pathway	0.306 %	-1.3661	0.77	0.72
GO:0009741	response to brassinosteroid	0.410 %	-1.5143	0.88	0.63
GO:0009740	gibberellic acid mediated signaling pathway	0.341 %	-2.0825	0.77	0.62
GO:0048545	response to steroid hormone	0.306 %	-1.3661	0.89	0.72
GO:0010476	gibberellin mediated signaling pathway	0.345 %	-1.9541	0.77	0.99
GO:0043401	steroid hormone mediated signaling pathway	0.306 %	-1.3661	0.77	1.00
GO:0071367	cellular response to brassinosteroid stimulus	0.315 %	-1.3629	0.85	0.95
GO:0071370	cellular response to gibberellin stimulus	0.354 %	-1.8727	0.85	0.89
GO:0071383	cellular response to steroid hormone stimulus	0.306 %	-1.3661	0.85	1.00

GO:0009653	anatomical structure morphogenesis	3.500 %	-1.3788	0.87	0.30
GO:0044707	single-multicellular organism process	10.699 %	-1.2096	0.75	0.86
GO:0044767	single-organism developmental process	12.123 %	-1.1380	0.76	0.82
GO:0048856	anatomical structure development	11.722 %	-1.1259	0.86	0.85
GO:0007275	multicellular organism development	10.423 %	-1.3504	0.72	0.67
GO:0048229	gametophyte development	1.653 %	-1.1875	0.77	0.51
GO:0010076	maintenance of floral meristem identity	0.026 %	-1.2929	0.82	0.33
GO:0010077	maintenance of inflorescence meristem identity	0.039 %	-1.1623	0.82	0.80
GO:0009698	phenylpropanoid metabolic process	0.505 %	-4.5252	0.73	0.33
GO:0009806	lignan metabolic process	0.022 %	-1.4282	0.76	0.74
GO:0009808	lignin metabolic process	0.285 %	-1.1970	0.72	0.91
GO:0009801	cinnamic acid ester metabolic process	0.009 %	-1.5511	0.77	0.53
GO:0009803	cinnamic acid metabolic process	0.026 %	-1.2929	0.72	0.75
GO:0010345	suberin biosynthetic process	0.060 %	-1.5844	0.72	0.80
GO:0044550	secondary metabolite biosynthetic process	1.183 %	-3.2758	0.72	0.80
GO:0009699	phenylpropanoid biosynthetic process	0.337 %	-3.6263	0.69	0.70
GO:0046274	lignin catabolic process	0.078 %	-2.7729	0.72	0.82
GO:0046271	phenylpropanoid catabolic process	0.078 %	-2.6992	0.72	0.82
GO:0007034	vacuolar transport	0.293 %	-2.7556	0.89	0.35
GO:0000914	phragmoplast assembly	0.009 %	-1.4023	0.84	0.35
GO:1902407	assembly of actomyosin apparatus involved in mitotic cytokinesis	0.009 %	-1.4023	0.84	1.00
GO:0000912	assembly of actomyosin apparatus involved in cytokinesis	0.009 %	-1.4023	0.84	0.66
GO:0006557	S-adenosylmethioninamine biosynthetic process	0.017 %	-1.3780	0.75	0.35
GO:0046499	S-adenosylmethioninamine metabolic process	0.017 %	-1.3780	0.76	0.71
GO:0050982	detection of mechanical stimulus	0.017 %	-1.5168	0.93	0.36
GO:0016042	lipid catabolic process	1.032 %	-4.1033	0.75	0.36
GO:0010430	fatty acid omega-oxidation	0.004 %	-1.5883	0.79	0.53
GO:0044712	single-organism catabolic process	2.676 %	-2.9157	0.77	0.56
GO:0030148	sphingolipid biosynthetic process	0.117 %	-1.0861	0.73	0.52
GO:0044255	cellular lipid metabolic process	3.224 %	-1.4082	0.72	0.82
GO:0006631	fatty acid metabolic process	1.101 %	-1.1619	0.71	0.71
GO:1901575	organic substance catabolic process	7.402 %	-2.0523	0.90	0.74
GO:0046459	short-chain fatty acid metabolic process	0.013 %	-1.2065	0.78	0.56
GO:0019432	triglyceride biosynthetic process	0.104 %	-1.0030	0.75	0.53
GO:0008610	lipid biosynthetic process	2.464 %	-2.5237	0.72	0.71
GO:0019605	butyrate metabolic process	0.009 %	-1.2389	0.78	0.55
GO:0031408	oxylipin biosynthetic process	0.099 %	-1.9272	0.73	0.65
GO:0031407	oxylipin metabolic process	0.104 %	-2.8790	0.75	0.51
GO:0001944	vasculature development	0.108 %	-1.3333	0.80	0.37
GO:0072358	cardiovascular system development	0.108 %	-1.3333	0.80	1.00
GO:0072359	circulatory system development	0.108 %	-1.3333	0.81	0.37
GO:0015669	gas transport	0.009 %	-1.4023	0.83	0.38
GO:0015671	oxygen transport	0.009 %	-1.7826	0.83	0.38
GO:0048859	formation of anatomical boundary	0.069 %	-1.4268	0.82	0.39
GO:0019748	secondary metabolic process	1.938 %	-3.4503	0.80	0.39
GO:0071456	cellular response to hypoxia	0.117 %	-1.4979	0.85	0.41
GO:0036294	cellular response to decreased oxygen levels	0.129 %	-1.4207	0.86	0.99
GO:0071453	cellular response to oxygen levels	0.129 %	-1.4207	0.86	0.87
GO:1990267	response to transition metal nanoparticle	2.017 %	-1.2424	0.91	0.41
GO:0006002	fructose 6-phosphate metabolic process	0.060 %	-1.0322	0.93	0.41
GO:0051762	sesquiterpene biosynthetic process	0.009 %	-1.3333	0.78	0.42
GO:0051761	sesquiterpene metabolic process	0.009 %	-1.3333	0.81	0.78
GO:0006097	glyoxylate cycle	0.030 %	-1.0978	0.77	0.43
GO:0046487	glyoxylate metabolic process	0.035 %	-1.0747	0.78	0.72
GO:0006880	intracellular sequestering of iron ion	0.060 %	-2.1485	0.72	0.44
GO:0055076	transition metal ion homeostasis	0.678 %	-1.5757	0.92	0.76
GO:0055072	iron ion homeostasis	0.237 %	-1.4392	0.92	0.86
GO:0051238	sequestering of metal ion	0.104 %	-1.1623	0.86	0.81
GO:0097577	sequestering of iron ion	0.060 %	-2.1485	0.74	0.93
GO:0048878	chemical homeostasis	1.765 %	-1.0361	0.92	0.78
GO:0046916	cellular transition metal ion homeostasis	0.505 %	-1.2299	0.77	0.92
GO:0080144	amino acid homeostasis	0.060 %	-2.1013	0.93	0.58
GO:0043090	amino acid import	0.056 %	-1.7556	0.77	0.44
GO:0015827	tryptophan transport	0.004 %	-1.4023	0.80	0.62
GO:0015800	acidic amino acid transport	0.035 %	-1.0529	0.78	0.71
GO:0015804	neutral amino acid transport	0.086 %	-1.0222	0.76	0.79
GO:0015801	aromatic amino acid transport	0.017 %	-1.4023	0.79	0.68
GO:0015802	basic amino acid transport	0.035 %	-1.1099	0.78	0.71
GO:0006865	amino acid transport	0.419 %	-1.3347	0.74	0.76
GO:1905039	carboxylic acid transmembrane transport	0.505 %	-1.0747	0.74	0.92

GO:0015809	arginine transport	0.017 %	-1.1224	0.79	0.68
GO:0015810	aspartate transport	0.009 %	-1.4023	0.79	0.65
GO:0032973	amino acid export	0.082 %	-1.1485	0.76	0.79
GO:1903825	organic acid transmembrane transport	0.505 %	-1.0747	0.74	0.94
GO:0003333	amino acid transmembrane transport	0.337 %	-1.0747	0.74	0.96
GO:0080160	selenate transport	0.077 %	-1.2224	0.86	0.56
GO:0010540	basipetal auxin transport	0.078 %	-1.0635	0.78	0.45
GO:0010440	stomatal lineage progression	0.069 %	-1.0424	0.76	0.47
GO:0051607	defense response to virus	0.203 %	-1.3135	0.93	0.48
GO:0034050	host programmed cell death induced by symbiont	0.345 %	-1.1556	0.83	0.90
GO:0009626	plant-type hypersensitive response	0.341 %	-1.2027	0.77	0.69
GO:0045490	pectin catabolic process	0.423 %	-2.1753	0.89	0.48
GO:0000272	polysaccharide catabolic process	0.777 %	-1.1425	0.89	0.85
GO:0010393	galacturonan metabolic process	0.652 %	-1.6305	0.91	0.74
GO:0045488	pectin metabolic process	0.647 %	-1.6553	0.91	0.95
GO:0044711	single-organism biosynthetic process	7.549 %	-2.2888	0.75	0.49
GO:0032504	multicellular organism reproduction	0.613 %	-2.1875	0.87	0.49
GO:0019953	sexual reproduction	0.596 %	-1.1425	0.92	0.50

Biological Process (317) Tag Clouds

Scatterplot & Table Interactive Graph TreeMap

GROUP 37

Tip: your resulting list of GO terms seems to be quite long. If you want to reduce it further, press the Back button in your browser and choose a different setting for the "allowed similarity" parameter.

Hide/show dispensable GO terms

Export results to text table (CSV)

Make R script for plotting

term ID	description	frequency	pin?	log_{10} p-value	uniqueness	dispensability
GO:0000003	reproduction	6.655 %		-5.1434	1.00	0.00
GO:0010228	vegetative to reproductive phase transition of meristem	0.738 %		-14.0121	0.79	0.00
GO:0044702	*single organism reproductive process*	*5.939 %*		*-1.0706*	*0.85*	*0.91*
GO:0099402	*plant organ development*	*3.763 %*		*-3.0494*	*0.78*	*0.77*
GO:0003006	*developmental process involved in reproduction*	*5.611 %*		*-6.0271*	*0.81*	*0.85*
GO:0010218	*response to far red light*	*0.250 %*		*-3.4949*	*0.82*	*0.80*
GO:0048586	*regulation of long-day photoperiodism, flowering*	*0.086 %*		*-5.0892*	*0.59*	*0.86*
GO:0048575	*short-day photoperiodism, flowering*	*0.056 %*		*-2.0330*	*0.70*	*0.83*
GO:0048573	*photoperiodism, flowering*	*0.388 %*		*-8.1221*	*0.66*	*0.56*
GO:0048574	*long-day photoperiodism, flowering*	*0.121 %*		*-4.4771*	*0.68*	*0.95*
GO:0048579	*negative regulation of long-day photoperiodism, flowering*	*0.039 %*		*-1.2664*	*0.58*	*0.90*
GO:0010114	*response to red light*	*0.259 %*		*-1.5411*	*0.82*	*0.80*
GO:0048578	*positive regulation of long-day photoperiodism, flowering*	*0.030 %*		*-4.4790*	*0.57*	*0.89*
GO:0048571	*long-day photoperiodism*	*0.138 %*		*-4.1257*	*0.82*	*0.90*
GO:0048572	*short-day photoperiodism*	*0.056 %*		*-2.0330*	*0.83*	*0.83*
GO:0048608	*reproductive structure development*	*4.674 %*		*-6.7611*	*0.75*	*0.73*

GO:0009642	response to light intensity	0.587 %	-1.2463	0.81	0.71
GO:0009637	response to blue light	0.341 %	-3.5798	0.82	0.62
GO:0009639	response to red or far red light	0.872 %	-6.1335	0.81	0.68
GO:0010588	cotyledon vascular tissue pattern formation	0.060 %	-1.7225	0.80	0.91
GO:0009640	photomorphogenesis	0.311 %	-3.6917	0.69	0.62
GO:0009648	photoperiodism	0.427 %	-7.3960	0.82	0.63
GO:0010099	regulation of photomorphogenesis	0.104 %	-1.7806	0.60	0.74
GO:0010094	specification of carpel identity	0.013 %	-1.6493	0.81	0.77
GO:0048462	carpel formation	0.026 %	-1.3936	0.81	0.93
GO:0048467	gynoecium development	0.315 %	-1.6757	0.79	0.85
GO:0048466	androecium development	0.345 %	-1.2343	0.78	0.87
GO:0048440	carpel development	0.268 %	-1.9877	0.78	0.67
GO:0048443	stamen development	0.345 %	-1.2343	0.78	0.96
GO:0048445	carpel morphogenesis	0.035 %	-1.1228	0.81	0.83
GO:2000241	regulation of reproductive process	1.092 %	-1.0857	0.77	0.54
GO:0010016	shoot system morphogenesis	0.708 %	-2.7535	0.80	0.72
GO:2000028	regulation of photoperiodism, flowering	0.190 %	-5.8389	0.56	0.92
GO:2000030	regulation of response to red or far red light	0.134 %	-1.5648	0.71	0.76
GO:1905392	plant organ morphogenesis	1.485 %	-2.8498	0.79	0.77
GO:0060776	simple leaf morphogenesis	0.022 %	-2.7541	0.84	0.70
GO:0048825	cotyledon development	0.190 %	-2.5033	0.79	0.72
GO:0048826	cotyledon morphogenesis	0.073 %	-3.6638	0.80	0.77
GO:0048827	phyllome development	1.921 %	-4.3181	0.78	0.55
GO:0048367	shoot system development	3.599 %	-3.1138	0.79	0.76
GO:0048366	leaf development	1.368 %	-3.4228	0.78	0.79
GO:0048653	anther development	0.224 %	-1.7272	0.79	0.83
GO:0022414	reproductive process	6.629 %	-5.1933	0.90	0.90
GO:0090697	post-embryonic plant organ morphogenesis	0.419 %	-1.0121	0.80	0.77
GO:0009416	response to light stimulus	2.771 %	-4.5289	0.78	0.83
GO:0009965	leaf morphogenesis	0.380 %	-3.9672	0.80	0.67
GO:0061458	reproductive system development	4.674 %	-6.7611	0.78	0.80
GO:0032501	multicellular organismal process	11.360 %	-3.8848	0.99	0.00
GO:0032502	developmental process	12.689 %	-5.3701	0.99	0.00
GO:0044699	single-organism process	41.709 %	-2.3423	1.00	0.00
GO:0050896	response to stimulus	25.943 %	-4.6545	1.00	0.00
GO:0065007	biological regulation	28.554 %	-2.0868	1.00	0.00
GO:0071497	cellular response to freezing	0.013 %	-6.6798	0.83	0.00
GO:0050826	response to freezing	0.086 %	-4.9007	0.85	0.51
GO:0070417	cellular response to cold	0.147 %	-3.6060	0.80	0.60
GO:0009409	response to cold	1.580 %	-2.4524	0.81	0.69
GO:0010286	heat acclimation	0.207 %	-1.0113	0.84	0.71
GO:0031325	positive regulation of cellular metabolic process	2.741 %	-5.0993	0.66	0.03
GO:0032774	RNA biosynthetic process	11.813 %	-6.1641	0.77	0.82
GO:0048522	positive regulation of cellular process	3.582 %	-3.1659	0.70	0.87
GO:0080090	regulation of primary metabolic process	13.681 %	-4.3820	0.69	0.80
GO:0031323	regulation of cellular metabolic process	13.906 %	-4.2593	0.67	0.83
GO:1902680	positive regulation of RNA biosynthetic process	1.869 %	-3.0297	0.62	0.97
GO:0052318	regulation of phytoalexin metabolic process	0.009 %	-1.5776	0.77	0.81
GO:0052319	regulation of phytoalexin biosynthetic process	0.009 %	-1.5776	0.75	1.00
GO:0052320	positive regulation of phytoalexin metabolic process	0.009 %	-1.5776	0.73	1.00
GO:0031328	positive regulation of cellular biosynthetic process	2.102 %	-3.3359	0.64	0.96
GO:0052322	positive regulation of phytoalexin biosynthetic process	0.009 %	-1.5776	0.71	1.00
GO:0031326	regulation of cellular biosynthetic process	12.339 %	-5.2050	0.64	0.87
GO:0051252	regulation of RNA metabolic process	11.463 %	-5.8767	0.63	0.87
GO:0051247	positive regulation of protein metabolic process	0.721 %	-2.0734	0.66	0.81
GO:2001141	regulation of RNA biosynthetic process	11.282 %	-6.0876	0.61	0.88
GO:0019219	regulation of nucleobase-containing compound metabolic process	11.700 %	-5.5871	0.65	0.84
GO:0051254	positive regulation of RNA metabolic process	1.912 %	-3.0297	0.64	0.96
GO:0016070	RNA metabolic process	17.562 %	-3.4690	0.80	0.66
GO:0045935	positive regulation of nucleobase-containing compound metabolic process	2.007 %	-2.9185	0.65	0.96
GO:0010628	positive regulation of gene expression	2.050 %	-2.8312	0.67	0.92
GO:0006355	regulation of transcription, DNA-templated	11.234 %	-6.0876	0.61	0.59
GO:0006351	transcription, DNA-templated	11.709 %	-6.1641	0.76	0.87
GO:0045893	positive regulation of transcription, DNA-templated	1.864 %	-3.0297	0.62	0.91
GO:1901182	regulation of camalexin biosynthetic process	0.009 %	-1.5776	0.74	1.00
GO:1901183	positive regulation of camalexin biosynthetic process	0.009 %	-1.5776	0.70	0.53
GO:0010604	positive regulation of macromolecule metabolic process	2.672 %	-4.4339	0.66	0.95
GO:0044550	secondary metabolite biosynthetic process	1.183 %	-1.1245	0.81	0.56

GO:0010556	regulation of macromolecule biosynthetic process	12.076 %	-4.9208	0.64	0.87
GO:0010557	positive regulation of macromolecule biosynthetic process	2.007 %	-2.7771	0.64	0.97
GO:0009699	phenylpropanoid biosynthetic process	0.337 %	-1.0479	0.79	0.76
GO:2000243	positive regulation of reproductive process	0.246 %	-1.4036	0.73	0.62
GO:0010468	regulation of gene expression	12.706 %	-4.8311	0.67	0.79
GO:0043455	regulation of secondary metabolic process	0.173 %	-1.0774	0.75	0.72
GO:2000112	regulation of cellular macromolecule biosynthetic process	12.003 %	-4.9451	0.62	0.86
GO:0060255	regulation of macromolecule metabolic process	14.091 %	-4.3352	0.67	0.82
GO:1903508	positive regulation of nucleic acid-templated transcription	1.869 %	-3.0297	0.62	0.98
GO:1903506	regulation of nucleic acid-templated transcription	11.282 %	-6.0876	0.61	0.89
GO:0097659	nucleic acid-templated transcription	11.761 %	-6.1641	0.77	0.88
GO:0009893	positive regulation of metabolic process	2.892 %	-4.7330	0.69	0.84
GO:0009891	positive regulation of biosynthetic process	2.145 %	-3.2938	0.66	0.93
GO:0009889	regulation of biosynthetic process	12.408 %	-5.5733	0.66	0.76
GO:1900378	positive regulation of secondary metabolite biosynthetic process	0.017 %	-1.2989	0.71	0.79
GO:0051176	positive regulation of sulfur metabolic process	0.035 %	-1.3936	0.75	0.81
GO:0051173	positive regulation of nitrogen compound metabolic process	2.119 %	-3.3722	0.67	0.92
GO:0034654	nucleobase-containing compound biosynthetic process	13.056 %	-4.9886	0.79	0.80
GO:0051171	regulation of nitrogen compound metabolic process	12.430 %	-4.9981	0.68	0.78
GO:0034645	cellular macromolecule biosynthetic process	16.737 %	-1.9372	0.78	0.74
GO:0010497	plasmodesmata-mediated intercellular transport	0.056 %	-1.2510	0.95	0.04
GO:0009399	nitrogen fixation	0.017 %	-1.4385	0.95	0.04
GO:0032259	methylation	1.834 %	-2.2641	0.95	0.07
GO:0018027	peptidyl-lysine dimethylation	0.022 %	-4.7774	0.83	0.07
GO:0034968	histone lysine methylation	0.298 %	-2.9370	0.76	0.79
GO:0018205	peptidyl-lysine modification	0.725 %	-1.9673	0.82	0.59
GO:0043414	macromolecule methylation	0.928 %	-1.5106	0.83	0.60
GO:0016571	histone methylation	0.354 %	-2.4738	0.77	0.97
GO:0018022	peptidyl-lysine methylation	0.350 %	-2.9120	0.80	0.93
GO:0006479	protein methylation	0.475 %	-2.2943	0.81	0.97
GO:0032196	transposition	0.013 %	-3.7329	0.92	0.07
GO:0045490	pectin catabolic process	0.423 %	-2.3459	0.91	0.09
GO:0000272	polysaccharide catabolic process	0.777 %	-1.2897	0.90	0.85
GO:0045488	pectin metabolic process	0.647 %	-1.1869	0.91	0.95
GO:0010393	galacturonan metabolic process	0.652 %	-1.1687	0.91	0.74
GO:0009058	biosynthetic process	24.432 %	-1.8317	0.93	0.10
GO:0016311	dephosphorylation	1.515 %	-1.1687	0.91	0.11
GO:0042547	cell wall modification involved in multidimensional cell growth	0.017 %	-2.3687	0.87	0.13
GO:0016049	cell growth	1.519 %	-1.6947	0.87	0.60
GO:0045926	negative regulation of growth	0.121 %	-1.6046	0.82	0.66
GO:0030308	negative regulation of cell growth	0.043 %	-2.2510	0.73	0.50
GO:0009825	multidimensional cell growth	0.091 %	-1.3881	0.90	0.64
GO:0030418	nicotianamine biosynthetic process	0.022 %	-2.0855	0.83	0.13
GO:0072351	tricarboxylic acid biosynthetic process	0.022 %	-2.0855	0.85	0.70
GO:0030417	nicotianamine metabolic process	0.022 %	-2.0855	0.86	0.71
GO:0009739	response to gibberellin	0.626 %	-3.3843	0.81	0.14
GO:0071229	cellular response to acid chemical	2.158 %	-1.6803	0.76	0.64
GO:0042542	response to hydrogen peroxide	0.268 %	-1.1687	0.83	0.71
GO:0000302	response to reactive oxygen species	0.678 %	-1.8188	0.82	0.51
GO:0033993	response to lipid	3.267 %	-2.0806	0.80	0.58
GO:0071369	cellular response to ethylene stimulus	0.906 %	-1.3490	0.77	0.65
GO:0009735	response to cytokinin	1.010 %	-1.1509	0.82	0.56
GO:0009725	response to hormone	6.871 %	-2.2697	0.78	0.82
GO:0009723	response to ethylene	1.269 %	-3.3067	0.81	0.53
GO:0071495	cellular response to endogenous stimulus	3.988 %	-2.2532	0.82	0.64
GO:0055078	sodium ion homeostasis	0.030 %	-1.6120	0.90	0.15
GO:1901804	beta-glucoside metabolic process	0.009 %	-1.4888	0.90	0.16
GO:1901038	cyanidin 3-O-glucoside metabolic process	0.004 %	-1.4888	0.90	0.89
GO:0010467	gene expression	18.304 %	-2.0632	0.88	0.17
GO:0010529	negative regulation of transposition	0.009 %	-4.4790	0.80	0.18
GO:0010528	regulation of transposition	0.009 %	-4.4790	0.83	0.96
GO:0046486	glycerolipid metabolic process	0.764 %	-1.5620	0.84	0.21
GO:0009299	mRNA transcription	0.060 %	-1.1819	0.87	0.21
GO:0043086	negative regulation of catalytic activity	1.019 %	-1.9537	0.86	0.22
GO:0044092	negative regulation of molecular function	1.070 %	-1.3026	0.86	0.79
GO:0009061	anaerobic respiration	0.047 %	-1.2362	0.88	0.22
GO:0061647	histone H3-K9 modification	0.069 %	-3.4701	0.81	0.23
GO:1902275	regulation of chromatin organization	0.233 %	-2.9244	0.76	0.60
GO:0016570	histone modification	0.893 %	-1.6278	0.78	0.89
GO:0016569	covalent chromatin modification	1.273 %	-1.5308	0.79	0.77
GO:0051130	positive regulation of cellular component organization	0.388 %	-2.3843	0.71	0.72

GO:0051129	negative regulation of cellular component organization	0.211 %	-2.5617	0.74	0.68
GO:0033528	S-methylmethionine cycle	0.004 %	-1.7356	0.91	0.24
GO:0045017	glycerolipid biosynthetic process	0.445 %	-1.5089	0.80	0.65
GO:0006650	glycerophospholipid metabolic process	0.613 %	-1.1372	0.83	0.92
GO:0046463	acylglycerol biosynthetic process	0.117 %	-1.0257	0.81	0.99
GO:0046460	neutral lipid biosynthetic process	0.117 %	-1.0257	0.82	0.96
GO:0006659	phosphatidylserine biosynthetic process	0.009 %	-1.6120	0.83	0.65
GO:0006658	phosphatidylserine metabolic process	0.009 %	-1.6120	0.85	0.60
GO:0019432	triglyceride biosynthetic process	0.104 %	-1.0618	0.82	0.79
GO:0033477	S-methylmethionine metabolic process	0.004 %	-1.7356	0.91	0.58
GO:0019438	aromatic compound biosynthetic process	14.247 %	-5.8421	0.82	0.24
GO:0009059	macromolecule biosynthetic process	17.035 %	-1.7388	0.82	0.66
GO:0044249	cellular biosynthetic process	22.844 %	-1.7754	0.81	0.72
GO:0044271	cellular nitrogen compound biosynthetic process	17.018 %	-2.7758	0.80	0.61
GO:1901576	organic substance biosynthetic process	22.969 %	-2.2276	0.84	0.66
GO:0018130	heterocycle biosynthetic process	14.014 %	-4.8683	0.81	0.58
GO:1901362	organic cyclic compound biosynthetic process	14.769 %	-5.3327	0.83	0.55
GO:0006805	xenobiotic metabolic process	0.009 %	-1.4155	0.82	0.25
GO:0042178	xenobiotic catabolic process	0.004 %	-1.5164	0.82	0.96
GO:0071466	cellular response to xenobiotic stimulus	0.009 %	-1.4155	0.84	0.96
GO:0010496	intercellular transport	0.073 %	-1.1339	0.95	0.26
GO:0070483	detection of hypoxia	0.013 %	-1.6493	0.84	0.26
GO:0003032	detection of oxygen	0.013 %	-1.6493	0.85	0.78
GO:0034059	response to anoxia	0.030 %	-1.1454	0.86	0.69
GO:0050832	defense response to fungus	2.236 %	-2.2386	0.83	0.26
GO:0043207	response to external biotic stimulus	4.981 %	-1.6884	0.83	0.94
GO:0051707	response to other organism	4.968 %	-1.6884	0.83	0.85
GO:0009620	response to fungus	2.521 %	-1.9622	0.84	0.76
GO:0098542	defense response to other organism	4.044 %	-1.5038	0.82	0.92
GO:0023051	regulation of signaling	1.126 %	-2.0920	0.82	0.26
GO:0035556	intracellular signal transduction	3.112 %	-1.0175	0.63	0.55
GO:0010226	response to lithium ion	0.017 %	-1.3729	0.88	0.27
GO:0043543	protein acylation	0.345 %	-2.8754	0.86	0.28
GO:0010646	regulation of cell communication	1.139 %	-2.0801	0.80	0.29
GO:0048583	regulation of response to stimulus	2.521 %	-3.5641	0.72	0.29
GO:0051128	regulation of cellular component organization	1.467 %	-1.6322	0.77	0.30
GO:1901983	regulation of protein acetylation	0.056 %	-4.3934	0.72	0.31
GO:0032269	negative regulation of cellular protein metabolic process	0.673 %	-1.8285	0.66	0.89
GO:0032270	positive regulation of cellular protein metabolic process	0.699 %	-2.3843	0.64	0.92
GO:2000756	regulation of peptidyl-lysine acetylation	0.056 %	-4.3934	0.70	1.00
GO:1900111	positive regulation of histone H3-K9 dimethylation	0.017 %	-4.7774	0.64	0.89
GO:1900109	regulation of histone H3-K9 dimethylation	0.022 %	-4.7774	0.68	0.99
GO:2000757	negative regulation of peptidyl-lysine acetylation	0.039 %	-4.5709	0.68	1.00
GO:0031400	negative regulation of protein modification process	0.220 %	-3.3937	0.68	0.72
GO:0018394	peptidyl-lysine acetylation	0.263 %	-3.0427	0.80	0.96
GO:0031401	positive regulation of protein modification process	0.337 %	-3.1897	0.64	0.83
GO:0018393	internal peptidyl-lysine acetylation	0.263 %	-3.0427	0.80	1.00
GO:1901984	negative regulation of protein acetylation	0.039 %	-4.5709	0.69	0.95
GO:0031060	regulation of histone methylation	0.086 %	-4.1666	0.66	0.92
GO:0031057	negative regulation of histone modification	0.047 %	-4.5709	0.66	0.95
GO:0031058	positive regulation of histone modification	0.069 %	-4.5241	0.64	0.87
GO:0031056	regulation of histone modification	0.134 %	-3.6389	0.66	0.88
GO:0031062	positive regulation of histone methylation	0.047 %	-4.5241	0.63	0.94
GO:0051570	regulation of histone H3-K9 methylation	0.039 %	-4.4790	0.67	0.87
GO:0051567	histone H3-K9 methylation	0.065 %	-3.4701	0.78	0.96
GO:1905269	positive regulation of chromatin organization	0.086 %	-4.2749	0.71	0.81
GO:0016573	histone acetylation	0.263 %	-3.0427	0.75	0.86
GO:1905268	negative regulation of chromatin organization	0.065 %	-3.8614	0.73	0.84
GO:0051574	positive regulation of histone H3-K9 methylation	0.022 %	-4.6195	0.64	0.92
GO:0035065	regulation of histone acetylation	0.056 %	-4.3934	0.65	0.98
GO:0035067	negative regulation of histone acetylation	0.039 %	-4.5709	0.64	0.75
GO:0006473	protein acetylation	0.319 %	-2.9887	0.83	0.97
GO:0006475	internal protein amino acid acetylation	0.281 %	-3.0427	0.83	0.97
GO:0036123	histone H3-K9 dimethylation	0.022 %	-4.7774	0.79	1.00
GO:0019748	secondary metabolic process	1.938 %	-1.0882	0.87	0.31
GO:0009718	anthocyanin-containing compound biosynthetic process	0.121 %	-1.3682	0.84	0.32
GO:0031540	regulation of anthocyanin biosynthetic process	0.052 %	-1.0172	0.75	0.88
GO:0048518	positive regulation of biological process	4.398 %	-3.1028	0.81	0.32
GO:0048016	inositol phosphate-mediated signaling	0.009 %	-1.2823	0.77	0.32
GO:0009628	response to abiotic stimulus	7.946 %	-3.5385	0.84	0.32
GO:0010233	phloem transport	0.065 %	-1.9867	0.87	0.33

GO:0008213	protein alkylation	0.475 %	-2.2943	0.86	0.33
GO:0048519	negative regulation of biological process	3.716 %	-1.5038	0.81	0.33
GO:0015669	gas transport	0.009 %	-1.4629	0.94	0.33
GO:0015671	oxygen transport	0.009 %	-1.8438	0.94	0.33
GO:0048510	regulation of timing of transition from vegetative to reproductive phase	0.164 %	-5.6209	0.75	0.33
GO:0048509	regulation of meristem development	0.445 %	-3.0728	0.73	0.78
GO:0048507	meristem development	0.945 %	-2.1861	0.86	0.66
GO:0048506	regulation of timing of meristematic phase transition	0.164 %	-5.6209	0.75	0.95
GO:0051240	positive regulation of multicellular organismal process	0.401 %	-1.0834	0.68	0.78
GO:0048582	positive regulation of post-embryonic development	0.319 %	-1.2565	0.65	0.79
GO:0048580	regulation of post-embryonic development	1.303 %	-1.1494	0.66	0.68
GO:2000026	regulation of multicellular organismal development	1.765 %	-1.0205	0.67	0.92
GO:0040034	regulation of development, heterochronic	0.224 %	-5.0199	0.75	0.57
GO:0051094	positive regulation of developmental process	0.436 %	-1.0012	0.68	0.73
GO:0032957	inositol trisphosphate metabolic process	0.035 %	-1.0257	0.87	0.34
GO:0034551	mitochondrial respiratory chain complex III assembly	0.009 %	-1.5164	0.91	0.34
GO:0017062	respiratory chain complex III assembly	0.009 %	-1.5164	0.91	0.70
GO:0033108	mitochondrial respiratory chain complex assembly	0.117 %	-1.3343	0.89	0.61
GO:0010305	leaf vascular tissue pattern formation	0.117 %	-1.3026	0.85	0.35
GO:0048598	embryonic morphogenesis	0.125 %	-2.9977	0.84	0.35
GO:0009886	post-embryonic animal morphogenesis	0.526 %	-1.4385	0.81	0.64
GO:0090698	post-embryonic plant morphogenesis	0.716 %	-2.8963	0.85	0.56
GO:0009607	response to biotic stimulus	5.158 %	-1.8815	0.85	0.36
GO:0009314	response to radiation	2.892 %	-4.4364	0.81	0.37
GO:0009266	response to temperature stimulus	2.287 %	-1.9463	0.81	0.64
GO:0080167	response to karrikin	0.531 %	-2.0402	0.84	0.53
GO:0009605	response to external stimulus	6.366 %	-1.2079	0.84	0.38
GO:0010232	vascular transport	0.065 %	-1.9867	0.87	0.38
GO:0071323	cellular response to chitin	0.026 %	-1.3343	0.82	0.38
GO:0018193	peptidyl-amino acid modification	1.813 %	-1.4587	0.84	0.38
GO:0010378	temperature compensation of the circadian clock	0.004 %	-5.5160	0.80	0.39
GO:0042752	regulation of circadian rhythm	0.220 %	-1.8906	0.86	0.64
GO:0009719	response to endogenous stimulus	7.359 %	-2.1152	0.84	0.39
GO:0006863	purine nucleobase transport	0.095 %	-1.2664	0.93	0.39
GO:0015851	nucleobase transport	0.108 %	-1.2664	0.93	0.60
GO:0009647	skotomorphogenesis	0.026 %	-5.8764	0.75	0.41
GO:0009646	response to absence of light	0.142 %	-4.2066	0.83	0.69
GO:0072350	tricarboxylic acid metabolic process	0.259 %	-1.0257	0.86	0.41
GO:0050793	regulation of developmental process	2.577 %	-2.5900	0.71	0.42
GO:0044707	single-multicellular organism process	10.699 %	-4.8706	0.82	0.86
GO:0044767	single-organism developmental process	12.123 %	-4.0276	0.79	0.85
GO:0007275	multicellular organism development	10.423 %	-5.0168	0.78	0.82
GO:0009653	anatomical structure morphogenesis	3.500 %	-1.5566	0.85	0.51
GO:0048856	anatomical structure development	11.722 %	-5.1673	0.83	0.82
GO:0009791	post-embryonic development	5.848 %	-6.0828	0.79	0.61
GO:0048731	system development	7.225 %	-5.7964	0.78	0.74
GO:0019222	regulation of metabolic process	14.847 %	-4.0866	0.75	0.43
GO:0050794	regulation of cellular process	22.244 %	-1.2984	0.73	0.63
GO:0050789	regulation of biological process	24.333 %	-1.5227	0.79	0.54
GO:0009554	megasporogenesis	0.043 %	-1.2082	0.81	0.43
GO:0006979	response to oxidative stress	1.908 %	-1.4675	0.85	0.43
GO:0042221	response to chemical	12.434 %	-2.6902	0.83	0.43
GO:0048585	negative regulation of response to stimulus	0.699 %	-4.1409	0.69	0.44
GO:0048523	negative regulation of cellular process	2.719 %	-1.8321	0.74	0.74
GO:1901701	cellular response to oxygen-containing compound	2.788 %	-1.5481	0.75	0.82
GO:0097306	cellular response to alcohol	1.170 %	-1.6385	0.76	0.80
GO:0023057	negative regulation of signaling	0.345 %	-4.4523	0.76	0.78
GO:0051248	negative regulation of protein metabolic process	0.678 %	-1.8285	0.69	0.64
GO:0048584	positive regulation of response to stimulus	0.902 %	-1.2263	0.65	0.74
GO:0010648	negative regulation of cell communication	0.345 %	-4.4523	0.77	0.78
GO:0032870	cellular response to hormone stimulus	3.871 %	-1.7452	0.73	0.79
GO:0071215	cellular response to abscisic acid stimulus	1.170 %	-1.6385	0.75	0.98
GO:0071396	cellular response to lipid	1.770 %	-1.3255	0.76	0.81
GO:0071310	cellular response to organic substance	4.601 %	-1.6664	0.74	0.88
GO:0031399	regulation of protein modification process	0.699 %	-1.5173	0.69	0.76
GO:0009755	hormone-mediated signaling pathway	3.617 %	-1.3931	0.57	0.94
GO:0009738	abscisic acid-activated signaling pathway	1.062 %	-2.0702	0.61	0.71
GO:0034052	positive regulation of plant-type hypersensitive response	0.026 %	-1.3343	0.67	0.54
GO:0009787	regulation of abscisic acid-activated signaling pathway	0.350 %	-2.7771	0.62	0.93
GO:0009788	negative regulation of abscisic acid-activated signaling pathway	0.160 %	-4.0178	0.62	0.93
GO:0009968	negative regulation of signal transduction	0.332 %	-4.4937	0.64	0.67
GO:0009966	regulation of signal transduction	1.109 %	-2.1162	0.63	0.87

GO:0009938	negative regulation of gibberellic acid mediated signaling pathway	0.039 %	-1.1120	0.65	0.83
GO:1901419	regulation of response to alcohol	0.350 %	-2.7771	0.69	0.68
GO:1901420	negative regulation of response to alcohol	0.160 %	-4.0178	0.68	0.81
GO:0097033	mitochondrial respiratory chain complex III biogenesis	0.009 %	-1.5164	0.95	0.46
GO:0009746	response to hexose	0.220 %	-2.4458	0.83	0.46
GO:0009749	response to glucose	0.194 %	-1.8711	0.84	0.98
GO:0034284	response to monosaccharide	0.237 %	-2.3519	0.83	0.86
GO:0071368	cellular response to cytokinin stimulus	0.337 %	-1.4459	0.79	0.46
GO:0033554	cellular response to stress	3.772 %	-1.4872	0.78	0.47
GO:1901700	response to oxygen-containing compound	6.504 %	-2.0670	0.80	0.64
GO:0070887	cellular response to chemical stimulus	5.287 %	-2.2180	0.76	0.58
GO:0010033	response to organic substance	8.580 %	-1.7015	0.79	0.70
GO:0001101	response to acid chemical	5.011 %	-1.5388	0.80	0.62
GO:0006952	defense response	6.090 %	-1.8185	0.83	0.55
GO:0071219	cellular response to molecule of bacterial origin	0.030 %	-1.2510	0.83	0.48
GO:0009743	response to carbohydrate	0.518 %	-2.6258	0.83	0.49
GO:0006950	response to stress	14.156 %	-1.6385	0.83	0.49
GO:0071216	cellular response to biotic stimulus	0.052 %	-1.1819	0.85	0.49
GO:0051716	cellular response to stimulus	12.637 %	-1.4322	0.79	0.50

Biological Process (344) Tag Clouds

Scatterplot & Table Interactive Graph TreeMap

GROUP 38

Tip: your resulting list of GO terms seems to be quite long. If you want to reduce it further, press the Back button in your browser and choose a different setting for the "allowed similarity" parameter.

Hide/show dispensable GO terms

Export results to text table (CSV)

Make R script for plotting

term ID	description	frequency	pin?	log_{10} p-value	uniqueness	dispensability
GO:0002376	immune system process	1.532 %		-2.7772	0.99	0.00
GO:0009646	response to absence of light	0.142 %		-6.9447	0.89	0.00
GO:0010114	response to red light	0.259 %		-1.4689	0.89	0.80
GO:0009642	response to light intensity	0.587 %		-3.0294	0.88	0.60
GO:0009637	response to blue light	0.341 %		-1.8325	0.88	0.64
GO:0009639	response to red or far red light	0.872 %		-2.2704	0.87	0.83
GO:0009640	photomorphogenesis	0.311 %		-1.9021	0.71	0.64
GO:0009647	skotomorphogenesis	0.026 %		-5.7627	0.75	0.69
GO:0009416	response to light stimulus	2.771 %		-2.7210	0.86	0.79
GO:0009653	anatomical structure morphogenesis	3.500 %		-5.1467	0.82	0.00
GO:0044707	single-multicellular organism process	10.699 %		-2.4686	0.78	0.70
GO:0099402	plant organ development	3.763 %		-3.1300	0.74	0.57
GO:0010228	vegetative to reproductive phase transition of meristem	0.738 %		-1.1400	0.77	0.60
GO:0044767	single-organism developmental process	12.123 %		-3.3726	0.75	0.68
GO:0022622	root system development	1.895 %		-1.9920	0.76	0.67
GO:0007275	multicellular organism development	10.423 %		-2.0269	0.73	0.86

GO:0048367	shoot system development	3.599 %	-1.6177	0.74	0.74
GO:0048856	anatomical structure development	11.722 %	-2.9602	0.81	0.85
GO:0009791	post-embryonic development	5.848 %	-1.2912	0.75	0.76
GO:0048869	cellular developmental process	4.148 %	-3.0934	0.71	0.57
GO:0048731	system development	7.225 %	-2.4830	0.74	0.79
GO:0015837	amine transport	0.043 %	-3.6708	0.95	0.00
GO:0006868	glutamine transport	0.026 %	-1.7073	0.88	0.67
GO:0006865	amino acid transport	0.419 %	-1.3943	0.85	0.79
GO:0046942	carboxylic acid transport	0.682 %	-1.0137	0.85	0.95
GO:0010585	glutamine secretion	0.004 %	-1.7073	0.84	0.77
GO:0032890	regulation of organic acid transport	0.030 %	-3.6708	0.81	0.63
GO:0032973	amino acid export	0.082 %	-2.6697	0.87	0.72
GO:0051955	regulation of amino acid transport	0.030 %	-3.6708	0.80	1.00
GO:0051952	regulation of amine transport	0.030 %	-3.6708	0.86	0.96
GO:0015743	malate transport	0.086 %	-1.0638	0.87	0.79
GO:0015804	neutral amino acid transport	0.086 %	-1.0538	0.87	0.79
GO:0080143	regulation of amino acid export	0.030 %	-3.6708	0.80	0.52
GO:0023052	signaling	8.908 %	-1.4305	0.99	0.00
GO:0032501	multicellular organismal process	11.360 %	-2.3479	0.99	0.00
GO:0032502	developmental process	12.689 %	-3.1110	0.99	0.00
GO:0040007	growth	2.529 %	-1.8433	0.99	0.00
GO:0044419	interspecies interaction between organisms	0.423 %	-1.5710	0.98	0.00
GO:0051701	interaction with host	0.177 %	-1.0613	0.97	0.84
GO:0051817	modification of morphology or physiology of other organism involved in symbiotic interaction	0.129 %	-1.3123	0.88	0.82
GO:0035821	modification of morphology or physiology of other organism	1.385 %	-1.2477	0.86	0.67
GO:0019048	modulation by virus of host morphology or physiology	0.017 %	-1.1180	0.88	0.87
GO:0044003	modification by symbiont of host morphology or physiology	0.125 %	-1.3269	0.88	0.54
GO:0044550	secondary metabolite biosynthetic process	1.183 %	-6.8198	0.76	0.00
GO:0052314	phytoalexin metabolic process	0.052 %	-1.7357	0.79	0.71
GO:0052315	phytoalexin biosynthetic process	0.052 %	-1.7357	0.75	1.00
GO:0052317	camalexin metabolic process	0.047 %	-1.7896	0.74	0.99
GO:0052318	regulation of phytoalexin metabolic process	0.009 %	-3.4338	0.73	0.81
GO:0052319	regulation of phytoalexin biosynthetic process	0.009 %	-3.4338	0.72	1.00
GO:0052320	positive regulation of phytoalexin metabolic process	0.009 %	-3.4338	0.71	1.00
GO:0052322	positive regulation of phytoalexin biosynthetic process	0.009 %	-3.4338	0.70	1.00
GO:0010120	camalexin biosynthetic process	0.047 %	-1.7896	0.72	0.90
GO:1901182	regulation of camalexin biosynthetic process	0.009 %	-3.4338	0.70	1.00
GO:1901183	positive regulation of camalexin biosynthetic process	0.009 %	-3.4338	0.68	0.56
GO:0009691	cytokinin biosynthetic process	0.091 %	-1.0846	0.81	0.84
GO:0009698	phenylpropanoid metabolic process	0.505 %	-5.6683	0.75	0.80
GO:0009700	indole phytoalexin biosynthetic process	0.052 %	-1.7357	0.73	0.99
GO:0009699	phenylpropanoid biosynthetic process	0.337 %	-6.6438	0.72	0.76
GO:0010023	proanthocyanidin biosynthetic process	0.035 %	-1.0538	0.76	0.77
GO:0009683	indoleacetic acid metabolic process	0.078 %	-1.9607	0.75	0.67
GO:0033473	indoleacetic acid conjugate metabolic process	0.004 %	-1.9607	0.79	0.69
GO:0043455	regulation of secondary metabolic process	0.173 %	-1.0292	0.74	0.72
GO:0034754	cellular hormone metabolic process	0.263 %	-1.6011	0.85	0.75
GO:0009808	lignin metabolic process	0.285 %	-2.7854	0.75	0.91
GO:0009809	lignin biosynthetic process	0.181 %	-3.2826	0.74	0.88
GO:1900376	regulation of secondary metabolite biosynthetic process	0.078 %	-1.5254	0.72	0.80
GO:1900378	positive regulation of secondary metabolite biosynthetic process	0.017 %	-2.8563	0.72	0.79
GO:0046217	indole phytoalexin metabolic process	0.052 %	-1.7357	0.75	1.00
GO:0010345	suberin biosynthetic process	0.060 %	-5.4153	0.76	0.80
GO:0009404	toxin metabolic process	0.250 %	-1.4997	0.78	0.74
GO:0009403	toxin biosynthetic process	0.052 %	-1.7357	0.76	0.79
GO:0051176	positive regulation of sulfur metabolic process	0.035 %	-3.0515	0.81	0.81
GO:0042435	indole-containing compound biosynthetic process	0.237 %	-1.3943	0.84	0.84
GO:0046274	lignin catabolic process	0.078 %	-2.8678	0.76	0.82
GO:0042445	hormone metabolic process	0.695 %	-1.3906	0.85	0.82
GO:0046271	phenylpropanoid catabolic process	0.078 %	-2.7938	0.76	0.82
GO:0044699	single-organism process	41.709 %	-4.1231	1.00	0.00
GO:0050896	response to stimulus	25.943 %	-4.5709	1.00	0.00
GO:0051704	multi-organism process	3.362 %	-4.2453	0.99	0.00
GO:0071555	cell wall organization	2.430 %	-6.3807	0.88	0.00
GO:0042547	cell wall modification involved in multidimensional cell growth	0.017 %	-1.0073	0.83	0.58
GO:0042545	cell wall modification	0.561 %	-2.2990	0.89	0.80
GO:0071669	plant-type cell wall organization or biogenesis	1.152 %	-2.4400	0.93	0.82
GO:0009664	plant-type cell wall organization	0.652 %	-4.2257	0.89	0.81
GO:0016998	cell wall macromolecule catabolic process	0.112 %	-2.4507	0.89	0.64

GO:0071840	cellular component organization or biogenesis	14.454 %	-1.9846	1.00	0.00
GO:0042744	hydrogen peroxide catabolic process	0.380 %	-2.9333	0.90	0.05
GO:0019439	aromatic compound catabolic process	0.898 %	-1.6340	0.87	0.52
GO:0042743	hydrogen peroxide metabolic process	0.466 %	-2.1872	0.92	0.89
GO:0018027	peptidyl-lysine dimethylation	0.022 %	-1.2884	0.90	0.06
GO:0071554	cell wall organization or biogenesis	3.168 %	-5.0980	0.94	0.06
GO:0042762	regulation of sulfur metabolic process	0.091 %	-1.6935	0.83	0.07
GO:0009056	catabolic process	8.118 %	-1.6001	0.95	0.07
GO:0042430	indole-containing compound metabolic process	0.384 %	-1.5508	0.88	0.07
GO:0007154	cell communication	9.698 %	-1.1565	0.94	0.08
GO:0072593	reactive oxygen species metabolic process	0.734 %	-1.2133	0.93	0.08
GO:0008219	cell death	0.734 %	-1.6055	0.84	0.11
GO:0009410	response to xenobiotic stimulus	0.013 %	-2.0532	0.90	0.13
GO:0055088	lipid homeostasis	0.199 %	-1.0346	0.89	0.14
GO:0010268	brassinosteroid homeostasis	0.138 %	-1.1419	0.89	0.62
GO:0044763	single-organism cellular process	23.034 %	-3.0652	0.84	0.17
GO:0009299	mRNA transcription	0.060 %	-1.1544	0.87	0.18
GO:0051707	response to other organism	4.968 %	-4.7212	0.84	0.21
GO:0043207	response to external biotic stimulus	4.981 %	-4.7212	0.84	0.94
GO:0080027	response to herbivore	0.043 %	-2.2277	0.90	0.53
GO:0050832	defense response to fungus	2.236 %	-3.3018	0.83	0.84
GO:0071219	cellular response to molecule of bacterial origin	0.030 %	-1.2234	0.84	0.51
GO:0071216	cellular response to biotic stimulus	0.052 %	-1.1544	0.85	0.53
GO:0009620	response to fungus	2.521 %	-3.3974	0.85	0.85
GO:0009617	response to bacterium	1.748 %	-1.3604	0.86	0.81
GO:0098542	defense response to other organism	4.044 %	-3.8851	0.83	0.92
GO:0042742	defense response to bacterium	1.476 %	-1.0095	0.84	0.79
GO:0010921	regulation of phosphatase activity	0.035 %	-1.0253	0.84	0.23
GO:0043666	regulation of phosphoprotein phosphatase activity	0.022 %	-1.0441	0.81	0.93
GO:0080163	regulation of protein serine/threonine phosphatase activity	0.017 %	-1.1297	0.81	0.89
GO:0035445	borate transmembrane transport	0.017 %	-1.3875	0.95	0.23
GO:0098661	inorganic anion transmembrane transport	0.203 %	-1.3253	0.94	0.72
GO:0046713	borate transport	0.022 %	-1.3875	0.95	0.62
GO:1901983	regulation of protein acetylation	0.056 %	-1.1672	0.80	0.23
GO:2000756	regulation of peptidyl-lysine acetylation	0.056 %	-1.1672	0.79	1.00
GO:1900111	positive regulation of histone H3-K9 dimethylation	0.017 %	-1.2884	0.76	0.89
GO:1900109	regulation of histone H3-K9 dimethylation	0.022 %	-1.2884	0.77	0.99
GO:2000757	negative regulation of peptidyl-lysine acetylation	0.039 %	-1.2234	0.76	1.00
GO:1901984	negative regulation of protein acetylation	0.039 %	-1.2234	0.76	0.95
GO:0031060	regulation of histone methylation	0.086 %	-1.0954	0.76	0.92
GO:0031057	negative regulation of histone modification	0.047 %	-1.2234	0.73	0.95
GO:0031058	positive regulation of histone modification	0.069 %	-1.2086	0.74	0.87
GO:0031062	positive regulation of histone methylation	0.047 %	-1.2086	0.74	0.94
GO:0051570	regulation of histone H3-K9 methylation	0.039 %	-1.1943	0.77	0.87
GO:1905269	positive regulation of chromatin organization	0.086 %	-1.1297	0.79	0.81
GO:0051574	positive regulation of histone H3-K9 methylation	0.022 %	-1.2387	0.75	0.92
GO:0035065	regulation of histone acetylation	0.056 %	-1.1672	0.76	0.98
GO:0035067	negative regulation of histone acetylation	0.039 %	-1.2234	0.73	0.75
GO:0036123	histone H3-K9 dimethylation	0.022 %	-1.2884	0.86	1.00
GO:0046717	acid secretion	0.026 %	-1.7073	0.90	0.24
GO:0070988	demethylation	0.121 %	-1.0162	0.87	0.25
GO:0010497	plasmodesmata-mediated intercellular transport	0.056 %	-1.2234	0.93	0.25
GO:0010496	intercellular transport	0.073 %	-1.1065	0.93	0.26
GO:0045087	innate immune response	1.364 %	-3.1827	0.83	0.27
GO:0009626	plant-type hypersensitive response	0.341 %	-2.1872	0.69	0.84
GO:0009627	systemic acquired resistance	0.281 %	-3.0883	0.84	0.83
GO:0043067	regulation of programmed cell death	0.216 %	-1.0076	0.76	0.89
GO:0043068	positive regulation of programmed cell death	0.069 %	-2.1503	0.76	0.88
GO:0050778	positive regulation of immune response	0.255 %	-2.1915	0.74	0.99
GO:0050776	regulation of immune response	0.419 %	-1.7383	0.75	0.98
GO:0012501	programmed cell death	0.600 %	-1.5597	0.82	0.90
GO:0010942	positive regulation of cell death	0.104 %	-1.9437	0.75	0.77
GO:0002684	positive regulation of immune system process	0.255 %	-2.1915	0.81	0.90
GO:0002682	regulation of immune system process	0.453 %	-1.6369	0.84	0.85
GO:0010363	regulation of plant-type hypersensitive response	0.060 %	-2.1072	0.66	0.86
GO:0045088	regulation of innate immune response	0.397 %	-1.7926	0.74	0.94
GO:0045089	positive regulation of innate immune response	0.242 %	-2.2281	0.73	0.83
GO:0009814	defense response, incompatible interaction	0.725 %	-2.8019	0.82	0.91
GO:0034050	host programmed cell death induced by symbiont	0.345 %	-2.1131	0.83	0.90
GO:0034052	positive regulation of plant-type hypersensitive response	0.026 %	-2.9290	0.67	0.67
GO:0009866	induced systemic resistance, ethylene mediated signaling pathway	0.013 %	-1.7073	0.68	0.70
GO:0009871	jasmonic acid and ethylene-dependent systemic resistance, ethylene mediated signaling pathway	0.009 %	-2.3572	0.72	0.62

GO:0006955	immune response	1.398 %	-3.0907	0.84	0.96
GO:0048513	animal organ development	0.004 %	-1.9504	0.85	0.28
GO:0007043	cell-cell junction assembly	0.022 %	-6.0637	0.84	0.29
GO:0034329	cell junction assembly	0.026 %	-4.8095	0.84	0.94
GO:0045216	cell-cell junction organization	0.039 %	-4.2678	0.84	0.97
GO:1905328	plant septum development	0.009 %	-1.1297	0.89	0.30
GO:0070483	detection of hypoxia	0.013 %	-1.6211	0.88	0.30
GO:0003032	detection of oxygen	0.013 %	-1.6211	0.89	0.78
GO:0034059	response to anoxia	0.030 %	-1.1180	0.89	0.69
GO:0034330	cell junction organization	0.039 %	-4.2678	0.85	0.30
GO:0032870	cellular response to hormone stimulus	3.871 %	-2.5437	0.77	0.32
GO:0044700	single organism signaling	8.899 %	-1.4317	0.85	0.91
GO:0033993	response to lipid	3.267 %	-1.1028	0.84	0.67
GO:0071732	cellular response to nitric oxide	0.086 %	-1.7271	0.82	0.54
GO:0071731	response to nitric oxide	0.091 %	-1.6459	0.87	0.71
GO:0071365	cellular response to auxin stimulus	0.932 %	-1.4040	0.80	0.85
GO:0070887	cellular response to chemical stimulus	5.287 %	-2.9974	0.79	0.59
GO:0071369	cellular response to ethylene stimulus	0.906 %	-1.9874	0.79	0.78
GO:1902170	cellular response to reactive nitrogen species	0.104 %	-1.4451	0.84	0.77
GO:0071310	cellular response to organic substance	4.601 %	-2.4553	0.77	0.88
GO:0007165	signal transduction	8.731 %	-1.6218	0.63	0.67
GO:0010033	response to organic substance	8.580 %	-2.1529	0.83	0.68
GO:0009755	hormone-mediated signaling pathway	3.617 %	-2.2159	0.62	0.94
GO:0009739	response to gibberellin	0.626 %	-1.2550	0.85	0.60
GO:0009734	auxin-activated signaling pathway	0.868 %	-1.6455	0.67	0.77
GO:0009733	response to auxin	1.709 %	-2.1249	0.84	0.68
GO:0009725	response to hormone	6.871 %	-3.0451	0.82	0.82
GO:1990267	response to transition metal nanoparticle	2.017 %	-1.3417	0.86	0.51
GO:0009723	response to ethylene	1.269 %	-1.8731	0.85	0.65
GO:0034614	cellular response to reactive oxygen species	0.207 %	-1.1093	0.81	0.81
GO:0071495	cellular response to endogenous stimulus	3.988 %	-2.4500	0.85	0.75
GO:0019748	secondary metabolic process	1.938 %	-5.1414	0.84	0.33
GO:0019438	aromatic compound biosynthetic process	14.247 %	-4.7877	0.82	0.33
GO:0032774	RNA biosynthetic process	11.813 %	-1.7592	0.79	0.82
GO:0031323	regulation of cellular metabolic process	13.906 %	-1.2118	0.74	0.80
GO:0031326	regulation of cellular biosynthetic process	12.339 %	-1.2568	0.70	0.85
GO:0051252	regulation of RNA metabolic process	11.463 %	-1.5284	0.70	0.87
GO:2001141	regulation of RNA biosynthetic process	11.282 %	-1.5981	0.68	0.88
GO:0019219	regulation of nucleobase-containing compound metabolic process	11.700 %	-1.2836	0.71	0.84
GO:0044249	cellular biosynthetic process	22.844 %	-1.3110	0.83	0.63
GO:0006355	regulation of transcription, DNA-templated	11.234 %	-1.5981	0.68	0.55
GO:0006351	transcription, DNA-templated	11.709 %	-1.7592	0.79	0.87
GO:1901576	organic substance biosynthetic process	22.969 %	-1.0764	0.86	0.72
GO:0018130	heterocycle biosynthetic process	14.014 %	-1.5404	0.83	0.58
GO:1903506	regulation of nucleic acid-templated transcription	11.282 %	-1.5981	0.68	0.89
GO:0097659	nucleic acid-templated transcription	11.761 %	-1.7592	0.79	0.88
GO:0009889	regulation of biosynthetic process	12.408 %	-1.2200	0.74	0.78
GO:1901362	organic cyclic compound biosynthetic process	14.769 %	-4.6225	0.85	0.55
GO:0034654	nucleobase-containing compound biosynthetic process	13.056 %	-1.1564	0.80	0.80
GO:0051171	regulation of nitrogen compound metabolic process	12.430 %	-1.7565	0.76	0.76
GO:0009607	response to biotic stimulus	5.158 %	-7.2067	0.88	0.33
GO:0033310	chlorophyll a catabolic process	0.004 %	-2.3572	0.83	0.34
GO:0033304	chlorophyll a metabolic process	0.004 %	-2.3572	0.91	0.56
GO:0046688	response to copper ion	0.069 %	-2.5375	0.89	0.35
GO:0006521	regulation of cellular amino acid metabolic process	0.060 %	-1.1297	0.75	0.35
GO:0010160	formation of animal organ boundary	0.055 %	-2.2780	0.77	0.35
GO:0080110	sporopollenin biosynthetic process	0.030 %	-1.2234	0.68	0.62
GO:0048504	regulation of timing of animal organ formation	0.004 %	-1.3875	0.75	1.15
GO:1905393	plant organ formation	0.427 %	-1.5465	0.81	0.75
GO:0048859	formation of anatomical boundary	0.069 %	-1.4872	0.80	0.76
GO:0003156	regulation of animal organ formation	0.004 %	-1.0846	0.75	1.15
GO:0048645	animal organ formation	0.004 %	-1.7712	0.81	1.15
GO:0009887	animal organ morphogenesis	0.004 %	-2.4998	0.81	1.15
GO:0006949	syncytium formation	0.043 %	-2.1072	0.76	0.64
GO:0009605	response to external stimulus	6.366 %	-3.9215	0.87	0.35
GO:0001708	cell fate specification	0.134 %	-1.6693	0.77	0.36
GO:0010158	abaxial cell fate specification	0.030 %	-1.2387	0.79	0.77
GO:0045165	cell fate commitment	0.341 %	-1.3998	0.75	0.61
GO:0090627	plant epidermal cell differentiation	0.621 %	-1.6432	0.74	0.56
GO:0043693	monoterpene biosynthetic process	0.017 %	-3.4338	0.80	0.36

GO:0010686	tetracyclic triterpenoid biosynthetic process	0.004 %	-1.1672	0.80	0.52
GO:0010685	tetracyclic triterpenoid metabolic process	0.004 %	-1.1672	0.83	0.79
GO:0033383	geranyl diphosphate metabolic process	0.056 %	-2.6980	0.81	0.59
GO:0043692	monoterpene metabolic process	0.017 %	-3.4338	0.82	0.85
GO:0019745	pentacyclic triterpenoid biosynthetic process	0.004 %	-1.1672	0.80	0.81
GO:0016099	monoterpenoid biosynthetic process	0.004 %	-2.9679	0.82	0.52
GO:0016098	monoterpenoid metabolic process	0.004 %	-2.9679	0.84	0.51
GO:0016106	sesquiterpenoid biosynthetic process	0.138 %	-1.1544	0.77	0.84
GO:0016114	terpenoid biosynthetic process	0.591 %	-1.3599	0.74	0.78
GO:0006714	sesquiterpenoid metabolic process	0.181 %	-1.0538	0.80	0.86
GO:0006721	terpenoid metabolic process	0.699 %	-1.1593	0.77	0.94
GO:0042214	terpene metabolic process	0.065 %	-1.9324	0.81	0.64
GO:0008299	isoprenoid biosynthetic process	0.738 %	-1.1290	0.74	0.96
GO:0046246	terpene biosynthetic process	0.043 %	-2.2954	0.78	0.89
GO:0009719	response to endogenous stimulus	7.359 %	-2.4132	0.87	0.37
GO:0048284	organelle fusion	0.496 %	-1.2010	0.91	0.39
GO:0009968	negative regulation of signal transduction	0.332 %	-2.4069	0.65	0.40
GO:0048523	negative regulation of cellular process	2.719 %	-1.1643	0.77	0.77
GO:0023057	negative regulation of signaling	0.345 %	-2.3806	0.76	0.78
GO:1902679	negative regulation of RNA biosynthetic process	1.057 %	-1.6297	0.70	0.99
GO:0031327	negative regulation of cellular biosynthetic process	1.429 %	-1.2225	0.71	0.98
GO:0031325	positive regulation of cellular metabolic process	2.741 %	-1.1449	0.75	0.64
GO:0048584	positive regulation of response to stimulus	0.902 %	-1.1435	0.75	0.74
GO:0051253	negative regulation of RNA metabolic process	1.062 %	-1.6209	0.72	0.96
GO:0048585	negative regulation of response to stimulus	0.699 %	-1.2316	0.73	0.67
GO:0045934	negative regulation of nucleobase-containing compound metabolic process	1.139 %	-1.4990	0.72	0.92
GO:0010648	negative regulation of cell communication	0.345 %	-2.3806	0.79	0.78
GO:0010105	negative regulation of ethylene-activated signaling pathway	0.073 %	-1.2546	0.66	0.87
GO:0045892	negative regulation of transcription, DNA-templated	0.958 %	-1.6297	0.70	0.68
GO:0010629	negative regulation of gene expression	1.601 %	-1.0162	0.75	0.88
GO:0071370	cellular response to gibberellin stimulus	0.354 %	-1.1165	0.80	0.89
GO:0070298	negative regulation of phosphorelay signal transduction system	0.073 %	-1.2546	0.69	0.97
GO:0010558	negative regulation of macromolecule biosynthetic process	1.355 %	-1.3099	0.73	0.96
GO:0031349	positive regulation of defense response	0.341 %	-2.1646	0.74	0.63
GO:0009740	gibberellic acid mediated signaling pathway	0.341 %	-1.2521	0.69	0.88
GO:0010476	gibberellin mediated signaling pathway	0.345 %	-1.1690	0.69	0.99
GO:0010930	negative regulation of auxin mediated signaling pathway	0.013 %	-2.3572	0.70	0.77
GO:2000113	negative regulation of cellular macromolecule biosynthetic process	1.256 %	-1.3099	0.71	0.94
GO:1903507	negative regulation of nucleic acid-templated transcription	1.057 %	-1.6297	0.70	0.98
GO:0009893	positive regulation of metabolic process	2.892 %	-1.0162	0.78	0.84
GO:0009890	negative regulation of biosynthetic process	1.450 %	-1.2056	0.74	0.82
GO:1902532	negative regulation of intracellular signal transduction	0.091 %	-1.2546	0.68	0.89
GO:0080135	regulation of cellular response to stress	0.250 %	-1.4335	0.74	0.73
GO:0009937	regulation of gibberellic acid mediated signaling pathway	0.073 %	-1.6306	0.69	0.69
GO:0009938	negative regulation of gibberellic acid mediated signaling pathway	0.039 %	-2.4723	0.67	0.83
GO:0051172	negative regulation of nitrogen compound metabolic process	1.433 %	-1.2056	0.76	0.86
GO:0048440	carpel development	0.268 %	-4.4441	0.76	0.41
GO:0048528	post-embryonic root development	0.501 %	-1.2810	0.75	0.93
GO:0048527	lateral root development	0.457 %	-1.4852	0.75	0.92
GO:0090567	reproductive shoot system development	2.110 %	-2.1152	0.74	0.72
GO:0080060	integument development	0.039 %	-1.0846	0.79	0.83
GO:0016049	cell growth	1.519 %	-2.3898	0.82	0.83
GO:0048589	developmental growth	1.623 %	-2.0077	0.78	0.86
GO:0010102	lateral root morphogenesis	0.229 %	-2.1691	0.74	0.86
GO:0010101	post-embryonic root morphogenesis	0.233 %	-2.1691	0.75	0.90
GO:0010054	trichoblast differentiation	0.427 %	-2.0402	0.69	0.82
GO:0010053	root epidermal cell differentiation	0.488 %	-1.6960	0.68	0.94
GO:0060560	developmental growth involved in morphogenesis	1.157 %	-2.6258	0.75	0.75
GO:0048462	carpel formation	0.026 %	-1.3657	0.76	0.81
GO:0048467	gynoecium development	0.315 %	-4.7464	0.76	0.85
GO:0048466	androecium development	0.345 %	-1.8291	0.76	0.87
GO:0000902	cell morphogenesis	1.467 %	-2.6703	0.66	0.71
GO:0048438	floral whorl development	0.669 %	-3.5937	0.76	0.70
GO:0000904	cell morphogenesis involved in differentiation	0.880 %	-1.3416	0.67	0.91
GO:0048437	floral organ development	0.872 %	-2.5485	0.75	0.78
GO:0048444	floral organ morphogenesis	0.181 %	-1.1690	0.74	0.88
GO:0048443	stamen development	0.345 %	-1.8291	0.75	0.96
GO:0048446	petal morphogenesis	0.026 %	-1.1297	0.77	0.72

GO:0048445	carpel morphogenesis	0.035 %	-1.0954	0.76	0.83
GO:0010500	transmitting tissue development	0.017 %	-1.3064	0.81	0.79
GO:0010015	root morphogenesis	0.967 %	-2.8064	0.73	0.82
GO:0032989	cellular component morphogenesis	1.618 %	-2.8736	0.66	0.78
GO:0048766	root hair initiation	0.047 %	-1.1943	0.71	0.63
GO:1905392	plant organ morphogenesis	1.485 %	-2.6223	0.73	0.78
GO:0048827	phyllome development	1.921 %	-2.0290	0.74	0.82
GO:0030154	cell differentiation	3.332 %	-1.6520	0.70	0.81
GO:0009826	unidimensional cell growth	1.010 %	-2.4601	0.66	0.92
GO:0048364	root development	1.886 %	-2.0097	0.74	0.90
GO:0009908	flower development	2.059 %	-2.2588	0.73	0.88
GO:0048646	anatomical structure formation involved in morphogenesis	0.755 %	-2.7745	0.81	0.62
GO:0010311	lateral root formation	0.168 %	-1.0387	0.74	0.97
GO:0048653	anther development	0.224 %	-2.5061	0.76	0.83
GO:0009886	post-embryonic animal morphogenesis	0.526 %	-1.4105	0.76	0.64
GO:0090697	post-embryonic plant organ morphogenesis	0.419 %	-2.9251	0.74	0.55
GO:0090698	post-embryonic plant morphogenesis	0.716 %	-2.0920	0.81	0.66
GO:0090696	post-embryonic plant organ development	0.686 %	-2.0026	0.76	0.60
GO:0080126	ovary septum development	0.004 %	-1.7073	0.82	0.72
GO:0009561	megagametogenesis	0.281 %	-1.5324	0.80	0.41
GO:0010197	polar nucleus fusion	0.117 %	-1.2390	0.73	0.81
GO:0009559	embryo sac central cell differentiation	0.121 %	-1.2218	0.76	0.87
GO:0009553	embryo sac development	0.565 %	-1.0145	0.79	0.73
GO:0000741	karyogamy	0.117 %	-1.2176	0.91	0.88
GO:0015849	organic acid transport	0.682 %	-1.3943	0.87	0.43
GO:0006950	response to stress	14.156 %	-1.9677	0.86	0.44
GO:0006805	xenobiotic metabolic process	0.009 %	-1.3875	0.84	0.44
GO:0042178	xenobiotic catabolic process	0.004 %	-1.4883	0.83	0.96
GO:0071466	cellular response to xenobiotic stimulus	0.009 %	-1.3875	0.86	0.96
GO:0009628	response to abiotic stimulus	7.946 %	-1.8253	0.87	0.45
GO:1901361	organic cyclic compound catabolic process	0.937 %	-1.5002	0.90	0.45
GO:0044712	single-organism catabolic process	2.676 %	-1.0605	0.81	0.56
GO:0044248	cellular catabolic process	5.939 %	-1.3530	0.88	0.72
GO:0006997	nucleus organization	0.255 %	-1.0987	0.91	0.46
GO:0009623	response to parasitic fungus	0.009 %	-1.5495	0.91	0.46
GO:0009314	response to radiation	2.892 %	-2.4608	0.87	0.46
GO:0000160	phosphorelay signal transduction system	1.049 %	-1.5360	0.70	0.46
GO:0010529	negative regulation of transposition	0.009 %	-1.1943	0.78	0.47
GO:0010528	regulation of transposition	0.009 %	-1.1943	0.83	0.96
GO:0044711	single-organism biosynthetic process	7.549 %	-1.2430	0.78	0.47
GO:0006952	defense response	6.090 %	-4.4870	0.86	0.47
GO:0045229	external encapsulating structure organization	2.577 %	-6.2036	0.89	0.48
GO:0016043	cellular component organization	13.263 %	-2.4836	0.88	0.58
GO:0002215	defense response to nematode	0.013 %	-1.3064	0.89	0.48
GO:0044347	cell wall polysaccharide catabolic process	0.004 %	-3.2486	0.91	0.49
GO:0010817	regulation of hormone levels	1.109 %	-1.0181	0.87	0.49
GO:0042221	response to chemical	12.434 %	-1.6192	0.86	0.49
GO:0051782	negative regulation of cell division	0.022 %	-1.0846	0.78	0.50
GO:0051716	cellular response to stimulus	12.637 %	-1.0854	0.82	0.50

Biological Process (235) Tag Clouds

Scatterplot & Table Interactive Graph TreeMap

GROUP 39

Tip: your resulting list of GO terms seems to be quite long. If you want to reduce it further, press the Back button in your browser and choose a different setting for the "allowed similarity" parameter.

Hide/show dispensable GO terms

Export results to text table (CSV)

Make R script for plotting

term ID	description	frequency	pin?	$\log_{10}$ p-value	uniqueness	dispensability
GO:0000003	reproduction	6.655 %		-3.0039	1.00	0.00
GO:0009723	response to ethylene	1.269 %		-3.4854	0.86	0.00
GO:0071229	*cellular response to acid chemical*	*2.158 %*		*-1.3827*	*0.81*	*0.65*
GO:1901701	*cellular response to oxygen-containing compound*	*2.788 %*		*-1.0545*	*0.81*	*0.82*
GO:0010104	*regulation of ethylene-activated signaling pathway*	*0.095 %*		*-1.0179*	*0.73*	*0.98*
GO:0010105	*negative regulation of ethylene-activated signaling pathway*	*0.073 %*		*-1.3082*	*0.72*	*0.72*
GO:0032870	*cellular response to hormone stimulus*	*3.871 %*		*-1.2548*	*0.79*	*0.79*
GO:0071369	*cellular response to ethylene stimulus*	*0.906 %*		*-2.1769*	*0.81*	*0.55*
GO:0071370	*cellular response to gibberellin stimulus*	*0.354 %*		*-1.2120*	*0.82*	*0.89*
GO:0070298	*negative regulation of phosphorelay signal transduction system*	*0.073 %*		*-1.3082*	*0.76*	*0.97*
GO:0070297	*regulation of phosphorelay signal transduction system*	*0.095 %*		*-1.0179*	*0.77*	*0.87*
GO:0071396	*cellular response to lipid*	*1.770 %*		*-1.0006*	*0.81*	*0.81*
GO:0010033	*response to organic substance*	*8.580 %*		*-1.4399*	*0.85*	*0.54*
GO:0009740	*gibberellic acid mediated signaling pathway*	*0.341 %*		*-1.3501*	*0.72*	*0.62*
GO:0009739	*response to gibberellin*	*0.626 %*		*-1.4219*	*0.86*	*0.53*
GO:0009735	*response to cytokinin*	*1.010 %*		*-1.2138*	*0.87*	*0.56*

GO:0009725	response to hormone	6.871 %	-2.6283	0.84	0.82
GO:0010476	gibberellin mediated signaling pathway	0.345 %	-1.2655	0.72	0.99
GO:1902532	negative regulation of intracellular signal transduction	0.091 %	-1.3082	0.76	0.85
GO:0009873	ethylene-activated signaling pathway	0.811 %	-1.7397	0.70	0.91
GO:0071495	cellular response to endogenous stimulus	3.988 %	-1.2123	0.87	0.64
GO:0010191	mucilage metabolic process	0.142 %	-3.7422	0.95	0.00
GO:0032501	multicellular organismal process	11.360 %	-1.9235	0.99	0.00
GO:0032502	developmental process	12.689 %	-2.3434	0.99	0.00
GO:0042991	transcription factor import into nucleus	0.026 %	-5.3264	0.83	0.00
GO:1902593	single-organism nuclear import	0.242 %	-2.9398	0.81	0.99
GO:0044765	single-organism transport	5.287 %	-1.0954	0.80	0.71
GO:1902582	single-organism intracellular transport	0.790 %	-1.4233	0.80	0.87
GO:0033365	protein localization to organelle	1.148 %	-1.0764	0.90	0.84
GO:0044744	protein targeting to nucleus	0.246 %	-2.9398	0.89	0.98
GO:0034504	protein localization to nucleus	0.268 %	-2.8263	0.90	0.62
GO:0006606	protein import into nucleus	0.242 %	-2.9398	0.80	0.84
GO:0006605	protein targeting	1.006 %	-1.0289	0.89	0.84
GO:0072594	establishment of protein localization to organelle	0.924 %	-1.1514	0.89	0.83
GO:1902580	single-organism cellular localization	0.842 %	-1.3842	0.81	0.67
GO:0006913	nucleocytoplasmic transport	0.466 %	-1.8537	0.89	0.94
GO:0051170	nuclear import	0.246 %	-2.7529	0.90	0.89
GO:0051169	nuclear transport	0.470 %	-1.8537	0.91	0.54
GO:0044699	single-organism process	41.709 %	-2.3371	1.00	0.00
GO:0050896	response to stimulus	25.943 %	-1.0880	0.99	0.00
GO:0009812	flavonoid metabolic process	0.414 %	-1.9908	0.97	0.04
GO:0071554	cell wall organization or biogenesis	3.168 %	-1.9885	0.95	0.04
GO:0010411	xyloglucan metabolic process	0.259 %	-2.8592	0.88	0.07
GO:0016052	carbohydrate catabolic process	1.187 %	-1.1659	0.92	0.70
GO:0044275	cellular carbohydrate catabolic process	0.298 %	-1.2344	0.89	0.67
GO:0044264	cellular polysaccharide metabolic process	1.187 %	-1.2713	0.87	0.86
GO:0044036	cell wall macromolecule metabolic process	0.665 %	-1.2911	0.92	0.61
GO:0044042	glucan metabolic process	0.962 %	-1.6418	0.90	0.73
GO:0010383	cell wall polysaccharide metabolic process	0.518 %	-1.4914	0.87	0.93
GO:0006073	cellular glucan metabolic process	0.937 %	-1.6418	0.87	0.87
GO:0010410	hemicellulose metabolic process	0.423 %	-1.8808	0.88	0.92
GO:0005976	polysaccharide metabolic process	1.899 %	-1.2175	0.91	0.59
GO:0010214	seed coat development	0.168 %	-5.1620	0.70	0.07
GO:0044707	single-multicellular organism process	10.699 %	-2.3988	0.70	0.86
GO:0044702	single organism reproductive process	5.939 %	-2.5140	0.76	0.91
GO:0099402	plant organ development	3.763 %	-2.9276	0.65	0.77
GO:0003006	developmental process involved in reproduction	5.611 %	-2.8098	0.70	0.85
GO:0044767	single-organism developmental process	12.123 %	-2.5680	0.67	0.82
GO:0022622	root system development	1.895 %	-1.0972	0.67	0.69
GO:0010154	fruit development	2.443 %	-4.2577	0.64	0.86
GO:0048608	reproductive structure development	4.674 %	-4.0886	0.61	0.62
GO:0007275	multicellular organism development	10.423 %	-2.6342	0.64	0.82
GO:0048438	floral whorl development	0.669 %	-1.4316	0.66	0.72
GO:0032504	multicellular organism reproduction	0.613 %	-1.3314	0.78	0.62
GO:0048354	mucilage biosynthetic process involved in seed coat development	0.056 %	-1.3989	0.69	0.90
GO:0048825	cotyledon development	0.190 %	-3.5761	0.67	0.63
GO:0048827	phyllome development	1.921 %	-2.9336	0.66	0.69
GO:0048367	shoot system development	3.599 %	-1.6047	0.65	0.76
GO:0048366	leaf development	1.368 %	-1.9665	0.66	0.78
GO:0048364	root development	1.886 %	-1.1052	0.66	0.80
GO:0048359	mucilage metabolic process involved in seed coat development	0.086 %	-4.9309	0.69	0.55
GO:0048856	anatomical structure development	11.722 %	-2.5320	0.73	0.85
GO:0048316	seed development	2.305 %	-4.1009	0.63	0.85
GO:0009791	post-embryonic development	5.848 %	-3.7581	0.66	0.69
GO:0009793	embryo development ending in seed dormancy	1.584 %	-1.7824	0.63	0.92
GO:0022414	reproductive process	6.629 %	-3.0248	0.86	0.90
GO:0009965	leaf morphogenesis	0.380 %	-1.0935	0.67	0.79
GO:0061458	reproductive system development	4.674 %	-4.0886	0.65	0.80
GO:0048731	system development	7.225 %	-3.3035	0.65	0.74
GO:0031537	regulation of anthocyanin metabolic process	0.095 %	-3.0788	0.81	0.08
GO:0031539	positive regulation of anthocyanin metabolic process	0.022 %	-1.5150	0.81	0.87
GO:0031540	regulation of anthocyanin biosynthetic process	0.052 %	-2.3850	0.81	0.86
GO:0009718	anthocyanin-containing compound biosynthetic process	0.121 %	-2.4277	0.83	0.91
GO:0009813	flavonoid biosynthetic process	0.332 %	-1.6268	0.92	0.91

GO:0046148	pigment biosynthetic process	0.639 %	-1.5935	0.85	0.85
GO:0009962	regulation of flavonoid biosynthetic process	0.095 %	-1.6890	0.87	0.79
GO:0046283	anthocyanin-containing compound metabolic process	0.190 %	-2.7169	0.84	0.83
GO:0000374	Group III intron splicing	0.004 %	-2.2365	0.94	0.09
GO:0008283	cell proliferation	0.470 %	-2.6936	0.89	0.09
GO:0007015	actin filament organization	0.315 %	-2.1111	0.74	0.09
GO:0008154	actin polymerization or depolymerization	0.237 %	-1.4311	0.75	0.95
GO:0032970	regulation of actin filament-based process	0.194 %	-1.6075	0.76	0.90
GO:0051493	regulation of cytoskeleton organization	0.289 %	-1.4620	0.79	0.78
GO:0051017	actin filament bundle assembly	0.086 %	-1.0101	0.75	0.88
GO:0061572	actin filament bundle organization	0.086 %	-1.0101	0.76	0.88
GO:0030036	actin cytoskeleton organization	0.354 %	-1.8359	0.74	0.95
GO:0046209	nitric oxide metabolic process	0.030 %	-1.0598	0.94	0.10
GO:0006809	nitric oxide biosynthetic process	0.022 %	-1.1710	0.93	0.75
GO:0046777	protein autophosphorylation	0.604 %	-1.0006	0.93	0.12
GO:0006468	protein phosphorylation	5.334 %	-1.1548	0.92	0.62
GO:0042127	regulation of cell proliferation	0.293 %	-1.9230	0.84	0.17
GO:0008284	positive regulation of cell proliferation	0.129 %	-1.8221	0.83	0.85
GO:0006383	transcription from RNA polymerase III promoter	0.147 %	-2.1120	0.92	0.17
GO:0009682	induced systemic resistance	0.125 %	-1.4944	0.91	0.18
GO:0071219	cellular response to molecule of bacterial origin	0.030 %	-1.2769	0.87	0.54
GO:0030029	actin filament-based process	0.440 %	-1.7680	0.85	0.20
GO:0080167	response to karrikin	0.531 %	-2.8222	0.90	0.21
GO:0000160	phosphorelay signal transduction system	1.049 %	-1.6779	0.74	0.23
GO:0048533	sporocyte differentiation	0.004 %	-1.0425	0.79	0.23
GO:0042440	pigment metabolic process	0.785 %	-1.8852	0.87	0.23
GO:0006636	unsaturated fatty acid biosynthetic process	0.108 %	-1.0872	0.83	0.24
GO:0033559	unsaturated fatty acid metabolic process	0.112 %	-1.0872	0.84	0.65
GO:1905328	plant septum development	0.009 %	-1.1829	0.84	0.24
GO:0007049	cell cycle	2.525 %	-1.0917	0.83	0.25
GO:0006260	DNA replication	0.665 %	-1.6704	0.88	0.26
GO:0006310	DNA recombination	0.656 %	-1.0825	0.89	0.71
GO:0042023	DNA endoreduplication	0.160 %	-1.4412	0.77	0.62
GO:0006261	DNA-dependent DNA replication	0.419 %	-1.2776	0.88	0.84
GO:0051726	regulation of cell cycle	0.811 %	-1.3390	0.78	0.64
GO:0044786	cell cycle DNA replication	0.181 %	-1.4361	0.77	0.86
GO:0032876	negative regulation of DNA endoreduplication	0.030 %	-1.2923	0.76	0.87
GO:0032875	regulation of DNA endoreduplication	0.086 %	-1.0511	0.75	0.93
GO:0008156	negative regulation of DNA replication	0.039 %	-1.2478	0.83	0.88
GO:2000104	negative regulation of DNA-dependent DNA replication	0.039 %	-1.2478	0.83	0.98
GO:0022402	cell cycle process	1.713 %	-1.1456	0.80	0.81
GO:0000725	recombinational repair	0.350 %	-1.0341	0.80	0.88
GO:0000724	double-strand break repair via homologous recombination	0.345 %	-1.0341	0.80	0.67
GO:0006949	syncytium formation	0.043 %	-3.6510	0.73	0.27
GO:0048598	embryonic morphogenesis	0.125 %	-2.0829	0.72	0.56
GO:0048766	root hair initiation	0.047 %	-1.2478	0.65	0.52
GO:1905393	plant organ formation	0.427 %	-1.0916	0.76	0.73
GO:0048645	animal organ formation	0.004 %	-1.8752	0.76	0.54
GO:0048646	anatomical structure formation involved in morphogenesis	0.755 %	-3.1302	0.75	0.51
GO:0009887	animal organ morphogenesis	0.004 %	-1.5637	0.77	1.00
GO:0090698	post-embryonic plant morphogenesis	0.716 %	-1.7257	0.76	0.66
GO:0009719	response to endogenous stimulus	7.359 %	-2.0214	0.90	0.29
GO:0048513	animal organ development	0.004 %	-1.2047	0.79	0.30
GO:0043254	regulation of protein complex assembly	0.298 %	-1.6539	0.81	0.30
GO:0032271	regulation of protein polymerization	0.186 %	-1.6539	0.81	0.87
GO:0031334	positive regulation of protein complex assembly	0.190 %	-1.0024	0.80	0.92
GO:0051258	protein polymerization	0.263 %	-1.1197	0.86	0.73
GO:0033043	regulation of organelle organization	0.617 %	-1.0251	0.81	0.76
GO:0030041	actin filament polymerization	0.168 %	-1.6204	0.73	0.93
GO:0032535	regulation of cellular component size	0.319 %	-1.5007	0.77	0.30
GO:0032273	positive regulation of protein polymerization	0.108 %	-1.0024	0.80	0.95
GO:0030832	regulation of actin filament length	0.181 %	-1.6075	0.71	0.98
GO:0030833	regulation of actin filament polymerization	0.164 %	-1.6539	0.70	0.94
GO:0045010	actin nucleation	0.095 %	-1.0024	0.70	0.95
GO:0008064	regulation of actin polymerization or depolymerization	0.181 %	-1.6075	0.71	0.99
GO:0032956	regulation of actin cytoskeleton organization	0.194 %	-1.6075	0.72	0.99
GO:0030838	positive regulation of actin filament polymerization	0.095 %	-1.0024	0.70	0.98
GO:0051016	barbed-end actin filament capping	0.017 %	-1.2923	0.72	0.79
GO:0080006	internode patterning	0.009 %	-2.0155	0.76	0.31
GO:0010588	cotyledon vascular tissue pattern formation	0.060 %	-1.7724	0.67	0.58
GO:0010051	xylem and phloem pattern formation	0.259 %	-1.2049	0.73	0.76
GO:0010086	embryonic root morphogenesis	0.013 %	-1.2339	0.74	0.82

GO:0048439	flower morphogenesis	0.022 %	-1.6382	0.71	0.62
GO:0048826	cotyledon morphogenesis	0.073 %	-1.5111	0.67	0.91
GO:0048317	seed morphogenesis	0.026 %	-1.1950	0.71	0.63
GO:0010305	leaf vascular tissue pattern formation	0.117 %	-1.3501	0.75	0.84
GO:0071732	cellular response to nitric oxide	0.086 %	-1.8307	0.84	0.32
GO:0071731	response to nitric oxide	0.091 %	-1.7487	0.88	0.71
GO:0042631	cellular response to water deprivation	0.129 %	-1.4464	0.82	0.51
GO:1902170	cellular response to reactive nitrogen species	0.104 %	-1.5458	0.85	0.77
GO:0071462	cellular response to water stimulus	0.129 %	-1.4464	0.83	0.70
GO:0034599	cellular response to oxidative stress	0.453 %	-1.0224	0.83	0.62
GO:0034614	cellular response to reactive oxygen species	0.207 %	-1.2047	0.83	0.81
GO:1902578	single-organism localization	5.481 %	-1.0436	0.81	0.33
GO:0071836	nectar secretion	0.017 %	-1.9368	0.87	0.33
GO:1902047	polyamine transmembrane transport	0.022 %	-1.6756	0.87	0.33
GO:0002164	larval development	0.009 %	-1.4416	0.78	0.34
GO:0061062	regulation of nematode larval development	0.009 %	-1.4416	0.74	0.34
GO:0002119	nematode larval development	0.009 %	-1.4416	0.78	1.00
GO:0007143	female meiotic division	0.022 %	-1.8127	0.74	0.36
GO:0044843	cell cycle G1/S phase transition	0.069 %	-1.3422	0.84	0.51
GO:0010948	negative regulation of cell cycle process	0.117 %	-1.0511	0.79	0.57
GO:0000082	G1/S transition of mitotic cell cycle	0.052 %	-1.3422	0.85	0.83
GO:0048457	floral whorl morphogenesis	0.004 %	-2.0155	0.73	0.36
GO:0042493	response to drug	0.367 %	-1.0511	0.89	0.37
GO:0001944	vasculature development	0.108 %	-1.4197	0.74	0.37
GO:0072358	cardiovascular system development	0.108 %	-1.4197	0.74	1.00
GO:0072359	circulatory system development	0.108 %	-1.4197	0.74	0.37
GO:0009790	embryo development	1.683 %	-1.6589	0.70	0.39
GO:0001736	establishment of planar polarity	0.013 %	-1.2206	0.77	0.39
GO:0002009	morphogenesis of an epithelium	0.013 %	-1.1484	0.77	1.00
GO:0001738	morphogenesis of a polarized epithelium	0.013 %	-1.2206	0.77	1.00
GO:0009628	response to abiotic stimulus	7.946 %	-1.3969	0.90	0.39
GO:0071216	cellular response to biotic stimulus	0.052 %	-1.2076	0.89	0.39
GO:0048639	positive regulation of developmental growth	0.026 %	-1.3989	0.75	0.40
GO:0046620	regulation of organ growth	0.082 %	-1.0179	0.72	0.86
GO:0046622	positive regulation of organ growth	0.017 %	-1.3989	0.74	0.81
GO:0010197	polar nucleus fusion	0.117 %	-1.3367	0.66	0.41
GO:0009559	embryo sac central cell differentiation	0.121 %	-1.3193	0.72	0.87
GO:0000741	karyogamy	0.117 %	-1.3150	0.88	0.88
GO:0048729	tissue morphogenesis	0.013 %	-1.1484	0.77	0.41
GO:0009630	gravitropism	0.281 %	-1.4205	0.89	0.42
GO:0009959	negative gravitropism	0.035 %	-1.3422	0.91	0.83
GO:0009958	positive gravitropism	0.142 %	-1.2232	0.90	0.93
GO:0009313	oligosaccharide catabolic process	0.039 %	-1.8307	0.85	0.42
GO:0046352	disaccharide catabolic process	0.035 %	-1.0598	0.81	0.96
GO:0034484	raffinose catabolic process	0.004 %	-1.0511	0.86	0.85
GO:0005987	sucrose catabolic process	0.022 %	-1.1950	0.81	0.67
GO:0009629	response to gravity	0.311 %	-1.3734	0.91	0.42
GO:0006997	nucleus organization	0.255 %	-1.1939	0.88	0.44
GO:0009606	tropism	0.341 %	-1.2183	0.91	0.45
GO:0010229	inflorescence development	0.104 %	-1.1904	0.70	0.45
GO:0010068	protoderm histogenesis	0.004 %	-1.5150	0.75	0.45
GO:0010076	maintenance of floral meristem identity	0.026 %	-1.3791	0.77	0.50
GO:0010077	maintenance of inflorescence meristem identity	0.039 %	-1.2478	0.76	0.80
GO:0042547	cell wall modification involved in multidimensional cell growth	0.017 %	-1.0598	0.82	0.46
GO:0090066	regulation of anatomical structure size	0.319 %	-1.5007	0.92	0.46
GO:1900056	negative regulation of leaf senescence	0.043 %	-1.0179	0.68	0.46
GO:0048284	organelle fusion	0.496 %	-1.2981	0.87	0.46
GO:0009639	response to red or far red light	0.872 %	-1.9599	0.89	0.47
GO:0010224	response to UV-B	0.298 %	-1.0589	0.90	0.66
GO:0009411	response to UV	0.427 %	-1.2598	0.90	0.68
GO:0090351	seedling development	0.660 %	-2.1279	0.71	0.47
GO:0031507	heterochromatin assembly	0.035 %	-1.1829	0.88	0.48
GO:0070828	heterochromatin organization	0.043 %	-1.1829	0.90	0.51
GO:0009888	tissue development	2.326 %	-1.4281	0.76	0.48
GO:0009653	anatomical structure morphogenesis	3.500 %	-1.4260	0.75	0.53
GO:0044087	regulation of cellular component biogenesis	0.514 %	-1.1728	0.86	0.48
GO:0015846	polyamine transport	0.026 %	-1.6039	0.86	0.49
GO:0010201	response to continuous far red light stimulus by the high-irradiance response system	0.009 %	-1.0968	0.92	0.49
GO:0080126	ovary septum development	0.004 %	-1.7619	0.74	0.49
GO:0048462	carpel formation	0.026 %	-1.4197	0.69	0.65
GO:0048445	carpel morphogenesis	0.035 %	-1.1484	0.69	0.78
GO:0010500	transmitting tissue development	0.017 %	-1.3602	0.73	0.68
GO:0010480	microsporocyte differentiation	0.244 %	-1.0425	0.63	0.70
GO:0048653	anther development	0.224 %	-1.0116	0.67	0.93

GO:0080001	mucilage extrusion from seed coat	0.022 %	-3.6087	0.73	0.50
GO:0048609	*multicellular organismal reproductive process*	0.587 %	-1.4281	0.78	0.75
GO:0009845	*seed germination*	0.600 %	-2.2675	0.71	0.74
GO:0017038	protein import	0.531 %	-1.8316	0.82	0.50
GO:0034219	*carbohydrate transmembrane transport*	0.332 %	-1.5150	0.84	0.51

Biological Process (307) Tag Clouds

Scatterplot & Table Interactive Graph TreeMap

GROUP 40

Tip: your resulting list of GO terms seems to be quite long. If you want to reduce it further, press the Back button in your browser and choose a different setting for the "allowed similarity" parameter.

Hide/show dispensable GO terms Export results to text table (CSV) Make R script for plotting

term ID	description	frequency	pin?	$\log_{10}$ p-value	uniqueness	dispensability
GO:0009416	response to light stimulus	2.771 %		-4.3800	0.87	0.00
GO:0048573	*photoperiodism, flowering*	*0.388 %*		*-1.3278*	*0.67*	*0.75*
GO:0009641	*shade avoidance*	*0.069 %*		*-2.9312*	*0.90*	*0.63*
GO:0009642	*response to light intensity*	*0.587 %*		*-1.7808*	*0.89*	*0.79*
GO:0009643	*photosynthetic acclimation*	*0.035 %*		*-1.2558*	*0.91*	*0.60*
GO:0009637	*response to blue light*	*0.341 %*		*-1.8913*	*0.89*	*0.74*
GO:0009639	*response to red or far red light*	*0.872 %*		*-1.7900*	*0.88*	*0.83*
GO:0009640	*photomorphogenesis*	*0.311 %*		*-1.9615*	*0.69*	*0.74*
GO:0009645	*response to low light intensity stimulus*	*0.073 %*		*-1.0068*	*0.90*	*0.67*
GO:0009648	*photoperiodism*	*0.427 %*		*-1.1430*	*0.89*	*0.76*
GO:0009314	*response to radiation*	*2.892 %*		*-4.2886*	*0.88*	*0.65*
GO:0080167	*response to karrikin*	*0.531 %*		*-1.4359*	*0.90*	*0.53*
GO:0023052	signaling	8.908 %		-1.2745	0.99	0.00
GO:0032501	multicellular organismal process	11.360 %		-3.7587	0.99	0.00
GO:0032502	developmental process	12.689 %		-3.8470	0.99	0.00
GO:0040007	growth	2.529 %		-1.9151	0.99	0.00

GO:0044699	single-organism process	41.709 %	-5.0085	1.00	0.00
GO:0045893	positive regulation of transcription, DNA-templated	1.864 %	-2.2848	0.70	0.00
GO:0048522	positive regulation of cellular process	3.582 %	-1.0373	0.76	0.87
GO:0032774	RNA biosynthetic process	11.813 %	-2.8881	0.81	0.82
GO:0080090	regulation of primary metabolic process	13.681 %	-2.2757	0.75	0.82
GO:0031323	regulation of cellular metabolic process	13.906 %	-3.5019	0.73	0.78
GO:1902680	positive regulation of RNA biosynthetic process	1.869 %	-2.2848	0.70	0.97
GO:0031328	positive regulation of cellular biosynthetic process	2.102 %	-1.9736	0.71	0.97
GO:0031326	regulation of cellular biosynthetic process	12.339 %	-2.2060	0.70	0.87
GO:0031325	positive regulation of cellular metabolic process	2.741 %	-1.6951	0.73	0.95
GO:0051252	regulation of RNA metabolic process	11.463 %	-3.4545	0.70	0.87
GO:2001141	regulation of RNA biosynthetic process	11.282 %	-3.6053	0.68	0.88
GO:0019219	regulation of nucleobase-containing compound metabolic process	11.700 %	-3.2486	0.70	0.84
GO:0051254	positive regulation of RNA metabolic process	1.912 %	-2.2848	0.71	0.96
GO:0016070	RNA metabolic process	17.562 %	-1.1653	0.84	0.66
GO:0045935	positive regulation of nucleobase-containing compound metabolic process	2.007 %	-2.1932	0.71	0.96
GO:0010628	positive regulation of gene expression	2.050 %	-2.1216	0.74	0.90
GO:0006355	regulation of transcription, DNA-templated	11.234 %	-3.6053	0.68	0.63
GO:0006351	transcription, DNA-templated	11.709 %	-2.8881	0.81	0.87
GO:0010604	positive regulation of macromolecule metabolic process	2.672 %	-1.7485	0.74	0.92
GO:0010556	regulation of macromolecule biosynthetic process	12.076 %	-2.7802	0.71	0.86
GO:0010557	positive regulation of macromolecule biosynthetic process	2.007 %	-2.0771	0.72	0.95
GO:0010468	regulation of gene expression	12.706 %	-2.2889	0.74	0.80
GO:2000112	regulation of cellular macromolecule biosynthetic process	12.003 %	-2.7972	0.69	0.86
GO:0060255	regulation of macromolecule metabolic process	14.091 %	-1.9955	0.74	0.83
GO:1903508	positive regulation of nucleic acid-templated transcription	1.869 %	-2.2848	0.70	0.98
GO:1903506	regulation of nucleic acid-templated transcription	11.282 %	-3.6053	0.68	0.89
GO:0097659	nucleic acid-templated transcription	11.761 %	-2.8881	0.81	0.88
GO:0009893	positive regulation of metabolic process	2.892 %	-1.5264	0.76	0.79
GO:0009891	positive regulation of biosynthetic process	2.145 %	-1.9444	0.73	0.89
GO:0009889	regulation of biosynthetic process	12.408 %	-1.9421	0.72	0.80
GO:0051173	positive regulation of nitrogen compound metabolic process	2.119 %	-1.9989	0.74	0.88
GO:0034654	nucleobase-containing compound biosynthetic process	13.056 %	-2.0482	0.82	0.80
GO:0051171	regulation of nitrogen compound metabolic process	12.430 %	-2.8797	0.74	0.80
GO:0050896	response to stimulus	25.943 %	-5.3613	1.00	0.00
GO:0051704	multi-organism process	3.362 %	-1.2801	0.99	0.00
GO:0065007	biological regulation	28.554 %	-2.6835	1.00	0.00
GO:1905392	plant organ morphogenesis	1.485 %	-4.4799	0.71	0.00
GO:0044707	single-multicellular organism process	10.699 %	-4.2207	0.75	0.86
GO:0080110	sporopollenin biosynthetic process	0.030 %	-1.2404	0.69	0.72
GO:0099402	plant organ development	3.763 %	-3.2956	0.71	0.65
GO:0003006	developmental process involved in reproduction	5.611 %	-1.0803	0.79	0.58
GO:0044767	single-organism developmental process	12.123 %	-3.7182	0.72	0.82
GO:0022622	root system development	1.895 %	-1.2615	0.73	0.67
GO:0010102	lateral root morphogenesis	0.229 %	-1.4536	0.72	0.97
GO:0010101	post-embryonic root morphogenesis	0.233 %	-1.4536	0.72	0.88
GO:0048598	embryonic morphogenesis	0.125 %	-1.1289	0.77	0.59
GO:0050793	regulation of developmental process	2.577 %	-1.1420	0.70	0.51
GO:0007275	multicellular organism development	10.423 %	-4.3730	0.70	0.72
GO:0010094	specification of carpel identity	0.013 %	-1.6385	0.75	0.69
GO:0048462	carpel formation	0.026 %	-1.3830	0.75	0.93
GO:0048437	floral organ development	0.872 %	-1.0949	0.72	0.71
GO:0048444	floral organ morphogenesis	0.181 %	-1.1996	0.72	0.88
GO:0048448	stamen morphogenesis	0.047 %	-1.0240	0.74	0.87
GO:0048446	petal morphogenesis	0.026 %	-1.1467	0.75	0.83
GO:0048445	carpel morphogenesis	0.035 %	-1.1123	0.75	0.85
GO:0048451	petal formation	0.009 %	-1.3622	0.77	0.75
GO:0048449	floral organ formation	0.104 %	-1.5320	0.73	0.80
GO:0009653	anatomical structure morphogenesis	3.500 %	-3.0199	0.80	0.50
GO:0010015	root morphogenesis	0.967 %	-1.8182	0.71	0.82
GO:0010016	shoot system morphogenesis	0.708 %	-2.0578	0.73	0.71
GO:0048281	inflorescence morphogenesis	0.013 %	-1.2114	0.77	0.68
GO:1905393	plant organ formation	0.427 %	-3.8939	0.80	0.67
GO:0048826	cotyledon morphogenesis	0.073 %	-1.4427	0.74	0.77
GO:0048827	phyllome development	1.921 %	-2.1562	0.71	0.77
GO:0048367	shoot system development	3.599 %	-1.2828	0.72	0.74
GO:0048366	leaf development	1.368 %	-2.2374	0.72	0.78
GO:0048364	root development	1.886 %	-1.2708	0.71	0.90

GO:0048856	anatomical structure development	11.722 %	-3.6774	0.78	0.85
GO:0009791	post-embryonic development	5.848 %	-2.2970	0.72	0.78
GO:0048646	anatomical structure formation involved in morphogenesis	0.755 %	-2.8867	0.80	0.71
GO:0010311	lateral root formation	0.168 %	-1.8864	0.73	0.83
GO:0009888	tissue development	2.326 %	-1.8763	0.81	0.53
GO:0009886	post-embryonic animal morphogenesis	0.526 %	-1.4278	0.74	0.68
GO:0090697	post-embryonic plant organ morphogenesis	0.419 %	-2.2570	0.72	0.67
GO:0090698	post-embryonic plant morphogenesis	0.716 %	-3.5503	0.80	0.71
GO:0090696	post-embryonic plant organ development	0.686 %	-1.5109	0.74	0.69
GO:0009965	leaf morphogenesis	0.380 %	-2.2948	0.73	0.79
GO:0048229	gametophyte development	1.653 %	-1.5779	0.75	0.51
GO:0048731	system development	7.225 %	-2.3844	0.71	0.82
GO:0010166	wax metabolic process	0.082 %	-1.3389	0.97	0.05
GO:0042547	cell wall modification involved in multidimensional cell growth	0.017 %	-2.3475	0.85	0.08
GO:0042545	cell wall modification	0.561 %	-1.6654	0.93	0.52
GO:0016049	cell growth	1.519 %	-2.0577	0.82	0.56
GO:0045926	negative regulation of growth	0.121 %	-1.5844	0.84	0.66
GO:0045927	positive regulation of growth	0.104 %	-1.6410	0.82	0.65
GO:0030308	negative regulation of cell growth	0.043 %	-2.2299	0.76	0.60
GO:0071555	cell wall organization	2.430 %	-1.4672	0.92	0.80
GO:0009825	multidimensional cell growth	0.091 %	-1.3684	0.85	0.64
GO:0001558	regulation of cell growth	0.293 %	-1.3532	0.75	0.72
GO:0040008	regulation of growth	1.148 %	-1.5889	0.83	0.83
GO:0032973	amino acid export	0.082 %	-4.4030	0.80	0.09
GO:0006820	anion transport	1.524 %	-1.3016	0.89	0.54
GO:0006868	glutamine transport	0.026 %	-1.7248	0.82	0.71
GO:0006865	amino acid transport	0.419 %	-3.5872	0.79	0.79
GO:1905039	carboxylic acid transmembrane transport	0.505 %	-1.1235	0.78	0.96
GO:0043090	amino acid import	0.056 %	-3.0971	0.81	0.75
GO:0046942	carboxylic acid transport	0.682 %	-1.8469	0.78	0.95
GO:0010585	glutamine secretion	0.004 %	-1.7248	0.80	0.77
GO:0032890	regulation of organic acid transport	0.030 %	-1.6795	0.75	0.65
GO:1903825	organic acid transmembrane transport	0.505 %	-1.1235	0.79	0.96
GO:0051955	regulation of amino acid transport	0.030 %	-1.6795	0.74	1.00
GO:0051952	regulation of amine transport	0.030 %	-1.6795	0.80	0.96
GO:0003333	amino acid transmembrane transport	0.337 %	-1.1235	0.78	0.89
GO:0015827	tryptophan transport	0.004 %	-1.4522	0.84	0.64
GO:0015800	acidic amino acid transport	0.035 %	-1.1015	0.81	0.72
GO:0015804	neutral amino acid transport	0.086 %	-2.4433	0.80	0.79
GO:0015801	aromatic amino acid transport	0.017 %	-1.4522	0.82	0.69
GO:0015802	basic amino acid transport	0.035 %	-1.1588	0.81	0.72
GO:0015809	arginine transport	0.017 %	-1.1713	0.82	0.69
GO:0015810	aspartate transport	0.009 %	-1.4522	0.83	0.66
GO:0015837	amine transport	0.043 %	-1.6795	0.89	0.56
GO:0080143	regulation of amino acid export	0.030 %	-1.6795	0.74	0.72
GO:0015711	organic anion transport	0.945 %	-1.7813	0.86	0.81
GO:0015714	phosphoenolpyruvate transport	0.017 %	-1.2558	0.83	0.63
GO:0015979	photosynthesis	1.057 %	-1.1226	0.94	0.10
GO:0008283	cell proliferation	0.470 %	-1.7869	0.91	0.11
GO:0019748	secondary metabolic process	1.938 %	-3.1971	0.85	0.13
GO:0051100	negative regulation of binding	0.004 %	-1.6795	0.91	0.13
GO:0043392	negative regulation of DNA binding	0.004 %	-1.6795	0.91	0.84
GO:0051101	regulation of DNA binding	0.017 %	-1.5351	0.90	0.84
GO:0071291	cellular response to selenium ion	0.004 %	-2.1992	0.90	0.15
GO:0080144	amino acid homeostasis	0.060 %	-2.1992	0.91	0.16
GO:0030155	regulation of cell adhesion	0.009 %	-1.6385	0.91	0.16
GO:0051301	cell division	1.528 %	-1.2146	0.85	0.18
GO:1901334	lactone metabolic process	0.026 %	-1.2404	0.93	0.19
GO:0044763	single-organism cellular process	23.034 %	-1.3313	0.84	0.19
GO:0010025	wax biosynthetic process	0.078 %	-1.3993	0.94	0.19
GO:0002764	immune response-regulating signaling pathway	0.022 %	-1.1713	0.78	0.20
GO:0002218	activation of innate immune response	0.155 %	-1.1325	0.78	0.73
GO:0002253	activation of immune response	0.155 %	-1.1325	0.78	0.92
GO:0007043	cell-cell junction assembly	0.022 %	-1.7248	0.87	0.20
GO:0034329	cell junction assembly	0.026 %	-1.3425	0.87	0.94
GO:0045216	cell-cell junction organization	0.039 %	-1.1713	0.87	0.97
GO:0006083	acetate metabolic process	0.009 %	-1.3236	0.87	0.21
GO:0051302	regulation of cell division	0.199 %	-1.8814	0.79	0.23
GO:0009786	regulation of asymmetric cell division	0.017 %	-1.4781	0.82	0.55
GO:0008356	asymmetric cell division	0.091 %	-1.7264	0.88	0.62
GO:0065009	regulation of molecular function	2.443 %	-1.1944	0.87	0.23

GO:0042127	regulation of cell proliferation	0.293 %	-1.0281	0.81	0.24
GO:0007346	regulation of mitotic cell cycle	0.306 %	-1.7347	0.76	0.24
GO:0042023	DNA endoreduplication	0.160 %	-1.3735	0.79	0.59
GO:0006261	DNA-dependent DNA replication	0.419 %	-1.2115	0.88	0.84
GO:0045930	negative regulation of mitotic cell cycle	0.108 %	-1.1713	0.76	0.82
GO:0044786	cell cycle DNA replication	0.181 %	-1.3684	0.79	0.86
GO:0044839	cell cycle G2/M phase transition	0.091 %	-1.3425	0.85	0.87
GO:1902749	regulation of cell cycle G2/M phase transition	0.082 %	-1.3425	0.77	0.98
GO:1901987	regulation of cell cycle phase transition	0.164 %	-1.0068	0.76	0.95
GO:1901990	regulation of mitotic cell cycle phase transition	0.147 %	-1.0068	0.76	0.94
GO:0007135	meiosis II	0.073 %	-1.4278	0.82	0.55
GO:0010389	regulation of G2/M transition of mitotic cell cycle	0.078 %	-1.3425	0.77	0.74
GO:0000086	G2/M transition of mitotic cell cycle	0.086 %	-1.3425	0.85	0.99
GO:0009733	response to auxin	1.709 %	-3.3967	0.87	0.26
GO:0044700	single organism signaling	8.899 %	-1.2759	0.86	0.91
GO:1901700	response to oxygen-containing compound	6.504 %	-1.3553	0.86	0.70
GO:0032870	cellular response to hormone stimulus	3.871 %	-1.7083	0.81	0.94
GO:0033993	response to lipid	3.267 %	-1.5616	0.86	0.60
GO:0071365	cellular response to auxin stimulus	0.932 %	-1.4585	0.84	0.85
GO:0070887	cellular response to chemical stimulus	5.287 %	-1.9488	0.83	0.68
GO:0071310	cellular response to organic substance	4.601 %	-1.4492	0.81	0.87
GO:0007165	signal transduction	8.731 %	-1.2843	0.63	0.77
GO:0010033	response to organic substance	8.580 %	-3.0963	0.86	0.62
GO:0001101	response to acid chemical	5.011 %	-1.1604	0.87	0.67
GO:0009755	hormone-mediated signaling pathway	3.617 %	-1.9034	0.63	0.68
GO:0009751	response to salicylic acid	0.876 %	-1.0132	0.87	0.79
GO:0009741	response to brassinosteroid	0.410 %	-2.3879	0.88	0.63
GO:0009739	response to gibberellin	0.626 %	-2.5779	0.87	0.55
GO:0009734	auxin-activated signaling pathway	0.868 %	-1.7026	0.68	0.77
GO:0009735	response to cytokinin	1.010 %	-1.1254	0.87	0.58
GO:0009725	response to hormone	6.871 %	-3.7305	0.84	0.82
GO:0009788	negative regulation of abscisic acid-activated signaling pathway	0.160 %	-1.1047	0.70	0.57
GO:0071446	cellular response to salicylic acid stimulus	0.242 %	-1.5377	0.85	0.71
GO:0014070	response to organic cyclic compound	1.606 %	-2.2695	0.87	0.55
GO:1901420	negative regulation of response to alcohol	0.160 %	-1.1047	0.78	0.88
GO:0071495	cellular response to endogenous stimulus	3.988 %	-1.6657	0.88	0.74
GO:0009410	response to xenobiotic stimulus	0.013 %	-2.0870	0.92	0.28
GO:0006805	xenobiotic metabolic process	0.009 %	-1.4048	0.87	0.29
GO:0042178	xenobiotic catabolic process	0.004 %	-1.5056	0.87	0.96
GO:0071466	cellular response to xenobiotic stimulus	0.009 %	-1.4048	0.89	0.96
GO:0045229	external encapsulating structure organization	2.577 %	-1.6116	0.93	0.29
GO:0016043	cellular component organization	13.263 %	-1.1975	0.92	0.58
GO:0009698	phenylpropanoid metabolic process	0.505 %	-3.2872	0.80	0.30
GO:0044550	secondary metabolite biosynthetic process	1.183 %	-2.8904	0.80	0.80
GO:0009699	phenylpropanoid biosynthetic process	0.337 %	-3.0974	0.78	0.70
GO:0009808	lignin metabolic process	0.285 %	-2.0436	0.81	0.91
GO:0009809	lignin biosynthetic process	0.181 %	-1.5996	0.79	0.88
GO:0010345	suberin biosynthetic process	0.060 %	-2.8322	0.81	0.80
GO:0016143	S-glycoside metabolic process	0.514 %	-1.7101	0.83	0.30
GO:0016144	S-glycoside biosynthetic process	0.194 %	-1.2717	0.81	0.81
GO:0009759	indole glucosinolate biosynthetic process	0.026 %	-1.2717	0.81	0.74
GO:0042343	indole glucosinolate metabolic process	0.125 %	-1.7101	0.80	0.70
GO:0000103	sulfate assimilation	0.060 %	-1.0909	0.94	0.66
GO:0048518	positive regulation of biological process	4.398 %	-1.9254	0.84	0.30
GO:0051707	response to other organism	4.968 %	-1.8707	0.89	0.30
GO:0043207	response to external biotic stimulus	4.981 %	-1.8707	0.89	0.94
GO:0016045	detection of bacterium	0.013 %	-1.3236	0.92	0.95
GO:0009624	response to nematode	0.306 %	-1.4516	0.91	0.65
GO:0009595	detection of biotic stimulus	0.030 %	-1.1976	0.92	0.67
GO:0098543	detection of other organism	0.017 %	-1.3236	0.92	0.97
GO:0098581	detection of external biotic stimulus	0.030 %	-1.1976	0.92	0.51
GO:0034330	cell junction organization	0.039 %	-1.1713	0.88	0.30
GO:0034389	lipid particle organization	0.039 %	-1.2558	0.95	0.30
GO:0043467	regulation of generation of precursor metabolites and energy	0.117 %	-1.3735	0.83	0.32
GO:0042793	transcription from plastid promoter	0.039 %	-1.3425	0.89	0.32
GO:0010109	regulation of photosynthesis	0.160 %	-2.0092	0.82	0.33
GO:0042548	regulation of photosynthesis, light reaction	0.108 %	-1.4540	0.81	0.92
GO:0010205	photoinhibition	0.060 %	-1.6051	0.68	0.95
GO:0043155	negative regulation of photosynthesis, light reaction	0.060 %	-1.6051	0.81	0.97
GO:0009767	photosynthetic electron transport chain	0.224 %	-1.0341	0.82	0.79

GO:0019684	photosynthesis, light reaction	0.505 %	-1.0953	0.91	0.77
GO:1905156	negative regulation of photosynthesis	0.073 %	-1.5256	0.81	0.66
GO:0010269	response to selenium ion	0.009 %	-1.8995	0.92	0.33
GO:0019048	modulation by virus of host morphology or physiology	0.017 %	-1.1349	0.90	0.33
GO:0009299	mRNA transcription	0.060 %	-1.1713	0.89	0.33
GO:0048519	negative regulation of biological process	3.716 %	-1.0586	0.84	0.33
GO:0071705	nitrogen compound transport	1.515 %	-2.3228	0.91	0.34
GO:0006811	ion transport	4.174 %	-1.3352	0.90	0.52
GO:1902578	single-organism localization	5.481 %	-1.1993	0.82	0.60
GO:0007029	endoplasmic reticulum organization	0.073 %	-1.1349	0.95	0.34
GO:0048513	animal organ development	0.004 %	-1.1396	0.83	0.34
GO:0046417	chorismate metabolic process	0.056 %	-1.1588	0.85	0.35
GO:0009423	chorismate biosynthetic process	0.039 %	-1.2257	0.83	0.73
GO:0009719	response to endogenous stimulus	7.359 %	-2.9836	0.89	0.36
GO:0009956	radial pattern formation	0.078 %	-1.6193	0.79	0.36
GO:0003002	regionalization	0.617 %	-1.4143	0.76	0.79
GO:0060774	auxin mediated signaling pathway involved in phyllotactic patterning	0.009 %	-1.2558	0.68	0.79
GO:0060771	phyllotactic patterning	0.017 %	-1.1976	0.81	0.64
GO:0060772	leaf phyllotactic patterning	0.013 %	-1.2114	0.81	0.95
GO:0044347	cell wall polysaccharide catabolic process	0.004 %	-1.4781	0.93	0.36
GO:0000272	polysaccharide catabolic process	0.777 %	-1.2633	0.94	0.52
GO:0045490	pectin catabolic process	0.423 %	-1.3437	0.94	0.85
GO:0016998	cell wall macromolecule catabolic process	0.112 %	-1.0909	0.92	0.59
GO:0009607	response to biotic stimulus	5.158 %	-2.2621	0.90	0.36
GO:0046717	acid secretion	0.026 %	-1.7248	0.86	0.36
GO:0009605	response to external stimulus	6.366 %	-1.3445	0.90	0.37
GO:0019438	aromatic compound biosynthetic process	14.247 %	-4.6389	0.85	0.38
GO:0018130	heterocycle biosynthetic process	14.014 %	-2.0170	0.85	0.58
GO:1901362	organic cyclic compound biosynthetic process	14.769 %	-4.1233	0.86	0.55
GO:0009628	response to abiotic stimulus	7.946 %	-2.3985	0.89	0.39
GO:0006521	regulation of cellular amino acid metabolic process	0.060 %	-1.1467	0.77	0.40
GO:0051098	regulation of binding	0.039 %	-1.4522	0.90	0.40
GO:0044092	negative regulation of molecular function	1.070 %	-1.2833	0.87	0.58
GO:0040009	regulation of growth rate	0.013 %	-1.4278	0.88	0.41
GO:0046459	short-chain fatty acid metabolic process	0.013 %	-1.2558	0.86	0.41
GO:0010226	response to lithium ion	0.017 %	-1.3622	0.92	0.41
GO:0016106	sesquiterpenoid biosynthetic process	0.138 %	-1.1713	0.81	0.43
GO:1901601	strigolactone biosynthetic process	0.026 %	-1.2404	0.82	0.85
GO:1901600	strigolactone metabolic process	0.026 %	-1.2404	0.84	1.00
GO:0006714	sesquiterpenoid metabolic process	0.181 %	-1.0706	0.84	0.76
GO:1901336	lactone biosynthetic process	0.026 %	-1.2404	0.90	1.00
GO:0009553	embryo sac development	0.565 %	-2.3805	0.77	0.43
GO:0042221	response to chemical	12.434 %	-1.6715	0.89	0.43
GO:0009845	seed germination	0.600 %	-1.5148	0.75	0.43
GO:0010187	negative regulation of seed germination	0.073 %	-1.6410	0.69	0.81
GO:1900140	regulation of seedling development	0.315 %	-1.1453	0.67	0.92
GO:0010029	regulation of seed germination	0.298 %	-1.2253	0.67	0.91
GO:0090351	seedling development	0.660 %	-1.4072	0.75	0.53
GO:0001944	vasculature development	0.108 %	-1.3830	0.79	0.44
GO:0072358	cardiovascular system development	0.108 %	-1.3830	0.79	1.00
GO:0072359	circulatory system development	0.108 %	-1.3830	0.79	0.44
GO:0007389	pattern specification process	0.742 %	-1.2102	0.77	0.44
GO:0010229	inflorescence development	0.104 %	-1.1254	0.77	0.45
GO:0015849	organic acid transport	0.682 %	-3.5872	0.82	0.46
GO:0044765	single-organism transport	5.287 %	-1.2593	0.79	0.69
GO:0008643	carbohydrate transport	0.630 %	-1.3105	0.82	0.55
GO:2000123	positive regulation of stomatal complex development	0.013 %	-1.7248	0.70	0.46
GO:0048509	regulation of meristem development	0.445 %	-1.5763	0.72	0.51
GO:0048510	regulation of timing of transition from vegetative to reproductive phase	0.164 %	-1.2402	0.74	0.78
GO:0048507	meristem development	0.945 %	-1.1269	0.82	0.73
GO:0048506	regulation of timing of meristematic phase transition	0.164 %	-1.2402	0.74	0.95
GO:0051241	negative regulation of multicellular organismal process	0.445 %	-1.1364	0.70	0.54
GO:0051240	positive regulation of multicellular organismal process	0.401 %	-1.0584	0.68	0.70
GO:0045995	regulation of embryonic development	0.056 %	-1.0609	0.72	0.60
GO:0010492	maintenance of shoot apical meristem identity	0.056 %	-1.0153	0.80	0.72
GO:0046622	positive regulation of organ growth	0.017 %	-1.3622	0.70	0.81
GO:0010374	stomatal complex development	0.220 %	-1.1765	0.77	0.64
GO:0040034	regulation of development, heterochronic	0.224 %	-1.0589	0.74	0.62
GO:0048639	positive regulation of developmental growth	0.026 %	-1.3622	0.70	0.63
GO:0051093	negative regulation of developmental process	0.492 %	-1.0080	0.71	0.66
GO:0040019	positive regulation of embryonic development	0.009 %	-1.1467	0.72	0.60
GO:0050794	regulation of cellular process	22.244 %	-3.5666	0.77	0.46
GO:0019222	regulation of metabolic process	14.847 %	-2.7738	0.79	0.63

GO:0050789	*regulation of biological process*	*24.333 %*	*-2.7297*	*0.82*	*0.61*
GO:0015770	sucrose transport	0.086 %	-1.6795	0.84	0.46
GO:0015772	*oligosaccharide transport*	*0.091 %*	*-1.6795*	*0.84*	*0.72*
GO:0015766	*disaccharide transport*	*0.091 %*	*-1.6795*	*0.84*	*1.00*
GO:0034219	*carbohydrate transmembrane transport*	*0.332 %*	*-1.4781*	*0.82*	*0.79*
GO:0019605	butyrate metabolic process	0.009 %	-1.2883	0.87	0.49
GO:0006950	response to stress	14.156 %	-1.5890	0.89	0.49
GO:0006833	water transport	0.194 %	-1.5071	0.84	0.50
GO:0042044	fluid transport	0.194 %	-1.5071	0.84	0.50

Biological Process (218) Tag Clouds

Scatterplot & Table **Interactive Graph** **TreeMap**

GROUP 41

Tip: your resulting list of GO terms seems to be quite long. If you want to reduce it further, press the Back button in your browser and choose a different setting for the "allowed similarity" parameter.

Hide/show dispensable GO terms

Export results to text table (CSV)

Make R script for plotting

term ID	description	frequency	pin?	$\log_{10}$ p-value	uniqueness	dispensability
GO:0006629	lipid metabolic process	4.683 %		-3.6879	0.81	0.00
GO:0032501	multicellular organismal process	11.360 %		-2.2702	0.99	0.00
GO:0032502	developmental process	12.689 %		-2.7214	0.99	0.00
GO:0044699	single-organism process	41.709 %		-3.3980	0.99	0.00
GO:0048522	positive regulation of cellular process	3.582 %		-4.0461	0.67	0.00
GO:0071229	*cellular response to acid chemical*	*2.158 %*		*-1.8463*	*0.76*	*0.75*
GO:1901701	*cellular response to oxygen-containing compound*	*2.788 %*		*-1.8880*	*0.75*	*0.69*
GO:0045862	*positive regulation of proteolysis*	*0.272 %*		*-1.6421*	*0.66*	*0.84*
GO:0023056	*positive regulation of signaling*	*0.332 %*		*-1.6550*	*0.68*	*0.66*
GO:0097306	*cellular response to alcohol*	*1.170 %*		*-2.3931*	*0.75*	*0.80*
GO:1902680	*positive regulation of RNA biosynthetic process*	*1.869 %*		*-2.4870*	*0.61*	*0.97*
GO:0031331	*positive regulation of cellular catabolic process*	*0.289 %*		*-1.6421*	*0.66*	*0.98*
GO:0031328	*positive regulation of cellular biosynthetic process*	*2.102 %*		*-3.5418*	*0.62*	*0.83*
GO:0031325	*positive regulation of cellular metabolic process*	*2.741 %*		*-3.8386*	*0.64*	*0.92*
GO:0048584	*positive regulation of response to stimulus*	*0.902 %*		*-1.3173*	*0.62*	*0.74*
GO:0051254	*positive regulation of RNA metabolic process*	*1.912 %*		*-2.4870*	*0.63*	*0.96*

GO:0045935	positive regulation of nucleobase-containing compound metabolic process	2.007 %	-3.0974	0.63	0.90
GO:0010647	positive regulation of cell communication	0.350 %	-1.6194	0.70	0.66
GO:1901800	positive regulation of proteasomal protein catabolic process	0.259 %	-1.6421	0.65	0.98
GO:0032870	cellular response to hormone stimulus	3.871 %	-1.2696	0.73	0.82
GO:0010628	positive regulation of gene expression	2.050 %	-2.3195	0.65	0.89
GO:0045893	positive regulation of transcription, DNA-templated	1.864 %	-2.4870	0.61	0.96
GO:0071215	cellular response to abscisic acid stimulus	1.170 %	-2.3931	0.74	0.98
GO:0010604	positive regulation of macromolecule metabolic process	2.672 %	-3.2104	0.65	0.95
GO:0071365	cellular response to auxin stimulus	0.932 %	-1.5893	0.77	0.85
GO:0071396	cellular response to lipid	1.770 %	-1.4499	0.75	0.77
GO:0010557	positive regulation of macromolecule biosynthetic process	2.007 %	-2.9536	0.63	0.97
GO:0032436	positive regulation of proteasomal ubiquitin-dependent protein catabolic process	0.207 %	-1.6421	0.65	0.63
GO:0032434	regulation of proteasomal ubiquitin-dependent protein catabolic process	0.211 %	-1.6421	0.72	0.96
GO:0071310	cellular response to organic substance	4.601 %	-1.0981	0.74	0.88
GO:0061136	regulation of proteasomal protein catabolic process	0.268 %	-1.6421	0.72	0.99
GO:1903364	positive regulation of cellular protein catabolic process	0.263 %	-1.6421	0.65	0.99
GO:1903362	regulation of cellular protein catabolic process	0.272 %	-1.6421	0.72	0.95
GO:0009741	response to brassinosteroid	0.410 %	-1.8128	0.82	0.66
GO:0009738	abscisic acid-activated signaling pathway	1.062 %	-2.1958	0.60	0.65
GO:0009734	auxin-activated signaling pathway	0.868 %	-1.8392	0.62	0.69
GO:1901485	positive regulation of transcription factor catabolic process	0.004 %	-1.6421	0.72	0.76
GO:1901483	regulation of transcription factor catabolic process	0.004 %	-1.6421	0.78	1.00
GO:0030162	regulation of proteolysis	0.591 %	-1.1307	0.75	0.67
GO:0009789	positive regulation of abscisic acid-activated signaling pathway	0.147 %	-2.2114	0.55	0.93
GO:0009787	regulation of abscisic acid-activated signaling pathway	0.350 %	-2.0813	0.59	0.93
GO:1903508	positive regulation of nucleic acid-templated transcription	1.869 %	-2.4870	0.61	0.98
GO:0009893	positive regulation of metabolic process	2.892 %	-3.5466	0.67	0.87
GO:0009891	positive regulation of biosynthetic process	2.145 %	-3.4989	0.64	0.93
GO:1903052	positive regulation of proteolysis involved in cellular protein catabolic process	0.263 %	-1.6421	0.65	1.00
GO:1903050	regulation of proteolysis involved in cellular protein catabolic process	0.272 %	-1.6421	0.72	1.00
GO:0009967	positive regulation of signal transduction	0.332 %	-1.6550	0.57	0.93
GO:0009966	regulation of signal transduction	1.109 %	-1.1160	0.61	0.87
GO:0051173	positive regulation of nitrogen compound metabolic process	2.119 %	-3.5787	0.66	0.92
GO:1901419	regulation of response to alcohol	0.350 %	-2.0813	0.68	0.71
GO:1901421	positive regulation of response to alcohol	0.147 %	-2.2114	0.62	0.61
GO:0050896	response to stimulus	25.943 %	-2.7405	0.99	0.00
GO:0051704	multi-organism process	3.362 %	-1.5330	0.99	0.00
GO:0046209	nitric oxide metabolic process	0.030 %	-1.0635	0.91	0.05
GO:0006809	nitric oxide biosynthetic process	0.022 %	-1.1748	0.88	0.75
GO:0042126	nitrate metabolic process	0.181 %	-1.0061	0.78	0.98
GO:0042128	nitrate assimilation	0.181 %	-1.0061	0.78	0.86
GO:0010166	wax metabolic process	0.082 %	-1.4134	0.96	0.05
GO:0036369	transcription factor catabolic process	0.004 %	-1.6421	0.90	0.06
GO:0006723	cuticle hydrocarbon biosynthetic process	0.013 %	-2.1159	0.84	0.08
GO:1901615	organic hydroxy compound metabolic process	1.265 %	-1.6061	0.94	0.10
GO:1901642	nucleoside transmembrane transport	0.069 %	-2.4161	0.90	0.10
GO:0015858	nucleoside transport	0.134 %	-1.7205	0.90	0.83
GO:0009626	plant-type hypersensitive response	0.341 %	-3.4615	0.72	0.12
GO:0043067	regulation of programmed cell death	0.216 %	-1.1076	0.75	0.86
GO:0012501	programmed cell death	0.600 %	-2.4846	0.83	0.90
GO:0010363	regulation of plant-type hypersensitive response	0.060 %	-2.2211	0.66	0.86
GO:0034050	host programmed cell death induced by symbiont	0.345 %	-3.3593	0.84	0.90
GO:0034052	positive regulation of plant-type hypersensitive response	0.026 %	-1.3641	0.61	0.81
GO:0010025	wax biosynthetic process	0.078 %	-1.4745	0.93	0.13
GO:0010039	response to iron ion	0.237 %	-1.9927	0.85	0.17
GO:0042542	response to hydrogen peroxide	0.268 %	-1.9348	0.82	0.53
GO:0000302	response to reactive oxygen species	0.678 %	-1.4099	0.81	0.71
GO:0031122	cytoplasmic microtubule organization	0.216 %	-1.3754	0.86	0.19
GO:0043622	cortical microtubule organization	0.112 %	-1.3941	0.87	0.81
GO:0030865	cortical cytoskeleton organization	0.129 %	-1.3527	0.95	0.71
GO:2000278	regulation of DNA biosynthetic process	0.022 %	-1.6421	0.78	0.20
GO:0071897	DNA biosynthetic process	0.315 %	-1.3830	0.86	0.50
GO:0051973	positive regulation of telomerase activity	0.009 %	-1.6795	0.72	0.92
GO:2000573	positive regulation of DNA biosynthetic process	0.013 %	-1.6795	0.71	0.66
GO:0051972	regulation of telomerase activity	0.013 %	-1.6795	0.78	0.94
GO:0051054	positive regulation of DNA metabolic process	0.086 %	-1.3287	0.69	0.72
GO:0006069	ethanol oxidation	0.004 %	-1.3830	0.87	0.21
GO:0006067	ethanol metabolic process	0.004 %	-1.3830	0.87	0.75
GO:0006066	alcohol metabolic process	0.505 %	-1.0345	0.82	0.56
GO:0048513	animal organ development	0.004 %	-1.2116	0.89	0.21

GO:0008219	cell death	0.734 %	-1.8332	0.86	0.21
GO:0009821	alkaloid biosynthetic process	0.795 %	-1.1748	0.86	0.22
GO:1901804	beta-glucoside metabolic process	0.009 %	-1.5188	0.88	0.22
GO:1901038	cyanidin 3-O-glucoside metabolic process	0.004 %	-1.5188	0.87	0.89
GO:0048730	epidermis morphogenesis	0.033 %	-1.9407	0.85	0.22
GO:0002009	morphogenesis of an epithelium	0.013 %	-1.1522	0.86	1.06
GO:0007017	microtubule-based process	0.859 %	-1.0015	0.86	0.23
GO:0043446	cellular alkane metabolic process	0.017 %	-1.4685	0.86	0.24
GO:0043901	negative regulation of multi-organism process	0.393 %	-1.1867	0.83	0.24
GO:0043900	regulation of multi-organism process	0.190 %	-1.1838	0.85	0.56
GO:0042592	homeostatic process	2.637 %	-3.2171	0.80	0.26
GO:0055088	lipid homeostasis	0.199 %	-1.0910	0.81	0.65
GO:0055080	cation homeostasis	1.083 %	-1.7561	0.78	0.92
GO:0055078	sodium ion homeostasis	0.030 %	-1.6421	0.83	0.67
GO:0055076	transition metal ion homeostasis	0.678 %	-1.8161	0.79	0.86
GO:0055072	iron ion homeostasis	0.237 %	-2.7241	0.80	0.55
GO:0055065	metal ion homeostasis	0.842 %	-1.8511	0.79	0.92
GO:0019725	cellular homeostasis	1.549 %	-2.1745	0.70	0.69
GO:0006879	cellular iron ion homeostasis	0.108 %	-2.3927	0.74	0.77
GO:0050801	ion homeostasis	1.299 %	-1.4177	0.78	0.83
GO:0046916	cellular transition metal ion homeostasis	0.505 %	-1.3941	0.71	0.92
GO:0045454	cell redox homeostasis	0.634 %	-2.1557	0.70	0.77
GO:0048878	chemical homeostasis	1.765 %	-1.8799	0.78	0.86
GO:0010268	brassinosteroid homeostasis	0.138 %	-1.1989	0.82	0.63
GO:0098771	inorganic ion homeostasis	1.139 %	-1.5845	0.78	0.95
GO:0010345	suberin biosynthetic process	0.060 %	-1.7561	0.79	0.26
GO:0052318	regulation of phytoalexin metabolic process	0.009 %	-1.6078	0.75	0.81
GO:0052319	regulation of phytoalexin biosynthetic process	0.009 %	-1.6078	0.72	1.00
GO:0052320	positive regulation of phytoalexin metabolic process	0.009 %	-1.6078	0.68	1.00
GO:0052322	positive regulation of phytoalexin biosynthetic process	0.009 %	-1.6078	0.66	1.00
GO:1901182	regulation of camalexin biosynthetic process	0.009 %	-1.6078	0.71	1.00
GO:1901183	positive regulation of camalexin biosynthetic process	0.009 %	-1.6078	0.65	0.52
GO:1900378	positive regulation of secondary metabolite biosynthetic process	0.017 %	-1.3287	0.67	0.79
GO:0051176	positive regulation of sulfur metabolic process	0.035 %	-1.4236	0.73	0.81
GO:0006826	iron ion transport	0.121 %	-2.3101	0.96	0.27
GO:0000041	transition metal ion transport	0.358 %	-1.3350	0.97	0.73
GO:0030001	metal ion transport	1.588 %	-1.3818	0.96	0.66
GO:0023051	regulation of signaling	1.126 %	-1.1013	0.81	0.27
GO:0065008	regulation of biological quality	5.991 %	-1.5573	0.83	0.29
GO:0043447	alkane biosynthetic process	0.017 %	-1.4685	0.83	0.30
GO:0010646	regulation of cell communication	1.139 %	-1.0940	0.79	0.30
GO:0048583	regulation of response to stimulus	2.521 %	-1.2223	0.74	0.31
GO:0048229	gametophyte development	1.653 %	-1.7995	0.83	0.31
GO:0044707	single-multicellular organism process	10.699 %	-1.8946	0.84	0.86
GO:0044767	single-organism developmental process	12.123 %	-2.9701	0.81	0.68
GO:0007275	multicellular organism development	10.423 %	-2.0946	0.80	0.82
GO:0009653	anatomical structure morphogenesis	3.500 %	-1.2226	0.89	0.51
GO:0048856	anatomical structure development	11.722 %	-2.9275	0.88	0.85
GO:0048869	cellular developmental process	4.148 %	-1.2169	0.76	0.55
GO:0009820	alkaloid metabolic process	0.009 %	-1.0910	0.94	0.32
GO:0048518	positive regulation of biological process	4.398 %	-2.4343	0.81	0.33
GO:0010231	maintenance of seed dormancy	0.035 %	-1.7205	0.86	0.34
GO:0097437	maintenance of dormancy	0.035 %	-1.7205	0.88	0.82
GO:0019438	aromatic compound biosynthetic process	14.247 %	-1.2404	0.84	0.34
GO:1901362	organic cyclic compound biosynthetic process	14.769 %	-1.2002	0.86	0.55
GO:0009718	anthocyanin-containing compound biosynthetic process	0.121 %	-1.4232	0.81	0.34
GO:0046148	pigment biosynthetic process	0.639 %	-1.0083	0.84	0.85
GO:0046283	anthocyanin-containing compound metabolic process	0.190 %	-1.0235	0.85	0.84
GO:0009409	response to cold	1.580 %	-2.6751	0.85	0.34
GO:0009651	response to salt stress	2.270 %	-1.2179	0.85	0.62
GO:0009266	response to temperature stimulus	2.287 %	-1.7685	0.86	0.59
GO:0006970	response to osmotic stress	2.512 %	-1.6611	0.84	0.62
GO:0006950	response to stress	14.156 %	-2.7376	0.84	0.35
GO:0080170	hydrogen peroxide transmembrane transport	0.022 %	-1.0138	0.97	0.36
GO:0048645	animal organ formation	0.004 %	-1.8826	0.87	0.36
GO:0010160	formation of animal organ boundary	0.055 %	-1.0463	0.83	1.15
GO:0009887	animal organ morphogenesis	0.004 %	-1.5710	0.87	1.00
GO:0042991	transcription factor import into nucleus	0.026 %	-1.4929	0.91	0.36
GO:0009733	response to auxin	1.709 %	-1.9255	0.81	0.36
GO:1901700	response to oxygen-containing compound	6.504 %	-1.8107	0.80	0.54
GO:0010033	response to organic substance	8.580 %	-1.4584	0.79	0.70
GO:0009725	response to hormone	6.871 %	-1.1996	0.77	0.82

GO:0071495	cellular response to endogenous stimulus	3.988 %	-1.2280	0.82	0.66
GO:0042335	cuticle development	0.082 %	-1.7108	0.87	0.36
GO:0006631	fatty acid metabolic process	1.101 %	-3.5780	0.76	0.37
GO:0032787	monocarboxylic acid metabolic process	2.128 %	-2.0955	0.76	0.71
GO:0072330	monocarboxylic acid biosynthetic process	1.101 %	-2.5041	0.73	0.84
GO:0016053	organic acid biosynthetic process	2.296 %	-1.1249	0.72	0.92
GO:0046394	carboxylic acid biosynthetic process	2.296 %	-1.3787	0.72	0.89
GO:0044255	cellular lipid metabolic process	3.224 %	-2.1641	0.77	0.73
GO:0008610	lipid biosynthetic process	2.464 %	-1.9866	0.76	0.82
GO:0010430	fatty acid omega-oxidation	0.004 %	-1.6795	0.83	0.52
GO:0043436	oxoacid metabolic process	5.002 %	-1.0479	0.74	0.83
GO:0008202	steroid metabolic process	0.427 %	-1.1201	0.81	0.59
GO:0042304	regulation of fatty acid biosynthetic process	0.052 %	-1.0548	0.68	0.90
GO:0006633	fatty acid biosynthetic process	0.682 %	-3.6487	0.73	0.79
GO:0042761	very long-chain fatty acid biosynthetic process	0.056 %	-1.0379	0.77	0.63
GO:0080140	regulation of jasmonic acid metabolic process	0.017 %	-1.1867	0.72	0.57
GO:0080141	regulation of jasmonic acid biosynthetic process	0.013 %	-1.1989	0.71	0.83
GO:0045490	pectin catabolic process	0.423 %	-1.4183	0.89	0.37
GO:0010410	hemicellulose metabolic process	0.423 %	-1.0714	0.91	0.71
GO:0010305	leaf vascular tissue pattern formation	0.117 %	-1.3571	0.85	0.37
GO:0003002	regionalization	0.617 %	-1.0531	0.83	0.88
GO:0010051	xylem and phloem pattern formation	0.259 %	-1.2143	0.84	0.80
GO:0006811	ion transport	4.174 %	-1.1081	0.96	0.38
GO:0009607	response to biotic stimulus	5.158 %	-1.5507	0.86	0.41
GO:0010068	protoderm histogenesis	0.004 %	-1.5188	0.87	0.41
GO:0010076	maintenance of floral meristem identity	0.026 %	-1.3830	0.85	0.50
GO:0010077	maintenance of inflorescence meristem identity	0.039 %	-1.2516	0.85	0.80
GO:0009645	response to low light intensity stimulus	0.073 %	-1.0463	0.90	0.41
GO:0006979	response to oxidative stress	1.908 %	-1.6253	0.86	0.41
GO:0010182	sugar mediated signaling pathway	0.147 %	-1.4382	0.67	0.42
GO:0071322	cellular response to carbohydrate stimulus	0.220 %	-1.1694	0.79	0.83
GO:0009756	carbohydrate mediated signaling	0.147 %	-1.4382	0.67	0.94
GO:0009605	response to external stimulus	6.366 %	-1.4519	0.85	0.42
GO:1901264	carbohydrate derivative transport	0.306 %	-1.2659	0.90	0.43
GO:1900424	regulation of defense response to bacterium	0.129 %	-2.3586	0.73	0.43
GO:0043207	response to external biotic stimulus	4.981 %	-1.5774	0.83	0.94
GO:0051707	response to other organism	4.968 %	-1.5774	0.83	0.64
GO:0002832	negative regulation of response to biotic stimulus	0.065 %	-1.1867	0.75	0.83
GO:0002831	regulation of response to biotic stimulus	0.293 %	-2.0661	0.73	0.57
GO:1900425	negative regulation of defense response to bacterium	0.043 %	-1.5760	0.74	0.81
GO:0032101	regulation of response to external stimulus	0.315 %	-1.9676	0.73	0.62
GO:0032102	negative regulation of response to external stimulus	0.065 %	-1.1867	0.75	0.82
GO:0080135	regulation of cellular response to stress	0.250 %	-1.5412	0.70	0.67
GO:1901698	response to nitrogen compound	1.174 %	-1.3934	0.83	0.43
GO:0048729	tissue morphogenesis	0.013 %	-1.1522	0.86	0.43
GO:0007267	cell-cell signaling	0.371 %	-1.0725	0.83	0.44
GO:0009719	response to endogenous stimulus	7.359 %	-1.8220	0.85	0.44
GO:0009628	response to abiotic stimulus	7.946 %	-2.0543	0.85	0.45
GO:0009555	pollen development	1.230 %	-1.9741	0.84	0.48
GO:0046473	phosphatidic acid metabolic process	0.009 %	-1.3121	0.85	0.48
GO:0010167	response to nitrate	0.078 %	-1.4037	0.84	0.48
GO:0010200	response to chitin	0.544 %	-1.3547	0.81	0.71
GO:0010243	response to organonitrogen compound	0.768 %	-1.0062	0.81	0.87
GO:0006952	defense response	6.090 %	-1.0742	0.84	0.50
GO:0010103	stomatal complex morphogenesis	0.125 %	-1.9397	0.83	0.50
GO:0008544	epidermis development	0.069 %	-1.5649	0.90	0.53
GO:0060429	epithelium development	0.082 %	-1.4535	0.89	0.53
GO:0010052	guard cell differentiation	0.082 %	-1.2377	0.79	0.89
GO:2000122	negative regulation of stomatal complex development	0.022 %	-1.8741	0.75	0.81
GO:0010374	stomatal complex development	0.220 %	-1.2490	0.83	0.71

Biological Process (229) Tag Clouds

Scatterplot & Table Interactive Graph TreeMap

GROUP 42

Tip: your resulting list of GO terms seems to be quite long. If you want to reduce it further, press the Back button in your browser and choose a different setting for the "allowed similarity" parameter.

Hide/show dispensable GO terms

Export results to text table (CSV)

Make R script for plotting

term ID	description	frequency	pin?	$\log_{10}$ p-value	uniqueness	dispensability
GO:0000380	alternative mRNA splicing, via spliceosome	0.091 %		-5.8197	0.89	0.00
GO:0000377	RNA splicing, via transesterification reactions with bulged adenosine as nucleophile	0.785 %		-2.9369	0.87	0.98
GO:0000375	RNA splicing, via transesterification reactions	0.807 %		-2.9369	0.87	0.88
GO:0000398	mRNA splicing, via spliceosome	0.729 %		-3.4171	0.87	0.81
GO:0008380	RNA splicing	1.312 %		-2.5339	0.87	0.56
GO:0006397	mRNA processing	1.692 %		-2.3485	0.87	0.84
GO:0009639	response to red or far red light	0.872 %		-4.6765	0.79	0.00
GO:0009582	detection of abiotic stimulus	0.311 %		-1.6061	0.81	0.94
GO:0009581	detection of external stimulus	0.311 %		-1.6061	0.83	0.93
GO:0009584	detection of visible light	0.022 %		-1.3527	0.81	0.82
GO:0009583	detection of light stimulus	0.268 %		-2.0115	0.78	0.97
GO:0009585	red, far-red light phototransduction	0.263 %		-2.2874	0.63	0.80
GO:0010114	response to red light	0.259 %		-2.4187	0.80	0.80
GO:0010161	red light signaling pathway	0.035 %		-2.2719	0.70	0.81
GO:0071214	cellular response to abiotic stimulus	0.889 %		-1.3618	0.76	0.56
GO:0009641	shade avoidance	0.069 %		-4.3653	0.81	0.58

GO:0009640	photomorphogenesis	0.311 %	-2.8883	0.67	0.72
GO:0009704	de-etiolation	0.047 %	-2.0227	0.71	0.63
GO:0010017	red or far-red light signaling pathway	0.341 %	-1.3098	0.66	0.97
GO:0007602	phototransduction	0.263 %	-2.2874	0.66	0.99
GO:0009314	response to radiation	2.892 %	-1.7813	0.80	0.56
GO:0009416	response to light stimulus	2.771 %	-1.8170	0.77	0.83
GO:0071489	cellular response to red or far red light	0.350 %	-1.2927	0.73	0.89
GO:0071491	cellular response to red light	0.043 %	-2.2126	0.77	0.82
GO:0023052	signaling	8.908 %	-2.3423	0.99	0.00
GO:0032501	multicellular organismal process	11.360 %	-1.0851	0.99	0.00
GO:0032502	developmental process	12.689 %	-1.0433	0.99	0.00
GO:0044699	single-organism process	41.709 %	-1.6880	0.99	0.00
GO:0050896	response to stimulus	25.943 %	-2.6783	0.99	0.00
GO:0065007	biological regulation	28.554 %	-1.1077	0.99	0.00
GO:0071166	ribonucleoprotein complex localization	0.224 %	-3.2135	0.91	0.00
GO:0051169	nuclear transport	0.470 %	-2.7544	0.90	0.59
GO:0032508	DNA duplex unwinding	0.255 %	-3.7641	0.94	0.03
GO:0032392	DNA geometric change	0.255 %	-3.7171	0.94	0.87
GO:0071103	DNA conformation change	0.539 %	-1.8974	0.93	0.79
GO:0007154	cell communication	9.698 %	-2.2979	0.94	0.05
GO:0009061	anaerobic respiration	0.047 %	-2.8206	0.84	0.06
GO:0045333	cellular respiration	0.514 %	-1.4904	0.81	0.79
GO:0015980	energy derivation by oxidation of organic compounds	0.604 %	-1.3278	0.81	0.80
GO:0009850	auxin metabolic process	0.332 %	-1.2598	0.87	0.06
GO:0080024	indolebutyric acid metabolic process	0.022 %	-1.0106	0.77	0.70
GO:0032350	regulation of hormone metabolic process	0.073 %	-1.0436	0.89	0.76
GO:0010600	regulation of auxin biosynthetic process	0.052 %	-1.3913	0.85	0.74
GO:0046885	regulation of hormone biosynthetic process	0.065 %	-1.1636	0.85	0.97
GO:0090354	regulation of auxin metabolic process	0.056 %	-1.2001	0.87	0.96
GO:0048444	floral organ morphogenesis	0.181 %	-2.1730	0.80	0.07
GO:0048532	anatomical structure arrangement	0.289 %	-1.4476	0.82	0.60
GO:0010228	vegetative to reproductive phase transition of meristem	0.738 %	-1.2785	0.82	0.52
GO:0010094	specification of carpel identity	0.013 %	-1.6679	0.81	0.90
GO:0010093	specification of floral organ identity	0.056 %	-2.1057	0.79	0.79
GO:0048462	carpel formation	0.026 %	-1.4121	0.81	0.93
GO:0048446	petal morphogenesis	0.026 %	-1.1754	0.82	0.80
GO:0048445	carpel morphogenesis	0.035 %	-1.1409	0.82	0.82
GO:0048449	floral organ formation	0.104 %	-1.5867	0.80	0.88
GO:0010016	shoot system morphogenesis	0.708 %	-1.0784	0.80	0.65
GO:1905392	plant organ morphogenesis	1.485 %	-1.0200	0.79	0.71
GO:0010358	leaf shaping	0.035 %	-1.0704	0.83	0.58
GO:0048856	anatomical structure development	11.722 %	-1.0632	0.87	0.72
GO:0009791	post-embryonic development	5.848 %	-1.4226	0.80	0.52
GO:0090697	post-embryonic plant organ morphogenesis	0.419 %	-1.6720	0.79	0.84
GO:0090698	post-embryonic plant morphogenesis	0.716 %	-1.6957	0.86	0.58
GO:0090696	post-embryonic plant organ development	0.686 %	-1.0946	0.81	0.56
GO:0017006	protein-tetrapyrrole linkage	0.030 %	-1.3527	0.93	0.09
GO:0044763	single-organism cellular process	23.034 %	-1.2967	0.85	0.13
GO:0080028	nitrile biosynthetic process	0.022 %	-2.2288	0.90	0.13
GO:1901334	lactone metabolic process	0.026 %	-1.2693	0.93	0.15
GO:0080037	negative regulation of cytokinin-activated signaling pathway	0.022 %	-4.6370	0.73	0.15
GO:0080036	regulation of cytokinin-activated signaling pathway	0.047 %	-3.6001	0.72	0.85
GO:0071368	cellular response to cytokinin stimulus	0.337 %	-3.7008	0.76	0.64
GO:0009736	cytokinin-activated signaling pathway	0.328 %	-4.0196	0.69	0.80
GO:0010469	regulation of receptor activity	0.009 %	-1.0798	0.78	0.50
GO:0009966	regulation of signal transduction	1.109 %	-1.6064	0.70	0.69
GO:0010119	regulation of stomatal movement	0.281 %	-1.1088	0.82	0.17
GO:0031539	positive regulation of anthocyanin metabolic process	0.022 %	-1.5073	0.84	0.17
GO:0010921	regulation of phosphatase activity	0.035 %	-1.0704	0.87	0.20
GO:0043666	regulation of phosphoprotein phosphatase activity	0.022 %	-1.0893	0.87	0.93
GO:0080163	regulation of protein serine/threonine phosphatase activity	0.017 %	-1.1754	0.87	0.89
GO:0008299	isoprenoid biosynthetic process	0.738 %	-2.7224	0.70	0.22
GO:0033383	geranyl diphosphate metabolic process	0.056 %	-1.2402	0.74	0.76
GO:0043692	monoterpene metabolic process	0.017 %	-1.5962	0.77	0.89
GO:0043693	monoterpene biosynthetic process	0.017 %	-1.5962	0.76	0.89
GO:0044255	cellular lipid metabolic process	3.224 %	-1.6332	0.72	0.82
GO:0016099	monoterpenoid biosynthetic process	0.004 %	-1.3715	0.78	0.63
GO:0016098	monoterpenoid metabolic process	0.004 %	-1.3715	0.79	0.63
GO:0016103	diterpenoid catabolic process	0.017 %	-1.4814	0.75	0.88
GO:0016102	diterpenoid biosynthetic process	0.147 %	-1.8514	0.72	0.97

GO:0016101	diterpenoid metabolic process	0.173 %	-1.6674	0.74	0.84
GO:0016106	sesquiterpenoid biosynthetic process	0.138 %	-2.7087	0.73	0.82
GO:0016114	terpenoid biosynthetic process	0.591 %	-2.2646	0.70	0.94
GO:1901601	strigolactone biosynthetic process	0.026 %	-1.2693	0.74	0.85
GO:1901600	strigolactone metabolic process	0.026 %	-1.2693	0.75	1.00
GO:0009686	gibberellin biosynthetic process	0.134 %	-1.8605	0.71	0.82
GO:0008610	lipid biosynthetic process	2.464 %	-1.9264	0.74	0.69
GO:0009685	gibberellin metabolic process	0.138 %	-1.7740	0.71	0.97
GO:0006714	sesquiterpenoid metabolic process	0.181 %	-2.5014	0.73	0.84
GO:0006720	isoprenoid metabolic process	0.850 %	-2.4169	0.74	0.69
GO:0006721	terpenoid metabolic process	0.699 %	-1.9780	0.71	0.96
GO:0042214	terpene metabolic process	0.065 %	-2.0227	0.75	0.77
GO:0045487	gibberellin catabolic process	0.017 %	-1.4814	0.74	0.86
GO:0033559	unsaturated fatty acid metabolic process	0.112 %	-1.0798	0.75	0.65
GO:0006636	unsaturated fatty acid biosynthetic process	0.108 %	-1.0798	0.74	0.62
GO:0046246	terpene biosynthetic process	0.043 %	-2.3874	0.75	0.75
GO:1901336	lactone biosynthetic process	0.026 %	-1.2693	0.90	1.00
GO:0010501	RNA secondary structure unwinding	0.281 %	-3.8639	0.90	0.22
GO:0050832	defense response to fungus	2.236 %	-2.3404	0.82	0.23
GO:0009606	tropism	0.341 %	-1.1998	0.85	0.54
GO:0009620	response to fungus	2.521 %	-2.0697	0.82	0.76
GO:0006952	defense response	6.090 %	-2.3983	0.83	0.51
GO:0006662	glycerol ether metabolic process	0.138 %	-2.0811	0.83	0.24
GO:0010034	response to acetate	0.004 %	-1.3715	0.87	0.24
GO:0018298	protein-chromophore linkage	0.203 %	-1.2130	0.92	0.25
GO:0050898	nitrile metabolic process	0.022 %	-2.2288	0.93	0.27
GO:0080160	selenate transport	0.077 %	-1.3007	0.92	0.28
GO:0001708	cell fate specification	0.134 %	-1.7578	0.80	0.29
GO:0010158	abaxial cell fate specification	0.030 %	-1.2847	0.82	0.77
GO:0045165	cell fate commitment	0.341 %	-1.4857	0.79	0.53
GO:0016071	mRNA metabolic process	2.145 %	-1.5735	0.89	0.30
GO:1990267	response to transition metal nanoparticle	2.017 %	-2.8735	0.81	0.30
GO:0018874	benzoate metabolic process	0.009 %	-1.1301	0.83	0.30
GO:0009719	response to endogenous stimulus	7.359 %	-2.3972	0.83	0.31
GO:0006403	RNA localization	0.367 %	-3.0563	0.92	0.31
GO:0071311	cellular response to acetate	0.004 %	-1.3715	0.83	0.32
GO:0050657	nucleic acid transport	0.332 %	-3.0563	0.90	0.32
GO:0051236	establishment of RNA localization	0.332 %	-3.0563	0.90	0.97
GO:0008643	carbohydrate transport	0.630 %	-1.3637	0.83	0.51
GO:0015931	nucleobase-containing compound transport	0.561 %	-2.6811	0.90	0.76
GO:0050658	RNA transport	0.332 %	-3.0563	0.89	0.99
GO:0051028	mRNA transport	0.293 %	-3.2602	0.89	0.95
GO:0006405	RNA export from nucleus	0.203 %	-3.3254	0.87	0.87
GO:0006406	mRNA export from nucleus	0.138 %	-3.5859	0.83	0.92
GO:0071426	ribonucleoprotein complex export from nucleus	0.224 %	-3.2135	0.89	0.96
GO:0071427	mRNA-containing ribonucleoprotein complex export from nucleus	0.138 %	-3.5859	0.90	0.92
GO:0006913	nucleocytoplasmic transport	0.466 %	-2.7544	0.89	0.92
GO:0051168	nuclear export	0.281 %	-2.7616	0.89	0.95
GO:0010646	regulation of cell communication	1.139 %	-1.5774	0.87	0.33
GO:0023051	regulation of signaling	1.126 %	-1.5870	0.90	0.34
GO:0044700	single organism signaling	8.899 %	-2.3462	0.83	0.91
GO:0070887	cellular response to chemical stimulus	5.287 %	-2.3525	0.74	0.67
GO:0007165	signal transduction	8.731 %	-2.3715	0.64	0.64
GO:0010033	response to organic substance	8.580 %	-1.9402	0.78	0.68
GO:0006741	NADP biosynthetic process	0.013 %	-1.7542	0.79	0.34
GO:0019363	pyridine nucleotide biosynthetic process	0.056 %	-1.1195	0.77	0.91
GO:0019359	nicotinamide nucleotide biosynthetic process	0.043 %	-1.1875	0.78	0.90
GO:0019674	NAD metabolic process	0.078 %	-1.1636	0.79	0.66
GO:0006629	lipid metabolic process	4.683 %	-1.2547	0.80	0.35
GO:0050794	regulation of cellular process	22.244 %	-1.4931	0.87	0.36
GO:0050789	regulation of biological process	24.333 %	-1.3109	0.92	0.61
GO:0010043	response to zinc ion	0.186 %	-2.5339	0.84	0.36
GO:0071248	cellular response to metal ion	0.216 %	-1.1225	0.78	0.96
GO:0071281	cellular response to iron ion	0.177 %	-1.3684	0.78	0.61
GO:0010039	response to iron ion	0.237 %	-1.1160	0.84	0.63
GO:0019762	glucosinolate catabolic process	0.104 %	-1.9290	0.77	0.36
GO:0019760	glucosinolate metabolic process	0.514 %	-1.0845	0.76	0.86
GO:0019759	glycosinolate catabolic process	0.104 %	-1.9290	0.77	1.00
GO:0019757	glycosinolate metabolic process	0.514 %	-1.0845	0.76	0.99
GO:0018904	ether metabolic process	0.142 %	-2.0115	0.83	0.37
GO:0001944	vasculature development	0.108 %	-1.4121	0.84	0.37
GO:0072358	cardiovascular system development	0.108 %	-1.4121	0.84	1.00

GO:0072359	circulatory system development	0.108 %	-1.4121	0.85	0.37
GO:0009605	response to external stimulus	6.366 %	-1.0959	0.84	0.37
GO:0042493	response to drug	0.367 %	-1.0436	0.84	0.38
GO:0071705	nitrogen compound transport	1.515 %	-1.0911	0.92	0.38
GO:0042753	positive regulation of circadian rhythm	0.086 %	-1.0613	0.93	0.39
GO:0009649	entrainment of circadian clock	0.030 %	-1.0106	0.85	0.81
GO:0009628	response to abiotic stimulus	7.946 %	-1.8415	0.83	0.39
GO:0009611	response to wounding	0.816 %	-1.8699	0.86	0.39
GO:0010274	hydrotropism	0.009 %	-1.4339	0.89	0.39
GO:0071260	cellular response to mechanical stimulus	0.009 %	-1.3715	0.80	0.39
GO:0071475	cellular hyperosmotic salinity response	0.009 %	-1.1409	0.81	0.67
GO:0071474	cellular hyperosmotic response	0.013 %	-1.1409	0.80	0.51
GO:0005992	trehalose biosynthetic process	0.095 %	-1.8790	0.80	0.40
GO:0046351	disaccharide biosynthetic process	0.155 %	-1.3362	0.80	0.89
GO:0046487	glyoxylate metabolic process	0.035 %	-1.1521	0.82	0.72
GO:0006097	glyoxylate cycle	0.030 %	-1.1754	0.81	0.52
GO:0009312	oligosaccharide biosynthetic process	0.255 %	-1.1628	0.81	0.83
GO:0005991	trehalose metabolic process	0.104 %	-1.8424	0.82	0.85
GO:0071495	cellular response to endogenous stimulus	3.988 %	-2.8722	0.81	0.41
GO:0071230	cellular response to amino acid stimulus	0.095 %	-1.1195	0.79	0.59
GO:1901701	cellular response to oxygen-containing compound	2.788 %	-1.8122	0.73	0.82
GO:0032870	cellular response to hormone stimulus	3.871 %	-2.4927	0.71	0.75
GO:0071370	cellular response to gibberellin stimulus	0.354 %	-1.1985	0.76	0.89
GO:0071396	cellular response to lipid	1.770 %	-1.8899	0.74	0.81
GO:0071310	cellular response to organic substance	4.601 %	-2.6621	0.72	0.88
GO:0009755	hormone-mediated signaling pathway	3.617 %	-2.1015	0.62	0.94
GO:0009741	response to brassinosteroid	0.410 %	-2.5148	0.81	0.55
GO:0009740	gibberellic acid mediated signaling pathway	0.341 %	-1.3362	0.68	0.59
GO:0009735	response to cytokinin	1.010 %	-1.8701	0.81	0.62
GO:0009725	response to hormone	6.871 %	-2.5545	0.77	0.82
GO:0010476	gibberellin mediated signaling pathway	0.345 %	-1.2518	0.68	0.99
GO:0006879	cellular iron ion homeostasis	0.108 %	-1.0351	0.83	0.42
GO:0009631	cold acclimation	0.199 %	-1.3971	0.83	0.43
GO:0009409	response to cold	1.580 %	-1.0498	0.80	0.71
GO:0034614	cellular response to reactive oxygen species	0.207 %	-1.1912	0.77	0.43
GO:0034599	cellular response to oxidative stress	0.453 %	-1.0094	0.76	0.67
GO:0042221	response to chemical	12.434 %	-1.3467	0.82	0.43
GO:0006814	sodium ion transport	0.121 %	-1.1195	0.91	0.44
GO:0010623	programmed cell death involved in cell development	0.035 %	-1.1301	0.82	0.45
GO:0015749	monosaccharide transport	0.229 %	-1.3346	0.85	0.46
GO:0080167	response to karrikin	0.531 %	-2.7776	0.82	0.47
GO:0090701	specification of plant organ identity	0.060 %	-2.1057	0.82	0.47
GO:0080110	sporopollenin biosynthetic process	0.030 %	-1.2693	0.76	0.63
GO:0003002	regionalization	0.617 %	-1.0207	0.82	0.82
GO:0010159	specification of animal organ position	0.051 %	-2.1043	0.82	0.67
GO:1905393	plant organ formation	0.427 %	-1.0737	0.86	0.75
GO:0010305	leaf vascular tissue pattern formation	0.117 %	-1.3362	0.84	0.71
GO:1904062	regulation of cation transmembrane transport	0.022 %	-1.0798	0.88	0.47
GO:2001257	regulation of cation channel activity	0.009 %	-1.0798	0.87	0.69
GO:1901016	regulation of potassium ion transmembrane transporter activity	0.013 %	-1.0798	0.77	0.96
GO:1901979	regulation of inward rectifier potassium channel activity	0.009 %	-1.0798	0.68	0.94
GO:1901379	regulation of potassium ion transmembrane transport	0.017 %	-1.0798	0.78	0.94
GO:0043266	regulation of potassium ion transport	0.030 %	-1.0186	0.87	0.70
GO:0042991	transcription factor import into nucleus	0.026 %	-1.4814	0.84	0.47
GO:0051716	cellular response to stimulus	12.637 %	-1.3264	0.79	0.48
GO:0019433	triglyceride catabolic process	0.017 %	-1.6306	0.78	0.49
GO:0044242	cellular lipid catabolic process	0.315 %	-1.0231	0.75	0.63
GO:0046464	acylglycerol catabolic process	0.022 %	-1.3173	0.78	0.99
GO:0046461	neutral lipid catabolic process	0.022 %	-1.3173	0.78	0.76
GO:0046503	glycerolipid catabolic process	0.035 %	-1.3173	0.77	0.69
GO:0051276	chromosome organization	1.118 %	-1.2674	0.94	0.49
GO:0080027	response to herbivore	0.043 %	-2.3194	0.87	0.49
GO:0010201	response to continuous far red light stimulus by the high-irradiance response system	0.009 %	-1.0893	0.84	0.49
GO:0055122	response to very low light intensity stimulus	0.009 %	-1.6679	0.85	0.49
GO:0010203	response to very low fluence red light stimulus	0.004 %	-1.8050	0.84	0.53
GO:0009647	skotomorphogenesis	0.026 %	-1.1301	0.73	0.58
GO:0010450	inflorescence meristem growth	0.013 %	-1.5645	0.85	0.49
GO:0035266	meristem growth	0.216 %	-1.0259	0.85	0.61
GO:0033528	S-methylmethionine cycle	0.004 %	-1.7542	0.91	0.50
GO:0033477	S-methylmethionine metabolic process	0.004 %	-1.7542	0.91	0.58
GO:0033993	response to lipid	3.267 %	-2.1991	0.79	0.50
